# Ultrafast, Selective, and Highly Sensitive Nonchromatographic Analysis of Fourteen Cannabinoids in Cannabis Extracts, Δ8-Tetrahydrocannabinol Synthetic Mixtures, and Edibles by Cyclic Ion Mobility Spectrometry–Mass Spectrometry

**DOI:** 10.1021/acs.analchem.3c05879

**Published:** 2024-06-11

**Authors:** Si Huang, Laura Righetti, Frank W. Claassen, Akash Krishna, Ming Ma, Teris A. van Beek, Bo Chen, Han Zuilhof, Gert IJ. Salentijn

**Affiliations:** † Key Laboratory of Phytochemical R&D of Hunan Province and Key Laboratory of Chemical Biology & Traditional Chinese Medicine Research of Ministry of Education, Hunan Normal University, No.36, Lushan Road, Changsha 410081, China; ‡ Laboratory of Organic Chemistry, Wageningen University, Stippeneng 4, Wageningen 6708 WE, The Netherlands; § Wageningen Food Safety Research (WFSR), Wageningen University & Research, P.O. Box 230, Wageningen 6700 AE, The Netherlands

## Abstract

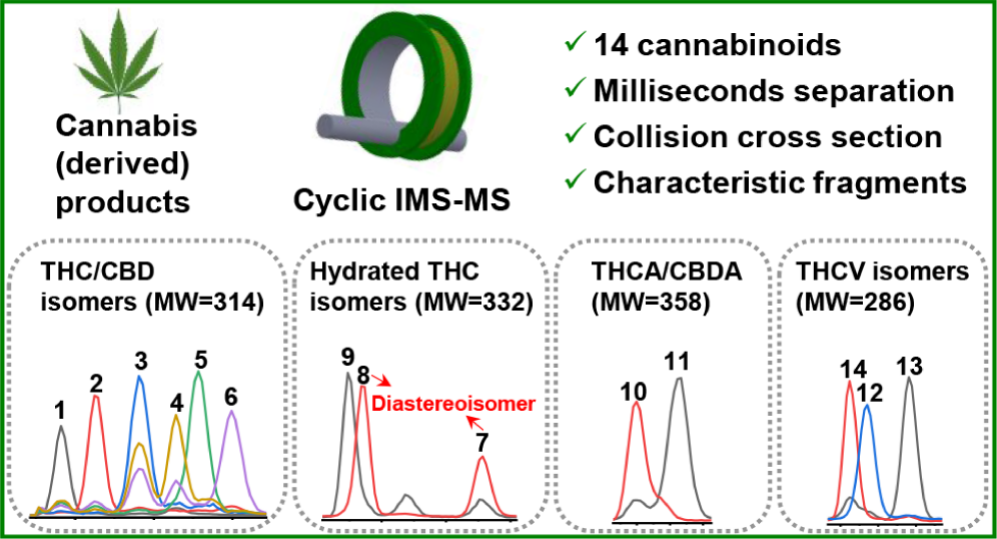

The diversity of cannabinoid isomers and complexity of Cannabis products pose significant challenges for analytical methodologies. In this study, we developed a method to analyze 14 different cannabinoid isomers in diverse samples within milliseconds by leveraging the unique adduct-forming behavior of silver ions in advanced cyclic ion mobility spectrometry–mass spectrometry. The developed method achieved the separation of isomers from four groups of cannabinoids: Δ3-tetrahydrocannabinol (THC) (**1**), Δ8-THC (**2**), Δ9-THC (**3**), cannabidiol (CBD) (**4**), Δ8-iso-THC (**5**), and Δ(4)8-iso-THC (**6**) (all MW = 314); 9α-hydroxyhexahydrocannabinol (**7**), 9β-hydroxyhexahydrocannabinol (**8**), and 8-hydroxy-iso-THC (**9**) (all MW = 332); tetrahydrocannabinolic acid (THCA) (**10**) and cannabidiolic acid (CBDA) (**11**) (both MW = 358); Δ8-tetrahydrocannabivarin (THCV) (**12**), Δ8-iso-THCV (**13**), and Δ9-THCV (**14**) (all MW = 286). Moreover, experimental and theoretical traveling wave collision cross section values in nitrogen (^TW^CCS_N2_) of cannabinoid-Ag(I) species were obtained for the first time with an average error between experimental and theoretical values of 2.6%. Furthermore, a workflow for the identification of cannabinoid isomers in Cannabis and Cannabis-derived samples was established based on three identification steps (*m*/*z* and isotope pattern of Ag(I) adducts, ^TW^CCS_N2_, and MS/MS fragments). Afterward, calibration curves of three major cannabinoids were established with a linear range of 1–250 ng·ml^–1^ for Δ8-THC (**2**) (*R*^2^ = 0.9999), 0.1–25 ng·ml^–1^ for Δ9-THC (**3**) (*R*^2^ = 0.9987), and 0.04–10 ng·ml^–1^ for CBD (**4**) (*R*^2^ = 0.9986) as well as very low limits of detection (0.008–0.2 ng·ml^–1^). Finally, relative quantification of Δ8-THC (**2**), Δ9-THC (**3**), and CBD (**4**) in eight complex acid-treated CBD mixtures was achieved without chromatographic separation. The results showed good correspondence (*R*^2^ = 0.999) with those obtained by gas chromatography-flame ionization detection/mass spectrometry.

Given the continuous growth of the Cannabis market and the variable composition of Cannabis products, comprehensive, sensitive, selective, and reliable analytical methods for the determination of cannabinoids, which are diverse and broad in occurrence, are needed to facilitate forensic oversight and understand health impact.^1^ Special focus is required on cannabinoid isomers, which—although similar in structure—can have highly varied pharmacology and legal status. The isomeric complexity of Cannabis-derived products extends far beyond the common isomers in Cannabis extracts, such as tetrahydrocannabinolic acid (THCA), cannabidiolic acid (CBDA), Δ9-tetrahydrocannabinol (THC), and cannabidiol (CBD). For instance, the common reaction of treating CBD with acid yields multiple classes of structural isomers, such as the THC isomers: Δ8-THC, Δ9-THC, Δ3-THC, Δ8-iso-THC, and Δ(4)8-iso-THC and hydrated THC isomers such as 8-hydroxy-iso-THC and 9α-hydroxyhexahydrocannabinol.^2−4^ The pharmacological effects of these byproducts have not been extensively studied, yet these compounds end up in popular Δ8-THC–containing products. These are bought by consumers for recreational or medicinal purposes, alarmingly resulting in increasing hospitalization cases.^5^

Comprehensive separation and distinction of these isomers are particularly challenging due to insufficient resolution of typical high pressure liquid chromatography (HPLC)-based methods and the incompatibility of acidic cannabinoids with gas chromatography (GC)-based methods.^3,4^ Alternatively, NMR can be used, but large amounts of samples are needed due to limited sensitivity, potentially resulting in undetected compounds with lower concentrations.^3,6^ Moreover, despite advancements in high-field NMR instruments, there are still significant challenges related to chemical shift resolution.^7^

Recently, efforts have been made to include ion mobility spectrometry (IMS) for the analysis of cannabinoid isomers in a more rapid, comprehensive, and sensitive way. IMS is a gas-phase separation technique for ions based on their mobility in an inert gas under an electric field. The mobility of ions is influenced not only by their size and charge but also by their three-dimensional conformation. This property is different from chromatographic retention times, *m*/*z* values, and MS fragmentation, and it is unaffected by various experimental conditions such as matrix, concentration, and specific equipment. Consequently, the collision cross section (CCS) can serve as a standardized molecular descriptor for both targeted and untargeted analysis.^8,9^ However, currently reported research on IMS-based separation for cannabinoid isomers suffers from limitations: (i) insufficient resolution toward cannabinoid isomers, especially THC isomers, (ii) limited types of cannabinoid isomers investigated, typically focusing mainly on the well-known THCA, CBDA, CBD, Δ8-THC, and Δ9-THC, (iii) no or indistinctive reporting of CCS values. Specifically, Tose et al.^8^ used traveling wave ion mobility spectrometry (TWIMS) and could resolve three out of five protonated cannabinoid isomers (assigned as Δ9-THC, Δ8-THC, cannabicyclol (CBL), cannabichromene (CBC), and CBD) but were unable to separate Δ9-THC and Δ8-THC. Similarly, by using TWIMS, Kiselak et al.^9^ could resolve protonated CBD and CBC but could not separate protonated Δ8-THC and Δ9-THC. Likewise, Zietek et al.^10^ were unable to distinguish protonated Δ9-THC and CBD by trapped IMS (TIMS), but Hädener et al.^11^ effectively resolved two isomeric pairs, Δ9-THC and CBD, as well as THCA and CBDA with a high-resolution drift-tube IMS (DTIMS, *R* > 150). Near-baseline separation was achieved, and experimental ^DT^CCS_N2_ values were obtained for protonated Δ9-THC and CBD as well as deprotonated THCA and CBDA. Clearly, it has been challenging to separate such isomeric cannabinoids by IMS. A major improvement of separation performance was obtained by leveraging the unique adduct-formation behavior of cannabinoids with silver ions to amplify structural differences and thus enhance isomer separation in the gas phase, first reported in 2018.^10^ In our previous work, we have subsequently reported that Ag(I) allows the distinction of cannabinoid isomers in both chromatography and mass spectrometry–based analysis due to different Ag(I) affinities.^4,12,13^ In 2021, we demonstrated, for the first time, that cannabinoid isomers with identical MS/MS product ion spectra of protonated precursor ions have completely different product ion spectra when selecting the silver adducts as precursor ions.^13^ Very recently, this effect was further studied for a wider range of cannabinoids, thus obtaining unique fragmentation for Δ8-THC (compound **2**), Δ9-THC (compound **3**), Δ8-iso-THC (compound **5**), Δ(4)8-iso-THC (compound **6**), cannabichromene (CBC), exo-THC, and CBD (compound **4**).^4,14^ In terms of IMS separation, Zietek et al.^10^ have demonstrated that the introduction of Ag(I) to TIMS allows the partial separation of Δ9-THC and CBD, despite the limited resolution of the instrument. Also, Ieritano et al.^14^ applied this strategy for differential mobility spectrometry (DMS) and could distinguish the isomers Δ8-THC, Δ9-THC, CBD, exo-THC, and CBC in oils. While this is a major step forward, those cannabinoids had previously been separated by reversed-phase HPLC.^15^ On the contrary, two cannabinoids that are typically found in synthetic Δ8-THC products and are known to interfere with HPLC analysis of Δ8-THC products remain to be addressed,^6,16^ namely, the Δ8-THC iso-forms (Δ8-iso-THC (compound **5**) and Δ(4)8-iso-THC (compound **6**)). Moreover, to the best of our knowledge, no reports of IMS separation of diastereomeric hydrated cannabinoids are available, while these compounds have recently been demonstrated to occur in Δ8-THC products for consumption.^17^ Finally, currently, no CCS values of any cannabinoid-Ag(I) adducts have been reported. The aim of the current work has thus been to address these challenges and to arrive at a broadly applicable set of operations that allows separation, identification, and quantification of many classes of isomeric cannabinoids, even those that are inseparable on chromatographic equipment, in a matter of milliseconds. Logically, this would require more advanced operations in the ion mobility space, mass spectrometry space, and interface between these than used in previous works, which is why cyclic IMS (cIMS) coupled to a quadrupole time-of-flight (qTOF) MS was selected for this purpose.

cIMS, first reported in 2019, greatly improves the resolving power of conventional TWIMS by the multipass running of ions in a 98-cm cyclic ion mobility tube to increase the separation path length.^18^ It has shown excellent separation performance for isomeric saponin,^19^ oligosaccharides,^20^ and flavonoids,^21^ but it has not yet been tried for isomeric cannabinoids. This novel state-of-the-art equipment has superior resolving power (a resolution (R) of ∼78 in 1 pass up to ∼750 in 100 passes, CCS/ΔCCS) compared to linear IMS-MS (*R* < 40 for a standard linear TWIMS cell, CCS/ΔCCS). Moreover, it incorporates high-resolution mass spectrometry (TOF) for accurate mass-to-charge ratio (*m*/*z*) measurements after IMS separation. Finally, also MS/MS fragments can be further separated with IM, providing a powerful technique for studies on fragmentation products and their 3D conformation.^18−21^ The latter is especially interesting to further study the interaction between analytes and less common adduct ions such as Ag(I)-cannabinoid interactions. These interactions are known to be quite diverse for different cannabinoids^4,12−14^ and could help in explaining the differences in 3D shape (and thus in their CCS) of these silver adduct ions and why they can be separated. This also means that through comparison of experimental and theoretical CCS values, potentially new insights can be gained into the interactions between silver ions and cannabinoids. Such studies have, to the best of our knowledge, not been performed in the context of cannabinoid analysis. Addressing these challenges is important to further allow the development of an analytical workflow, including multiple molecular descriptors, for the unambiguous analysis of a wide range of cannabinoids (Figure 1, 14 cannabinoids, four groups of isomers) in Cannabis and Cannabis-derived samples. Establishing such a workflow as well as benchmarking it against gold standard chromatography-based methods has been the objective of this paper.

**Figure 1:**
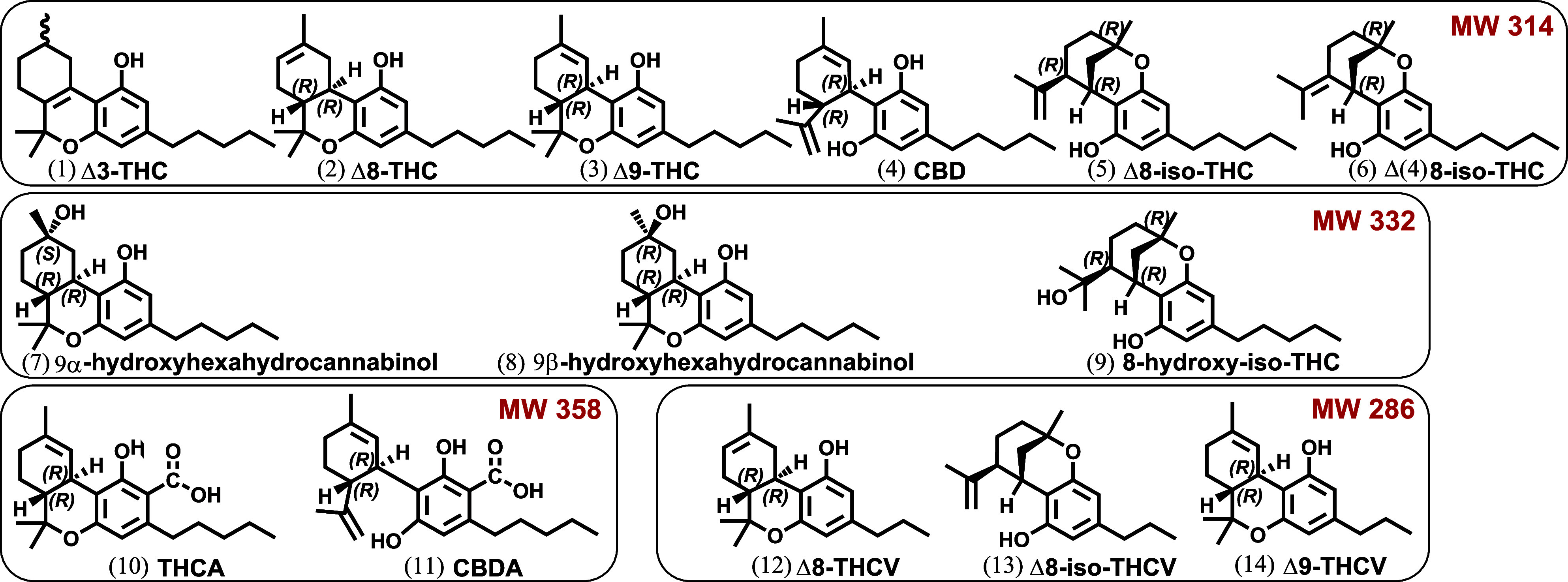
Structures of investigated cannabinoids (four groups of isomers) in this study.

## Experimental Section

### Chemicals and Reagents

Silver nitrate (AgNO_3_, analytical grade) was purchased from Fisher Scientific (Loughborough, Leicestershire). Methanol (MeOH, HPLC-grade) was obtained from VWR Chemicals (Gliwice, Poland). Methyl *tert*-butyl ether (MTBE) was purchased from Biosolve BV (Valkenswaard, the Netherlands). Major Mix IMS/ToF calibration kit was purchased from Waters (Wilmslow, UK). Acid-treated CBD mixtures were prepared in our previous study (Table S1, data from our previous study)^4^ and abbreviated as R#1–R#8. Cannabis materials (C#1–C#3) were purchased locally, and Δ8-THC gummies (G#1–G#2) were purchased online (Table S2). Δ(4)8-iso-THC (**6**) was kindly provided by Danielle Passarella (Dipartimento di Chimica, Università degli Studi di Milano, Milano, Italy).^3^ Crystalline CBD (**4**) (99%) was purchased from CBDolie.nl. CBDA isolate (90%–95%) was obtained from GVB Biopharma (Tygh Valley, USA). Δ8-THC (**2**), Δ9-THC (**3**), CBD (**4**), Δ8-iso-THC (**5**), 9α-hydroxyhexahydrocannabinol (**7**), 9β-hydroxyhexahydrocannabinol (**8**), and 8-hydroxy-iso-THC (**9**) standards (purity >98%) were isolated and identified in our previous study.^4^ THCA (**10**) was purified from Cannabis flowers. Δ3-THC (**1**) was purified from Δ10-THC vape oil obtained online. Δ9-THCV (**14**) and the mixture containing Δ8-THCV (**12**) and Δ8-iso-THCV (**13**) (with a mole ratio of 1:2) were isolated from 4% THCV oil purchased online. The newly isolated cannabinoids in the current study Δ3-THC (**1**), THCA (**10**), Δ9-THCV (**13**) were identified by 1D and 2D NMR (Bruker 700 MHz Avance, Bruker GmBH, Rheinstetten, Germany) and analyzed by reversed-phase UHPLC-UV/MS. The isolated mixture containing Δ8-THCV (**13**) and Δ8-iso-THCV (**14**) (with a mole ratio of 1:2) was identified by ^1^H NMR (Bruker 700 MHz Avance, Bruker GmBH, Rheinstetten, Germany), reversed-phase UHPLC-UV/MS, GC-FID/MS, and silica-Ag(I) HPLC-DAD^4^ (Figures S1–S4). According to NMR and peak integrations at UV 215 nm, the purity of compounds **1**, **14**, and the mixture of **12** and **13** (mole ratio 1:2) was >90%; the purity of compound **10** was >75%.

### Solutions and Samples

The stock solution of each standard cannabinoid and acid-treated CBD mixtures (R#1–R#8) was prepared in MeOH at 100 μg·mL^–1^. 6.0 mg, 6.4 mg, and 7.7 mg Cannabis (C#1, C#2, and C#3) were accurately weighed (Mettler Instrumente AG, CH-8606 Greifensee-Zurich) in 1.5 mL Eppendorf safe-lock tubes (Eppendorf Nederland B.V., Nijmegen, Netherlands). 600 μL, 640 μL, and 770 μL of MeOH were added individually with a micropipette (Eppendorf research plus, 100–1000 μL, Nijmegen, Netherlands). After a 10-min sonication extraction (Bandelin Sonorex, Rangendingen, Germany), the solutions were filtered over 0.2 μm PTFE membrane syringe filters (Pall Corporation, Port Washington, NY, USA) and diluted with MeOH by 100 times to 100 μg·mL^–1^ (= Cannabis stock solution). 10.0 mg of Δ8-THC gummies (G#1 and G#2) was extracted by 1.00 mL MTBE/H_2_O (v/v = 1:1) in 1.5 mL Eppendorf safe-lock tubes with handshaking for 15 min. After waiting 10 min for phase separation, 300 μL of the MTBE layer was filtered over 0.2 μm PTFE membrane syringe filters. After that, 100 μL of the filtered solution was blow-dried and reconstituted in 2.00 mL of MeOH to 100 μg·mL^–1^ (gummy stock solution). The MeOH or 10^–4^ M AgNO_3_ in MeOH (in a brown bottle) was used to further dilute the stock solutions ten times for cIMS analysis, unless otherwise stated. For CBD (**4**), CBDA (**11**), Δ8-THCV (**13**), and Δ8-iso-THCV (**14**), the dilution was 100 × by a 10^–4^ M AgNO_3_ MeOH solution. For the analysis of standard mixtures, diluted cannabinoid stock solutions were mixed in equal volumes.

### cIMS-qTOF-MS Analysis

A Select Series Cyclic Ion Mobility Mass Spectrometer (cIMS, Waters Corporation, Wilmslow, U.K.) was used in this study. Direct infusion analysis at a flow rate of 15 μL·min^–1^ was used, unless otherwise stated. For ionization, the capillary voltage was 2.5 kV when there was no Ag(I) and 1.2 kV when Ag(I) ions were present. The cone voltage was 40 V with the source temperature at 100 °C, the nitrogen desolvation gas flow at 800 L·h^–1^, and the desolvation temperature at 300 °C. TOF (V) mode was used for general MS analysis without mobility separation. Mobility mode was aimed at mobility separation and analysis. Major settings of the mobility mode (Cyclic Control) were 5 pushes per bin, traveling wave (TW) velocity 375 m·s^–1^, TW static height and start height 15 V, and inject time 10 ms. Multiple-pass separation was achieved by using the manual function with a slider. The qualitative analysis of standards and samples was conducted by MS full scan in TOF mode, isolating targeted *m*/*z* for mobility separation, trap fragmentation (by adjusting trap energy) before mobility separation, and transfer fragmentation (by adjusting the transfer energy) after mobility separation. For experiments without fragmentation, 6 V trap energy and 4 V transfer energy were used. Nitrogen was used as collision and cIMS gas. For quantitative analysis, loop injection (5 μL) instead of direct infusion was used. MeOH was used to thoroughly flush the system between different samples. Acquisition and processing were performed using MassLynx (version 4.2), DriftScope (version 3.0), and Microsoft Excel. The instrument was mass calibrated with a Major Mix IMS/ToF Calibration Kit (Waters Corp, Wilmslow UK) in both positive and negative ion electrospray mode at 60 000 resolution (FWHM) over an *m*/*z* range of 50–1000. 50 pg·μL^–1^ of leucine enkephalin in water/acetonitrile (50:50, v/v) was infused at 1 μL·min^–1^ to be used as lockmass calibrant ([M + H]^+^*m*/*z* 556.2766).

### Multipass CCS Calibration and Experimental Measurement

Multipass CCS calibration and experimental measurements were performed according to instructions from the manufacturer.^22−26^ Major Mix calibration standards were measured under 1-pass and 2-pass separation settings. Only six out of twenty-nine calibrants were selected in this study (Table S3), considering *m*/*z* values below and above the *m*/*z* of interest (cannabinoids range *m*/*z* 315–421). These compounds were peak detected and the arrival times (*t*_a_, eq 1) of the calibrant ions were determined for both 1-pass and 2-pass separation data sets. The drift time (*t*_d_) for a single pass of each calibrant ion (Eq 2) and the dead time (*t*_0_, eq 3) of the cIM-ToF system were then calculated using the arrival times (Eq 1). The CCS calibration curve was then constructed by using the 1-pass drift time (*t*_d_) values and the power function y = ax^b^. To measure CCS values of unknown analytes in multipass separation settings, a corrected single-pass transit time (^c^*t*_t_) should be calculated with eq 4, in which the multipass drift time (^mp^*t*_d_) was obtained from multiple-mass arrival times (*t*_a_) minus dead time (*t*_0_). After that, the plotted CCS calibration curve (Figure S5) was used to obtain CCS values of unknowns. Cannabinoid standards prepared at different concentrations (10.0 μg·mL^–1^ unless otherwise stated) in MeOH or 10^–4^ M AgNO_3_ in MeOH were injected in triplicate, thus obtaining the ^TW^CCS_N2_ from the average of *n* = 3, unless otherwise specified.
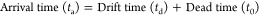
1
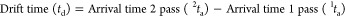
2
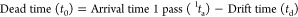
3
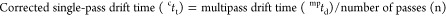
4

### Chromatography

Cannabis extracts (C#1–C#3) were analyzed by the reversed phase UHPLC-UV/MS method developed in our previous study.^4^ Δ8-THC gummy extracts (G#1–G#2) were analyzed by a slightly modified GC-FID/MS method developed in the same study.^4^ Specifically, a DB-5MS UI capillary column (Agilent J and W GC column, USA) instead of HP-5MS capillary column was used with a prolonged temperature program. The temperature program started with an initial column temperature of 200 °C, followed by a gradual increase at a rate of 1 °C·min^–1^ until reaching 223 °C. Subsequently, the column temperature was further elevated at a rate of 5 °C·min^–1^ to 250 °C and maintained at this level for 15 min, resulting in a total analysis time of 43 min. A 1 μL sample was injected with a 1:10 split ratio. The injection temperature was 200 °C. Helium was used as the carrier gas with a linear velocity of 26 cm/s, and the flow was constant during the entire analysis. The mass spectrometer was operated in 70 eV electron ionization (EI) mode, scanning from *m*/*z* 35 to 500 at 4 spectra/s. Measurements were delayed by 3.0 min following an injection to safeguard the filament of the mass spectrometer.

### Quantification of Δ8-THC, Δ9-THC, and CBD

1.00 μg·mL^–1^ of Δ8-THC (**2**), Δ9-THC (**3**), and CBD (**4**) in 10^–4^ M AgNO_3_ MeOH were prepared as stock solutions. 250 μL of 1.00 μg·mL^–1^ Δ8-THC (**2**), 25.0 μL of 1.00 μg·mL^–1^ Δ9-THC (**3**), and 10.0 μL of 1.00 μg·mL^–1^ CBD (**4**) were mixed and diluted with 10^–4^ M AgNO_3_ MeOH to 1.00 mL to obtain a mixed standard solution of Δ8-THC (**2**) (250 ng·mL^–1^), Δ9-THC (**3**) (25.0 ng·mL^–1^), and CBD (**4**) (10.0 ng·mL^–1^). The obtained mixed standard solution was then diluted to obtain a series of working solutions with the concentration range 1.00–250 ng·mL^–1^ for Δ8-THC (**2**), 0.100–25.0 ng·mL^–1^ for Δ9-THC (**3**), and 0.0400–10.0 ng·mL^–1^ for CBD (**4**). 5.00 μL portion of each working solution was injected by a loop injector to perform a multipass ion mobility separation and postmobility fragmentation (transfer energy 30 V) of the precursor ion at *m*/*z* 421. Calibration curves for Δ8-THC, Δ9-THC, and CBD were made by plotting areas of the extracted mobiligram of the characteristic fragment at *m*/*z* 245 for Δ8-THC (**2**), *m*/*z* 313 for Δ9-THC (**3**), and *m*/*z* 353 for CBD (**3**) against the used concentrations. Limit of detection (LOD) of Δ8-THC (**2**), Δ9-THC (**3**), and CBD (**4**) was calculated as follows: LOD = 3 × SD of the lowest concentration of the calibration curve/slope of the calibration curve.

### Prediction of and Calculation of the Theoretical CCS Values (tCCS)

Theoretical CCS of protonated and sodiated species were predicted using AllCCS (http://allccs.zhulab.cn/).^27,28^ In brief, using a training set of experimentally measured CCS, the software employs a machine learning algorithm that is able to predict CCS values for novel structures. To calculate the predicted CCS for [M + H]^+^ and [M + Na]^+^ species, the SMILES string of each cannabinoid was imported to the web interface of AllCCS Predictor. CCS values for Ag^+^ adducts could not be predicted by AllCCS because no Ag^+^ ions were used to build the training set. Therefore, density functional theory (DFT)–based methods were used to obtain theoretical CCS values of cannabinoid-Ag(I) species by a two-step procedure.^29^ Specifically, quantum chemistry–based optimization of cannabinoid-Ag(I) structures was used as an input in Collidoscope^30^ to obtain tCCS values, and a Boltzmann-weighted distribution of possible structures was considered to produce the final averaged tCCS values. The optimization was conducted through B97XD/def2TZVP calculations, utilizing the corresponding parameters in Gaussian 16. All structures were fully optimized, and vibrational frequency calculations were performed to confirm that these were minima and to obtain the free energy.

## Results and Discussion

### cIMS Multiple-Pass Separation of Cannabinoid Isomers

**Figure 2:**
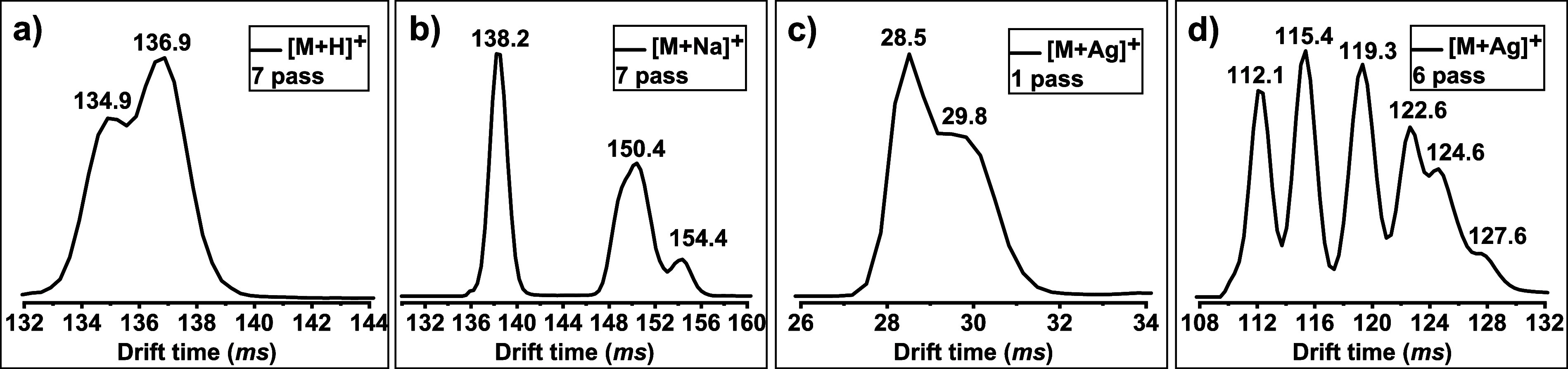
Mobiligrams of the mixture of six isomers (compounds **1**–**6**) (a) as protonated species (extracting [M + H]^+^ signal at *m*/*z* 315; drift time at 134.9 ms: Δ8-iso-THC and Δ(4)8-iso-THC; drift time at 136.9 ms: CBD, Δ8-THC, Δ9-THC, and Δ3-THC) after 7-pass separation; (b) as sodiated species (extracting [M + Na]^+^ signal at *m*/*z* 337; drift time = 138.2 ms: CBD; drift time = 150.4 ms: Δ9-THC, Δ8-iso-THC, Δ(4)8-iso-THC, and Δ8-THC, drift time = 154.4 ms: Δ3-THC) after 7-pass separation; (c) as Ag(I) adducts (extracting [M+Ag]^+^ signal = *m*/*z* 421; drift time = 28.5 ms: CBD and Δ9-THC; drift time = 29.8 ms: Δ(4)8-iso-THC, Δ8-iso-THC, Δ8-THC, and Δ3-THC) after 1-pass separation; and (d) as Ag(I) adducts (extracting [M+Ag]^+^signal = *m*/*z* 421; drift time = 112.1 ms: CBD; drift time = 115.4 ms: Δ9-THC; drift time = 119.3 ms: Δ(4)8-iso-THC; drift time = 122.6 ms: Δ8-iso-THC; drift time = 124.6 ms: Δ8-THC; drift time = 127.6 ms: Δ3-THC) after 6-pass separation.

Despite the isomeric separation power of IMS, only limited research has been performed toward the analysis of cannabinoid isomers, and the maximum number of resolved isomeric cannabinoids reported so far is five.^14^ In the current study, the comprehensive investigation of 14 acidic and neutral cannabinoids (Figure 1), forming four groups each with a specific MW, was carried out with advanced ccIMS-MS. First, the separation of six THC isomers (a mixture of compounds **1**–**6**) was investigated. The six protonated species (Figure S6a–c) showed no or little separation with 1 pass up to 7 passes (Figure 2a). Even increasing the number of passes to 11 resulted in only a shoulder peak. As the number of passes was further increased, peak broadening became increasingly prominent as opposed to yielding enhancements in separation. Alternatively, sodiated species (Figure S6d–e), which were more intense than the protonated signals, showed two peaks for the mixtures containing six isomers in the mobiligram after 1-pass separation. At best, three peaks could be observed after a 7-pass separation of sodiated species (Figure 2b), which is still not enough for the distinction of these six isomers. As revealed in our previous research,^31−33^ Ag(I) has different affinities toward compounds with different numbers or positions of olefinic double bonds, and recent work^4,10,12−14^ also proved cannabinoid isomers have different interactions with Ag(I). Therefore, we investigated whether Ag(I) complexation in combination with advanced cIMS would further benefit the analysis of more complex mixtures and diverse cannabinoids. While a 1-pass separation of Ag(I) adducts showed limited separation (Figure 2c), the 6-pass separation exhibited obvious improvement compared to its Na^+^-counterpart, with six identified peaks in the mobiligram for the six THC isomers (Figure 2d). Further increasing the number of passes would result in wrap-around effects (the fastest ions catching up with the slowest ones). The Supporting Information provides a full comparison of the various charged species (Figure S6a–c for protonated species, S6d–f for Na^+^ adducts, and S6f–h for Ag^+^ adducts).

### Postmobility Fragmentation for Improved Identification

#### THC/CBD Isomers (Compounds **1**–**6**, MW 314)

**Figure 3:**
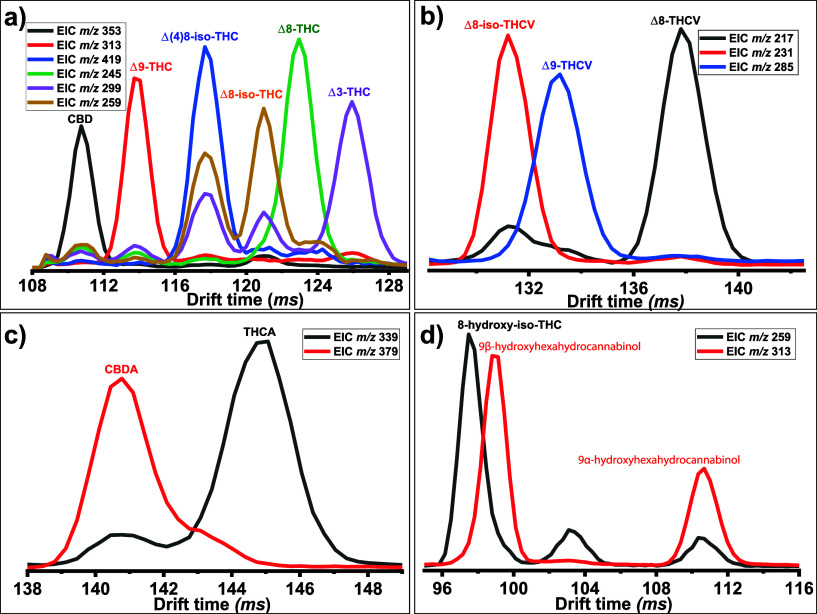
Extracted mobiligram of the characteristic fragment of (a) Δ8-THC, Δ9-THC (÷5), Δ3-THC, CBD, Δ8-iso-THC (×2), and Δ(4)8-iso-THC (×4) after 6-pass separation; (b) Δ9-THCV (÷5), Δ8-THCV and Δ8-iso-THCV after 7-pass separation; (c) THCA and CBDA after 7-pass separation; (d) 9α-hydroxyhexahydrocannabinol (÷10), 9β-hydroxyhexahydrocannabinol (÷10), and 8-hydroxy-iso-THC after 5-pass separation in the presence of Ag(I).

Though multiple-pass separation of Ag(I) adducts could already resolve all six THC isomers, a more selective strategy was explored for the unambiguous identification of each isomer. Based on previous research, different Ag(I) adducts are known to produce different ESI-MS fragmentation patterns, which thus can facilitate cannabinoid isomer distinctions.^4,12−14^ Therefore, postmobility fragmentation was performed. Since postmobility fragmentation happens after mobility separation, fragment and precursor drift times are aligned, which can facilitate the assignment of fragments to specific precursors. Indeed, the six isomers exhibited distinct fragmentation patterns with major characteristic fragments for Δ3-THC (**1**) at *m*/*z* 299, Δ8-THC (**2**) at *m*/*z* 245, Δ9-THC (**3**) at *m*/*z* 313, CBD (**4**) at *m*/*z* 353, Δ8-iso-THC (**5**) at *m*/*z* 259, and Δ(4)8-iso-THC (**6**) at *m*/*z* 419 (Figure S7a). By extracting the major characteristic fragment signal for each isomer after the 6-pass separation, six distinct traces can be observed (Figure 3a), even for cannabinoids, which have been impossible to separate to date by RP-UHPLC. Therefore, distinct fragments can provide extra evidence apart from drift time for cannabinoid identification.

#### THCV Isomers (Compounds **12**–**14**, MW 286)

Compared with Δ9-THC (**3**), Δ8-THCV (**12**), Δ8-iso-THCV (**13**), and Δ9-THCV (**14**) contain a propyl rather than a pentyl side chain. Δ9-THCV exists in Cannabis and shows pharmacological effects similar to those of Δ9-THC.^34^ Δ8-THCV (**13**) and Δ8-iso-THCV (**14**), while structurally similar to Δ8-THC (**2**) and Δ8-iso-THC (**5**), likely originate from organic synthesis,^35^ and there is only limited information about the pharmacological effects of these two cannabinoids. Despite the coelution in RP-UHPLC (Figure S2), with the cIMS method, the three isomeric THCV were well distinguished. Δ9-THCV (**14**) had a characteristic fragment at *m*/*z* 285, which is 28 Da less than the characteristic fragment of Δ9-THC (**3**) at *m*/*z* 313 (Figure S7b). Similarly, Δ8-iso-THCV (**13**) was characterized by fragments at *m*/*z* 231, 28 Da less than the corresponding fragments of Δ8-iso-THC (**5**) (*m*/*z* 259). This also worked for Δ8-THCV (**13**) and Δ8-THC (**2**), showing a difference of 28 Da between the characteristic fragments (*m*/*z* 217 vs *m*/*z* 245). Also, due to the shorter side chain, small conformational differences of Ag(I) adducts resulted in a slightly lower resolution of THCV isomers compared with their THC counterparts (Figure 3b).

#### THCA/CBDA (Compounds **10**–**11**, MW 358)

The Ag(I)-enhanced-multiple-pass separation combined with postmobility fragmentation works not only for the neutral cannabinoid isomers Δ3-THC (**1**), Δ8-THC (**2**), Δ9-THC (**3**), CBD (**4**), Δ8-iso-THC (**5**), Δ(4)8-iso-THC (**6**), Δ9-THCV (**14**), Δ8-THCV (**12**), and Δ8-iso-THCV (**13**) but also for the distinction of acidic cannabinoids THCA (**10**) (characteristic fragment at *m*/*z* 339) and CBDA (**11**) (characteristic fragment at *m*/*z* 379) (Figures 3c and S7c). This is highly valuable for the direct analysis of Cannabis extracts since they mainly contain such acidic cannabinoids.^36^

#### Hydrated THC Isomers (Compounds **7**–**9**, MW 332)

It was recently demonstrated that in addition to THC isomers, hydrated THC isomers occur in commercial Δ8-THC products.^17^ Three hydrated THC isomers were isolated and identified as 9α-hydroxyhexahydrocannabinol (**7**), 9β-hydroxyhexahydrocannabinol (**8**), and 8-hydroxy-iso-THC (**9**) in our previous study.^4^ cIMS was then also applied to their analysis. Unfortunately, these isomers could not be resolved by mobility separation alone (Figure S8), and thus, relying on drift time for compound assignments is insufficient. Through postmobility fragmentation and extracting characteristic fragments, three hydrated THC species, of which two are stereoisomers, could also be distinguished (Figure 3d). On the contrary, without cIMS separation, it would be impossible to separate these compounds based on their traces alone as several fragments also occur as minor fragments for other isomers (e.g., characteristic fragment of 8-hydroxy-iso-THC (**9**) at *m*/*z* 259 also occurs in 9α-hydroxyhexahydrocannabinol (**7**), and both 9α-hydroxyhexahydrocannabinol (**7**) and 9β-hydroxyhexahydrocannabinol (**8**) share the characteristic fragment at *m*/*z* 313) (Figure S7d). Therefore, these two techniques are truly complementary and together exhibit excellent distinction power for a comprehensive range of cannabinoid isomers.

Additionally, in both ESI-qTOF-MS and GC-MS, the three hydrated THC isomers tend to lose H_2_O and thus form a product with the same molecular weight as compounds **1**–**6** (Figure S9 and S10). In order to investigate whether the existence of hydrated THC isomers would interfere with the analysis of compounds **1**–**6**, the three hydrated THC isomers were analyzed individually by cIMS with or without Ag(I) being present. When selecting the protonated species at *m*/*z* 333 in the absence of Ag(I), apart from the signals at *m*/*z* 333.2420, there were peaks at *m*/*z* 315.2311 (dehydrated forms of signals at *m*/*z* 333.2420) detected for all three hydrated THC isomers (Figure S11a), which matches the results obtained by RP-UHPLC-ESI-Orbitrap-MS (Figure S9). However, with Ag(I), when the silver adduct was selected at *m*/*z* 439, no dehydrated silver adducts were detected under the same conditions (Figure S11b). Therefore, the formation of Ag(I) adducts is hypothesized to stabilize the hydrated THC isomers and prevent H_2_O loss during ionization to form products that might interfere with the analysis of THC isomers. That is very meaningful for preventing false-positive THC results when Cannabis products, e.g., Δ8-THC products, are analyzed without chromatographic separation.

### Experimental and Theoretical CCS Determination of Ag(I) Adducts for Unambiguous Identification

Apart from providing the extra dimension of separation to increase peak capacity, one of the most attractive parts of IMS is the ability to obtain CCS values, a structure-dependent parameter, for compound identification.^37^ Among the prominent ion mobility techniques, the Field Asymmetric IMS (FAIMS) also known as DMS cannot provide CCS information because of their asymmetric waveform and ion structural alterations induced by the oscillation between low and high electric field strengths.^38^ In contrast, DTIMS, TWIMS, and TIMS methodologies can yield CCS values through direct measurement or calculation derived from calibration curves between drift time and CCS values of calibrants.^39^ Furthermore, in contrast to the drift time observed in DTIMS, TWIMS, and TIMS, as well as the compensation voltage utilized in DMS, CCS as a molecular identifier remains unaffected by experimental conditions and facilitates cross-platform comparisons as well as untargeted analysis.^40^ Therefore, in the current study, CCS values of 14 cannabinoids were experimentally derived (Table S3 and Figure S5), for their proton, sodium, and Ag(I) adducts. To the best of our knowledge, this is the first time that experimental CCS (eCCS) values of Ag(I) adducts of cannabinoid isomers are reported. They showed a relatively higher variation than CCS values of proton and sodium adducts (Table 1), which facilitates the distinction between them. Comparison of the eCCS values of protonated Δ9-THC (**3**) and CBD (**4**) in this study with those obtained in other studies (see Table S4 for other different IMS) showed relative deviations (RD) of only −0.2% to −2.1%, demonstrating the accuracy of eCCS values measured by cIMS.

**Table 1:** Experimentally Derived Traveling Wave Collision Cross Section Values in Nitrogen (^TW^CCS_N2_, Å^2^) of THC Isomers as Protonated Species, Sodiated Species, and Ag(I) Adducts Were Measured by cIMS Under 7-Pass Separation Settingsi

		eCCS*			tCCS	calculation error
compounds	**MW**	**[M + H]**^**+**^	**[M + Na]**^**+**^	**[M + Ag]**^**+**^	**[M + Ag]**^**+**^	**[M + Ag]^+^**
Δ3-THC (**1**)	314.2	188.4 ± 0.01	197.9 ± 0.03	192.9 ± 0.03	186.1	–3.5%
Δ8-THC (**2**)	314.2	187.8 ± 0.05	196.4 ± 0.04	190.9 ± 0.02	194.7	2.0%
Δ9-THC (**3**)	314.2	187.8 ± 0.03	194.8 ± 0.01	183.9 ± 0.01	195.3	6.2%
CBD (**4**)	314.2	187.8 ± 0.01	188.5 ± 0.02	181.8 ± .01	180.3	–0.8%
Δ8-iso-THC (**5**)	314.2	186.5 ± 0.03	195.7 ± 0.01	189.5 ± 0.01	186.4	–1.7%
Δ(4)8-iso-THC (**6**)	314.2	186.6 ± 0.04	195.8 ± 0.01	187.1 ± 0.01	179.2	–4.2%
9α-hydroxyhexahydrocannabinol (**7**)	332.2	191.2 ± 0.02	201.7 ± 0.02	195.6 ± 0.01	195.1	–0.3%
9β-hydroxyhexahydrocannabinol (**8**)	332.2	191.6 ± 0.2	193.0 ± .01	185.9 ± 0.01	193.9	4.3%
8-hydroxy-iso-THC (**9**)	332.2	187.9 ± 0.01	200.8 ± .02	184.6 ± 0.02	190.2	3.0%
THCA (**10**)	358.2	194.0 ± 0.2	212.2 ± 0.01	190.9 ± 0.01	202.6	6.1%
CBDA (**11**)	358.2	193.6 ± 0.03	206.7 ± 0.01	188.6 ± 0.01	192.7	2.2%
Δ8-THCV (**12**)	286.2	174.9 ± 0.01	188.0 ± 0.02	187.9 ± 0.01	186.4	–0.8%
Δ8-iso-THCV (**13**)	286.2	173.5 ± 0.01	186.4 ± 0.02	183.8 ± 0.01	181.5	–1.3%
Δ9-THCV (**14**)	286.2	174.7 ± 0.01	185.9 ± 0.01	184.9 ± 0.01	183.6	–0.7%

i ^*TW^CCS_N2_ ± SD (Å^2^), *n* = 3.

We also compared the eCCS values of protonated and sodiated species with predicted CCS (pCCS) values obtained by the machine learning–based online tool AllCCS. (Table S4). For protonated species, the prediction errors  were all within ±2.1%, showing good prediction accuracy. Twelve out of 13 sodiated species of cannabinoids had a prediction error of ≤5%. However, for sodiated 9β-hydroxyhexahydrocannabinol (**8**), the pCCS was overestimated by 6.5%. While the AllCCS platform shows fairly good CCS prediction power for most protonated and sodiated cannabinoids, this platform does not include argentated species, which are more distinctive for cannabinoids based on our derived eCCS values. On the contrary, Duez et al.^29^ obtained theoretical CCS (tCCS) values of Ag(I)-complexed alkylamines based on density functional theory (DFT) computational methods. Thus, we applied this methodology to cannabinoids for the first time. The tCCS values agree quite nicely with the experimental values, with an overall error of 2.6% for these 14 cannobinoids (Table 1). However, there is quite some variation within this set, with errors ranging from −4% for Δ(4)8-iso-THC (**6**) to +6% (for THC (**3**) and THCA (**10**)). For seven out of the 14 cannabinoids an absolute calculation error within 2% was obtained,^41^ and the average calculation error of 2.6% is smaller than observed by other DFT-based studies, e.g., ISiCLE, with an average error of 3.2%.^42^ However, this study also points to clear limits of this approach, especially for isomers with small differences in structures. For example, the relative difference of eCCS between Δ8-THC (**2**) and Δ8-iso-THC (**5**) is 0.7%, which means that with a calculation error of 2.6%, it is not possible to distinguish between these isomers. The DFT calculation faces an intrinsic limitation due to the subjective empirical selection of possible conformations. Ideally, an unbiased set of conformations as obtained from, e.g., molecular dynamics should be used, even though it can be computationally very expensive.^39^ Then, for each of these, the CCS would be calculated and weighted with their Boltzmann factor. In this way, subjective biases can be mitigated, and thus, the accuracy of the results might be enhanced. On the contrary, a more stringent treatment of buffer gas (N_2_) could be incorporated in future version of the Collidoscope prediction software to mitigate errors associated with trajectory integration.^30^ In the future, the ability to calculate and predict CCS values of cannabinoid-Ag(I) might also benefit from the development of libraries to facilitate untargeted cannabinoid investigations if the prediction error can be strongly reduced. For now, as with gold standard separation on HPLC or GC, reference standards remain indispensable for unambiguous identification.

### Sequential Premobility and Postmobility Fragmentation for Further Investigation of Stereoisomers of 9α-Hydroxyhexahydrocannabinol and 9β-Hydroxyhexahydrocannabinol

It has been observed in our previous study that the distinction of cannabinoid isomers mainly relied on Ag(I)-alkene complexation.^4,12,13^ Interestingly, the three hydrated THC isomers (compound **7**–**9**), with no olefinic double bonds, still exhibited different eCCS and MS fragments in the presence of Ag(I). This showed that Ag(I) can also contribute to the distinction of cannabinoids without olefinic double bonds, which could not be achieved with protonated species. Our early research also revealed that polar groups like hydroxyls weakly interact with Ag(I).^31^ We therefore investigated whether the interaction of Ag(I) and hydroxyls contributed to the distinction and how they interacted.

First, as mentioned above, the presence of Ag(I) uniquely prevented H_2_O loss of the three hydrated THC isomers, thus providing evidence of Ag(I)-hydroxyl interactions. When applying various transfer fragmentation energies to fragment these Ag(I) adducts (Figure S12), they exhibited different stabilities in the order of [8-hydroxy-iso-THC+Ag]^+^ > [9α-hydroxyhexahydrocannabinol+Ag]^+^ > [9β-hydroxyhexahydrocannabinol+Ag]^+^, showing different interactions between Ag(I) and hydroxyls with different spatial orientation. In order to study the dehydrated species, we selected Ag(I) adducts at *m*/*z* 439 and performed premobility fragmentation to force the H_2_O loss. Except for signals of [M+Ag]^+^ at *m*/*z* 439/441, there were now also signals of [M+Ag–H_2_O]^+^ at *m*/*z* 421/423 for all three hydrated THC isomers (Figure S11c). Afterward, multiple-pass separation, CCS measurements, and postmobility fragmentation were conducted. As shown in Figure S13 and summarized in Table S5, [9α-hydroxyhexahydrocannabinol+Ag]^+^ had a CCS value of 195.6. After H_2_O loss, it formed two dehydrated Ag(I) adducts with CCS values of 182.0 and 190.6. [9β-hydroxyhexahydrocannabinol+Ag]^+^ showed a much smaller CCS value of 185.9 and formed three dehydrated Ag(I) adducts during premobility fragmentation, with CCS values of 181.9, 183.9, and 190.6. Despite the large difference in eCCS (195.6 vs 185.9) of 9α-hydroxyhexahydrocannabinol and 9β-hydroxyhexahydrocannabinol Ag(I) species, after H_2_O (hydroxyl) loss, the eCCS values (and thus the corresponding 3D structure) became almost identical, providing more evidence of the interaction between Ag(I) and hydroxyls. [8-hydroxy-iso-THC+Ag]^+^ with the eCCS of 184.6 formed only one dehydrated product (eCCS = 179.7). By comparing eCCS and postmobility fragmentation of dehydrated species (Table S5 and Figure S13) with reference standards, it was found that one of the dehydrated species formed from 9α-hydroxyhexahydrocannabinol and 9β-hydroxyhexahydrocannabinol might be Δ8-THC, while others remained unassigned. In short, it is likely that the interaction of Ag(I) and hydroxyls can also contribute to the distinction of cannabinoids without olefinic double bonds by different 3D conformers, stability, and dehydration. Similarly, Ollivier et al.^43^ found that the formation of lithium adducts would affect the mobility and dehydration of oligosaccharides and used this for the distinction of α-linked and β-linked glucans. Besides, in this study, we observed a large difference in eCCS between [9α-hydroxyhexahydrocannabinol+Na]^+^ and [9β-hydroxyhexahydrocannabinol+Na]^+^ (201.7 vs 193.0). Likewise, stereoisomers epicatechin and catechin, with different spatial orientations of only one hydroxyl moiety, could be separated by cIMS in the form of sodium adducts.^21^ These findings indicate that not only Ag(I) but also other metal ions, e.g., Li^+^ and Na^+^, interacted differently with hydroxyls depending on their stereochemistry, further substantiating the claim that such interactions can be used to facilitate the distinction of stereoisomers.

### Analysis of Cannabis Extracts, Acid-Catalyzed CBD Mixtures, and Commercial Δ8-THC Edibles

#### Identification Workflow for Cannabinoids

**Figure 4:**
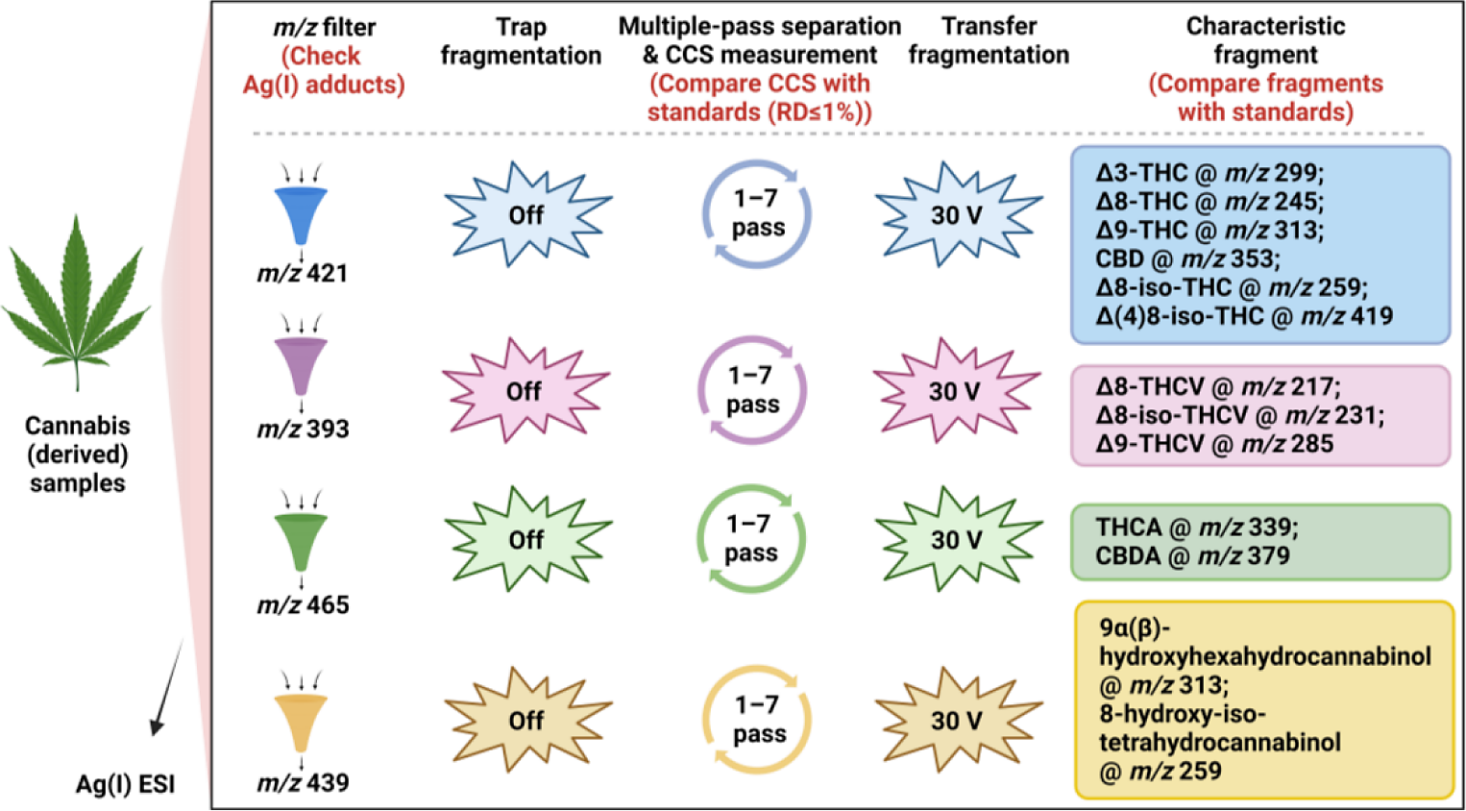
Qualitative identification workflow for cannabinoid isomers in a complex matrix.

Combining the aforementioned information, a workflow (Figure 4) for the identification of cannabinoids in complex samples was developed. Samples were mixed with a methanol AgNO_3_ solution and subjected to cIMS via direct infusion for full scan in positive ionization mode followed by multipass separation (1–7 passes) of selected ions (cannabinoid-Ag(I) species) to obtain the maximum number of peaks in a single pass. During this stage, the eCCS of each detected peak was calculated. Afterward, transfer fragmentation was performed at 30 V, at which pronounced diagnostic fragments were obtained for all investigated cannabinoids (the range of 20–40 V was tested, Figure S12). Finally, the identification procedure was used to check (i) precursor ions with Ag(I) isotope pattern (distinct [M+^107^Ag]^+^ and [M+^109^Ag]^+^ doublet with a ratio of approximately 1);^44^ (ii) eCCS of Ag(I) adducts (compared with reference standards); and (iii) MS/MS fragmentation (compared with reference standards). For the eCCS comparison with reference standards, the maximum RD was set at ±1%, considering the good intraday, interday, and interpass repeatability (RSD ≤ 0.3%, Table S6), despite ±2% being more commonly tolerated.^41^ This procedure applied a three-step check for the targeted analysis of the 14 cannabinoids investigated in this study. Detected signals that could not pass all steps of the check would be assigned as belonging to other compounds and in need of further investigations.

#### Qualitative Analysis of Cannabinoids in Samples

With the developed identification procedure, the distribution of 14 cannabinoids (four different MW) was investigated in Cannabis extracts, Δ8-THC gummies, and eight acid-treated CBD mixtures (Table S1 and Figure S14). The extracted ion chromatograms acquired with and without mobility separation demonstrate the necessity of ion mobility separation to resolve cannabinoids with the same molecular weight (Figures S14–1 to S14–5) when using direct infusion analysis. Besides, by checking Ag(I) isotope patterns, compounds that could not form Ag(I) adducts were easily excluded. The eCCS RD of detected cannabinoids in samples from specific standards were within ±0.7% (Table S7), much smaller than the reported CCS reproducibility of ±2% in literature.^41^ Subsequent comparison of characteristic fragments further improved the identification confidence. The screening results showed THCA (**10**), CBDA (**11**), Δ9-THC (**3**), CBD (**4**), and Δ9-THCV (**14**) were abundant cannabinoids in Cannabis extracts. Δ3-THC (**1**), Δ8-THC (**2**), Δ9-THC (**3**), Δ(4)8-iso-THC (**6**), 9α-hydroxyhexahydrocannabinol (**7**), and 9β-hydroxyhexahydrocannabinol (**8**) were found in Δ8-THC gummies. The largest number of cannabinoids that are isomers of THC/CBD that were detected in a single acid-treated CBD mixture was five. The identification results obtained by cIMS matched well with those obtained by UHPLC-UV/MS and GC-FID/MS^4^ (Figure S15), with the analysis time shortened from tens of minutes to milliseconds (actual separation), or—taking into account the actual machine use, within 3 min. It is noteworthy that the UHPLC-UV/MS method was unable to analyze acid-treated CBD mixtures with many cannabinoid isomers, and the GC-FID/MS method could not analyze acidic cannabinoids in Cannabis extracts due to thermal decomposition. However, with the cIMS method, the quite different samples could all be analyzed. Therefore, the developed cIMS method exhibited a unique combination of high accuracy, efficiency, and versatility for the qualitative analysis of cannabinoid samples.

#### Quantitative Analysis of Cannabinoids in Samples

**Figure 5:**
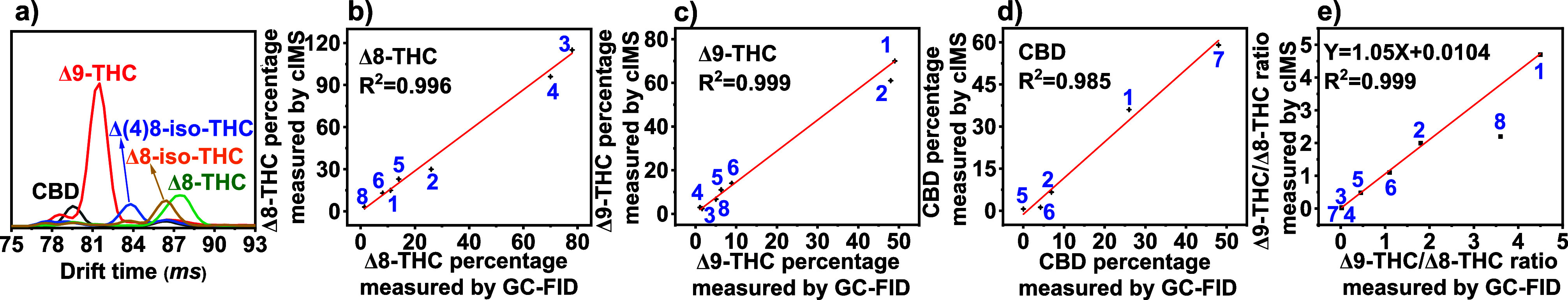
(a) Extracted mobiligram of the characteristic fragments in R#6 (EIC *m*/*z* 353 for CBD; EIC *m*/*z* 313 for Δ9-THC; EIC *m*/*z* 419 for Δ(4)8-iso-THC(×5); EIC *m*/*z* 259 for Δ8-iso-THC; EIC *m*/*z* 245 for Δ8-THC) after 4-pass separation; comparison of (b) absolute Δ8-THC percentage, (c) absolute Δ9-THC percentage, (d) absolute CBD percentage, and (e) the ratio of Δ9-THC/Δ8-THC in acid-treated CBD samples measured by the developed cIMS method and GC-FID method.

To explore the quantitative ability of the developed method, eight acid-treated CBD mixtures containing more isomeric cannabinoids than other samples investigated in this study were analyzed, and the major cannabinoids Δ8-THC (**2**), Δ9-THC (**3**), and CBD (**4**) were quantified as a proof of concept (Figure 5a, and S14). Calibration curves (Figure S16) showed excellent linearity for Δ8-THC (**2**) in the range of 1–250 ng·ml^–1^ (*R*^2^ = 0.9999), Δ9-THC (**3**) in the range of 0.1–25 ng·ml^–1^ (*R*^2^ = 0.9987), and CBD (**4**) in the range of 0.04–10 ng·ml^–1^ (*R*^2^ = 0.9986). Following that, the absolute weight percentages (w/w%, after solvent evaporation) of the three cannabinoids in the mixtures were compared to results obtained by GC-FID (Table S8).^4^ Plotting the results obtained by the two methods (Figure 5b–5d showed linear correlation (*R*^2^ > 0.985), but the cIMS method generally overestimated all three cannabinoids compared to the GC-FID method. Large deviations were observed for Δ8-THC (**2**) in R #8 (a deviation of 121%), Δ9-THC (**3**) in R #4 (RD of 164%), and CBD (**4**) in R #6 (RD of −71%), yet these deviations can be attributed to the low cannabinoid concentrations nearing the LOD of the GC-FID method. The systematic overestimation of the cIMS method could be attributed to matrix effects caused by competitive ionization, insufficient mobility separation, and the occurrence of diagnostic quantification fragments in other compounds. Particularly, for samples with multiple cannabinoid isomers, e.g., acid-treated CBD mixtures, a smaller number of separation passes was used to prevent wrap-around effects and thus resulted in overlapping peaks of isomers.

and thus to obvious overestimations. To improve the quantification performance, slicing of targeted peaks for more passes of separation might be a solution despite the sacrifice in time and operational simplicity. Moreover, data processing for subtracting signals from unresolved cannabinoids could be explored to further increase the quantification accuracy despite the complexity. Finally, the absence of an internal standard in the quantification of absolute cannabinoid content via MS fragments is another source of systematic errors.^45^ Therefore, using internal standards or determination of the ratio between two compounds with similar ionization efficiency might be a promising solution.^21,46^ With this in mind, we compared the ratio of Δ9-THC/Δ8-THC obtained by the cIMS method and the GC-FID method for the purposes of (i) evaluating the relative quantification capability of the developed cIMS method; (ii) checking whether the protocols of converting CBD (**4**) to Δ8-THC (**2**) mainly produced Δ8-THC (**2**); and (iii) checking whether illegal Δ9-THC (**3**) would be produced during Δ8-THC (**2**) production. As summarized in Table S9 and shown in Figure 5e, there was an excellent correlation (*R*^2^ = 0.999) of measured Δ9-THC/Δ8-THC ratios between the cIMS method and GC-FID method in all samples, except for R #8. There, the low concentrations of Δ8-THC and Δ9-THC, as shown in Figure 5b,c, resulted in a large difference (Δ9-THC/Δ8-THC = 3.6 and = 2.2 by GC-FID and cIMS, respectively). For other samples, both methods showed near-identical Δ9-THC/Δ8-THC ratios (slope = 1.05), demonstrating the capability of relative quantification. The Δ9-THC/Δ8-THC ratios also revealed that seven out of eight Δ8-THC production methods yielded Δ9-THC, and half of these methods produced more Δ9-THC than Δ8-THC (R #1, R #2, R #6, and R #8 with Δ9-THC/Δ8-THC ratio >1). If such mixtures are infused in Δ8-THC edibles, not surprisingly, these edibles would be problematic from forensic and health perspectives in terms of Δ9-THC, apart from being likely problematic regarding the presence of other compounds as well. Strikingly, Δ9-THC (**3**) was detected in both of the investigated commercial Δ8-THC gummies (Figure S14). In short, the developed cIMS method could be used for reliable relative quantification and has the potential for the direct analysis of Δ9-THC in commercial Δ8-THC samples.

Comparing the cIMS method with the gold standard GC-FID method, the cIMS method shows substantial advantages in terms of analysis time (∼150 ms vs ∼33 min) and sensitivity. LODs achieved with the cIMS method for Δ8-THC (**2**), Δ9-THC (**3**), and CBD (**4**) were 15, 75, and 1652 times lower than those achieved with the GC-FID method (Table S10). Even though the relative quantification performance was comparable to that of the GC-FID method, the absolute quantification ability remains to be improved. Further comparison with the very recent work using DMS^14^ can be done in terms of obtained LODs, which are 2–4 orders of magnitude lower in the current study with cIMS-qTOF-MS (0.008–0.2 ng·ml^–1^ in this study vs 10–20 ng·ml^–1^). The DMS-based method was used for the quantitative analysis of one Δ8-THC oil and one hemp oil, and the detected cannabinoid concentrations were compared to the commercially declared amounts. Even though the isomeric composition is relatively simple, with a maximum of three isomers, the RD between detected and claimed amounts varied from −2.1% to −82.5% across different cannabinoids. However, no validation with a chromatographic method was performed, which makes it challenging to pinpoint whether these deviations stem from method inaccuracies or incorrect product labeling.

## Conclusions

An ultrafast, ultrasensitive, and highly selective method using cIMS-qTOF-MS was developed for the analysis of Cannabis and Cannabis-derived samples. This method enabled the reliable identification of 14 cannabinoids with four different molecular weights, including acidic cannabinoids, neutral cannabinoids, and diastereoisomeric cannabinoids. This method can be expanded for more cannabinoids when reference standards are available. The analysis took milliseconds, and the full measurement of a sample roughly 3 min. Up to six isomeric cannabinoids in one sample could be separated, which was enough to resolve even the most complex cannabinoid mixture encountered in this study. The identification of these cannabinoids in complex samples was achieved by combining 3 molecular identifiers: Ag(I) isotope pattern and *m*/*z*, CCS values, and characteristic MS fragments. Moreover, experimental and theoretical CCS values of the cannabinoid-Ag(I) species were obtained for the first time. The eCCS values were very distinctive for the 14 cannabinoids, while the tCCS values, despite displaying only an average calculation error of 2.6%, yield species-to-species errors that are still too large for practical application, likely due to the small 3D structural differences of these cannabinoid isomers. Not only cannabinoids with C=C bonds but also cannabinoids without olefinic double bonds (three hydrated THC isomers) could be distinguished with the developed method by forming Ag^+^ or Na^+^ adducts. Most likely, different spatial orientations of hydroxyl groups result in different interactions with the metal ions. Through the examination of a diverse range of samples, including Cannabis extracts, commercial Δ8-THC edibles, and acid-treated CBD mixtures, the developed method demonstrated the ability to reliably identify and sensitively quantify cannabinoid isomers in complex matrixes in milliseconds rather than tens of minutes taken by the current gold standard UHPLC-UV/MS and GC-FID/MS methods. Besides, minimal solvent consumption is another merit. However, manual operation and high cost of the equipment as well as data interpretation need to be considered too, but to some extent, this is also true for chromatographic methods.

## Supporting Information Available

Preparation of acid-treated CBD mixtures; ^1^H NMR spectra and UHPLC-UV (215 nm) profile of isolated cannabinoids in this study; mobiligrams of cannabinoid mixture containing Δ3-THC, Δ8-THC, Δ9-THC, CBD, Δ8-iso-THC, and Δ(4)8-iso-THC as protonated, sodiated, and Ag(I) species; pre- and postmobility fragmentation of investigated cannabinoids; multipass CCS calibration, experimental CCS, CCS values from literature and AllCCS predicted CCS values; reversed-phase UHPLC-UV/MS and GC-FID/MS analysis of hydrated THC isomers and samples; comparison of CCS and fragments of detected cannabinoids in samples and cannabinoid standards; calibration curves of Δ8-THC, Δ9-THC, and CBD; comparison of Δ8-THC, Δ9-THC, and CBD percentages in acid-treated CBD samples measured by the developed cIMS method and GC-FID method (PDF)
