# Supplementary material for: Ultrafast, Selective, and Highly Sensitive Nonchromatographic Analysis of Fourteen Cannabinoids in Cannabis Extracts, Δ8-Tetrahydrocannabinol Synthetic Mixtures, and Edibles by Cyclic Ion Mobility Spectrometry–Mass Spectrometry
Source: Anal Chem. 2024 Jun 11;96(25):10170–81. doi: 10.1021/acs.analchem.3c05879 (PMC11209660; doi:10.1021/acs.analchem.3c05879)
Supplement: Supplementary file 1 — ac3c05879_si_001.pdf [file ac3c05879_si_001.pdf]

Supporting Information

Ultrafast, Selective and Highly Sensitive Non-Chromatographic Analysis of Fourteen Cannabinoids in Cannabis Extracts, Δ8-Tetrahydrocannabinol Synthetic Mixtures and Edibles by Cyclic Ion Mobility Spectrometry-Mass Spectrometry

Si Huang,[a,b] Laura Righetti,[b,c,*] Frank W. Claassen,[b] Akash Krishna,[b] Ming Ma,[a] Teris A. van Beek,[b] Bo Chen,[a,*] Han Zuilhof,[a,b,*] and Gert IJ. Salentijn[b,c,*]

[a]Key Laboratory of Phytochemical R&D of Hunan Province and Key Laboratory of Chemical Biology & Traditional Chinese Medicine Research of Ministry of Education, Hunan Normal University, No.36, Lushan Road, 410081, Changsha, China.

[b]Laboratory of Organic Chemistry, Wageningen University, Stippeneng 4, 6708 WE Wageningen, The Netherlands.

[c]Wageningen Food Safety Research (WFSR), Wageningen University & Research, P.O. Box 230, 6700 AE, Wageningen, The Netherlands.

E-mail corresponding authors:

laura.righetti@wur.nl; dr-chenpo@vip.sina.com; Han.Zuilhof@wur.nl; Gert.Salentijn@wur.nl.

Table of Contents

Table S1	Preparation of acid-treated CBD mixtures	Page S4
Table S2	Cannabinoid composition information of Cannabis and Δ8-THC gummies provided by merchants	Page S5
Figure S1	^{1}H NMR spectra of THCA, Δ9-THCV, Δ8-THCV, and Δ8-iso-THCV standards	Page S6-S10
Figure S2	Reversed phase UHPLC-UV (215 nm) profiles of Δ3-THC, Δ9-THCV, THCA, Δ8-THCV and Δ8-iso-THCV standards	Page S11
Figure S3	GC-FID profile of the mixture of Δ8-THCV and Δ8-iso-THCV, and EI-MS spectra of Δ8-THCV and Δ8-iso-THCV	Page S12-S14
Figure S4	Silica-Ag(Ⅰ) HPLC-DAD (215 nm) profile of the mixture of Δ8-THCV and Δ8-iso-THCV	Page S15
Table S3	Calibrants used for multi-pass CCS calibrations	Page S16
Figure S5	Calibration curves for drift time and CCS values	Page S16
Figure S6	Mobiligrams of the mixture of Δ8-THC, Δ9-THC, Δ3-THC, CBD, Δ8-iso-THC and Δ(4)8-iso-THC in protonated, sodiated, and Ag(I) species for different numbers of passes	Page S17
Figure S7	Characteristic fragments of investigated cannabinoids in the presence of Ag(I)	Page S18-S21
Figure S8	Mobiligram of the mixture of 8-hydroxy-iso-tetrahydrocannabinol, 9α-hydroxyhexahydrocannabinol, and 9β-hydroxyhexahydrocannabinol in the form of Ag(I) species.	Page S22
Figure S9	Reversed-phase UHPLC-ESI-Orbitrap mass spectra of hydrated THC isomers	Page S23
Figure S10	EI mass spectra of hydrated THC isomers after GC separation	Page S24
Figure S11	Mass spectra of protonated hydrated THC isomers, Ag(I) species of hydrated THC isomers, and pre-mobility fragmentation of hydrated THC isomers in the presence of Ag(I)	Page S25-S27
Table S4	Comparison of predicted CCS values by AllCCS, CCS values from references and experimental CCS values measured by cIMS of THC isomers as protonated species and sodiated species	Page S28
Figure S12	Post-mobility fragmentation of Ag(I) species of investigated cannabinoids after a 7-pass separation under different transfer energies	Page S29-S41
Figure S13	Pre- and post-mobility fragmentation of hydrated THC isomers in the presence of Ag(I)	Page S42-S44
Table S5	CCS of 9α-hydroxyhexahydrocannabinol, 9β-hydroxyhexahydrocannabinol, and 8-hydroxy-iso-THC as well as their dehydrated species in the presence of Ag(I)	Page S45
Table S6	Intra-day, inter-day, and inter-pass relative standard deviation of $[\Delta 8\text{-THC+Ag}]^+$ CCS	Page S46
Figure S14	Mobiligram and mass spectra of cannabinoids in samples	Page S47-S103
Table S7	CCS values of detected cannabinoids in acid-treated CBD mixtures	Page S104
Figure S15	Reversed-phase UHPLC-UV (215 nm) profile of samples C #1, C #2, and C #3 as well as GC-FID profile of samples G #1 and G #2	Page S105-S106
Figure S16	Calibration curves between the extracted ion chromatogram peak area of characteristic fragments and concentrations of Δ8-THC, Δ9-THC, and CBD	Page S107

Table S8	Absolute weight percentages of Δ8-THC, Δ9-THC, and CBD in acid-treated CBD mixtures by cIMS and GC-FID	Page S107
Table S9	Ratio of Δ9-THC/Δ8-THC in acid-treated CBD mixtures analyzed by cIMS and GC-FID	Page S108
Table S10	Comparison of LODs between the cIMS method and GC-FID method	Page S109
References		Page S110

Table S1. Preparation of acid-treated CBD mixtures.

#	Acid	Solvent	Temperature (°C)	Time (h)
1	pTSA 10% (mole ratio of acid/CBD=0.1)	Toluene	Room temperature	39
2	pTSA 200% (mole ratio of acid/CBD=2)	Toluene	Room temperature	39
3	pTSA 200% (mole ratio of acid/CBD=1)	DCM	Room temperature	43
4	pTSA 200% (mole ratio of acid/CBD=1)	Hexane	Room temperature	43
5	$BF_3 \cdot OEt_2$ (mole ratio of acid/CBD=1)	ACN	−10	6
6	H_2SO_4 (mole ratio of acid/CBD=1)	EtOH	70	39
7	acetic acid (mole ratio of acid/CBD=1)	EtOH	70	39
8	HCl (mole ratio of acid/CBD=1)	EtOH	70	39

Data are from our previous paper.[1]

Table S2. Cannabinoid composition information of Cannabis and Δ8-THC gummies provided by merchants.

Products	Cannabinoid composition information
C#1	Not available
C#2	THC 24.55%, CBD 7.72%; CBG 1.46%; THCA 6.17%; CBDA 2.43%; CBC 0.33%; THCV 2.31%; CBN 0.57%; CBL 0.
C#3	THC 1.05%; CBD 0; CBG 1.19%; THCA 19.31%; CBDA 0.02%; CBC 0.11%; THCV 0.63%; CBN 0.09%; CBL 0.
G#1	Δ8-THC 1 g; Δ9-THC<0.3%; CBD<0.3%
G#2	Δ8-THC 1.25%; Δ9-THC<0.3%

a)

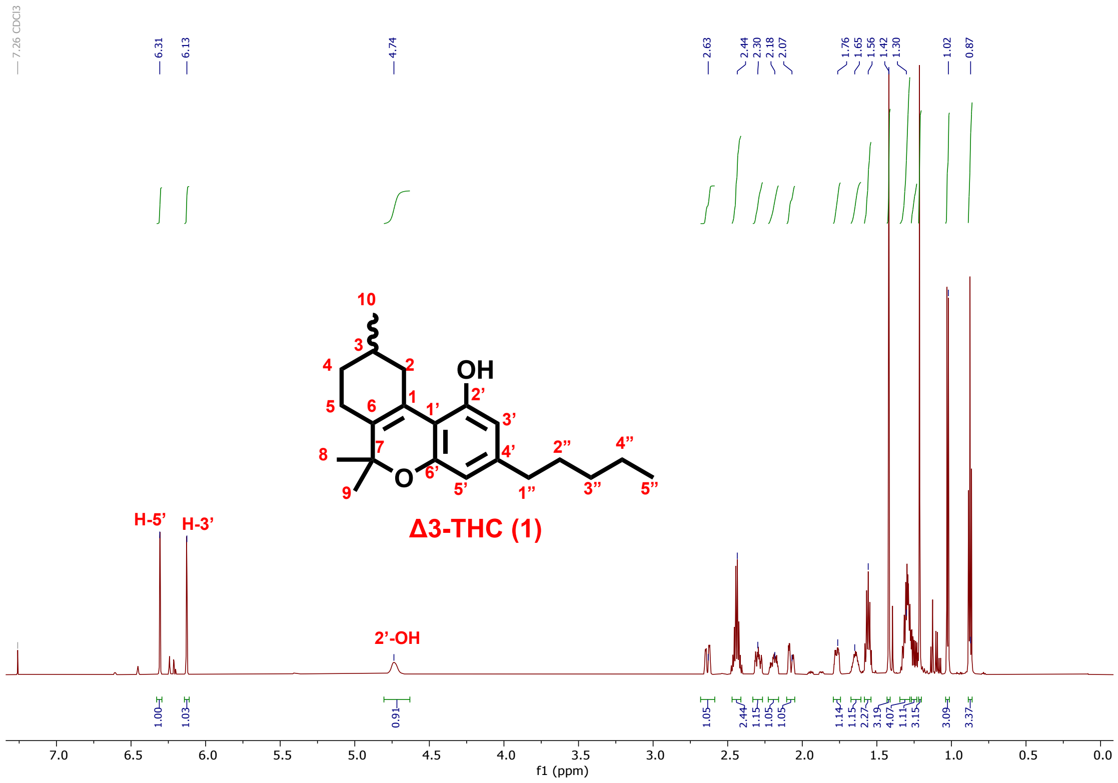
7.26 CDCl3
6.31
6.13
4.74
2.63
2.44
2.30
2.18
2.07
1.76
1.65
1.56
1.42
1.30
1.02
0.87
10
3
4
2
OH
2'
5
6
1
1'
3'
8
7
4'
2"
4"
O
6'
9
5'
1"
3"
5"
Δ3-THC (1)
H-5'
H-3'
2'-OH
1.00
1.03
0.91
1.05
2.44
1.15
1.05
1.05
1.14
1.15
2.27
3.19
4.07
1.11
3.15
3.09
3.37
7.0
6.5
6.0
5.5
5.0
4.5
4.0
3.5
3.0
2.5
2.0
1.5
1.0
0.5
0.0
f1 (ppm)

Zoom in

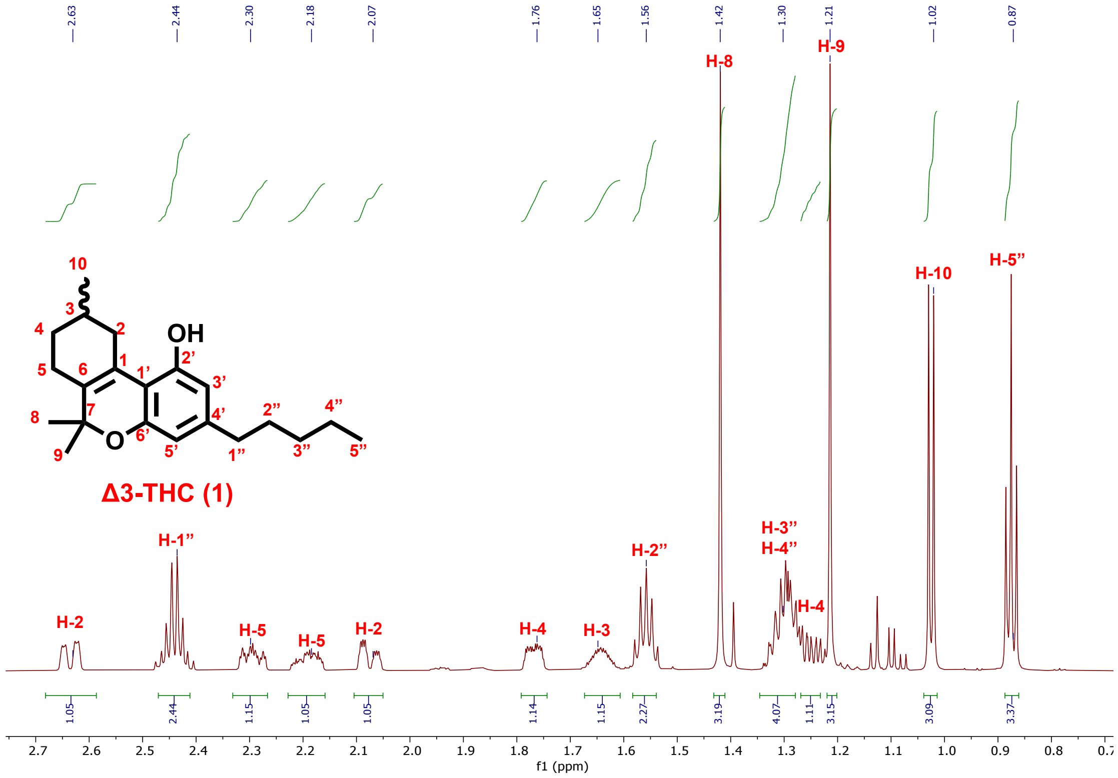
2.63
2.44
2.30
2.18
2.07
1.76
1.65
1.56
1.42
1.30
1.21
1.02
0.87
H-8
H-9
H-10
H-5"
10
3
4
2
OH
2'
5
6
1
1'
3'
8
7
4'
2"
4"
O
6'
9
5'
1"
3"
5"
Δ3-THC (1)
H-1"
H-2"
H-3"
H-4"
H-4
H-2
H-5
H-5
H-2
H-4
H-3
1.05
2.44
1.15
1.05
1.05
1.14
1.15
2.27
3.19
4.07
1.11
3.15
3.09
3.37
2.7
2.6
2.5
2.4
2.3
2.2
2.1
2.0
1.9
1.8
1.7
1.6
1.5
1.4
1.3
1.2
1.1
1.0
0.9
0.8
0.7
f1 (ppm)

b)

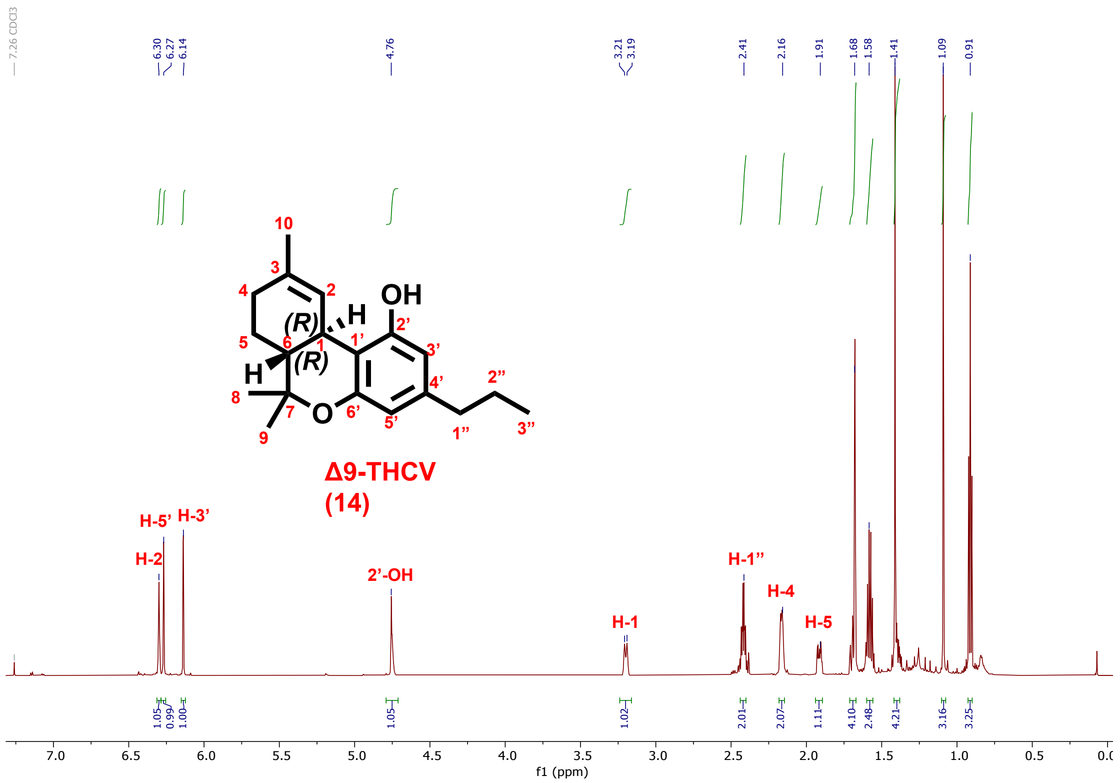
7.26 CDCl3
6.30
6.27
6.14
4.76
3.21
3.19
2.41
2.16
1.91
1.68
1.58
1.41
1.09
0.91
Δ9-THCV
(14)
H-5'
H-3'
H-2
2'-OH
H-1
H-1"
H-4
H-5
1.05
0.99
1.00
1.05
1.02
2.01
2.07
1.11
4.10
2.48
4.21
3.16
3.25
f1 (ppm)

Zoom in

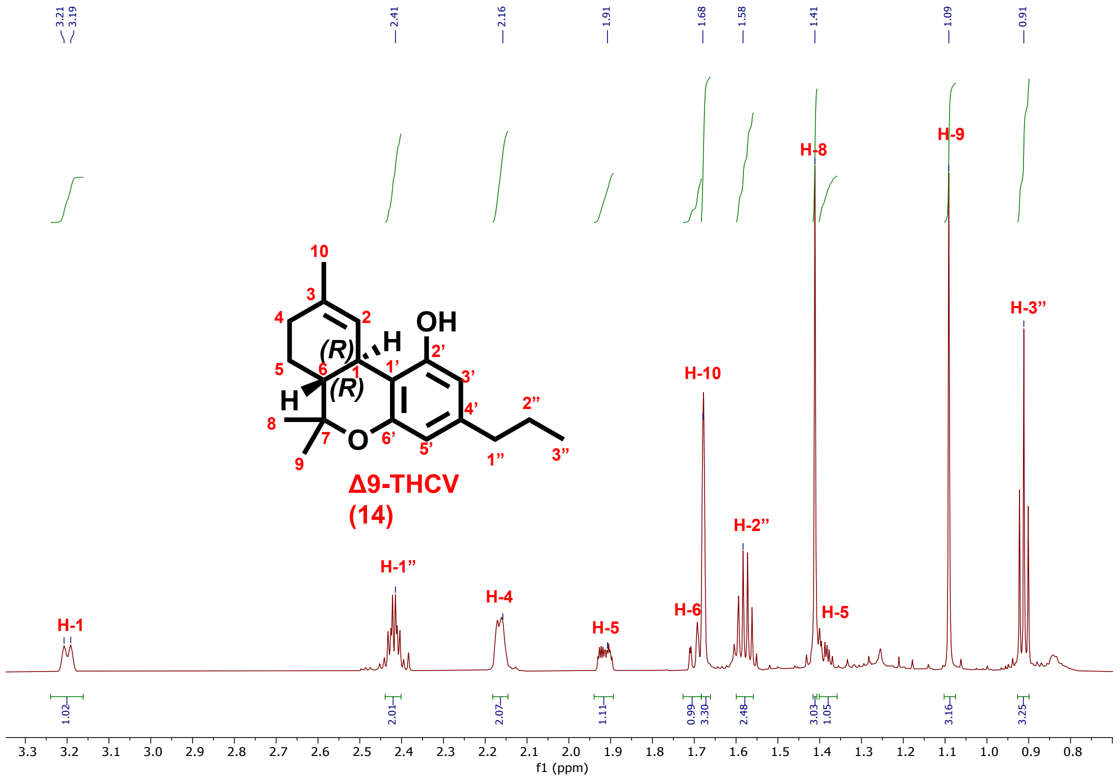
3.21
3.19
2.41
2.16
1.91
1.68
1.58
1.41
1.09
0.91
Δ9-THCV
(14)
H-8
H-9
H-3"
H-10
H-2"
H-1"
H-4
H-5
H-6
H-5
H-1
1.02
2.01
2.07
1.11
0.99
3.30
2.48
3.03
1.05
3.16
3.25
f1 (ppm)

c)

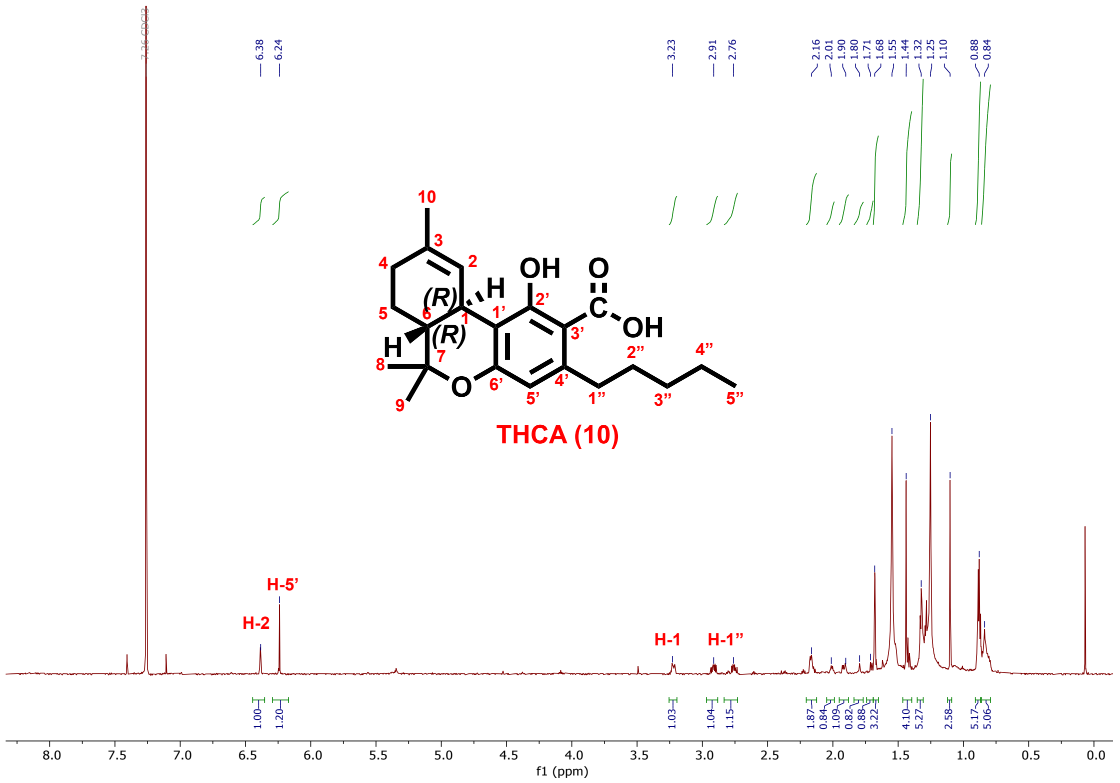
6.38
6.24
3.23
2.91
2.76
2.16
2.01
1.90
1.80
1.71
1.68
1.55
1.44
1.32
1.25
1.10
0.88
0.84
THCA (10)
H-5'
H-2
H-1
H-1"
1.00
1.20
1.03
1.04
1.15
1.87
0.84
1.09
0.82
0.88
3.22
4.10
5.27
2.58
5.17
5.06
f1 (ppm)

Zoom in

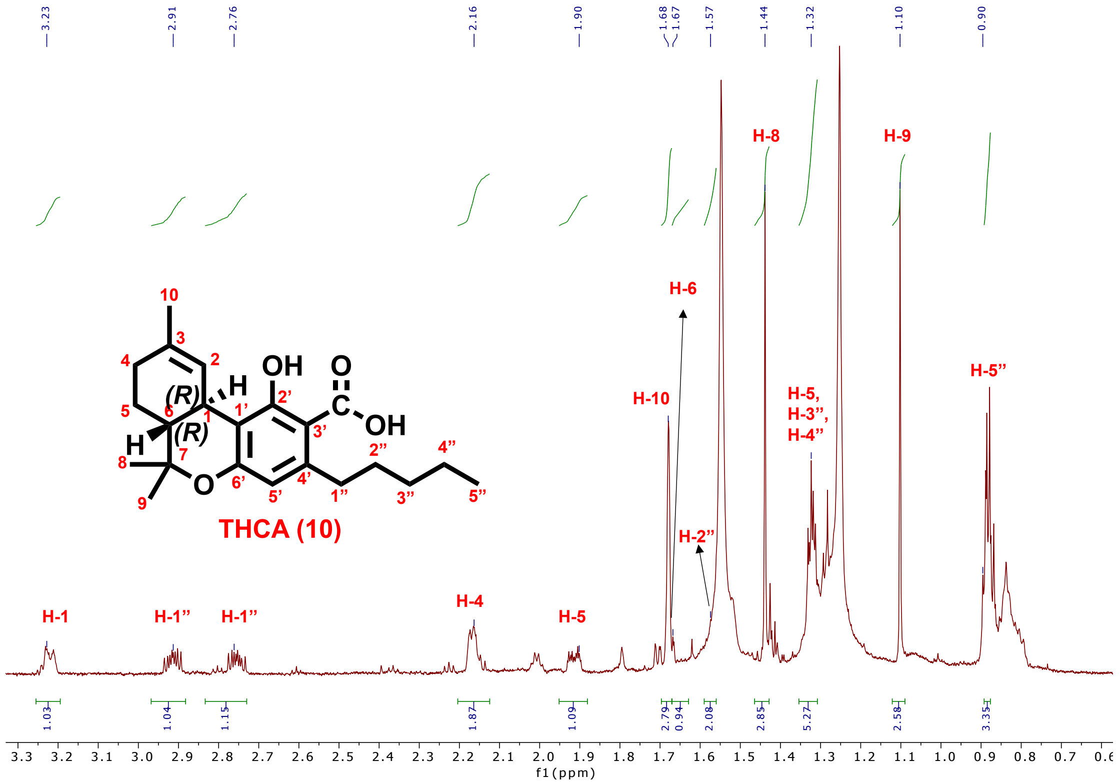
3.23
2.91
2.76
2.16
1.90
1.68
1.67
1.57
1.44
1.32
1.10
0.90
H-8
H-9
H-6
H-10
H-5,
H-3",
H-4"
H-5"
H-2"
THCA (10)
H-1
H-1"
H-1"
H-4
H-5
1.03
1.04
1.15
1.87
1.09
2.79
0.94
2.08
2.85
5.27
2.58
3.35
f1 (ppm)

d)

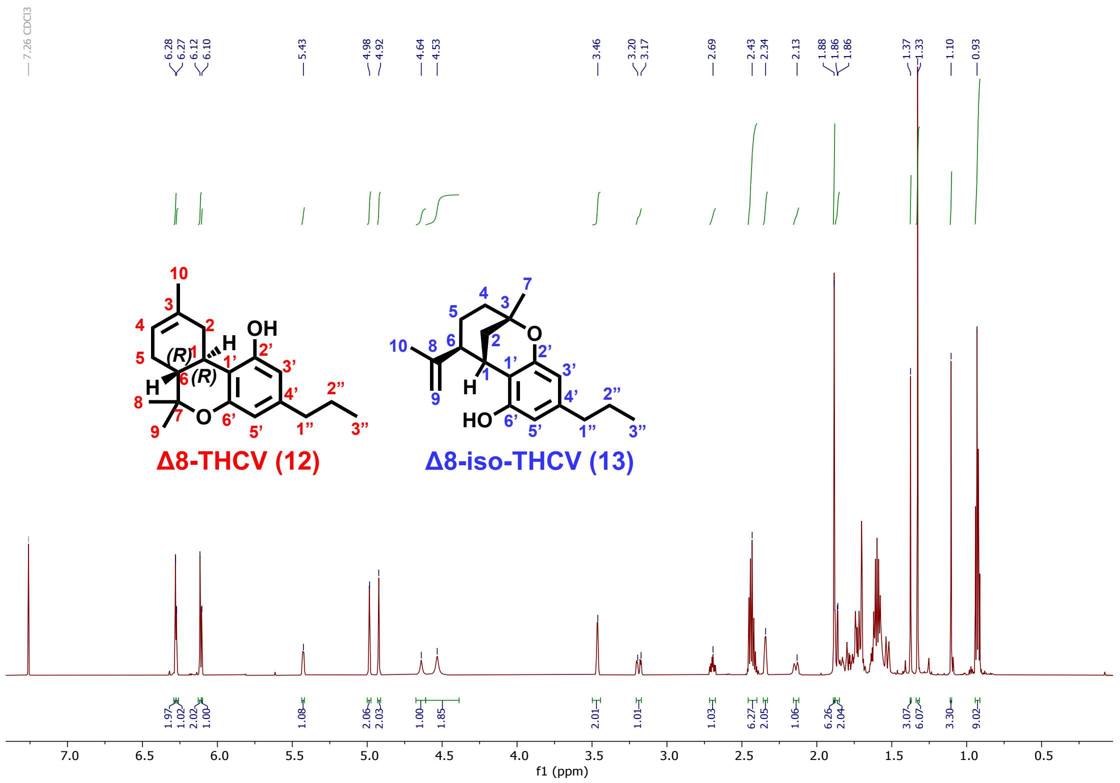

Zoom in

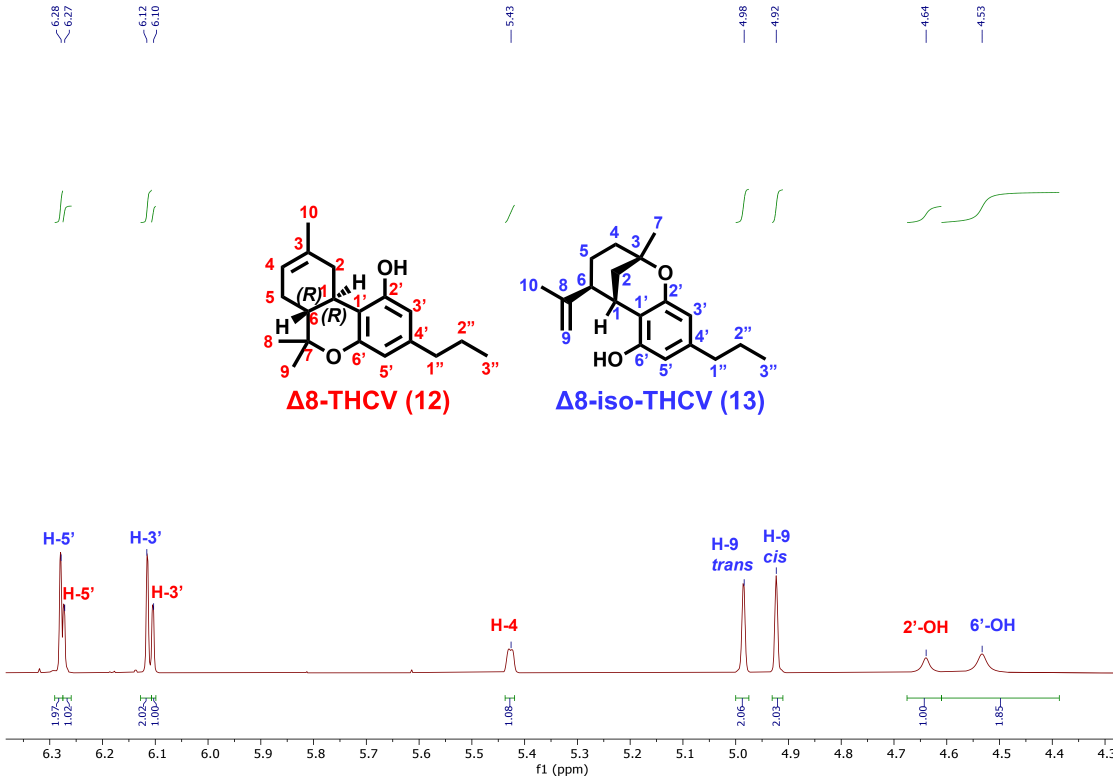

Zoom in

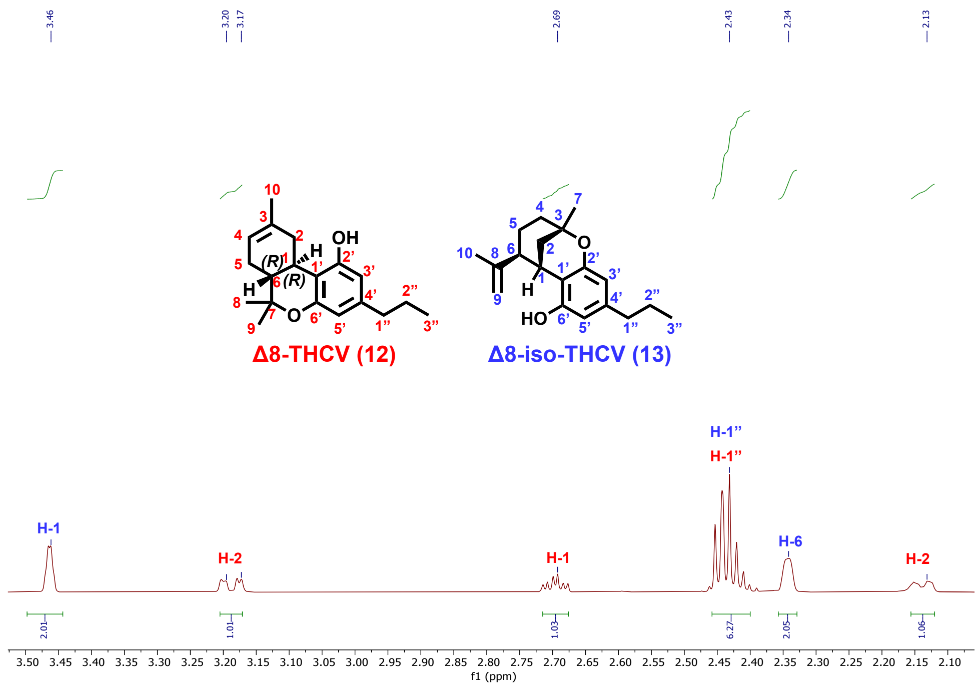

Zoom in

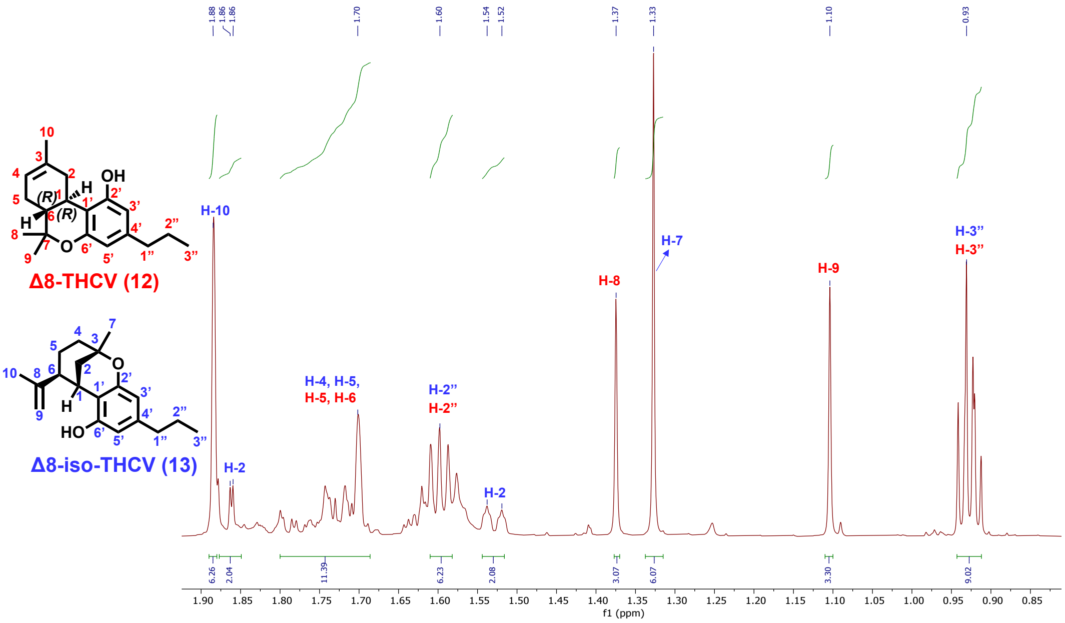

Figure S1. ^{1}H NMR spectra of a) Δ3-THC, b) Δ9-THCV, c) THCA, and d) a mixture of Δ8-THCV and Δ8-iso-THCV.

5 mg of Δ3-THC (**1**), Δ9-THCV (**14**), THCA (**10**), or a mixture of Δ8-THCV and Δ8-iso-THCV (**12, 13**) were dissolved in $CDCl_3$, and ^{1}H NMR spectra were recorded on a Bruker Avance 700 MHz spectrometer at 298 K. The assignments are based on data by Cheng et al.,[2] Srebnik et al.,[3] Dadiotis et al.,[4] Radwan et al.[5]

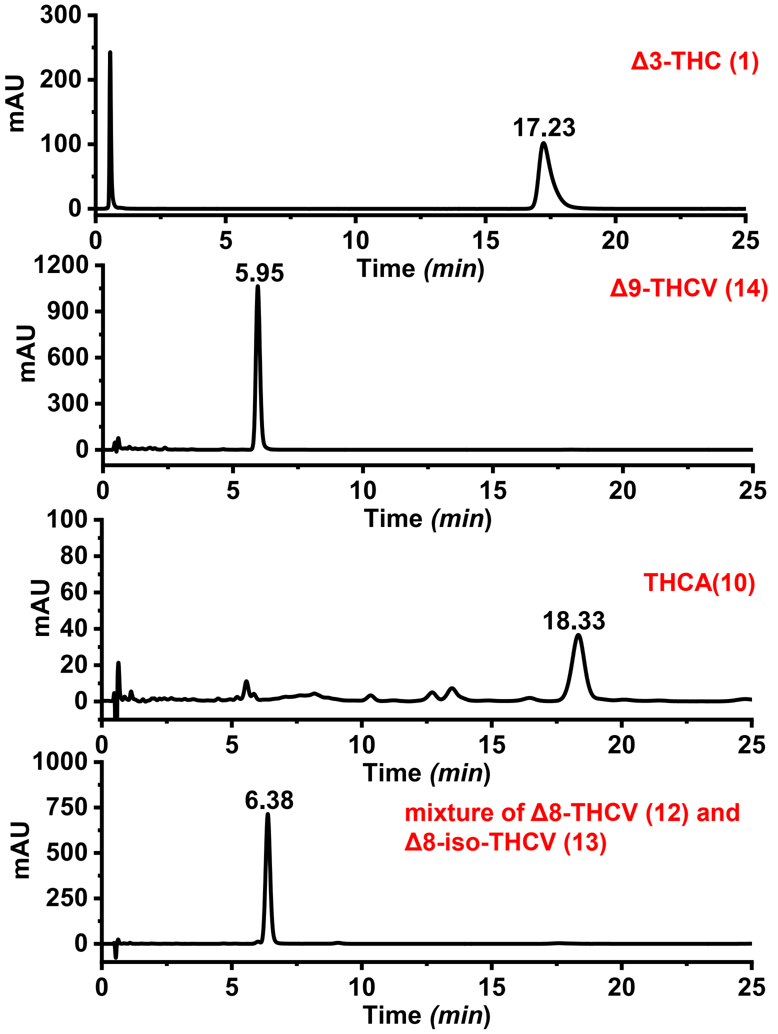

Figure S2. RP-UHPLC-UV (215nm) chromatograms of Δ3-THC, Δ9-THCV, THCA, and mixture of Δ8-THCV and Δ8-iso-THCV.

A Zorbax Eclipse Plus C18 chromatographic column (2.1 mm × 100 mm, particle size: 1.8 µm; Agilent Technologies, Santa Clara, CA, USA) was integrated with a 1290 Infinity ultra-performance liquid chromatography (UHPLC) system (Agilent Technologies, Santa Clara, United States) employing Diode Array Detection (DAD) as the detection method. The mobile phase employed was a solution comprising 5 mM formic acid in water (mobile phase A) and acetonitrile (mobile phase B). Isocratic elution was achieved by maintaining a constant ratio of 58% mobile phase B at a flow rate of 0.50 mL·min^{-1}. An aliquot of purified Δ3-THC (**1**), Δ9-THCV (**14**), THCA (**10**), and a mixture of Δ8-THCV (**12**) and Δ8-iso-THCV (**13**) was sampled with the tip of a spatula and dissolved in MeOH. Subsequently, 8 µL of each sample was injected for the analysis.

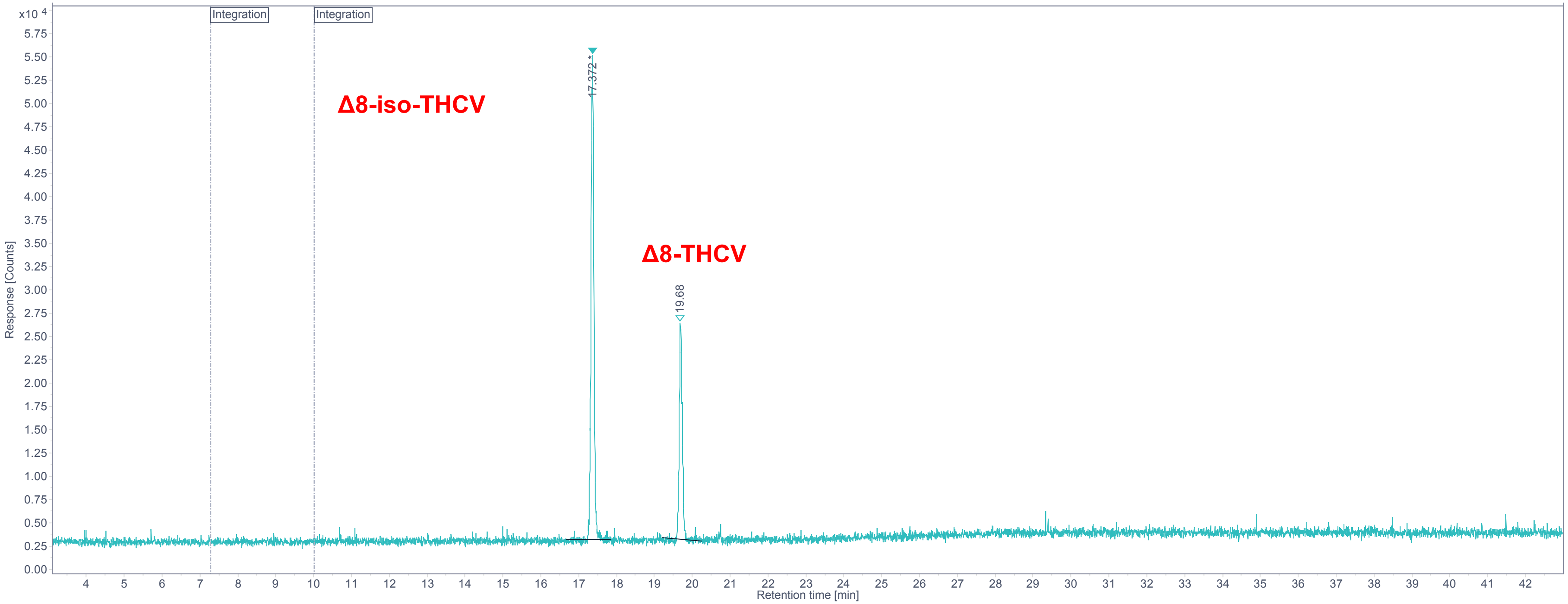

x10 4
5.75
5.50
5.25
5.00
4.75
4.50
4.25
4.00
3.75
3.50
3.25
3.00
2.75
2.50
2.25
2.00
1.75
1.50
1.25
1.00
0.75
0.50
0.25
0.00
Response [Counts]
Integration
Integration
Δ8-iso-THCV
17.372 *
Δ8-THCV
19.68
4 5 6 7 8 9 10 11 12 13 14 15 16 17 18 19 20 21 22 23 24 25 26 27 28 29 30 31 32 33 34 35 36 37 38 39 40 41 42
Retention time [min]

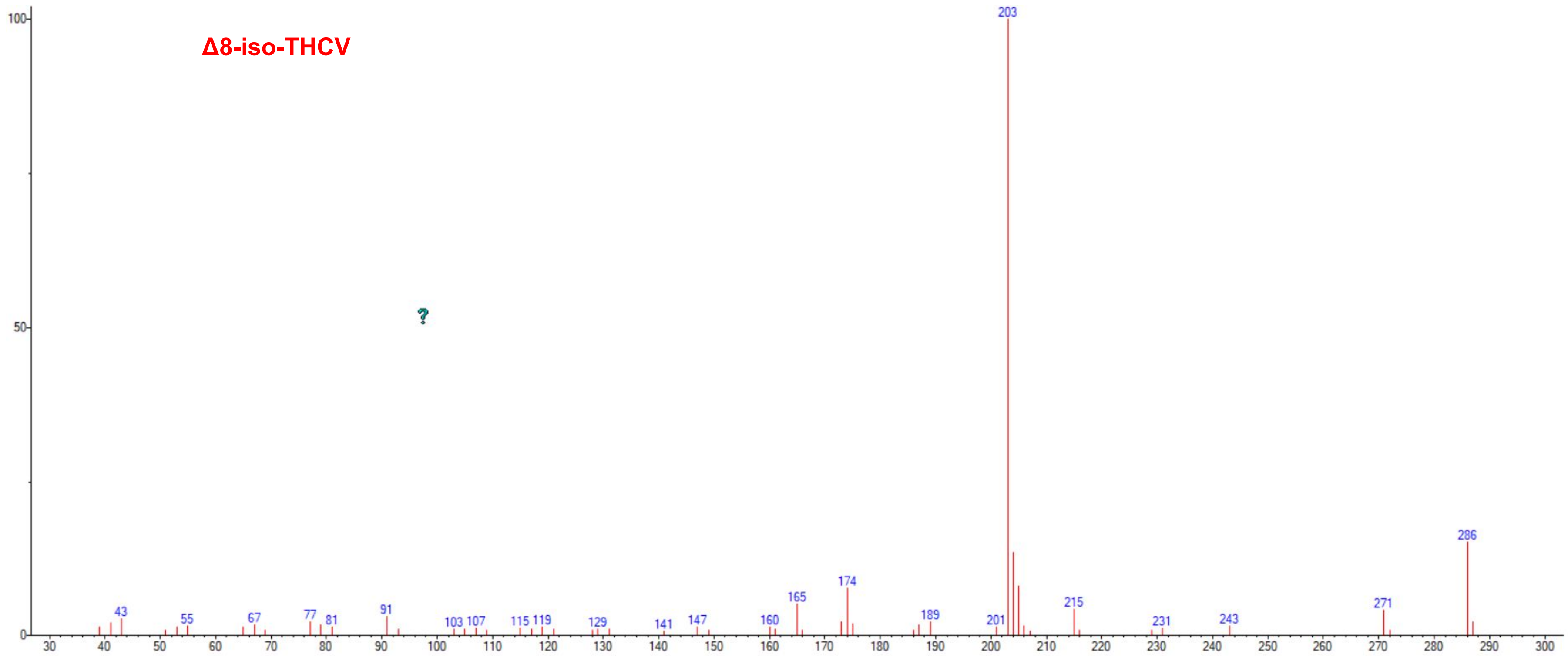
Δ8-iso-THCV
100
50
0
203
286
174
165
215
271
43
55
67
77
81
91
103
107
115
119
129
141
147
160
189
201
231
243
30
40
50
60
70
80
90
100
110
120
130
140
150
160
170
180
190
200
210
220
230
240
250
260
270
280
290
300

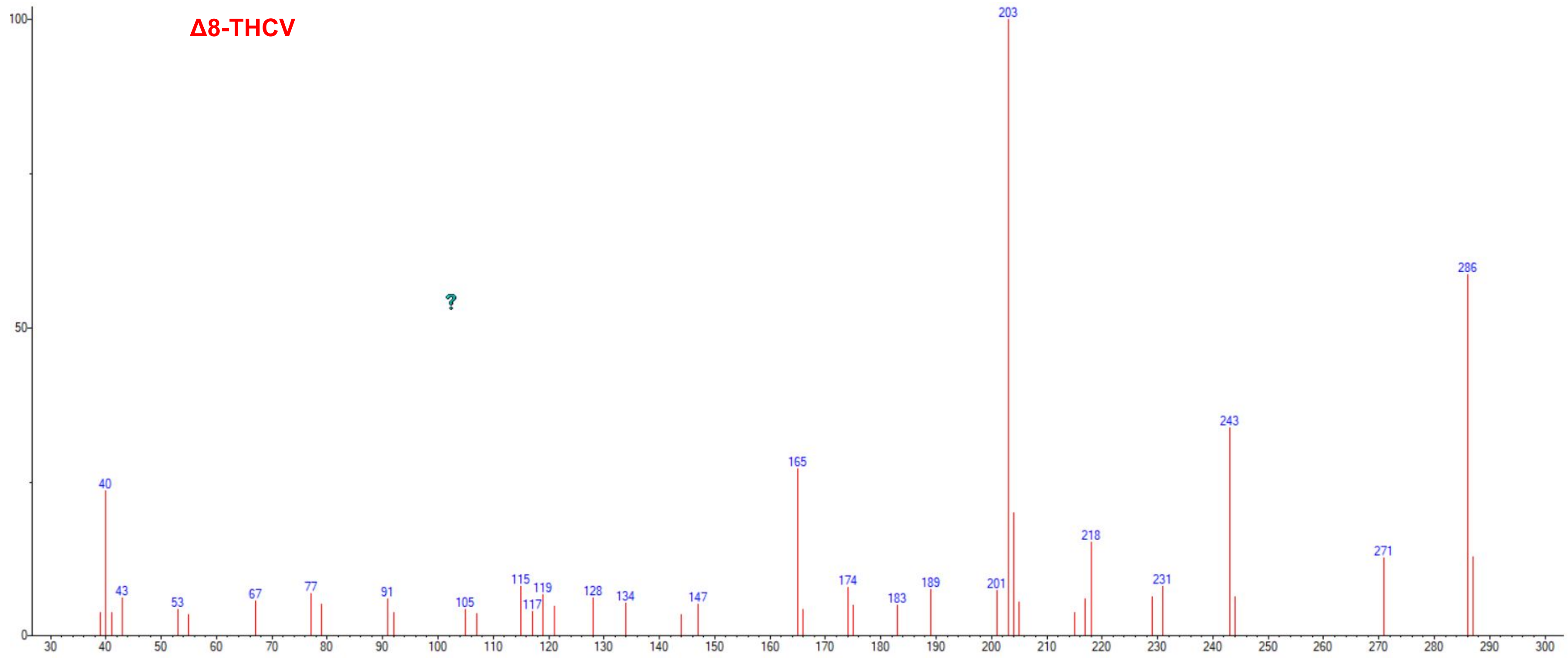

Figure S3. GC-FID profile of the mixture of Δ8-THCV and Δ8-iso-THCV, and EI-MS spectra of Δ8-THCV and Δ8-iso-THCV.

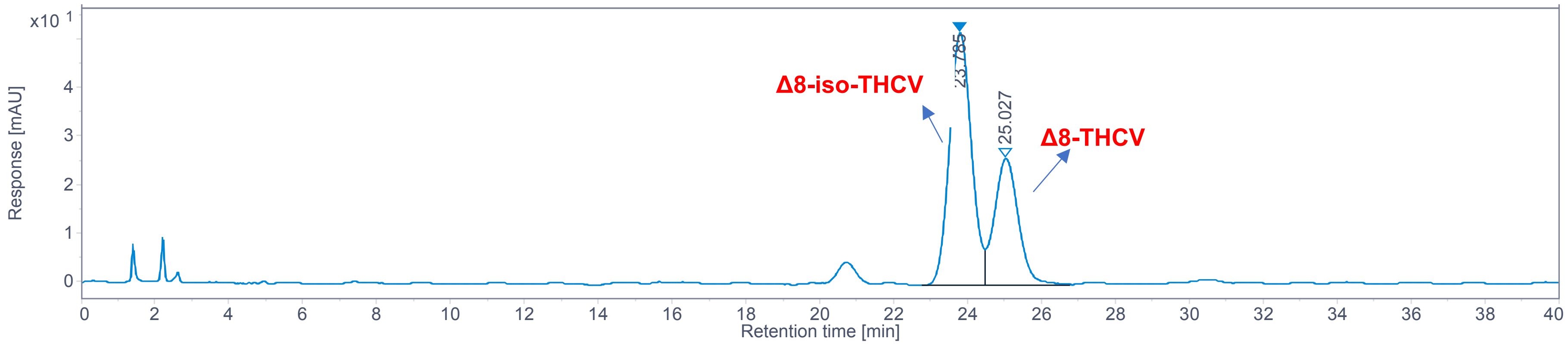

Figure S4. Silica-Ag(Ⅰ) HPLC-DAD (215 nm) profile of the mixture Δ8-THCV and Δ8-iso-THCV.

Table S3. Calibrants used for multi-pass CCS calibrations.

Major mix calibrant	Positive ionization mode	
	m/z	CCS (Å^2)
Sulphaguanidine	215.5970	146.8
Sulfadimethoxine	311.0809	168.4
Val-Tyr-Val	380.2180	191.7
Verapamil	455.2904	208.8
Terfenadine	472.3210	228.7
Reserpine	609.2807	252.3

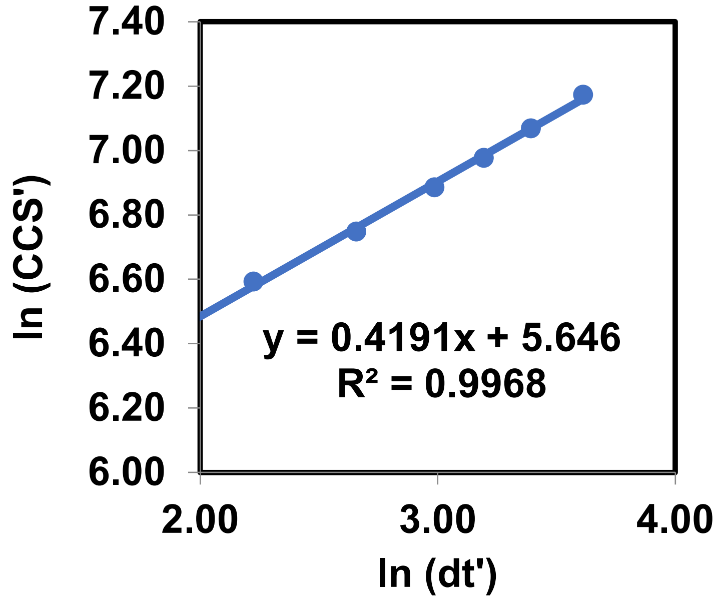

Figure S5. The logarithmic fit calibration curve between drift time and CCS values.

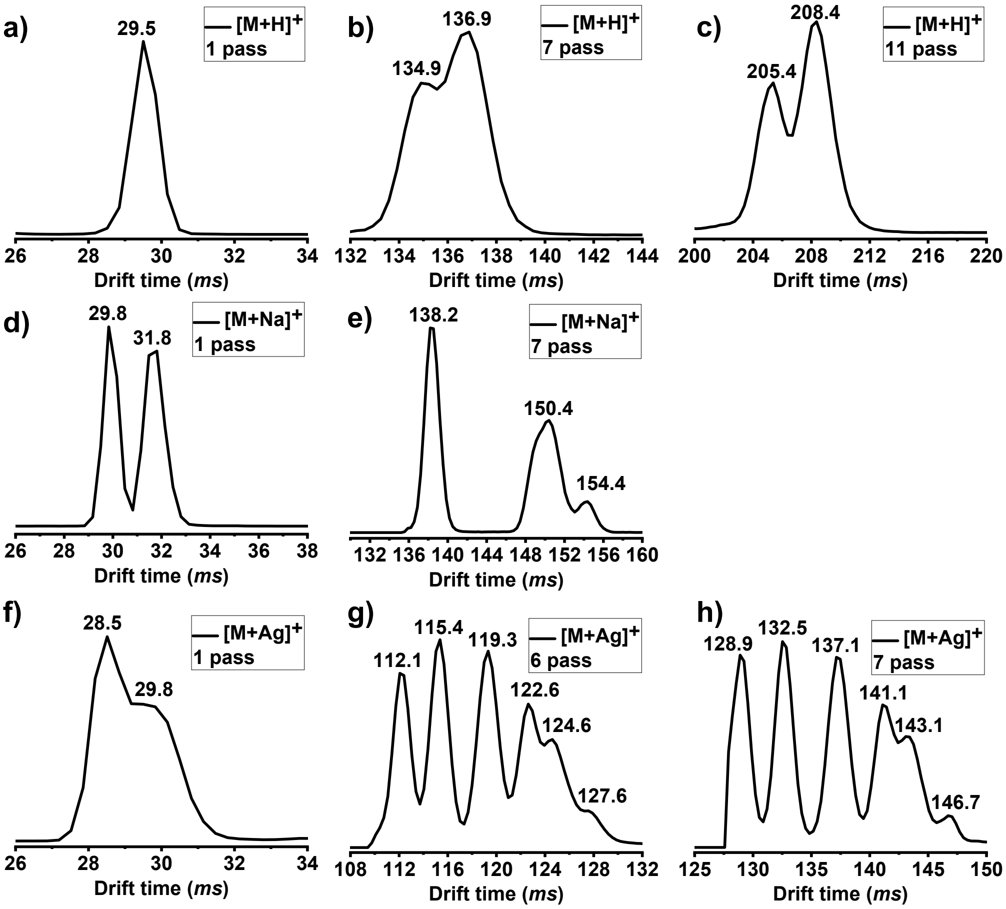

Figure S6. Mobiligrams of charged adducts of the mixture of Δ3-THC, Δ8-THC, Δ9-THC, CBD, Δ8-iso-THC and Δ(4)8-iso-THC. Protonated species (extracting $[M+H]^+$ signal at *m/z* 315) after a) 1 pass, b) 7 passes and c) 11 passes. Sodiated species (extracting $[M+Na]^+$ signal at *m/z* 337) after d) 1 pass and e) 7 passes. Argentated species (extracting $[M+Ag]^+$ signal at *m/z* 421) after f) 1 pass, g) 6 passes and h) 7 passes.

a)

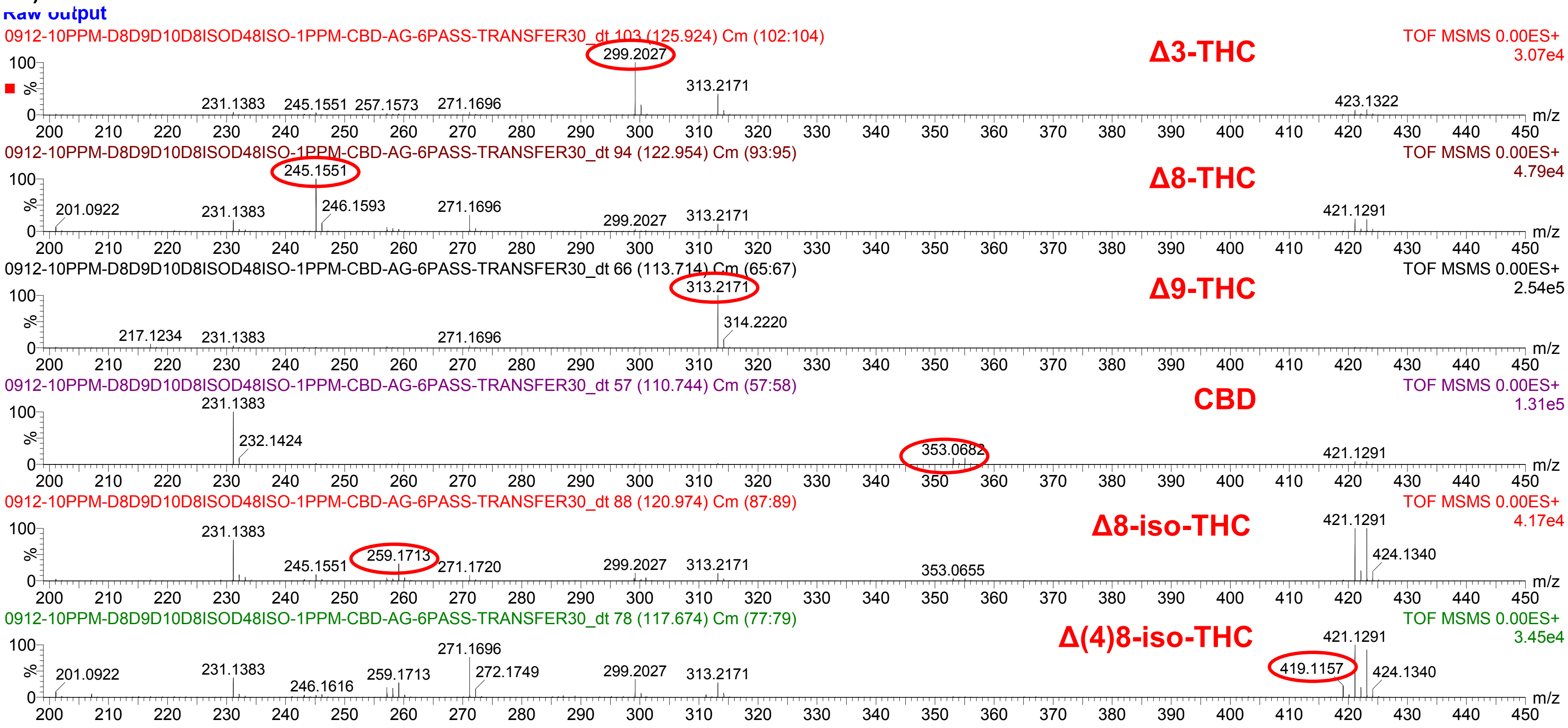
Raw output
0912-10PPM-D8D9D10D8ISOD48ISO-1PPM-CBD-AG-6PASS-TRANSFER30_dt 103 (125.924) Cm (102:104)
TOF MSMS 0.00ES+ 3.07e4
Δ3-THC
299.2027
313.2171
231.1383
245.1551
257.1573
271.1696
423.1322
0912-10PPM-D8D9D10D8ISOD48ISO-1PPM-CBD-AG-6PASS-TRANSFER30_dt 94 (122.954) Cm (93:95)
TOF MSMS 0.00ES+ 4.79e4
Δ8-THC
245.1551
201.0922
231.1383
246.1593
271.1696
299.2027
313.2171
421.1291
0912-10PPM-D8D9D10D8ISOD48ISO-1PPM-CBD-AG-6PASS-TRANSFER30_dt 66 (113.714) Cm (65:67)
TOF MSMS 0.00ES+ 2.54e5
Δ9-THC
313.2171
314.2220
217.1234
231.1383
271.1696
0912-10PPM-D8D9D10D8ISOD48ISO-1PPM-CBD-AG-6PASS-TRANSFER30_dt 57 (110.744) Cm (57:58)
TOF MSMS 0.00ES+ 1.31e5
CBD
231.1383
232.1424
353.0682
421.1291
0912-10PPM-D8D9D10D8ISOD48ISO-1PPM-CBD-AG-6PASS-TRANSFER30_dt 88 (120.974) Cm (87:89)
TOF MSMS 0.00ES+ 4.17e4
Δ8-iso-THC
231.1383
245.1551
259.1713
271.1720
299.2027
313.2171
353.0655
421.1291
424.1340
0912-10PPM-D8D9D10D8ISOD48ISO-1PPM-CBD-AG-6PASS-TRANSFER30_dt 78 (117.674) Cm (77:79)
TOF MSMS 0.00ES+ 3.45e4
Δ(4)8-iso-THC
201.0922
231.1383
246.1616
259.1713
271.1696
272.1749
299.2027
313.2171
419.1157
421.1291
424.1340
m/z
%

b)

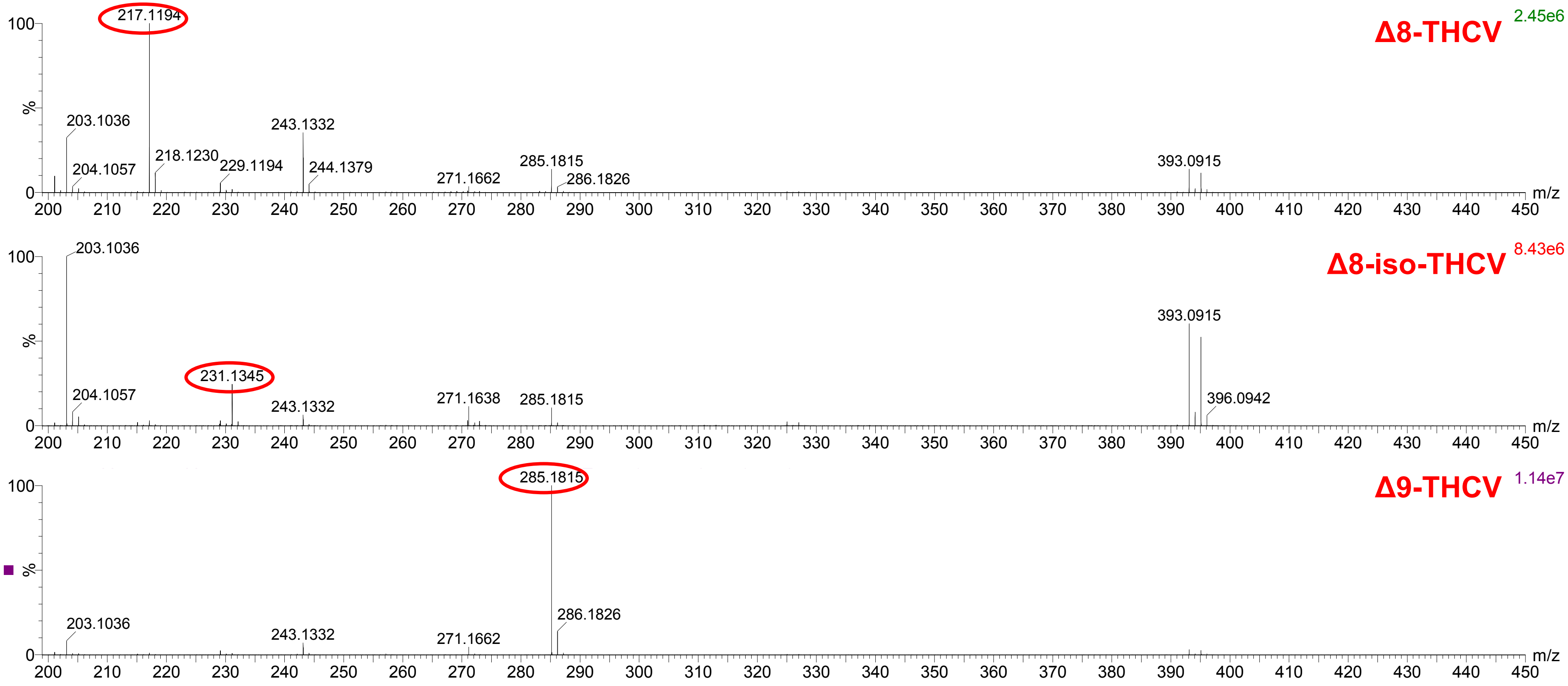
Δ8-THCV
2.45e6
217.1194
203.1036
243.1332
204.1057
218.1230
229.1194
244.1379
271.1662
285.1815
286.1826
393.0915
Δ8-iso-THCV
8.43e6
203.1036
393.0915
231.1345
204.1057
243.1332
271.1638
285.1815
396.0942
Δ9-THCV
1.14e7
285.1815
203.1036
243.1332
271.1662
286.1826
100
%
0
m/z
200
210
220
230
240
250
260
270
280
290
300
310
320
330
340
350
360
370
380
390
400
410
420
430
440
450

c)

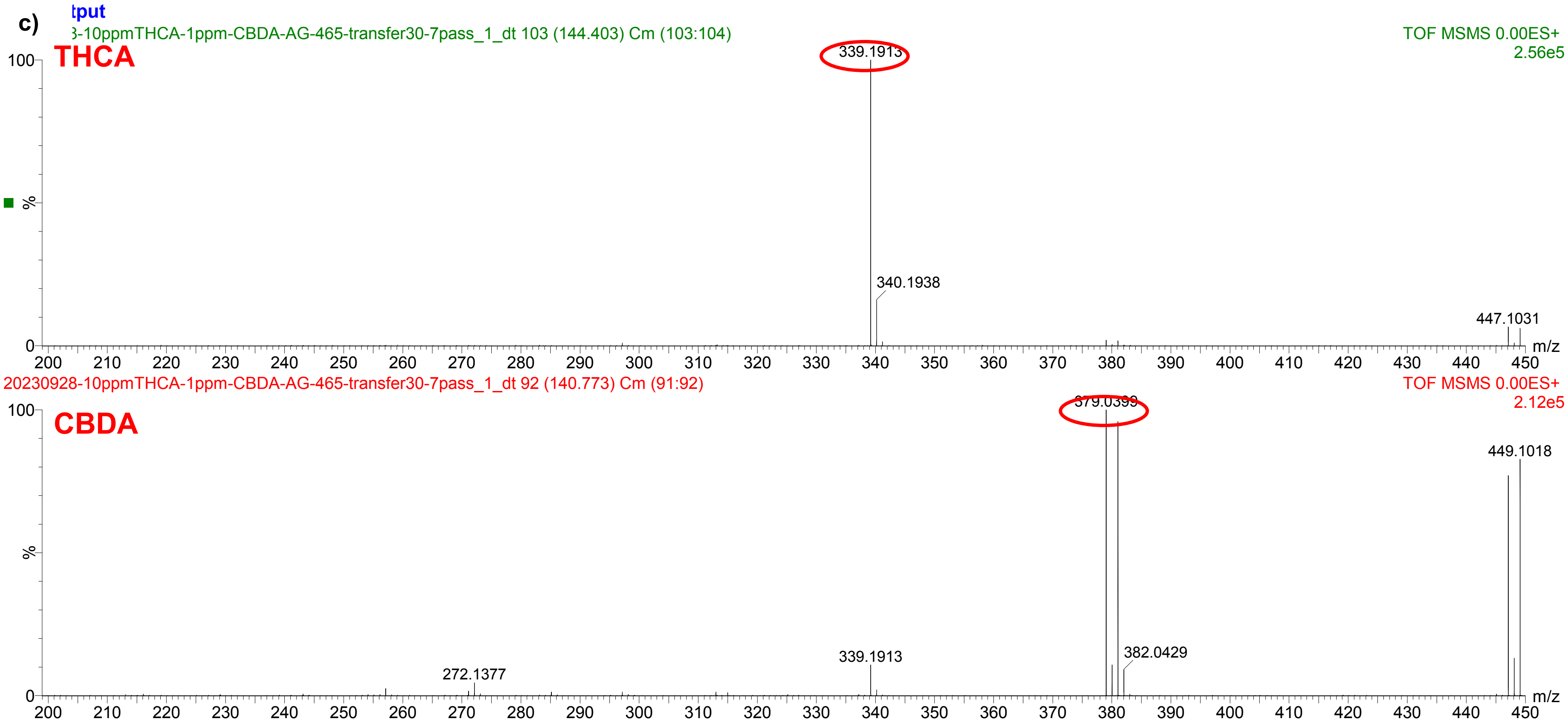
tput
3-10ppmTHCA-1ppm-CBDA-AG-465-transfer30-7pass_1_dt 103 (144.403) Cm (103:104)
TOF MSMS 0.00ES+
2.56e5
THCA
339.1913
340.1938
447.1031
20230928-10ppmTHCA-1ppm-CBDA-AG-465-transfer30-7pass_1_dt 92 (140.773) Cm (91:92)
TOF MSMS 0.00ES+
2.12e5
CBDA
379.0399
449.1018
382.0429
339.1913
272.1377
m/z
%

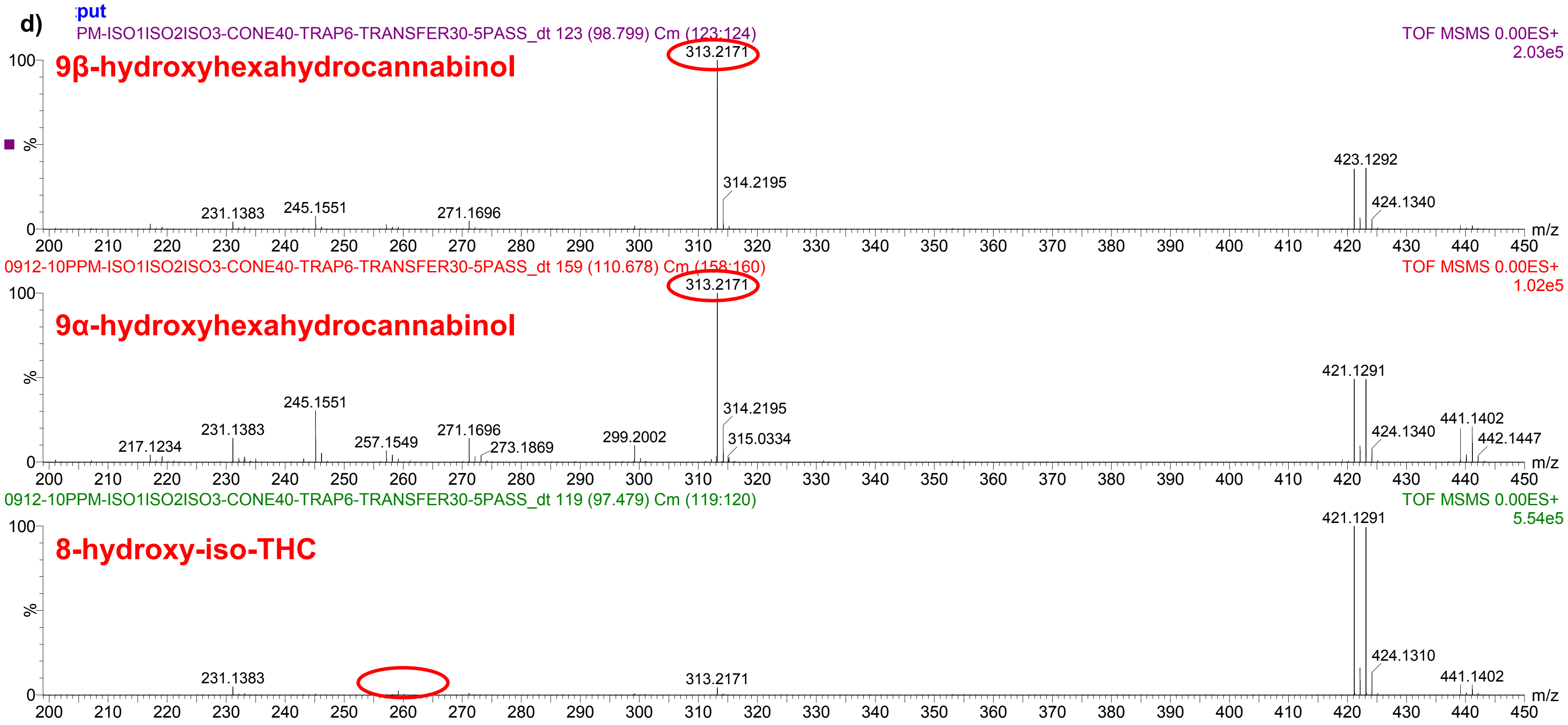

Figure S7. Characteristic fragments of a) Δ8-THC, Δ9-THC, Δ3-THC, CBD, Δ8-iso-THC and Δ(4)8-iso-THC; b) Δ8-THCV, Δ8-iso-THCV and Δ9-THCV; c) THCA and CBDA; d) 9α-hydroxyhexahydrocannabinol, 9β-hydroxyhexahydrocannabinol and 8-hydroxy-iso-THC in the presence of Ag(I) at trap energy of 30 V.

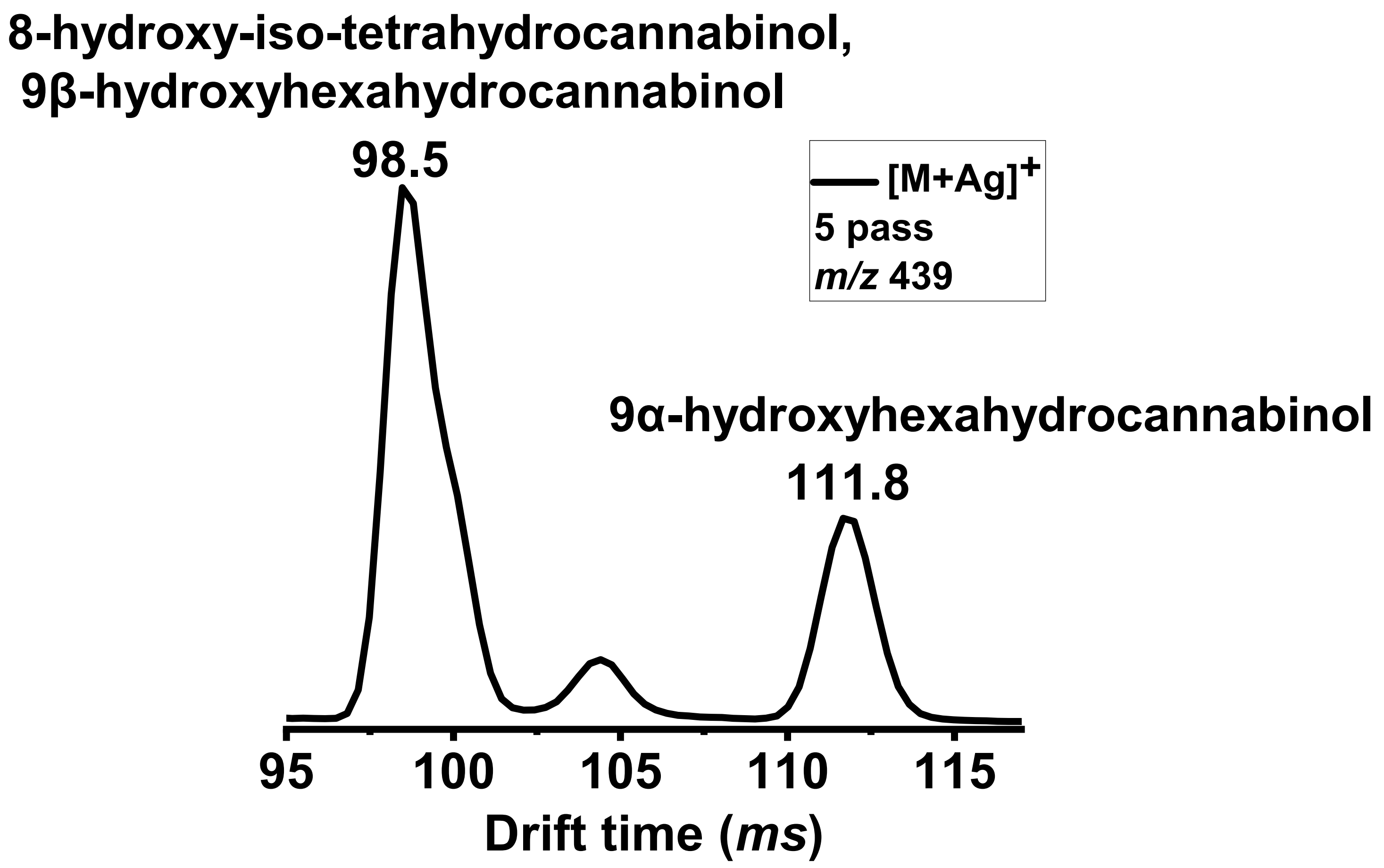

Figure S8. Mobiligram of the mixture consisting of 8-hydroxy-iso-THC, 9α-hydroxyhexahydrocannabinol, and 9β-hydroxyhexahydrocannabinol in the form of Ag(I) species.

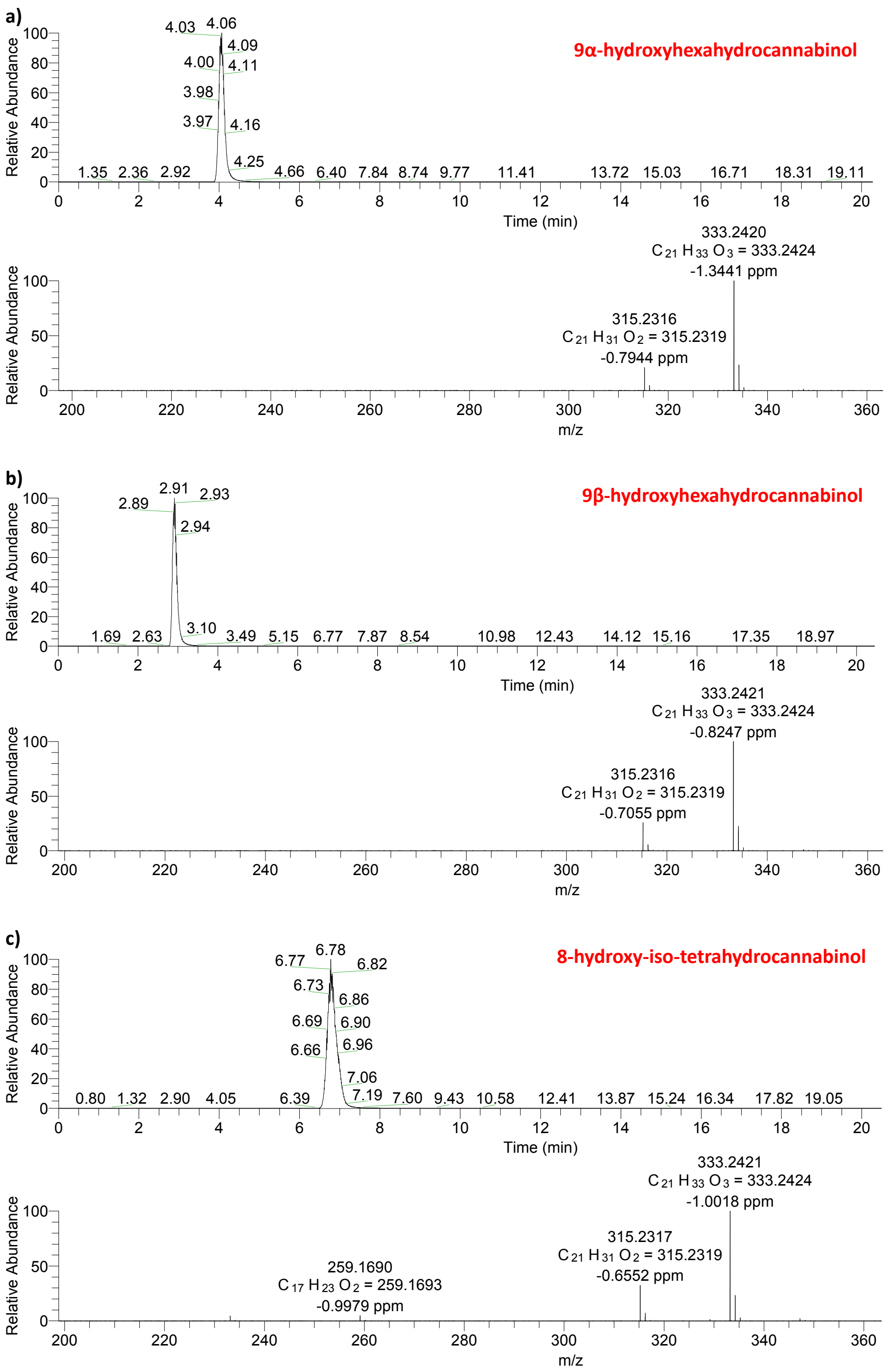

Figure S9. Reversed-phase UHPLC-ESI-Orbitrap mass spectra of a) 9α-hydroxyhexahydrocannabinol, b) 9β-hydroxyhexahydrocannabinol, and c) 8-hydroxy-iso-THC.

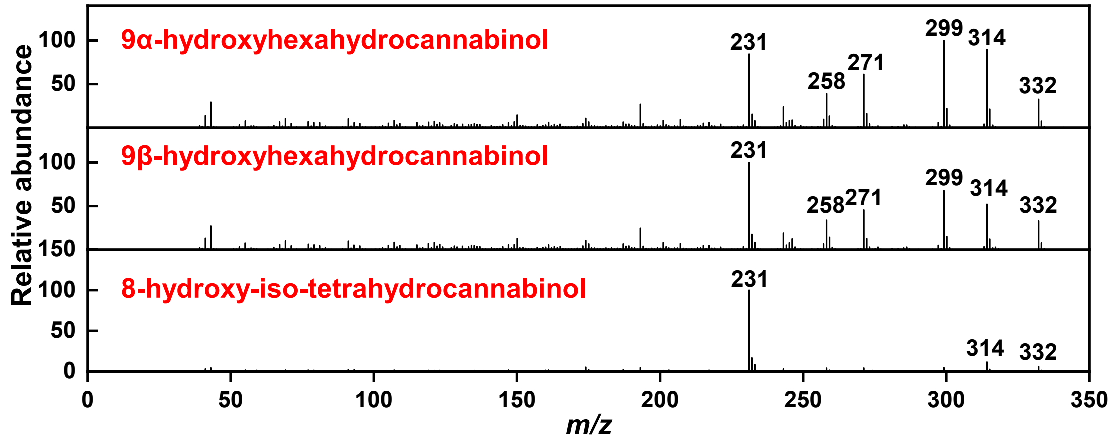

Figure S10. EI-MS spectra obtained after GC separation of 9α-hydroxyhexahydrocannabinol, 9β-hydroxyhexahydrocannabinol, and 8-hydroxy-iso-THC.

a)

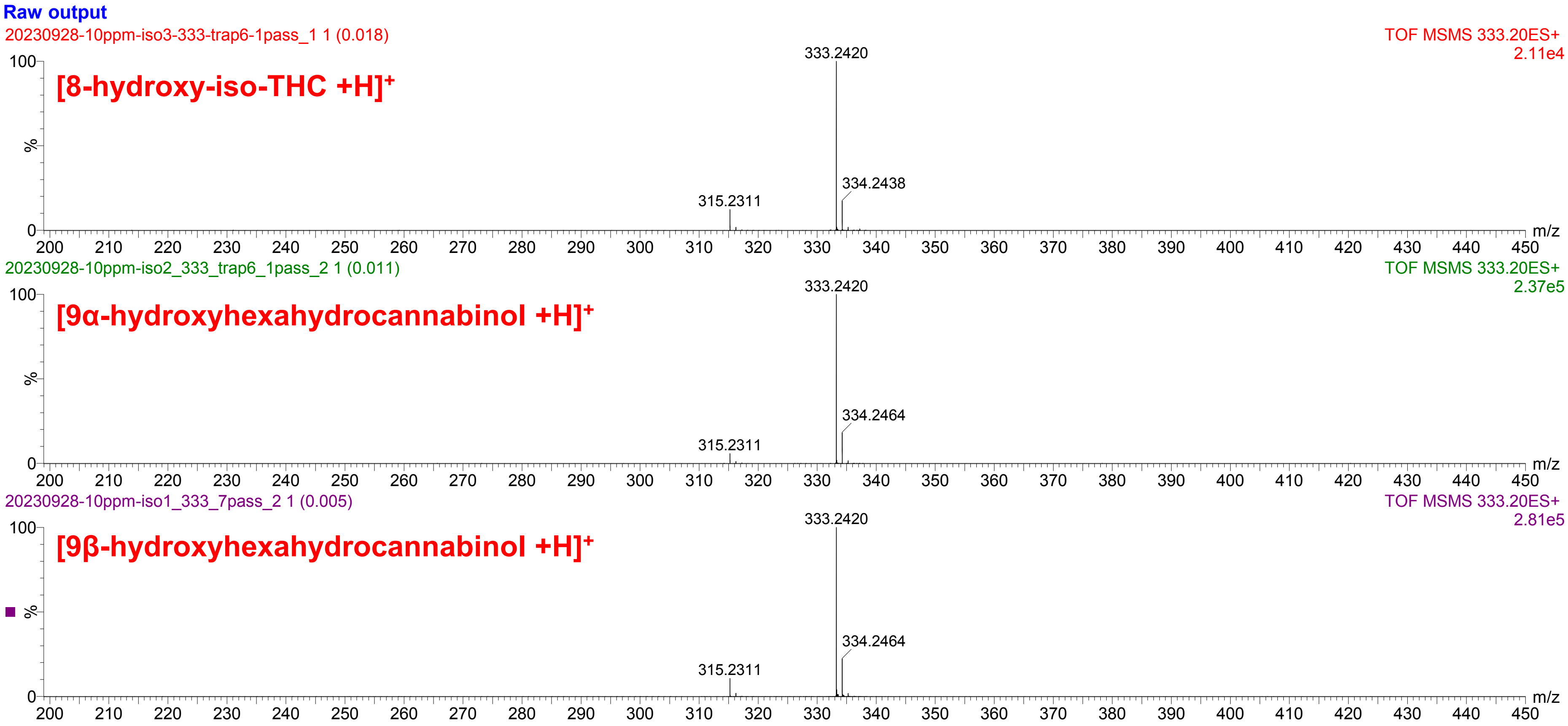
Raw output
20230928-10ppm-iso3-333-trap6-1pass_1 1 (0.018)
TOF MSMS 333.20ES+
2.11e4
[8-hydroxy-iso-THC +H]+
333.2420
334.2438
315.2311
20230928-10ppm-iso2_333_trap6_1pass_2 1 (0.011)
TOF MSMS 333.20ES+
2.37e5
[9α-hydroxyhexahydrocannabinol +H]+
333.2420
334.2464
315.2311
20230928-10ppm-iso1_333_7pass_2 1 (0.005)
TOF MSMS 333.20ES+
2.81e5
[9β-hydroxyhexahydrocannabinol +H]+
333.2420
334.2464
315.2311
%
m/z

b)

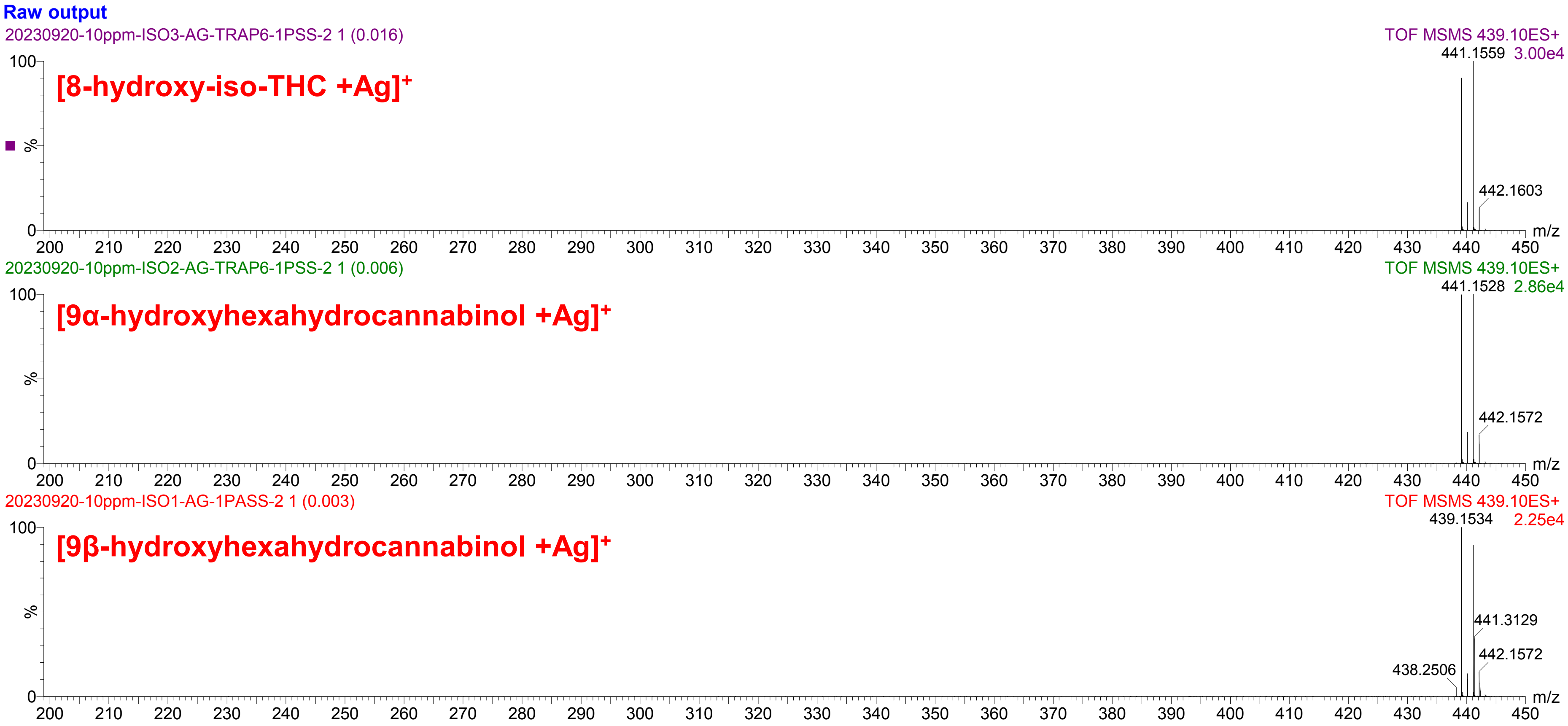

Raw output
20230920-10ppm-ISO3-AG-TRAP6-1PSS-2 1 (0.016)
TOF MSMS 439.10ES+
441.1559 3.00e4
[8-hydroxy-iso-THC +Ag]+
442.1603
20230920-10ppm-ISO2-AG-TRAP6-1PSS-2 1 (0.006)
TOF MSMS 439.10ES+
441.1528 2.86e4
[9α-hydroxyhexahydrocannabinol +Ag]+
442.1572
20230920-10ppm-ISO1-AG-1PASS-2 1 (0.003)
TOF MSMS 439.10ES+
439.1534 2.25e4
[9β-hydroxyhexahydrocannabinol +Ag]+
441.3129
442.1572
438.2506
%
m/z

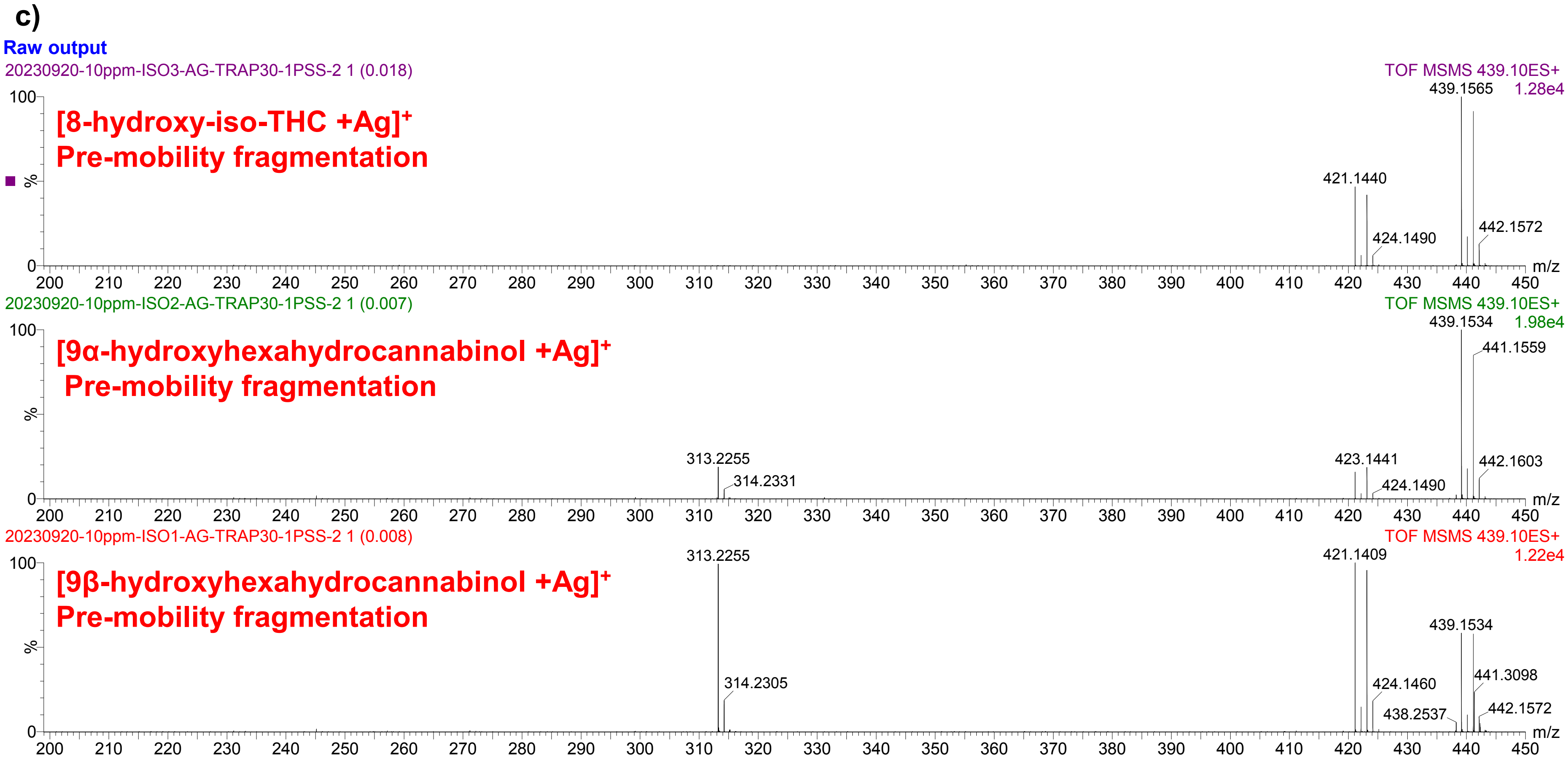

Figure S11. Mass spectra of a) protonated 8-hydroxy-iso-tetrahydrocannabinol, 9α-hydroxyhexahydrocannabinol, and 9β-hydroxyhexahydrocannabinol b) Ag(I) species of 8-hydroxy-iso-THC, 9α-hydroxyhexahydrocannabinol, and 9β-hydroxyhexahydrocannabinol, c) pre-mobility fragmentation of 8-hydroxy-iso-THC, 9α-hydroxyhexahydrocannabinol, and 9β-hydroxyhexahydrocannabinol in the presence of Ag(I) with trap energy of 30 V.

Table S4. Comparison of predicted CCS values by AllCCS, CCS values from literature and experimental CCS values obtained by cIMS of cannabinoids as protonated species and sodiated species.

Compounds	$[M+H]^+$ (*m/z* 315) (Å²)				$[M+Na]^+$ (*m/z* 337) (Å²)	
	Experimental $^{TW}CCS_{N2}$	Predicted CCS	Literature[6] $^{DT}CCS_{N2}$	Literature[7] $^{TI}CCS_{N2}$	Experimental $^{TW}CCS_{N2}$	Predicted CCS
Δ3-THC	188.4±0.01	186.5 (−1.0%)			197.9±0.03	203.5 (2.8%)
Δ8-THC	187.8±0.05	187.5 (−0.2%)			196.4±0.04	203.4 (3.6%)
Δ9-THC	187.8±0.03	187.5 (−0.2%)	187.4 (−0.2%)	183.9 (−2.1%)	194.8±0.01	203.5 (4.5%)
CBD	187.8±0.01	183.8 (−2.1%)	185.3 (−1.3%)	183.9 (−2.1%)	188.5±0.02	198.3 (5.2%)
Δ8-iso-THC	186.5±0.03	183.3 (−1.7%)			195.7±0.01	198.8 (1.6%)
Δ(4)8-iso-THC	186.6±0.04	185.1 (−0.8%)			195.8±0.01	202.3 (3.3%)
9α-hydroxyhexahydrocannabinol	191.2±0.02	190.7 (−0.3%)			201.7±0.02	205.8 (2.0%)
9β-hydroxyhexahydrocannabinol	191.6±0.2	190.6 (−0.5%)			193.0±0.01	205.6 (6.5%)
8-hydroxy-iso-THC	187.9±0.01	183.7 (−2.2%)			200.8±0.02	196.9 (−1.9%)
THCA	194.0±0.2	196.8 (1.4%)			212.2±0.01	210.8 (−0.7%)
CBDA	193.6±0.03	195.0 (0.7%)			206.7±0.01	208.3 (0.8%)
Δ8-THCV	174.9±0.01	172.5 (−1.4%)			188.0±0.02	191.5 (1.9%)
Δ8-iso-THCV	173.5±0.01	174.9 (0.8%)			183.8±0.01	194.4 (5.8%)
Δ9-THCV	174.7±0.01	175.0 (0.2%)			185.9±0.01	194.4 (4.6%)

*Values in brackets represent relative deviations from experimental CCS value

a) Δ3-THC

Raw output

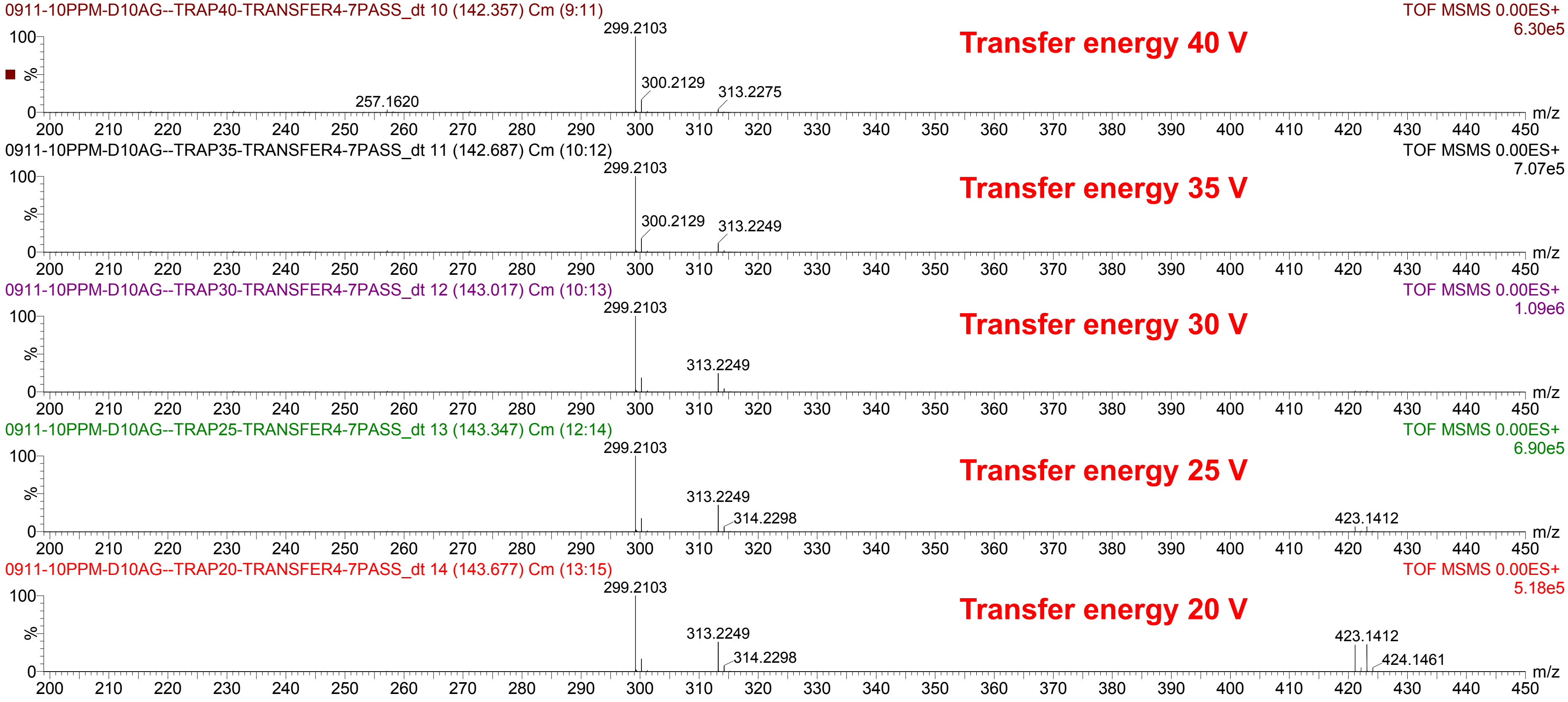

b) Δ8-THC

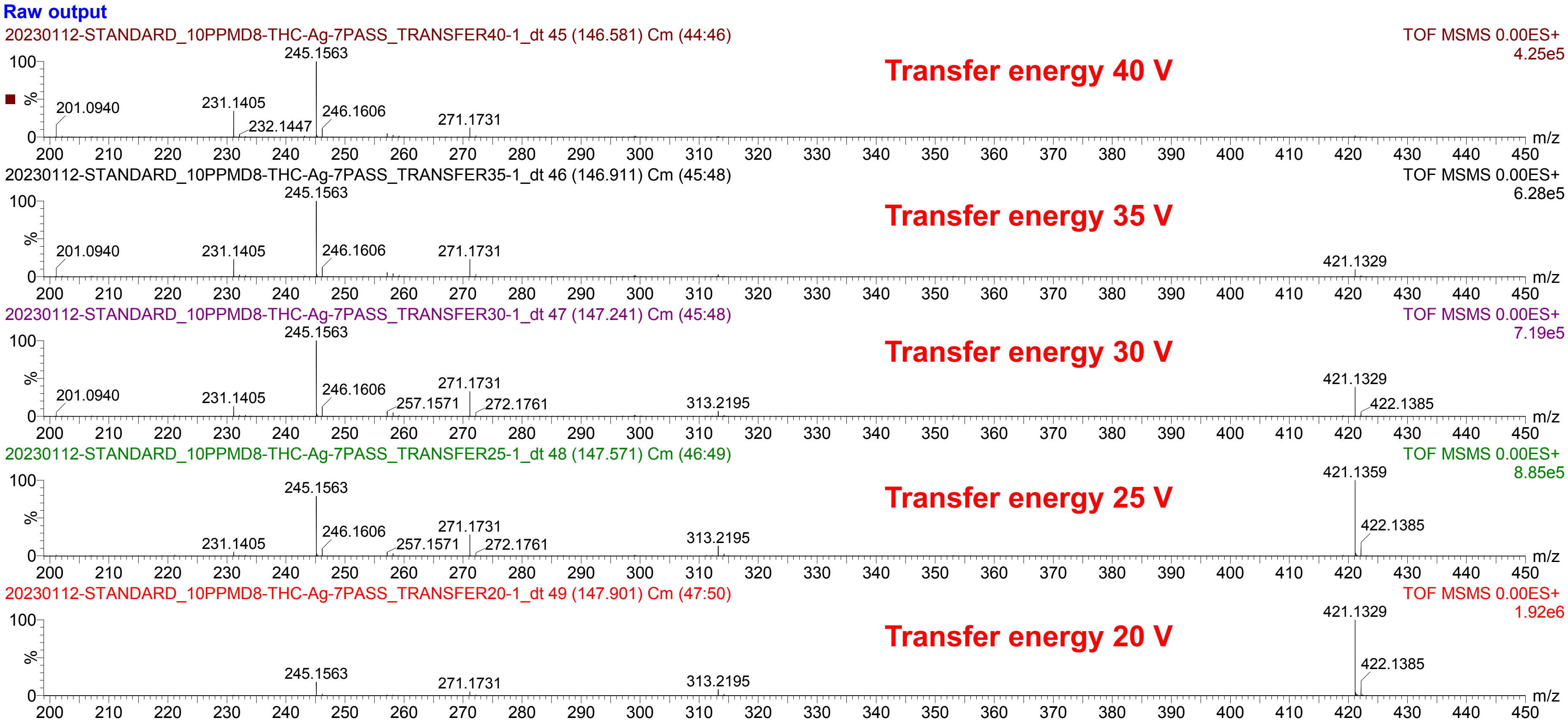

Raw output
20230112-STANDARD_10PPMD8-THC-Ag-7PASS_TRANSFER40-1_dt 45 (146.581) Cm (44:46)
TOF MSMS 0.00ES+
4.25e5
Transfer energy 40 V
245.1563
201.0940
231.1405
232.1447
246.1606
271.1731
20230112-STANDARD_10PPMD8-THC-Ag-7PASS_TRANSFER35-1_dt 46 (146.911) Cm (45:48)
TOF MSMS 0.00ES+
6.28e5
Transfer energy 35 V
245.1563
201.0940
231.1405
246.1606
271.1731
421.1329
20230112-STANDARD_10PPMD8-THC-Ag-7PASS_TRANSFER30-1_dt 47 (147.241) Cm (45:48)
TOF MSMS 0.00ES+
7.19e5
Transfer energy 30 V
245.1563
201.0940
231.1405
246.1606
257.1571
271.1731
272.1761
313.2195
421.1329
422.1385
20230112-STANDARD_10PPMD8-THC-Ag-7PASS_TRANSFER25-1_dt 48 (147.571) Cm (46:49)
TOF MSMS 0.00ES+
8.85e5
Transfer energy 25 V
245.1563
231.1405
246.1606
257.1571
271.1731
272.1761
313.2195
421.1359
422.1385
20230112-STANDARD_10PPMD8-THC-Ag-7PASS_TRANSFER20-1_dt 49 (147.901) Cm (47:50)
TOF MSMS 0.00ES+
1.92e6
Transfer energy 20 V
245.1563
271.1731
313.2195
421.1329
422.1385
%
m/z

c) Δ9-THC

Raw output

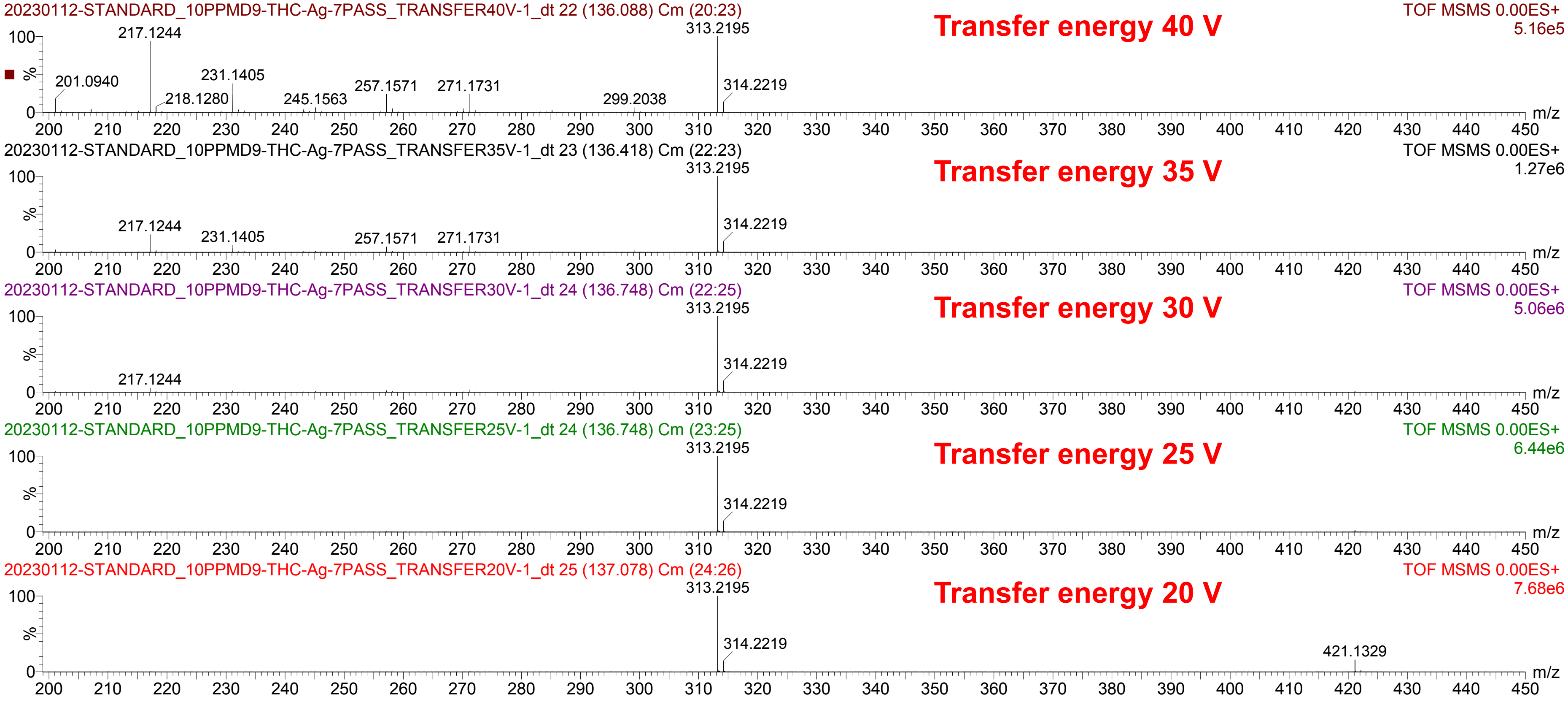

d) Δ8-iso-THC

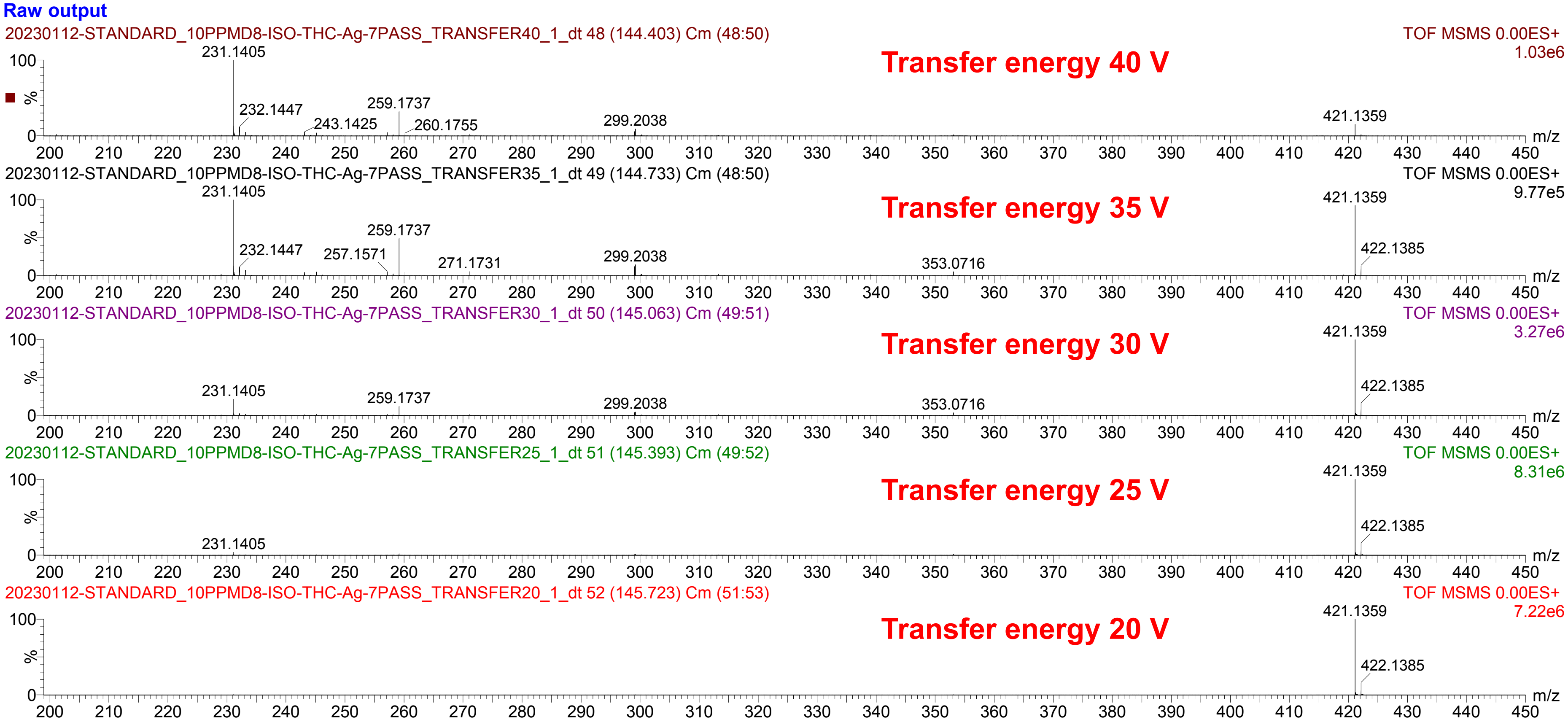

Raw output
20230112-STANDARD_10PPMD8-ISO-THC-Ag-7PASS_TRANSFER40_1_dt 48 (144.403) Cm (48:50)
TOF MSMS 0.00ES+
1.03e6
Transfer energy 40 V
231.1405
232.1447
243.1425
259.1737
260.1755
299.2038
421.1359
20230112-STANDARD_10PPMD8-ISO-THC-Ag-7PASS_TRANSFER35_1_dt 49 (144.733) Cm (48:50)
TOF MSMS 0.00ES+
9.77e5
Transfer energy 35 V
231.1405
232.1447
257.1571
259.1737
271.1731
299.2038
353.0716
421.1359
422.1385
20230112-STANDARD_10PPMD8-ISO-THC-Ag-7PASS_TRANSFER30_1_dt 50 (145.063) Cm (49:51)
TOF MSMS 0.00ES+
3.27e6
Transfer energy 30 V
231.1405
259.1737
299.2038
353.0716
421.1359
422.1385
20230112-STANDARD_10PPMD8-ISO-THC-Ag-7PASS_TRANSFER25_1_dt 51 (145.393) Cm (49:52)
TOF MSMS 0.00ES+
8.31e6
Transfer energy 25 V
231.1405
421.1359
422.1385
20230112-STANDARD_10PPMD8-ISO-THC-Ag-7PASS_TRANSFER20_1_dt 52 (145.723) Cm (51:53)
TOF MSMS 0.00ES+
7.22e6
Transfer energy 20 V
421.1359
422.1385
100
%
0
m/z
200 210 220 230 240 250 260 270 280 290 300 310 320 330 340 350 360 370 380 390 400 410 420 430 440 450

e) Δ(4)8-iso-THC

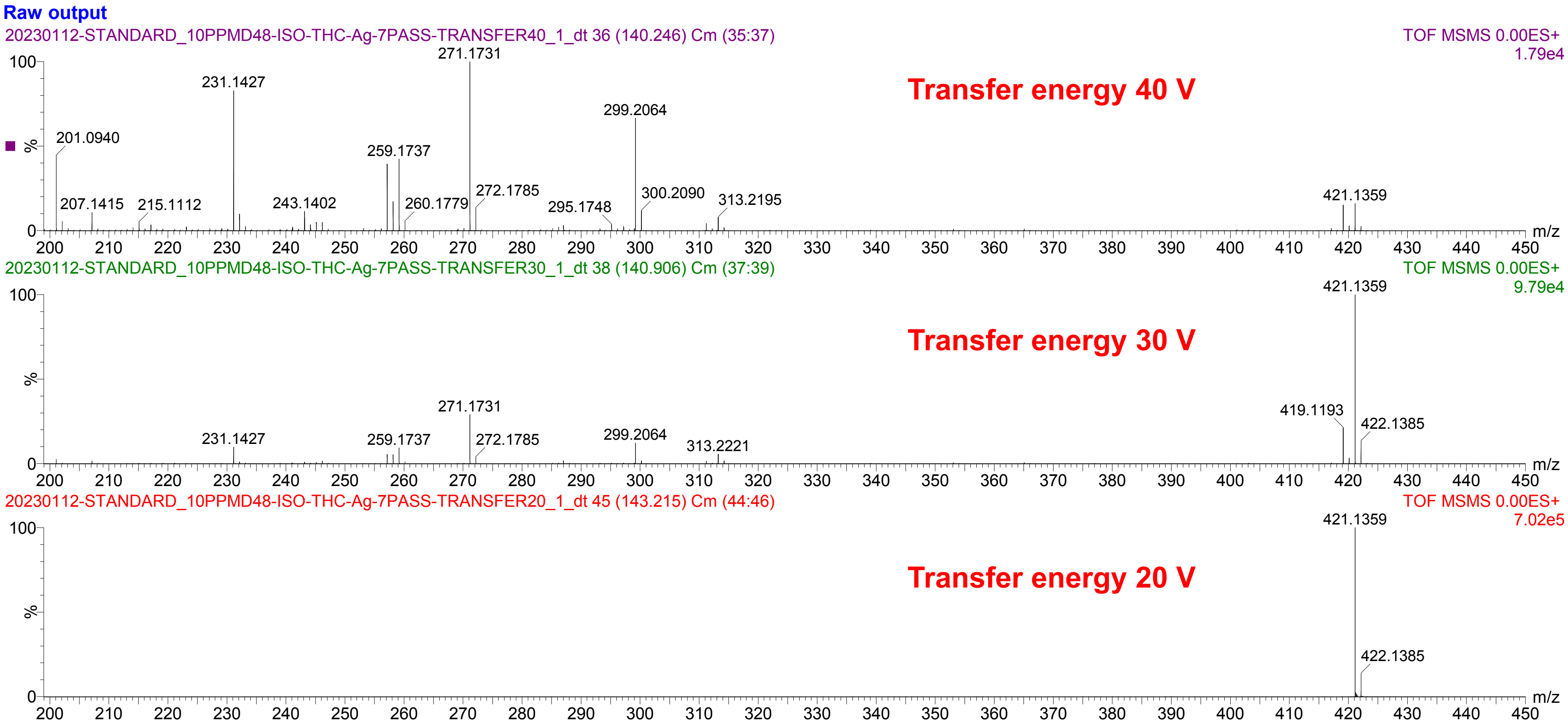

Raw output
20230112-STANDARD_10PPMD48-ISO-THC-Ag-7PASS-TRANSFER40_1_dt 36 (140.246) Cm (35:37)
TOF MSMS 0.00ES+
1.79e4
Transfer energy 40 V
271.1731
231.1427
299.2064
201.0940
259.1737
207.1415
215.1112
243.1402
260.1779
272.1785
295.1748
300.2090
313.2195
421.1359
20230112-STANDARD_10PPMD48-ISO-THC-Ag-7PASS-TRANSFER30_1_dt 38 (140.906) Cm (37:39)
TOF MSMS 0.00ES+
9.79e4
Transfer energy 30 V
421.1359
419.1193
422.1385
271.1731
231.1427
259.1737
272.1785
299.2064
313.2221
20230112-STANDARD_10PPMD48-ISO-THC-Ag-7PASS-TRANSFER20_1_dt 45 (143.215) Cm (44:46)
TOF MSMS 0.00ES+
7.02e5
Transfer energy 20 V
421.1359
422.1385
%
m/z

f) CBD

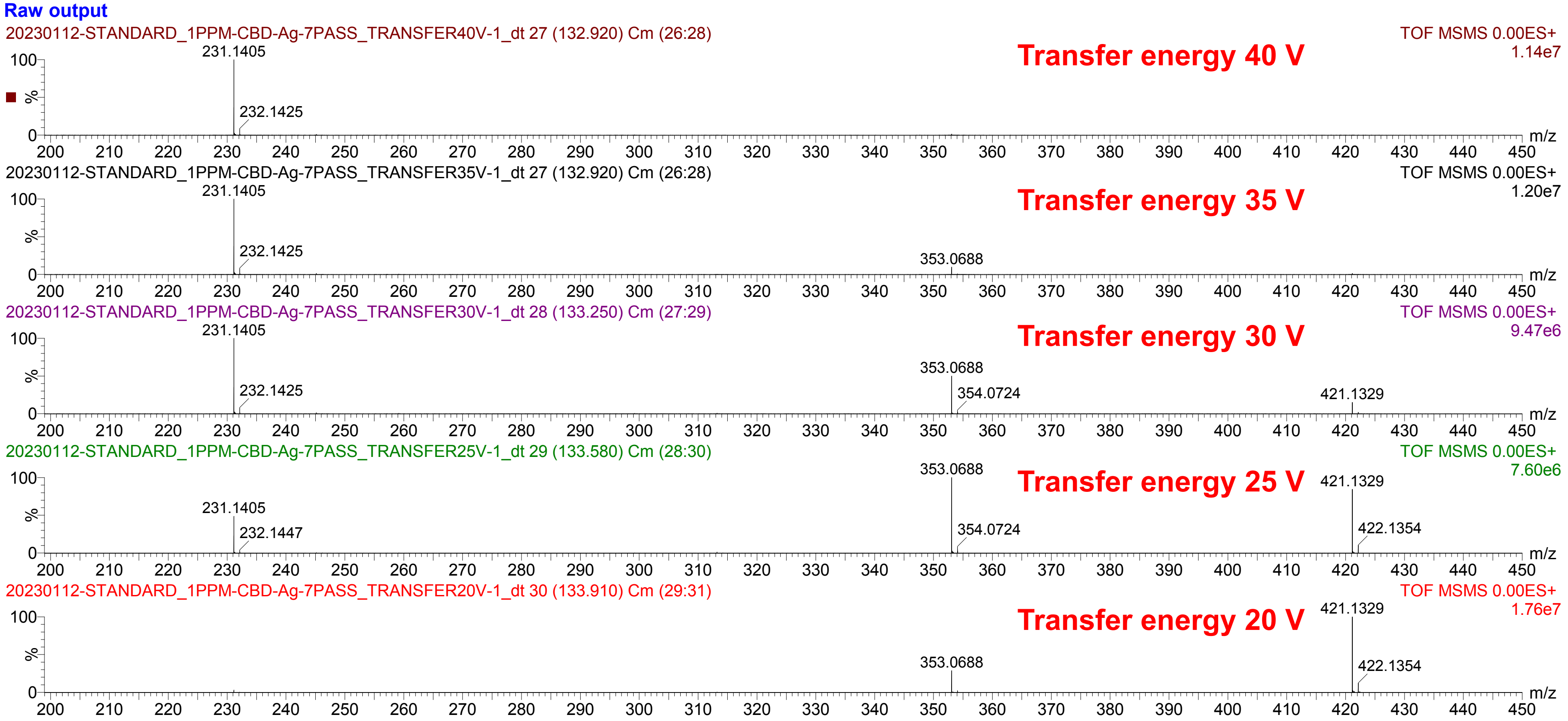

Figure S12-1. Post-mobility fragmentation of a) Δ3-THC (10 μg·mL^{-1}), b) Δ8-THC (10 μg·mL^{-1}), c) Δ9-THC (10 μg·mL^{-1}), d) Δ8-iso-THC (10 μg·mL^{-1}), e) Δ(4)8-iso-THC (10 μg·mL^{-1}), and f) CBD (1.0 μg·mL^{-1}), in the presence of Ag(I) after 7-pass separation under different transfer energies. (selecting $[M+Ag]^{+}$ precursor at *m/z* 421 for cIMS separation, followed by fragmentation).

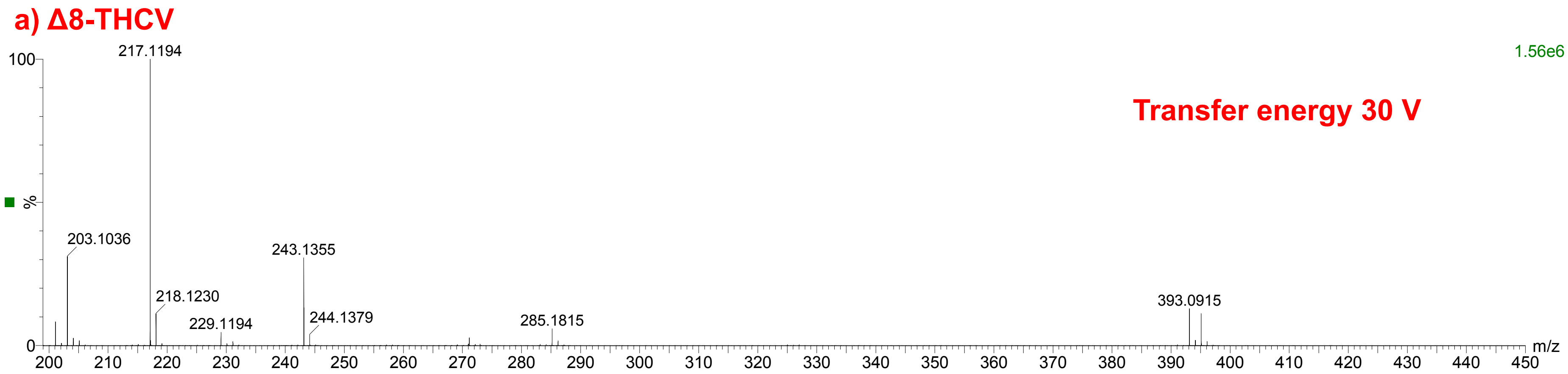

a) Δ8-THCV
Transfer energy 30 V
1.56e6
100
%
0
217.1194
203.1036
243.1355
218.1230
229.1194
244.1379
285.1815
393.0915
m/z
200 210 220 230 240 250 260 270 280 290 300 310 320 330 340 350 360 370 380 390 400 410 420 430 440 450

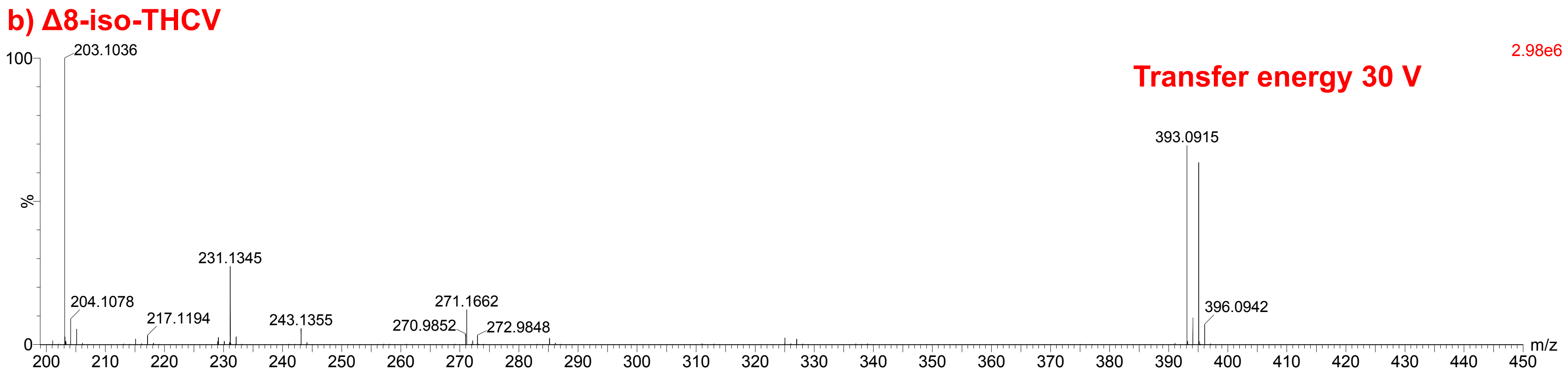

b) Δ8-iso-THCV
Transfer energy 30 V
2.98e6
100
%
0
203.1036
393.0915
231.1345
204.1078
217.1194
243.1355
270.9852
271.1662
272.9848
396.0942
m/z
200 210 220 230 240 250 260 270 280 290 300 310 320 330 340 350 360 370 380 390 400 410 420 430 440 450

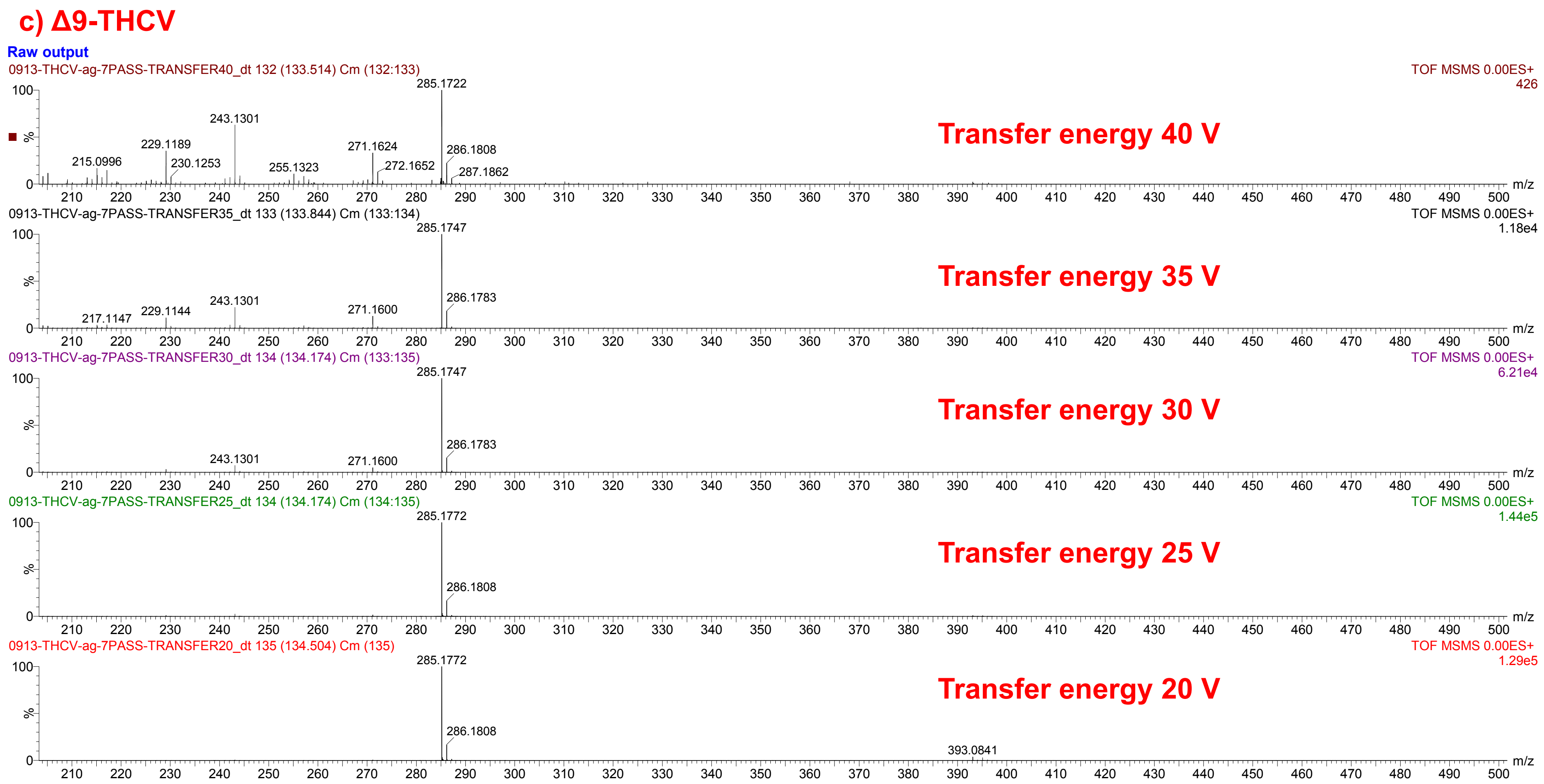

Figure S12-2. Post-mobility fragmentation of a) Δ8-THCV (1.0 μg·mL^{-1}), b) Δ8-iso-THCV (1.0 μg·mL^{-1}) and c) Δ9-THCV (10 μg·mL^{-1}) in the presence of Ag(I) after 7-pass separation under different transfer energies (selecting $[M+Ag]^+$ precursor at *m/z* 393 for cIMS separation, followed by fragmentation).

a) THCA

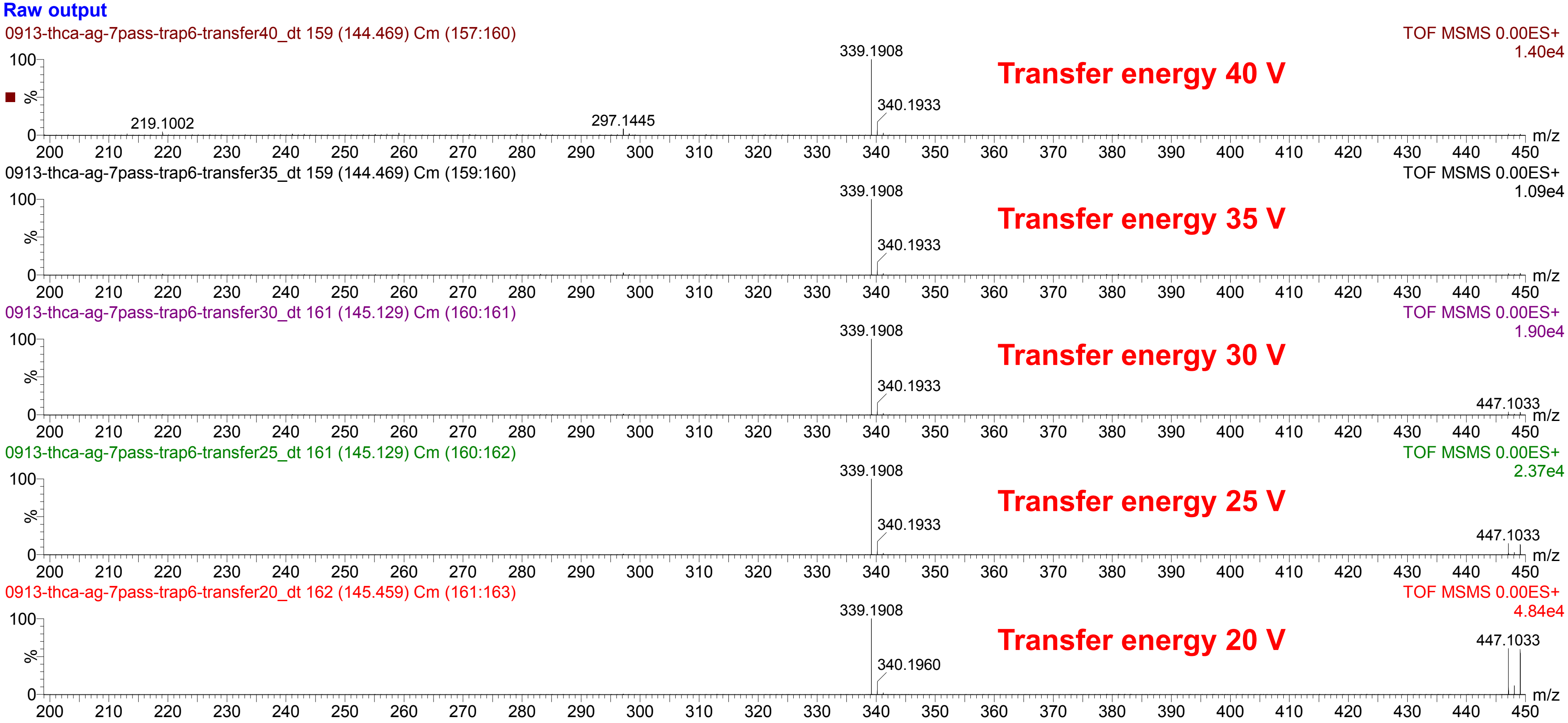
Raw output
0913-thca-ag-7pass-trap6-transfer40_dt 159 (144.469) Cm (157:160)
TOF MSMS 0.00ES+
1.40e4
Transfer energy 40 V
339.1908
340.1933
219.1002
297.1445
0913-thca-ag-7pass-trap6-transfer35_dt 159 (144.469) Cm (159:160)
TOF MSMS 0.00ES+
1.09e4
Transfer energy 35 V
339.1908
340.1933
0913-thca-ag-7pass-trap6-transfer30_dt 161 (145.129) Cm (160:161)
TOF MSMS 0.00ES+
1.90e4
Transfer energy 30 V
339.1908
340.1933
447.1033
0913-thca-ag-7pass-trap6-transfer25_dt 161 (145.129) Cm (160:162)
TOF MSMS 0.00ES+
2.37e4
Transfer energy 25 V
339.1908
340.1933
447.1033
0913-thca-ag-7pass-trap6-transfer20_dt 162 (145.459) Cm (161:163)
TOF MSMS 0.00ES+
4.84e4
Transfer energy 20 V
339.1908
340.1960
447.1033
100
%
0
m/z
200 210 220 230 240 250 260 270 280 290 300 310 320 330 340 350 360 370 380 390 400 410 420 430 440 450

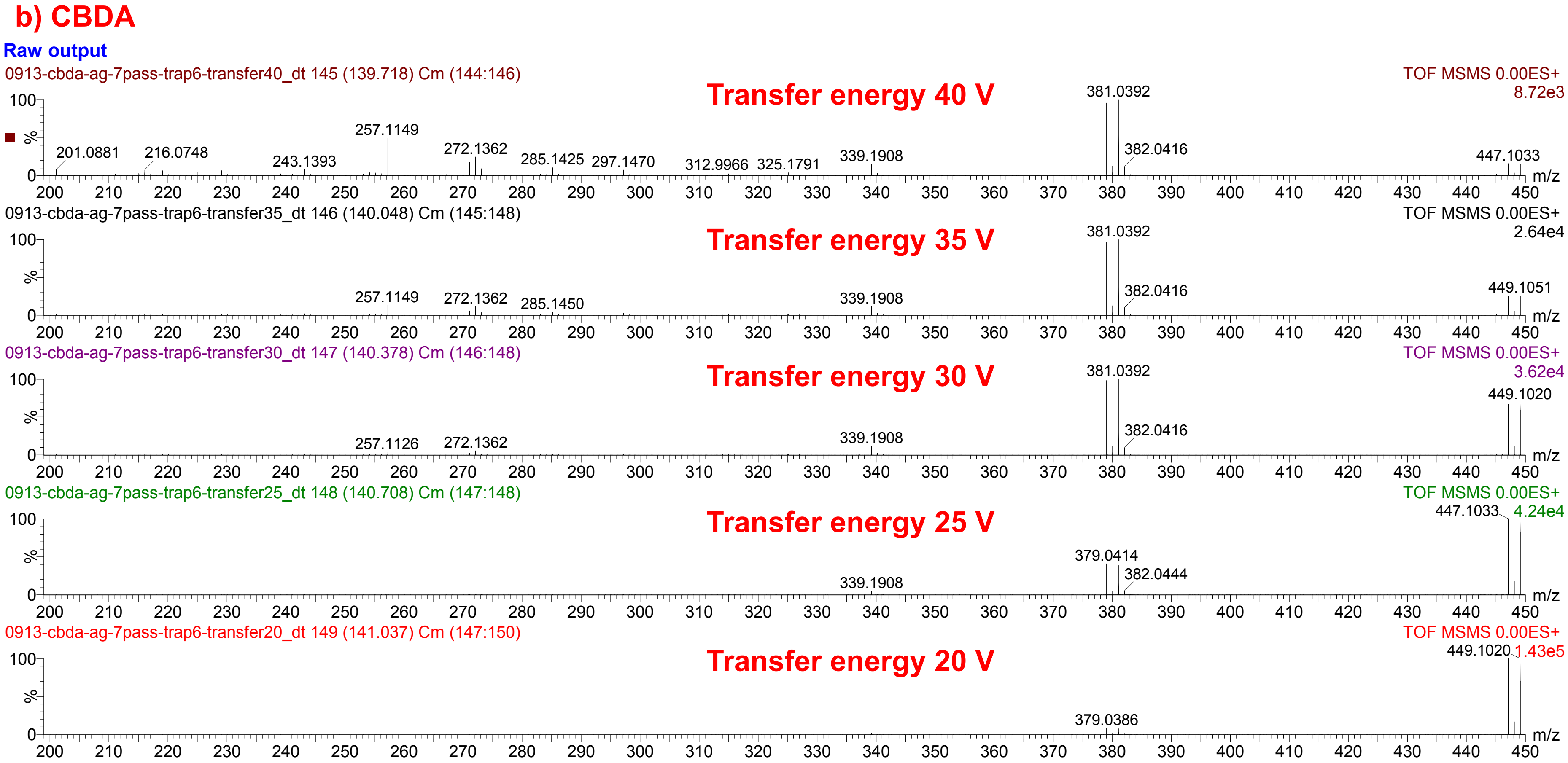

Figure S12-3. Post-mobility fragmentation of a) THCA (10 µg·mL^{-1}) and b) CBDA (10 µg·mL^{-1}) in the presence of Ag(I) after 7-pass separation under different transfer energies (selecting $[M+Ag]^+$ precursor at *m/z* 465 for cIMS separation, followed by fragmentation).

a) 9β-hydroxyhexahydrocannabinol

Raw output

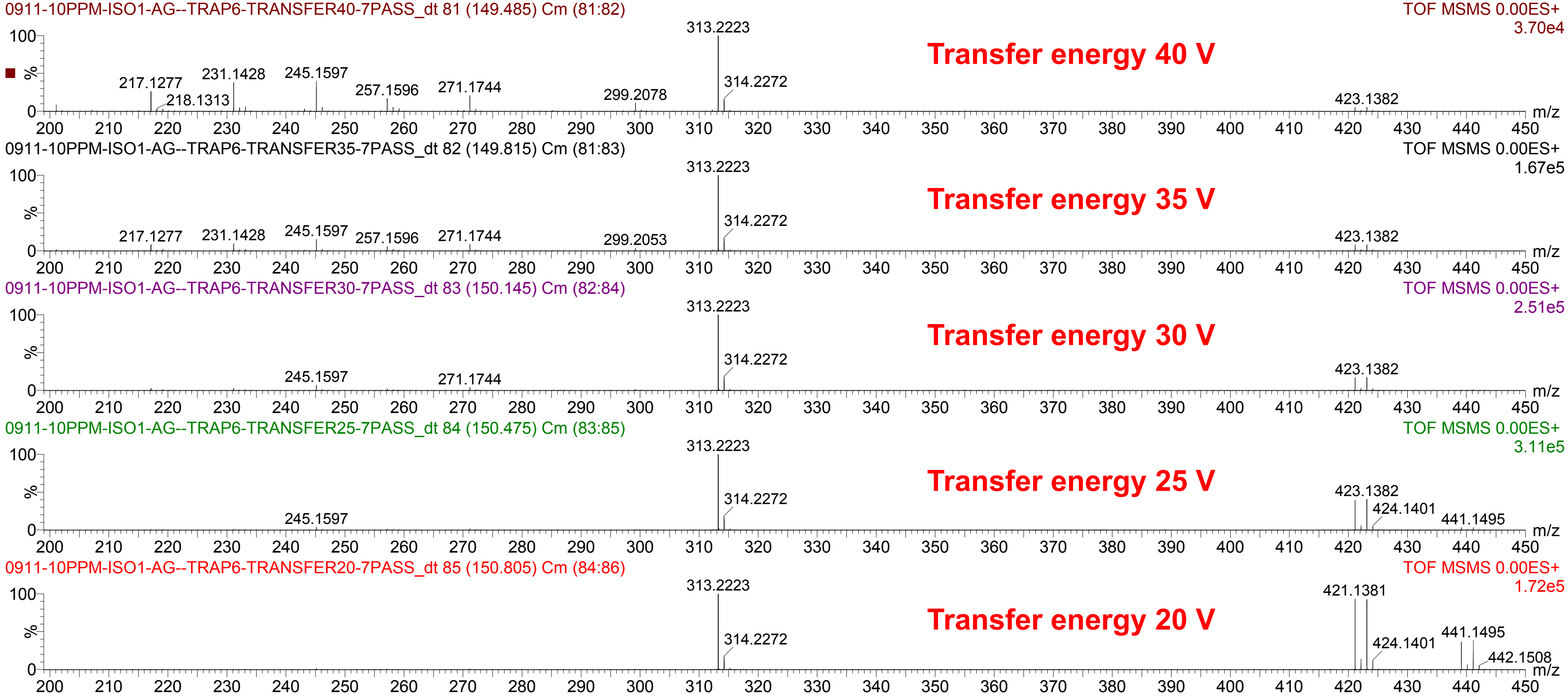

b) 9α-hydroxyhexahydrocannabinol

Raw output

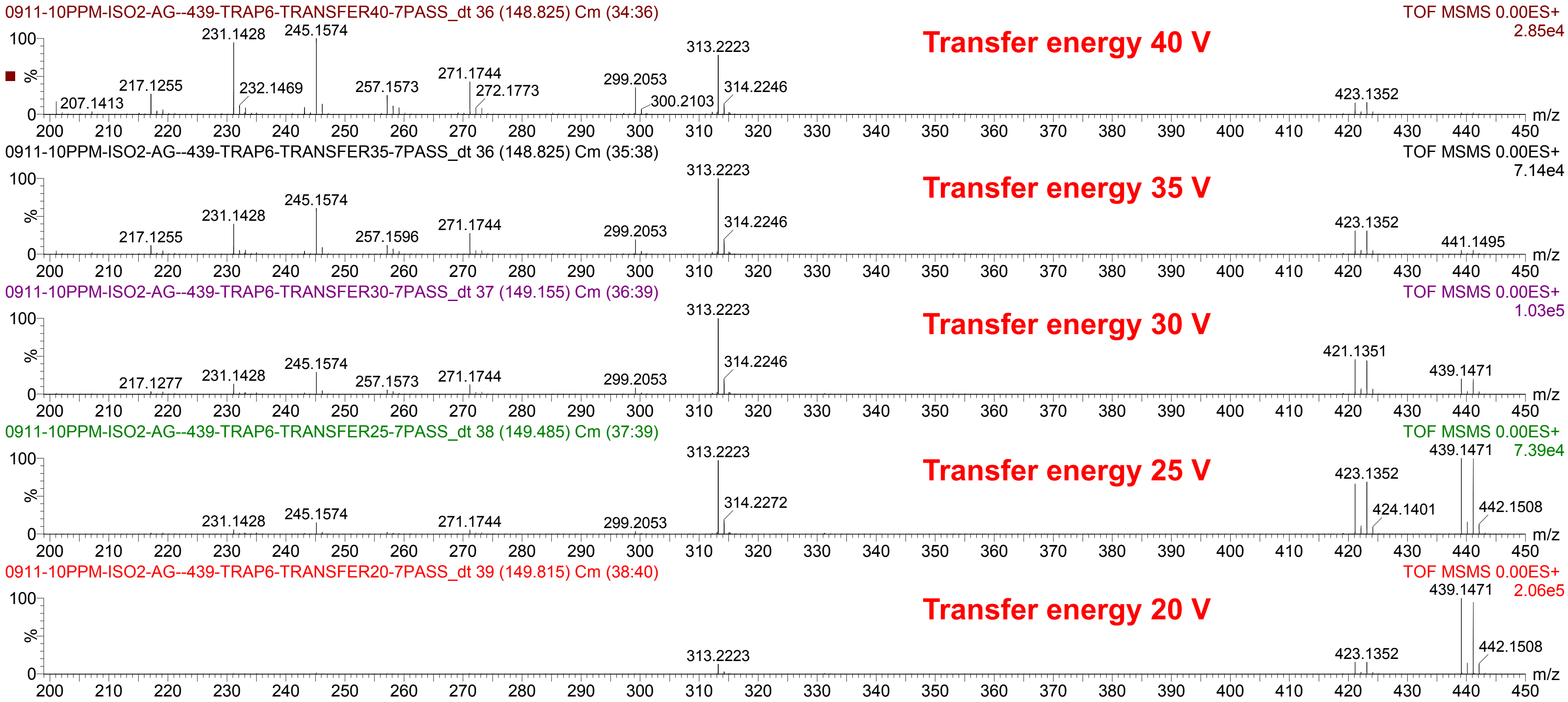

c) 8-hydroxy-iso-THC

Raw output

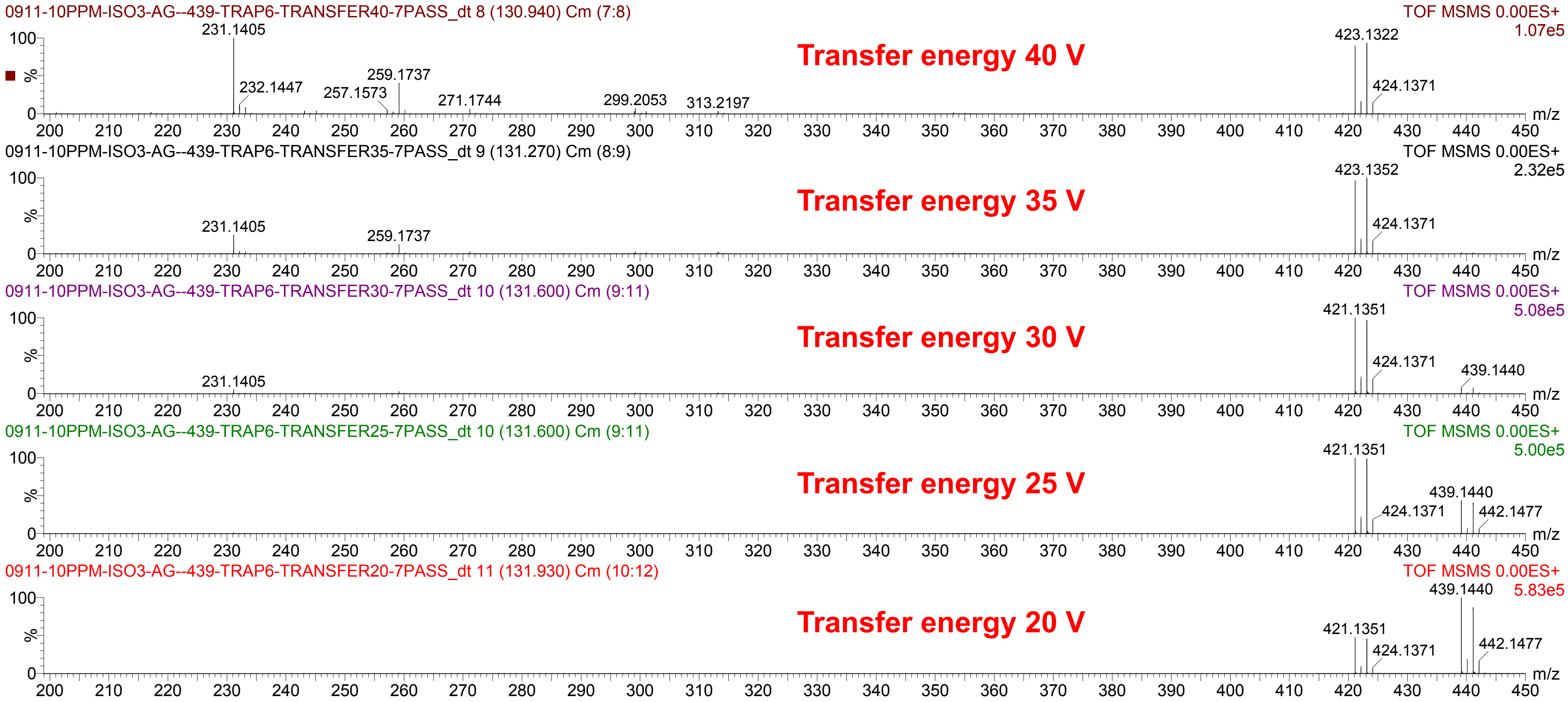

Figure S12-4. Post-mobility fragmentation of a) 9β-hydroxyhexahydrocannabinol (10 μg·mL^{-1}), b) 9α-hydroxyhexahydrocannabinol (10 μg·mL^{-1}) and c) 8-hydroxy-iso-tetrahydrocannabinol (10 μg·mL^{-1}) in the presence of Ag(I) after 7-pass separation under different transfer energies (selecting [M+Ag]$^+$ precursor at *m/z* 439 for cIMS separation, followed by fragmentation).

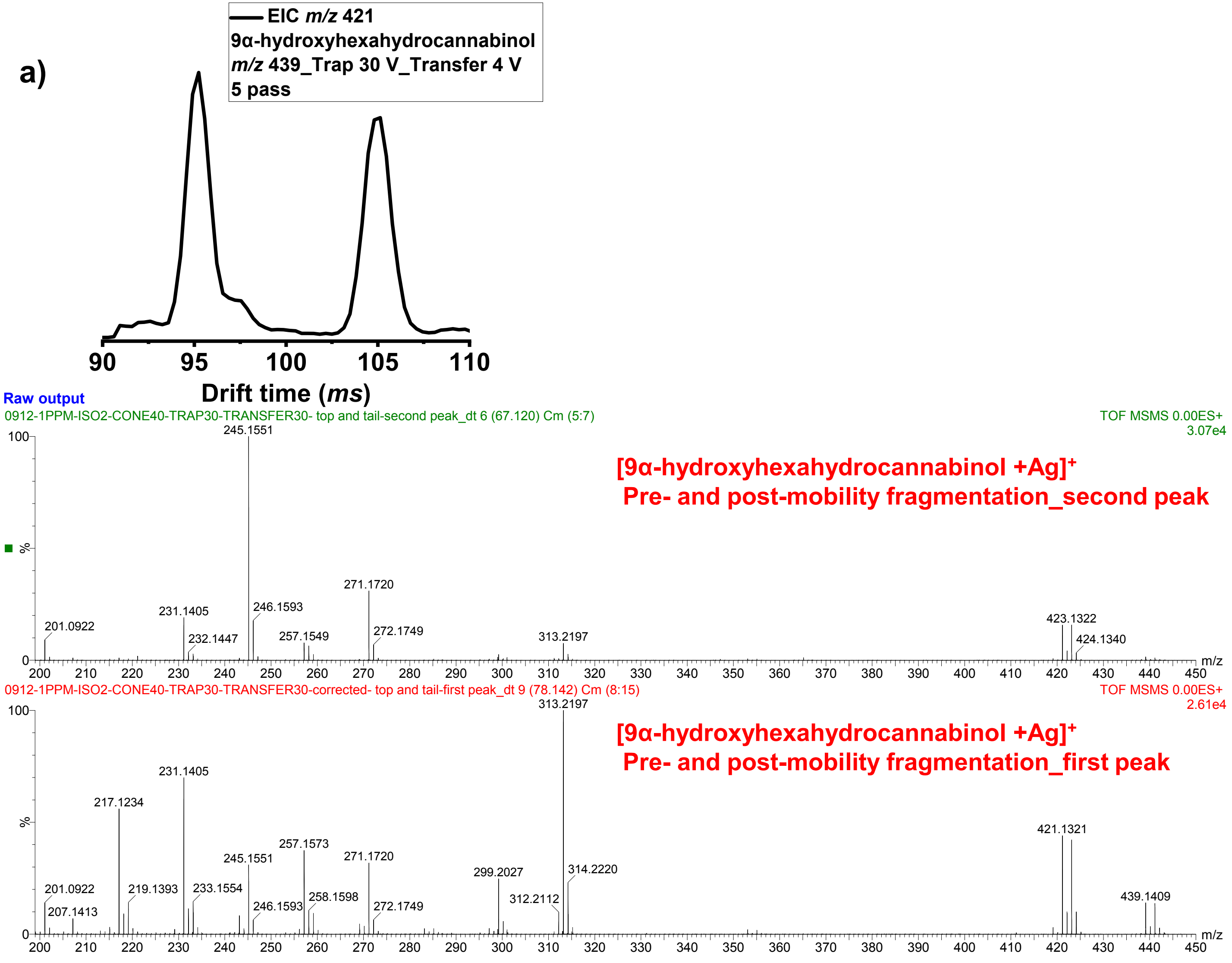
a)
EIC m/z 421
9α-hydroxyhexahydrocannabinol
m/z 439_Trap 30 V_Transfer 4 V
5 pass
90
95
100
105
110
Drift time (ms)
Raw output
0912-1PPM-ISO2-CONE40-TRAP30-TRANSFER30- top and tail-second peak_dt 6 (67.120) Cm (5:7)
TOF MSMS 0.00ES+
3.07e4
[9α-hydroxyhexahydrocannabinol +Ag]+
Pre- and post-mobility fragmentation_second peak
245.1551
271.1720
231.1405
246.1593
201.0922
232.1447
257.1549
272.1749
313.2197
423.1322
424.1340
0912-1PPM-ISO2-CONE40-TRAP30-TRANSFER30-corrected- top and tail-first peak_dt 9 (78.142) Cm (8:15)
TOF MSMS 0.00ES+
2.61e4
[9α-hydroxyhexahydrocannabinol +Ag]+
Pre- and post-mobility fragmentation_first peak
313.2197
231.1405
217.1234
421.1321
257.1573
245.1551
271.1720
299.2027
314.2220
201.0922
219.1393
233.1554
258.1598
439.1409
312.2112
207.1413
246.1593
272.1749
%
100
0
m/z
200
210
220
230
240
250
260
270
280
290
300
310
320
330
340
350
360
370
380
390
400
410
420
430
440
450

b)

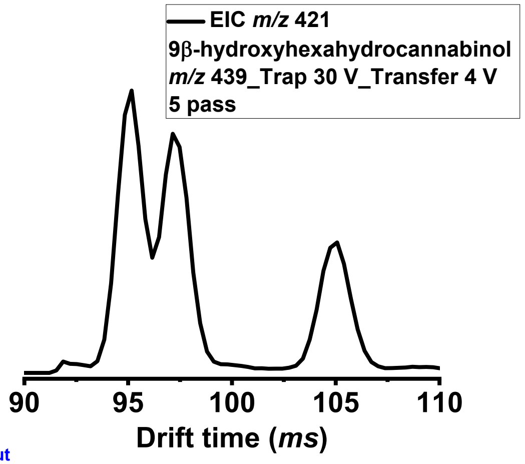

EIC m/z 421
9β-hydroxyhexahydrocannabinol
m/z 439_Trap 30 V_Transfer 4 V
5 pass
90
95
100
105
110
Drift time (ms)

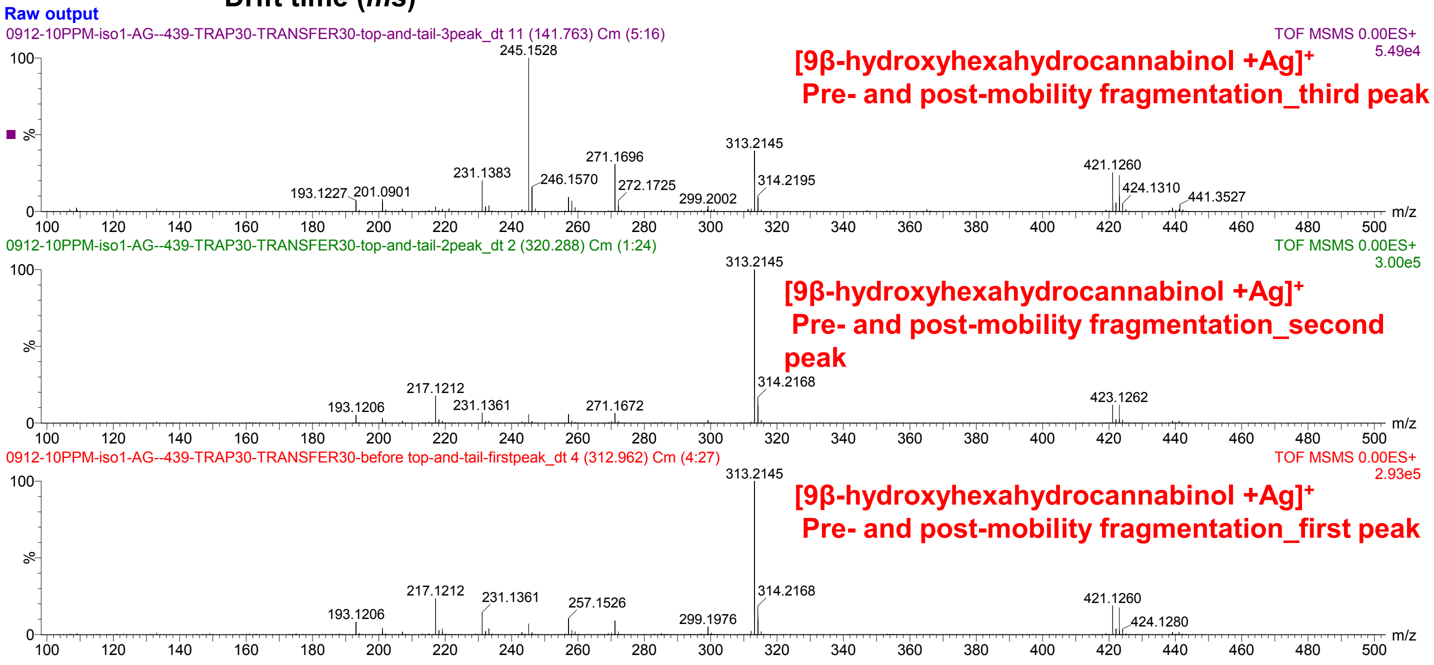

Raw output
0912-10PPM-iso1-AG--439-TRAP30-TRANSFER30-top-and-tail-3peak_dt 11 (141.763) Cm (5:16)
TOF MSMS 0.00ES+
5.49e4
[9β-hydroxyhexahydrocannabinol +Ag]+
Pre- and post-mobility fragmentation_third peak
245.1528
313.2145
271.1696
231.1383
246.1570
272.1725
193.1227
201.0901
299.2002
314.2195
421.1260
424.1310
441.3527
0912-10PPM-iso1-AG--439-TRAP30-TRANSFER30-top-and-tail-2peak_dt 2 (320.288) Cm (1:24)
TOF MSMS 0.00ES+
3.00e5
[9β-hydroxyhexahydrocannabinol +Ag]+
Pre- and post-mobility fragmentation_second peak
313.2145
314.2168
217.1212
193.1206
231.1361
271.1672
423.1262
0912-10PPM-iso1-AG--439-TRAP30-TRANSFER30-before top-and-tail-firstpeak_dt 4 (312.962) Cm (4:27)
TOF MSMS 0.00ES+
2.93e5
[9β-hydroxyhexahydrocannabinol +Ag]+
Pre- and post-mobility fragmentation_first peak
313.2145
314.2168
217.1212
231.1361
257.1526
193.1206
299.1976
421.1260
424.1280
%
m/z

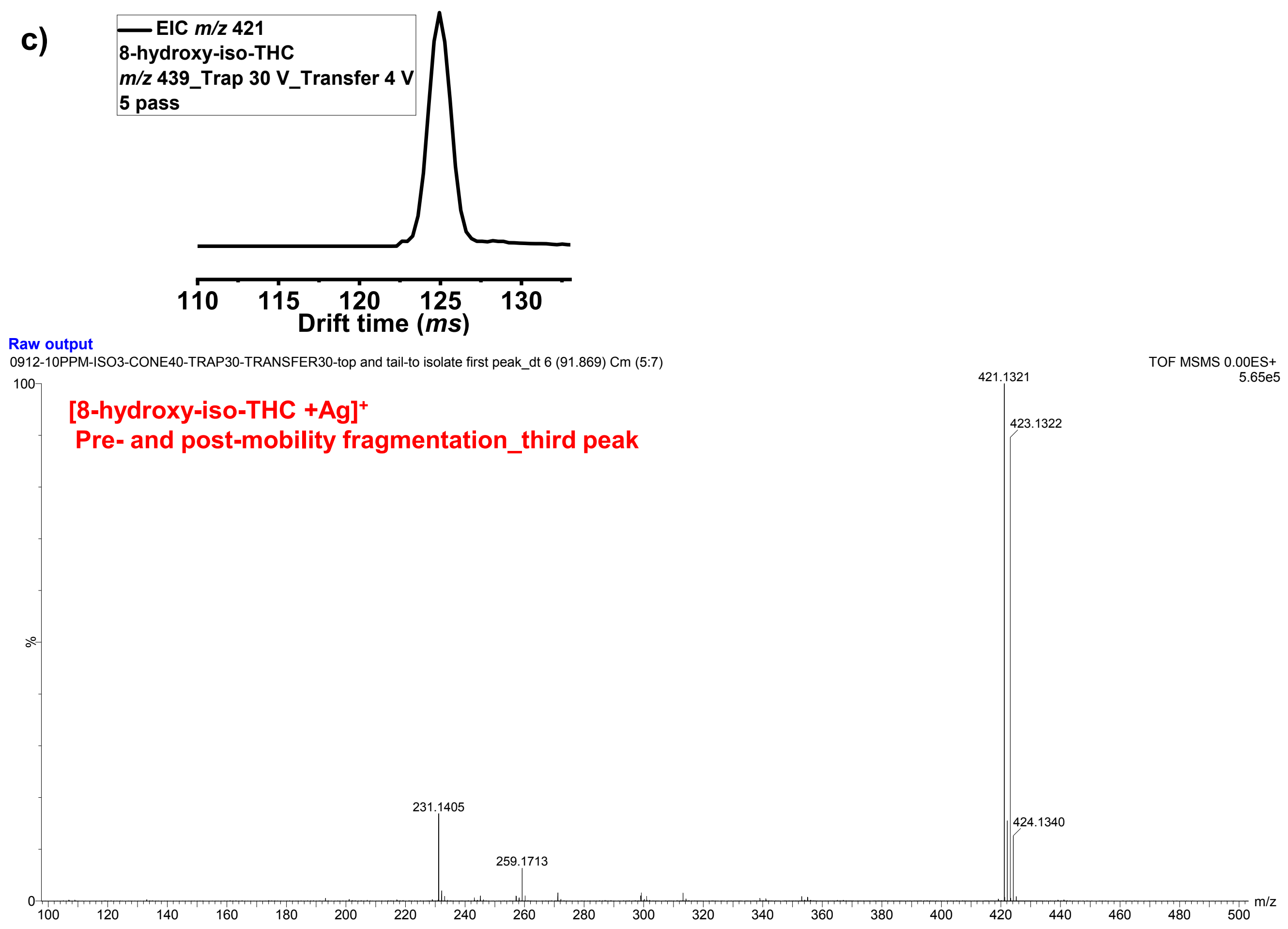

Figure S13. Pre- and post-mobility fragmentation of a) 9α-hydroxyhexahydrocannabinol, b) 9β-hydroxyhexahydrocannabinol, and c) 8-hydroxy-iso-THC in the presence of Ag(I) with trap energy of 30 V and transfer energy of 30 V.

Table S5. CCS of 9α-hydroxyhexahydrocannabinol, 9β-hydroxyhexahydrocannabinol, and 8-hydroxy-iso-THC as well as their dehydrated species in the presence of Ag(I).

	**$[M+Ag]^+$ $^{TW}CCS_{N2}$(Å^2)**
9α-hydroxyhexahydrocannabinol	195.6±0.01
Dehydrated 9α-hydroxyhexahydrocannabinol	182.0±0.01<br>190.6±0.02<br>(−0.2% to Δ8-THC)
9β-hydroxyhexahydrocannabinol	185.9±0.01
Dehydrated 9β-hydroxyhexahydrocannabinol	181.9±0.01<br>183.9±0.01<br>190.6±0.01<br>(−0.2% to Δ8-THC)
8-hydroxy-iso-THC	184.6±0.02
Dehydrated 8-hydroxy-iso-THC	179.7±0.01

Table S6. Intra-day, inter-day, and inter-pass relative standard deviation of $[\Delta8\text{-THC+Ag}]^+$ CCS.

Measured date and separation passes	$[\Delta8\text{-THC+Ag}]^+$ eCCS	Intra-day RSD (%)	Inter-day RSD (%)	Inter-pass RSD (%)
	190.7			
11 th November 2022_7 passes	190.7	0		
	190.7			
	190.9			
12 th January 2023_7 passes	190.9	0.03		
	190.8			
			0.04	
	190.7			
20 th September 2023_7 passes	190.7	0		
	190.7			
				0.3
	190.7			
28 th September 2023_7 passes	190.7	0		
	190.7			
	190.3			
12 th January 2023_1 pass	190.3	0.03		
	190.2			
			0.2	
	189.3			
20 th September 2023_1 pass	189.6	0.1		
	189.7			

C#1 _full scan_for checking Ag(I) adducts

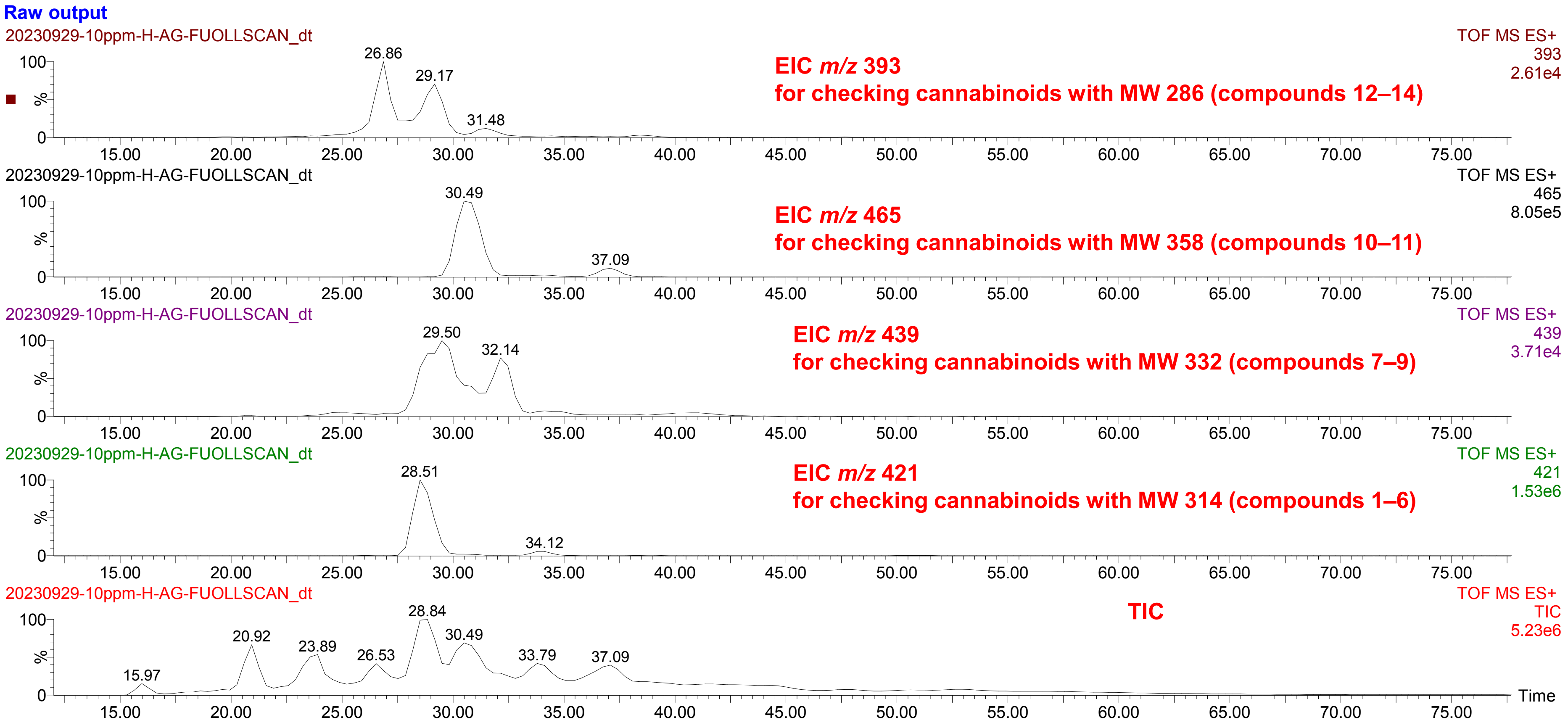

C#1 _SIM_for checking Ag(I) adducts

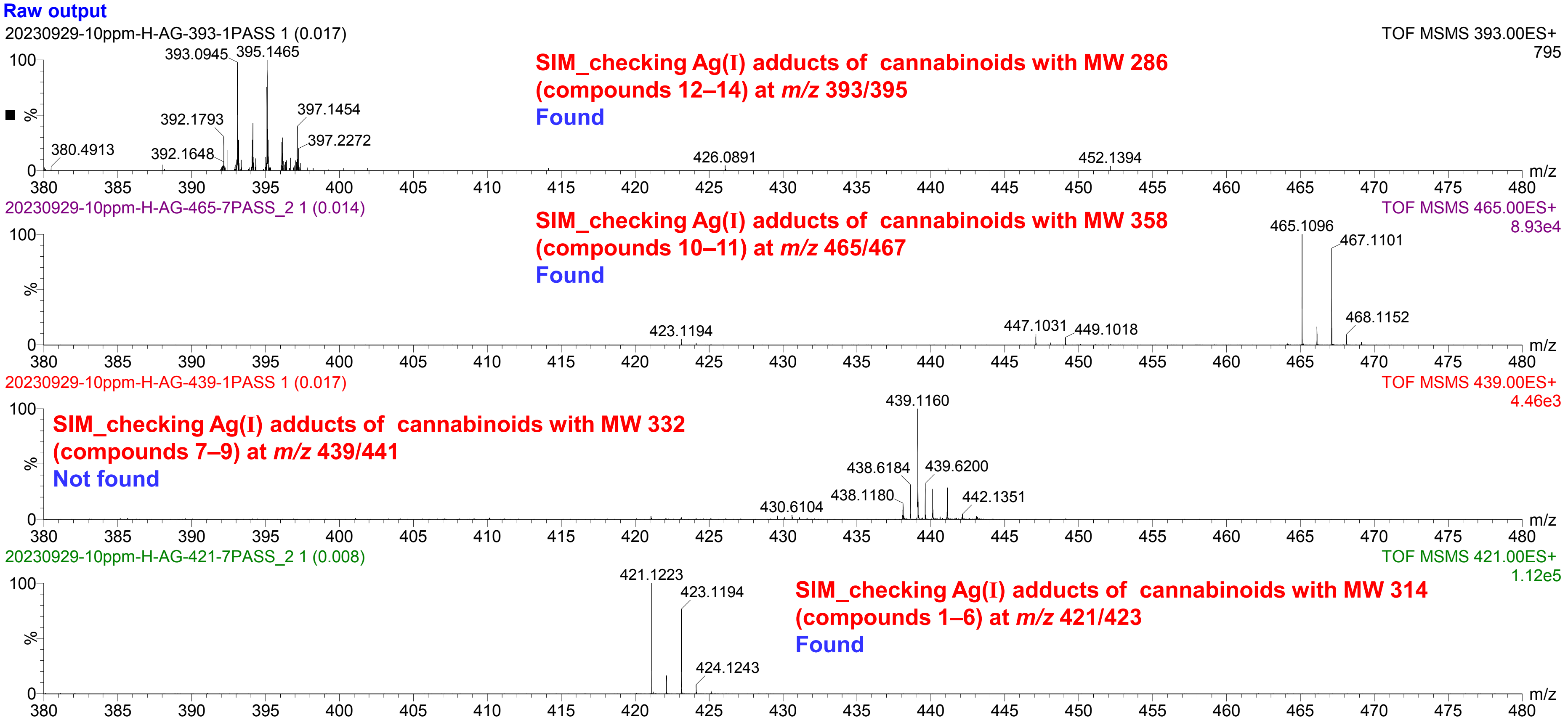

C#1 _mobility separation_for checking CCS

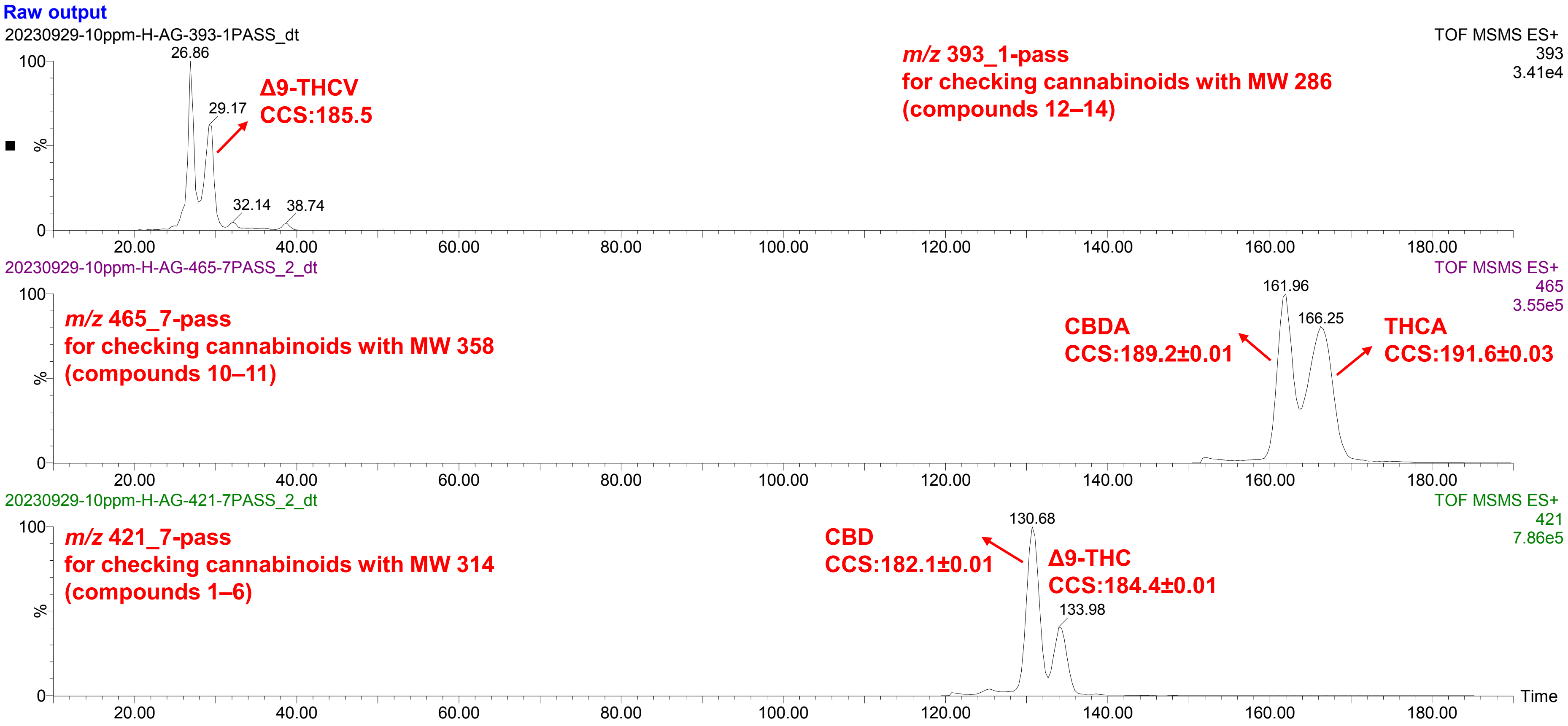

C#1_mobility separation+transfer fragmentation_for checking fragments

Raw output

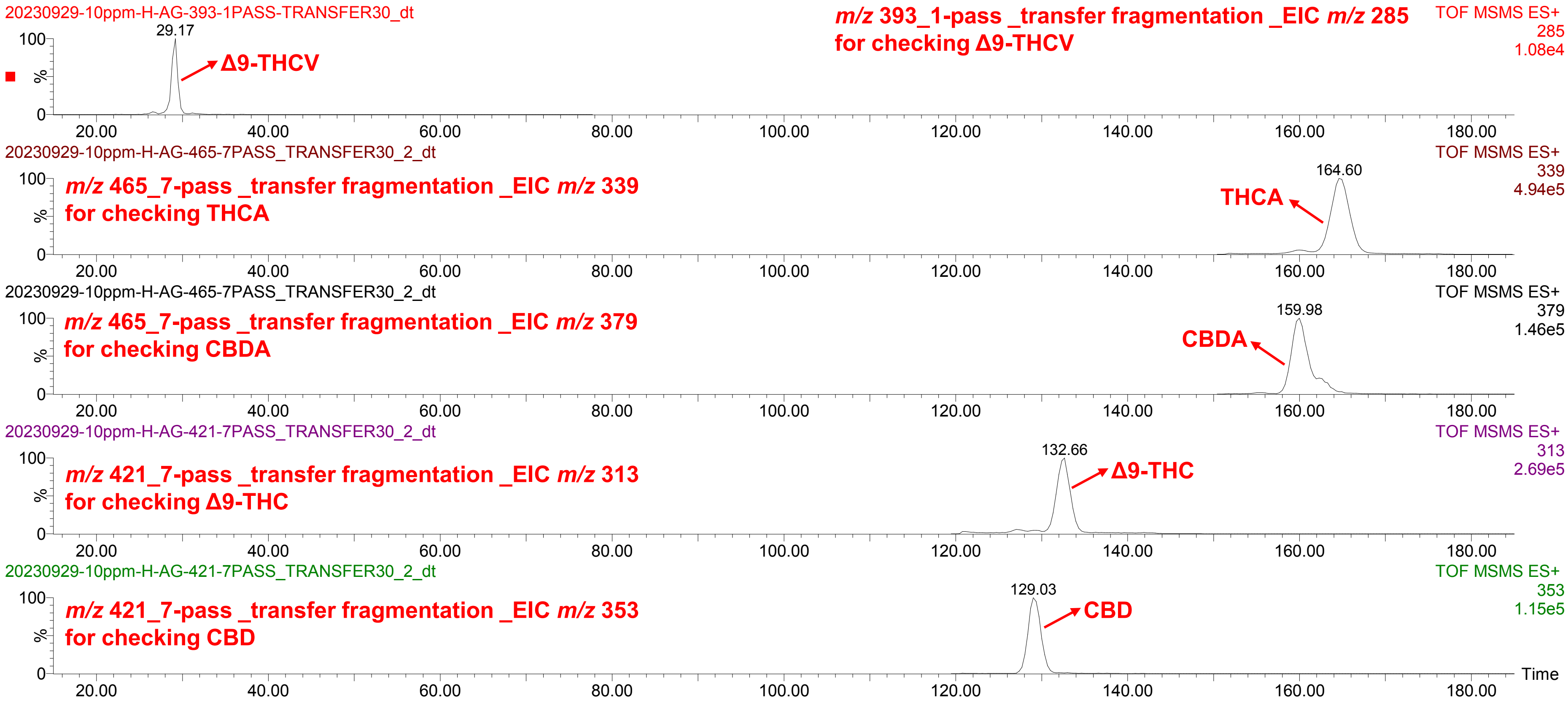

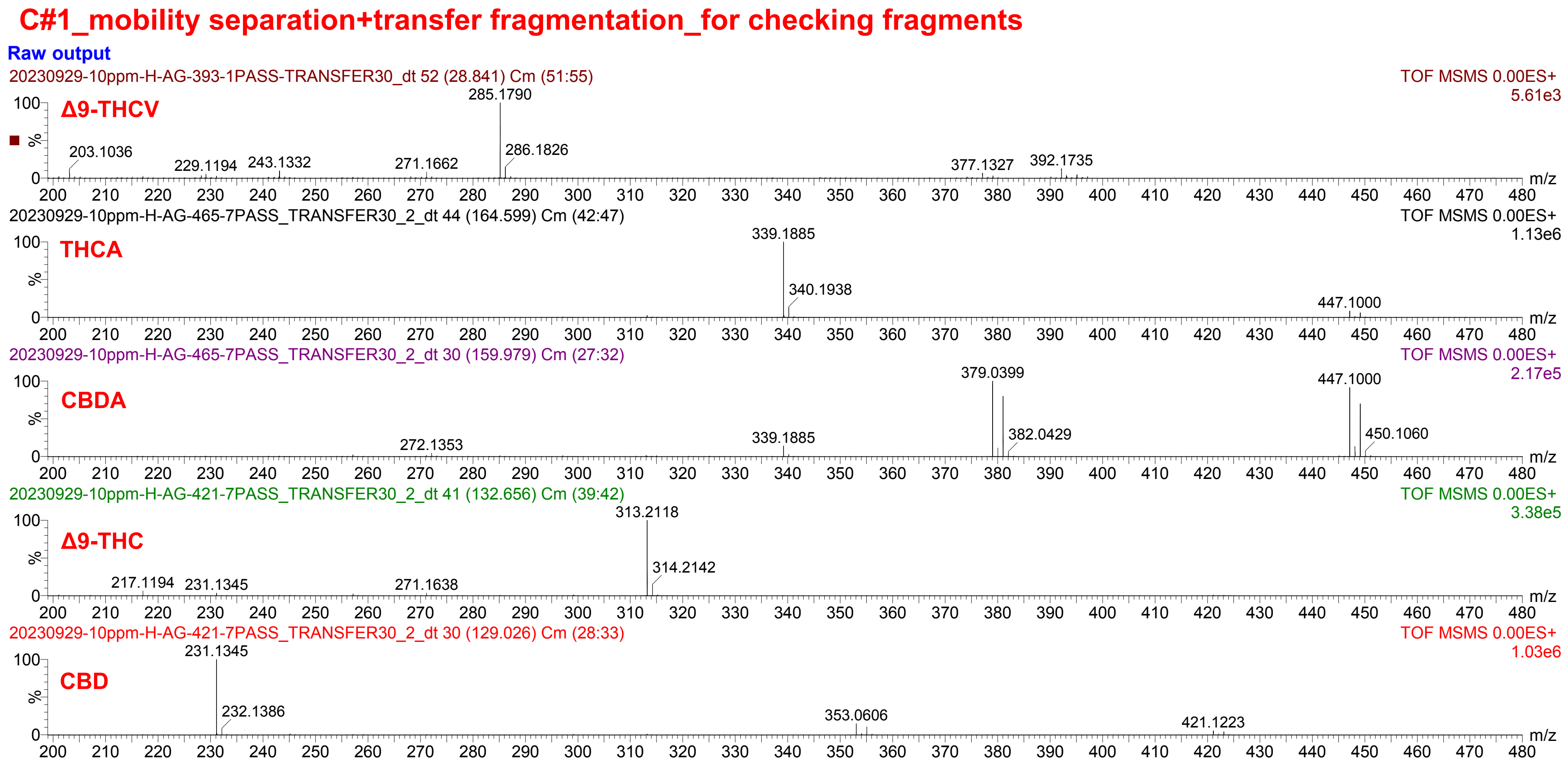

Figure S14-1. Mobiligram and mass spectra of cannabinoids in sample C#1.

C#2 _full scan_for checking Ag(I) adducts

Raw output

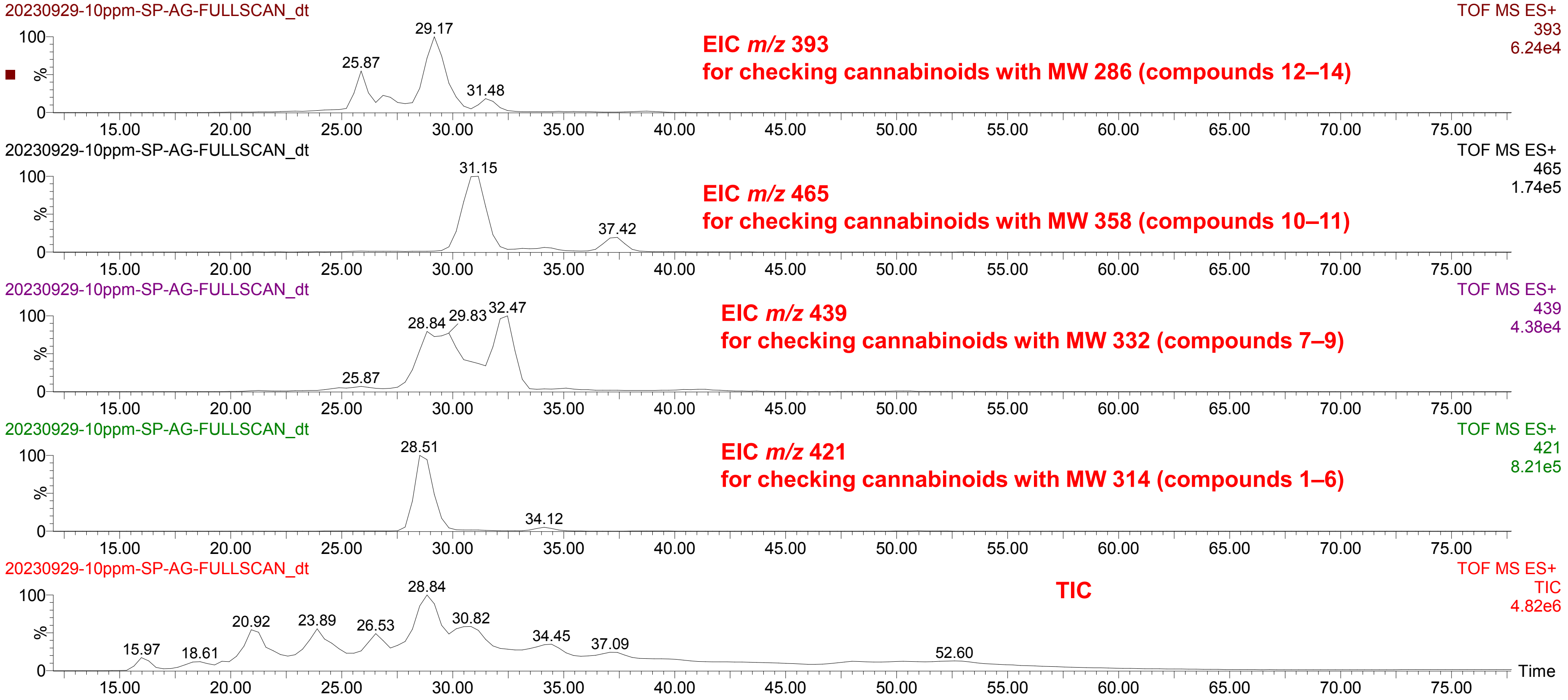

C#2 _SIM_for checking Ag(I) adducts

Raw output

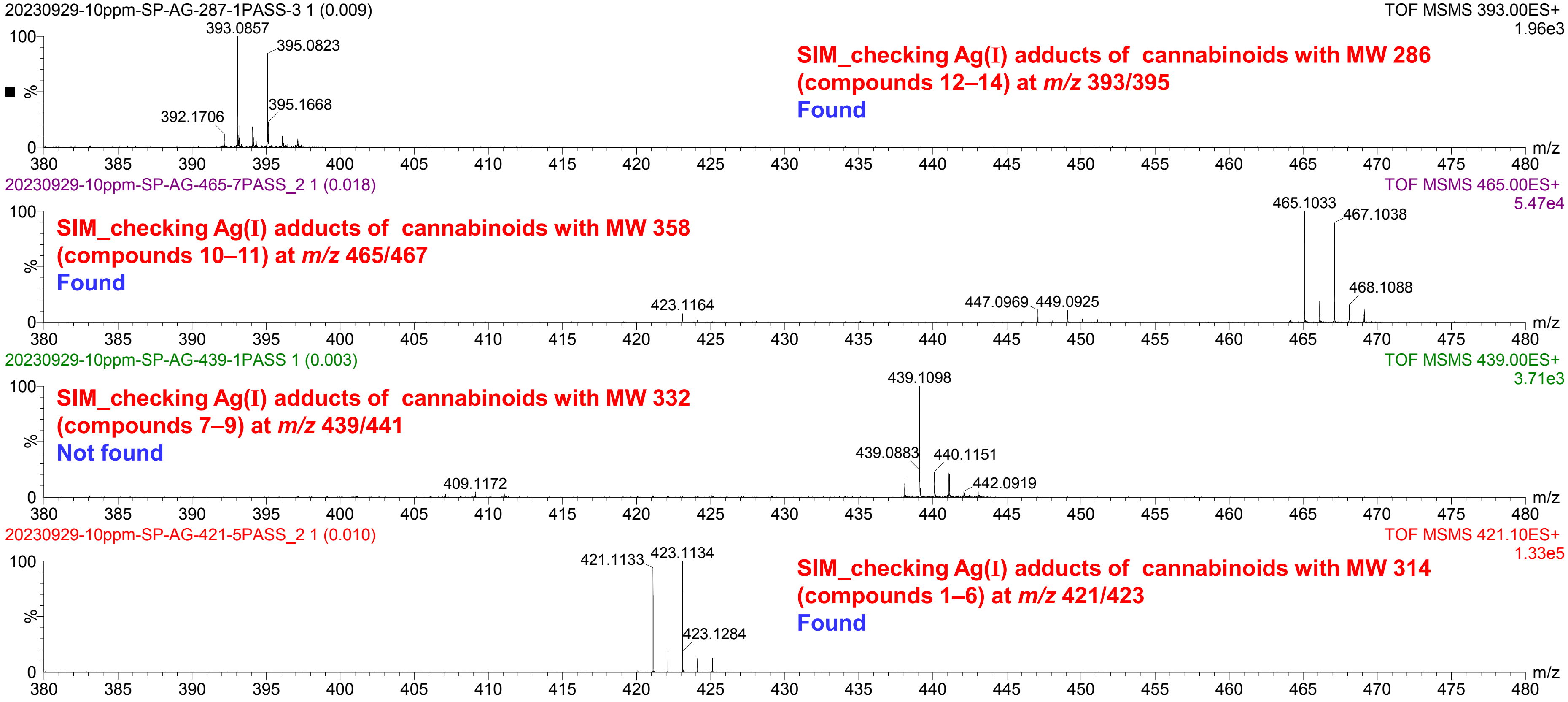

C#2 _mobility separation_for checking CCS

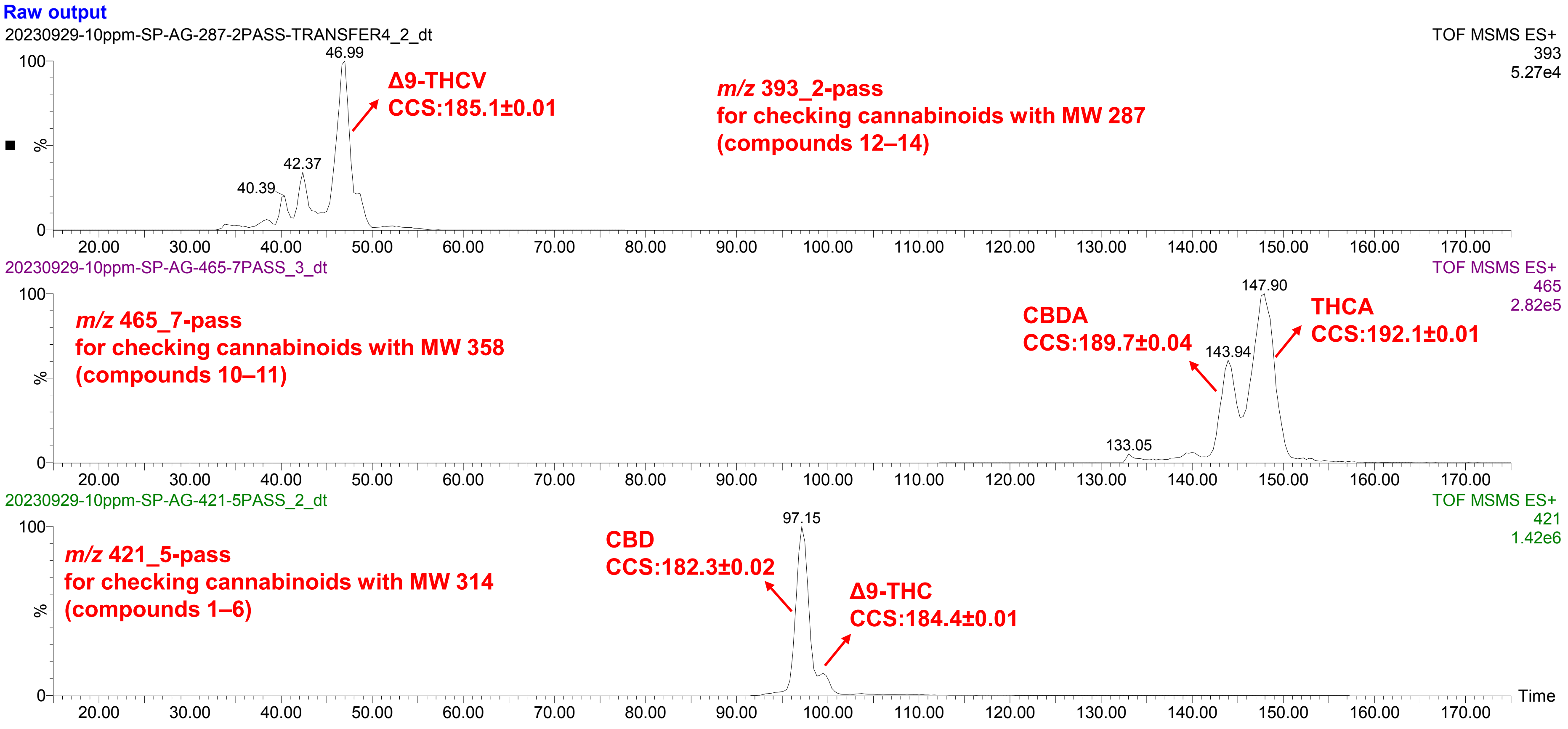

C#2_mobility separation+transfer fragmentation_for checking fragments

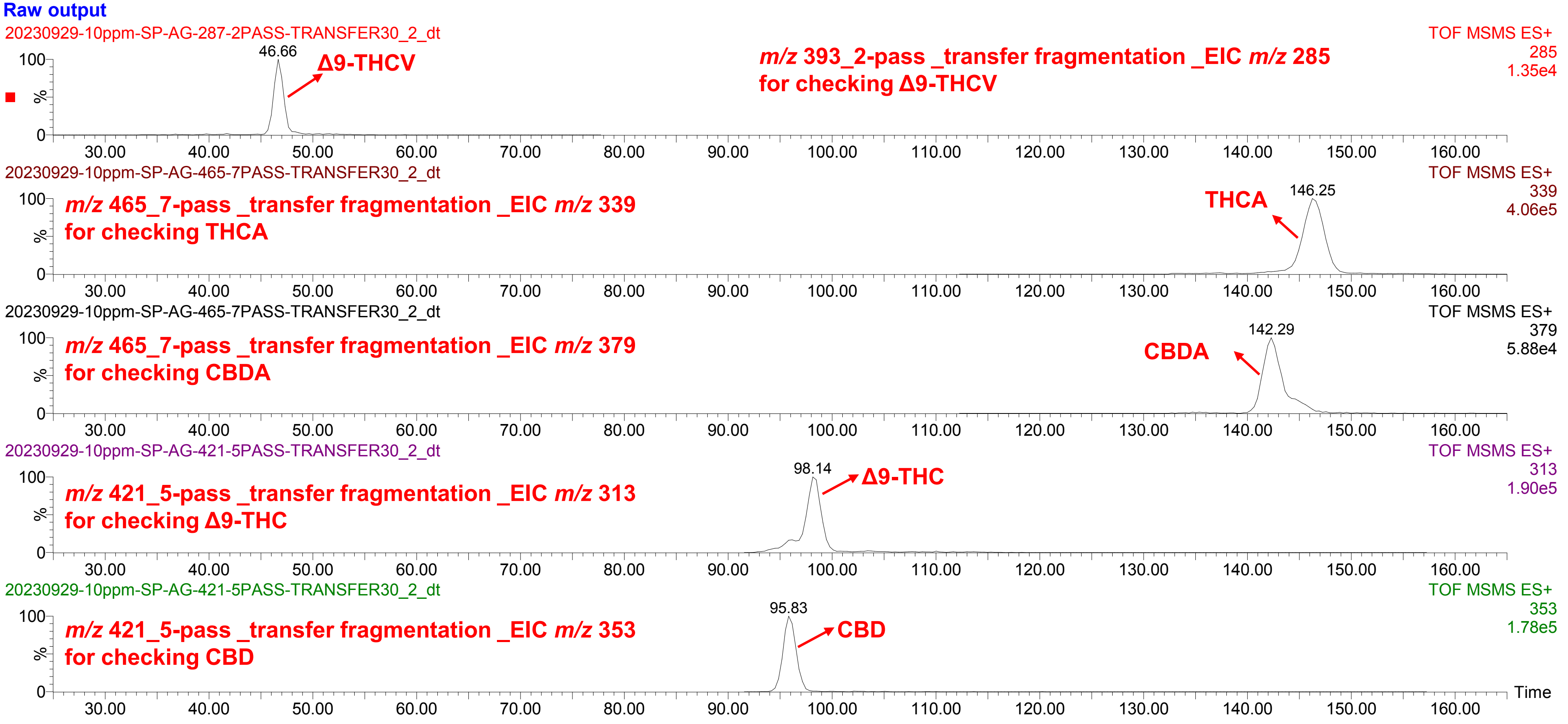

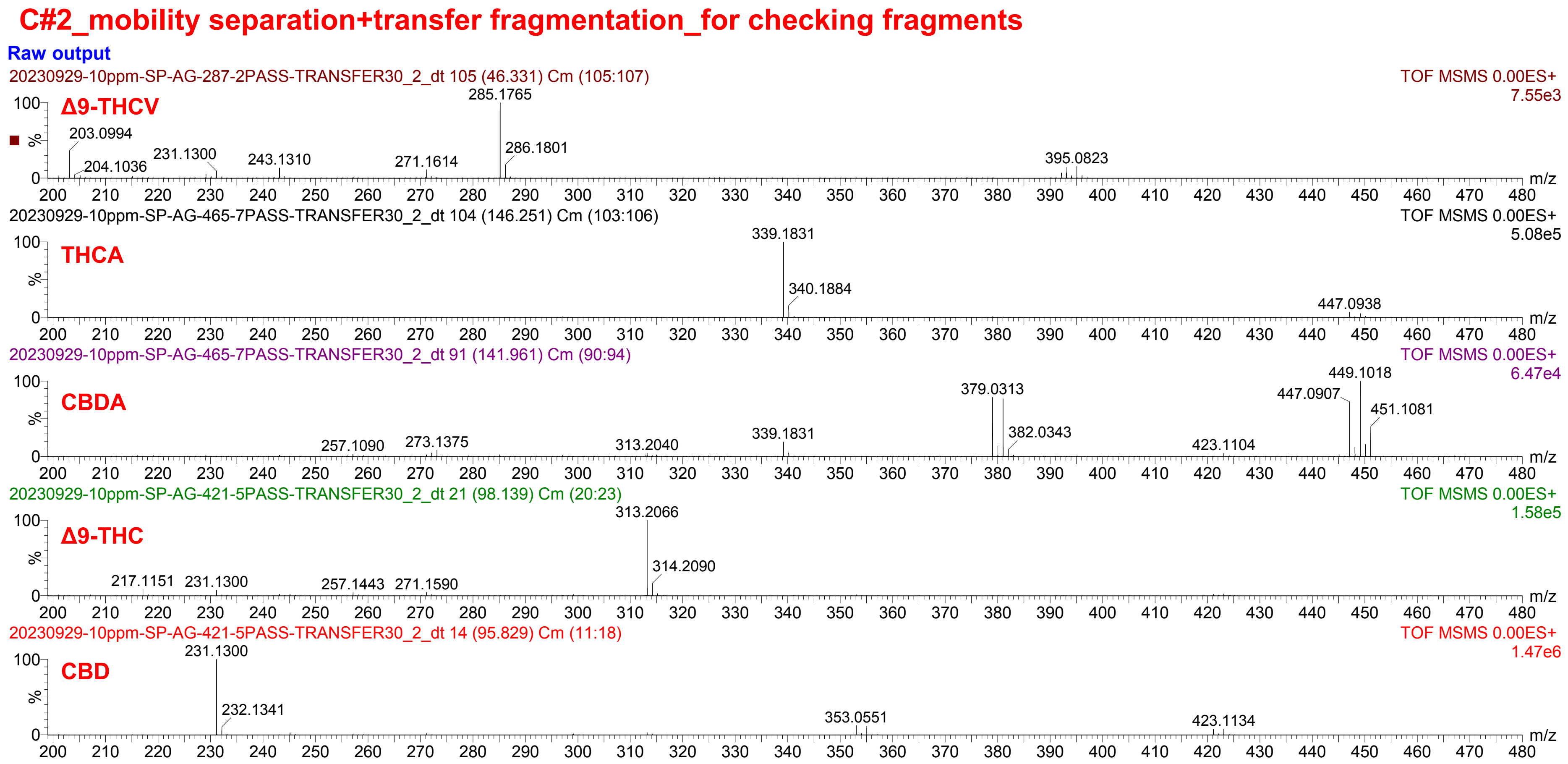

Figure S14-2. Mobiligram and mass spectra of cannabinoids in sample C#2.

C#3 _full scan_for checking Ag(I) adducts

Raw output

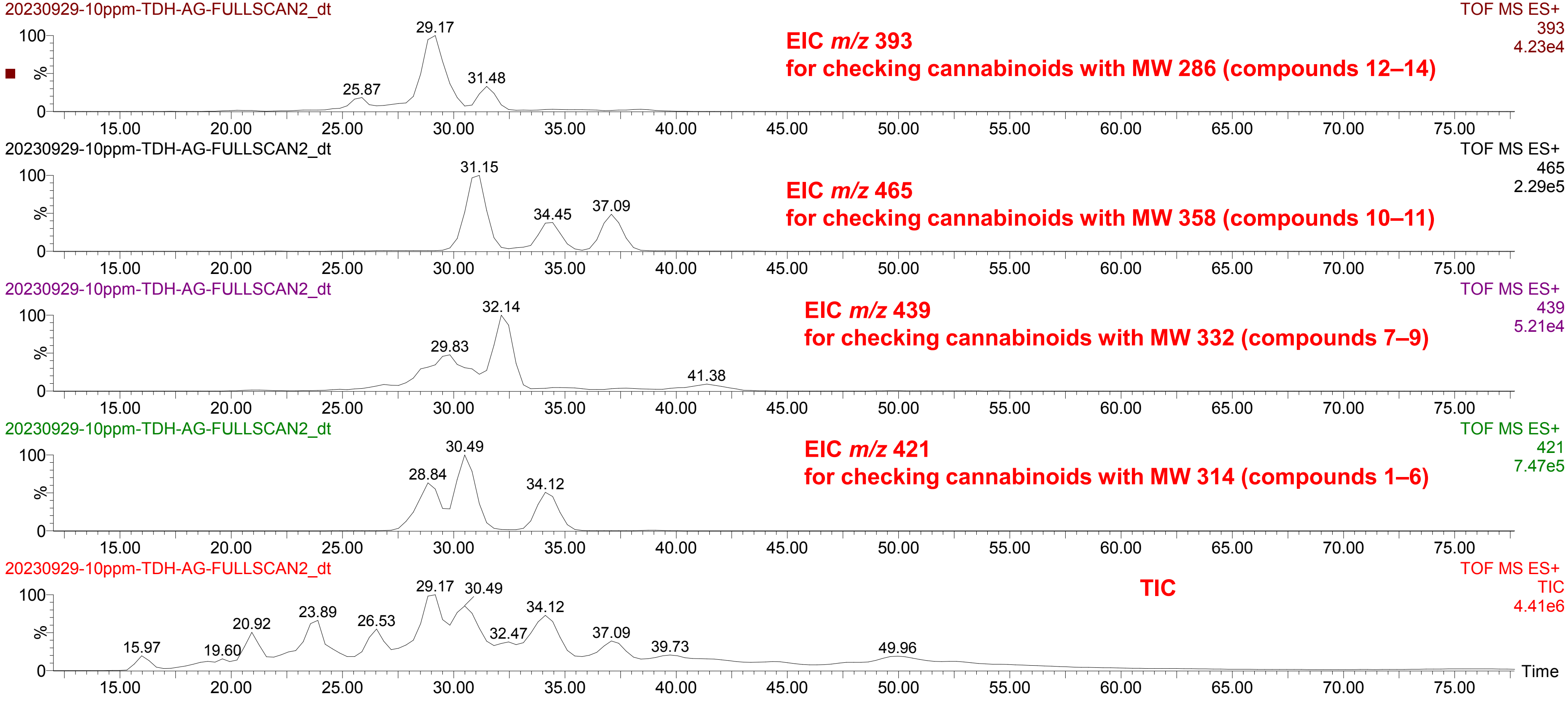

C#3 _SIM_for checking Ag(I) adducts

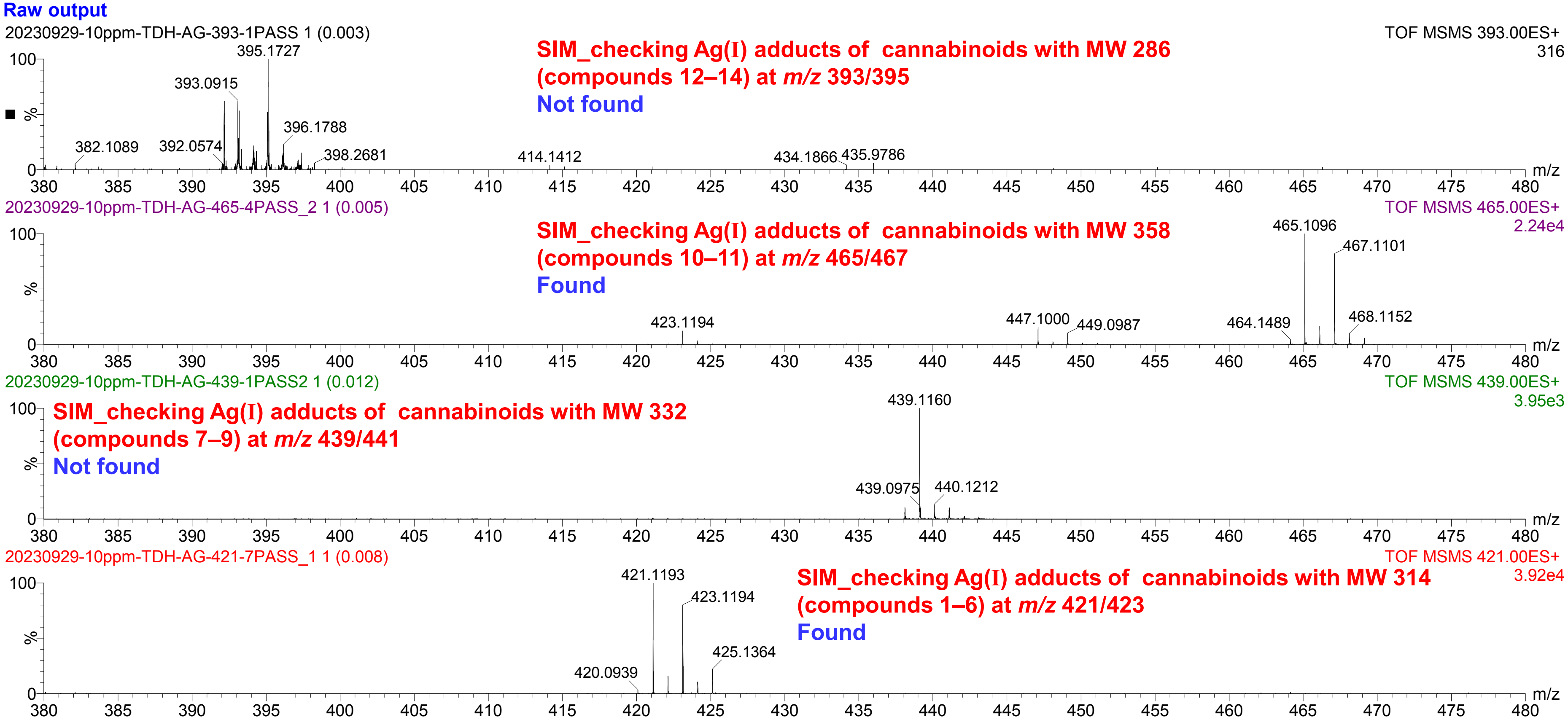

C#3 _mobility separation_for checking CCS

Raw output

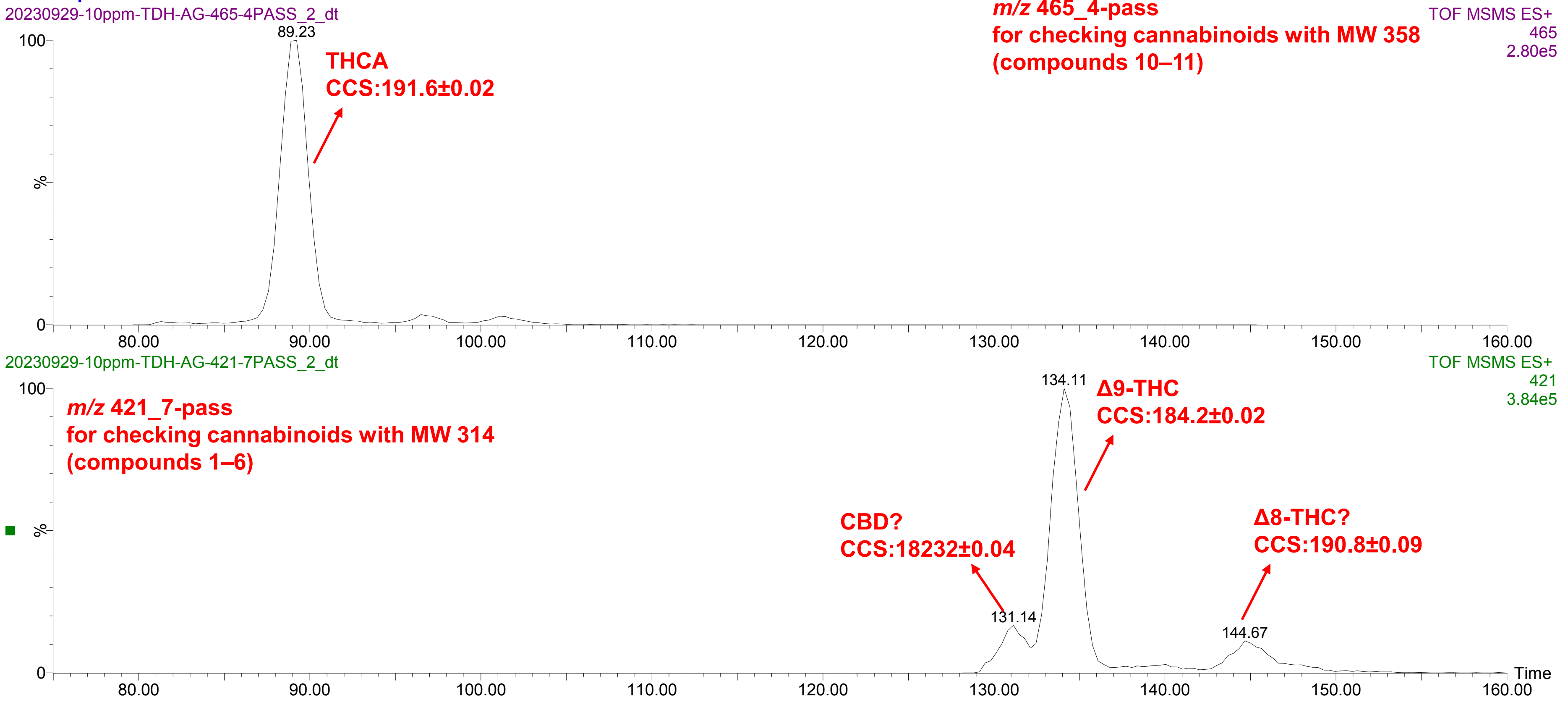

C#3_mobility separation+transfer fragmentation_for checking fragments

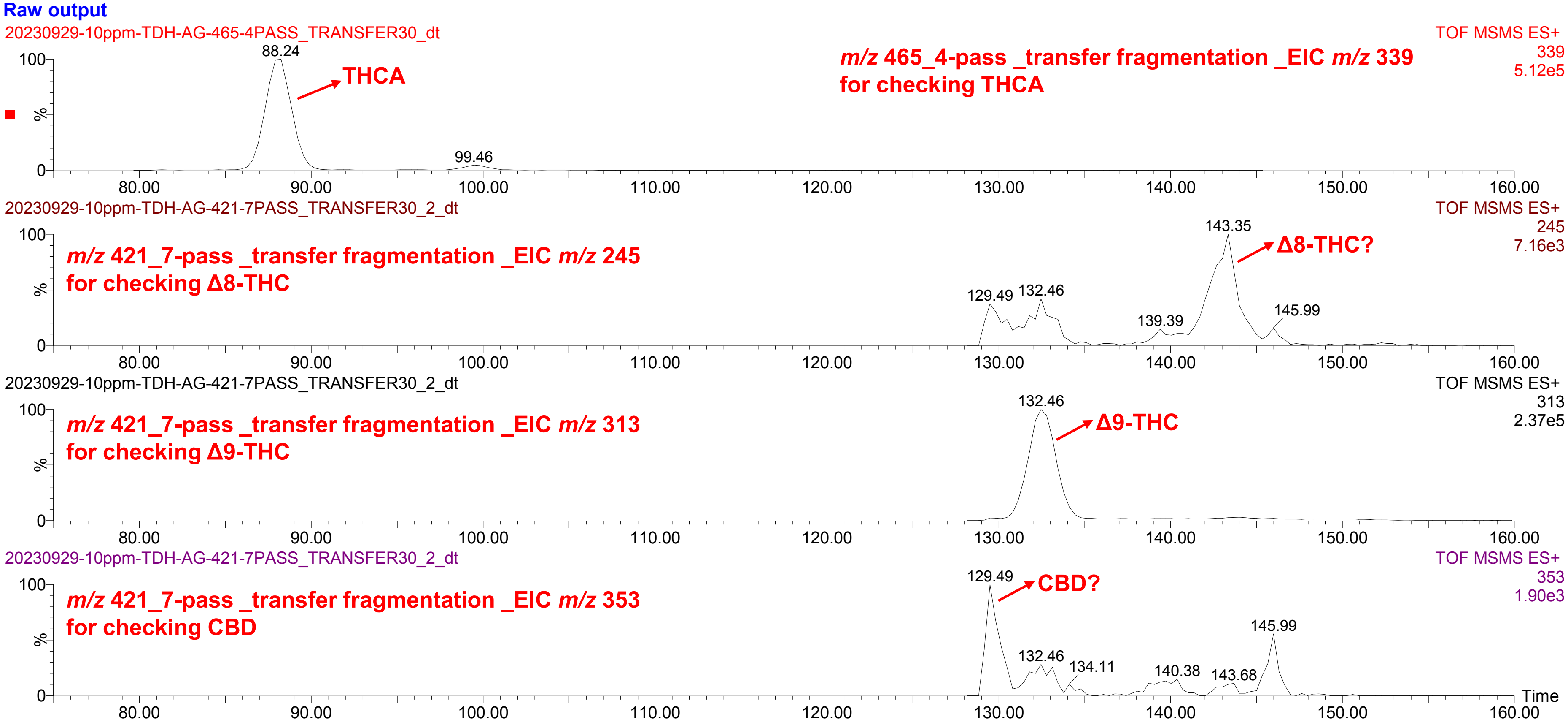

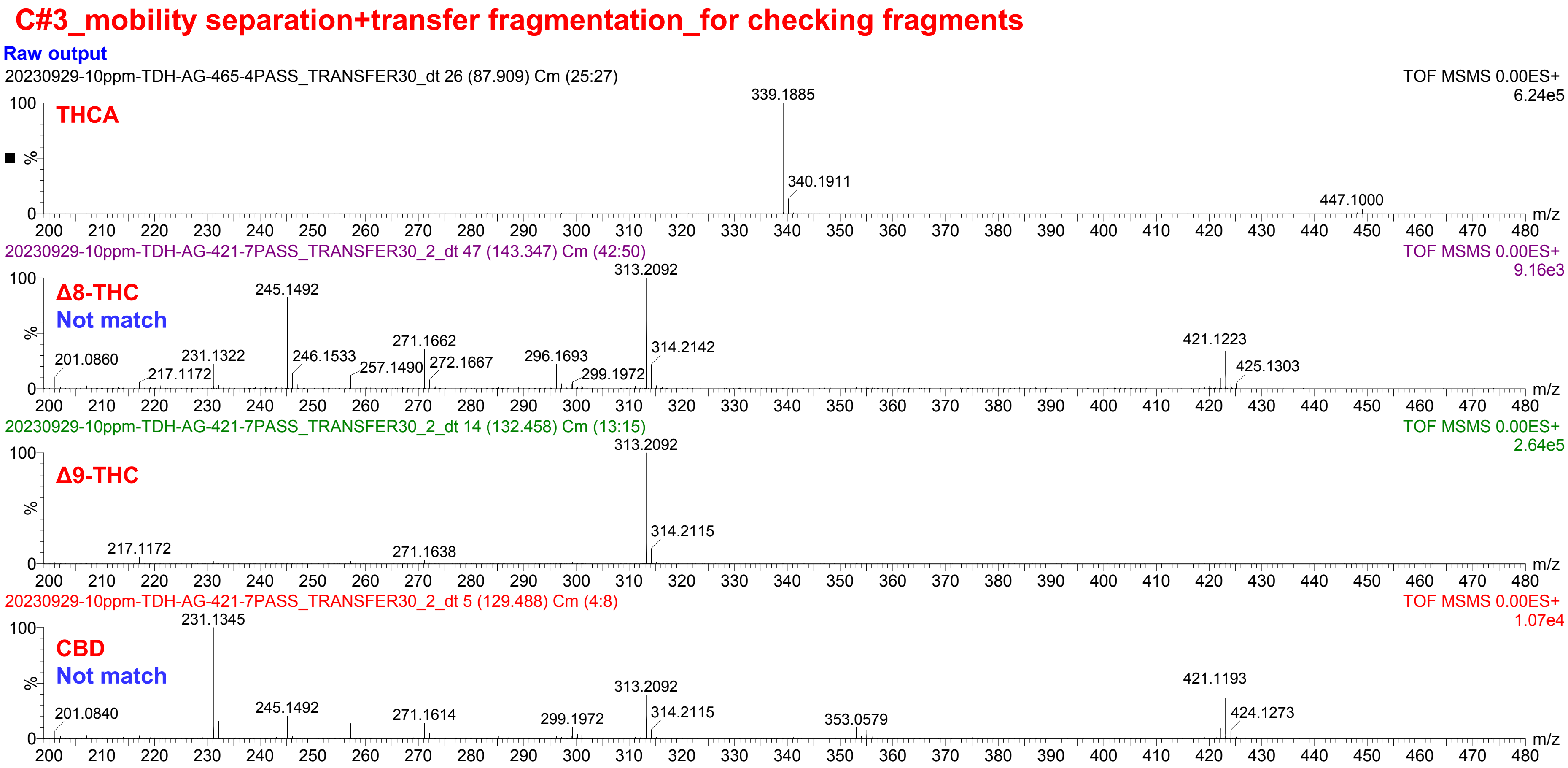

Figure S14-3. Mobiligram and mass spectra of cannabinoids in sample C#3.

G#1 _full scan_for checking Ag(I) adducts

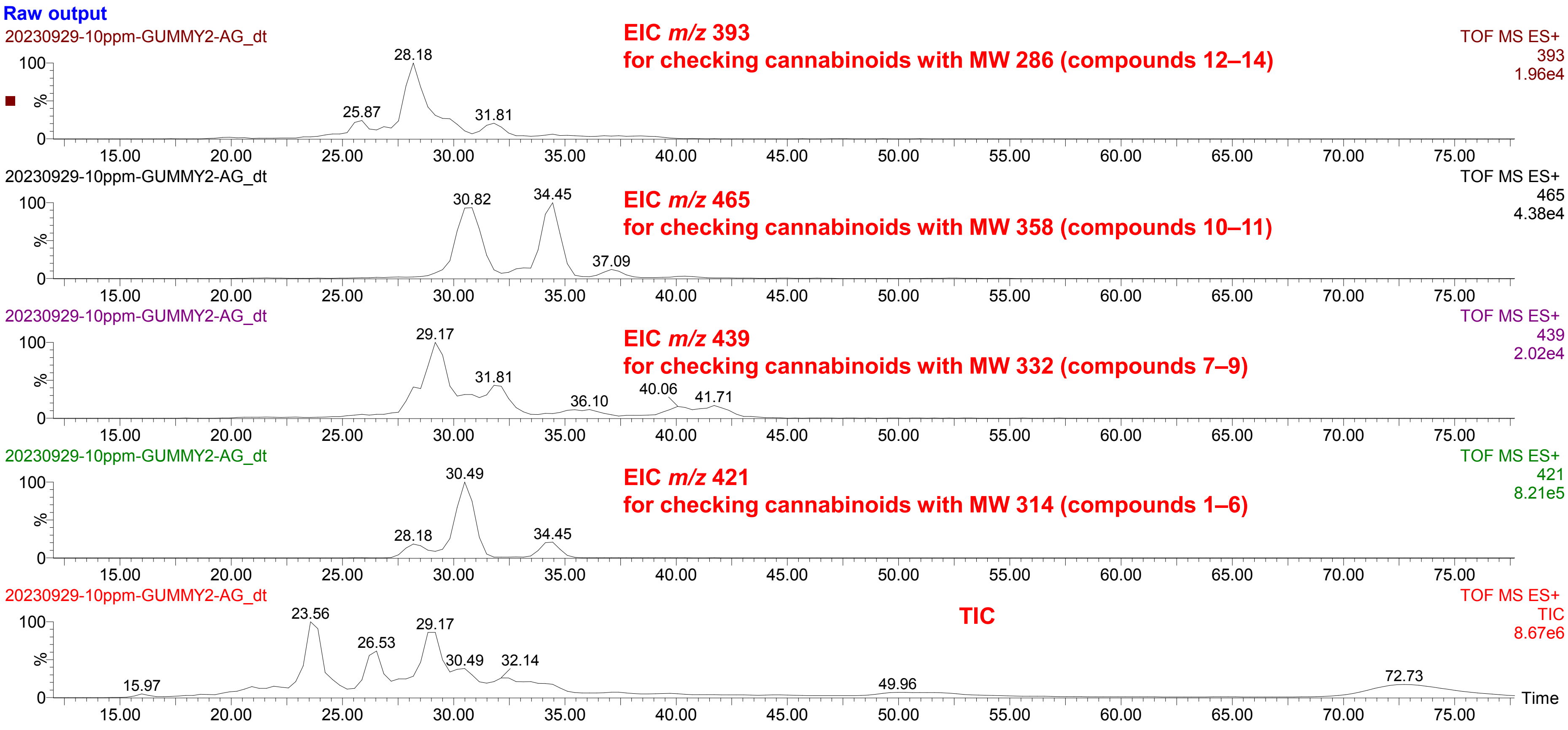

G#1 _SIM_for checking Ag(I) adducts

Raw output

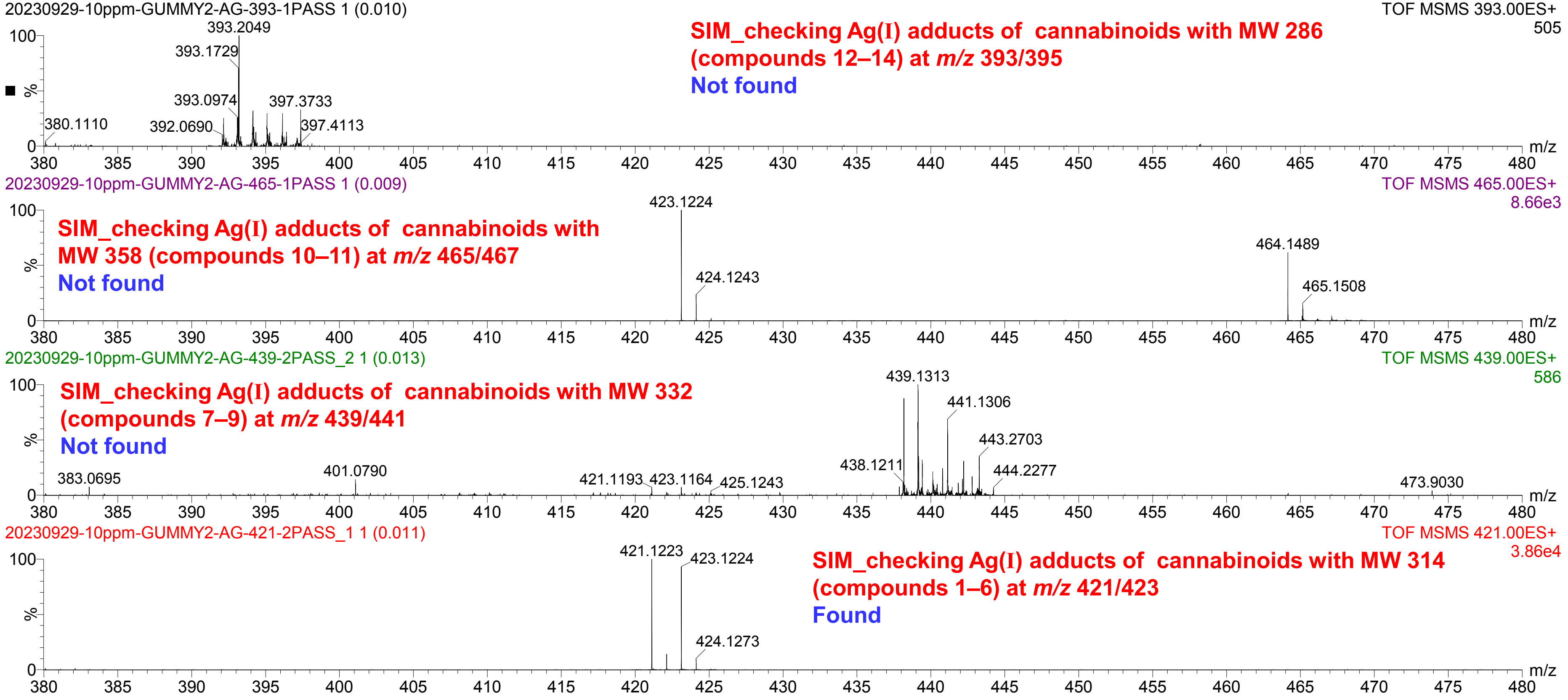

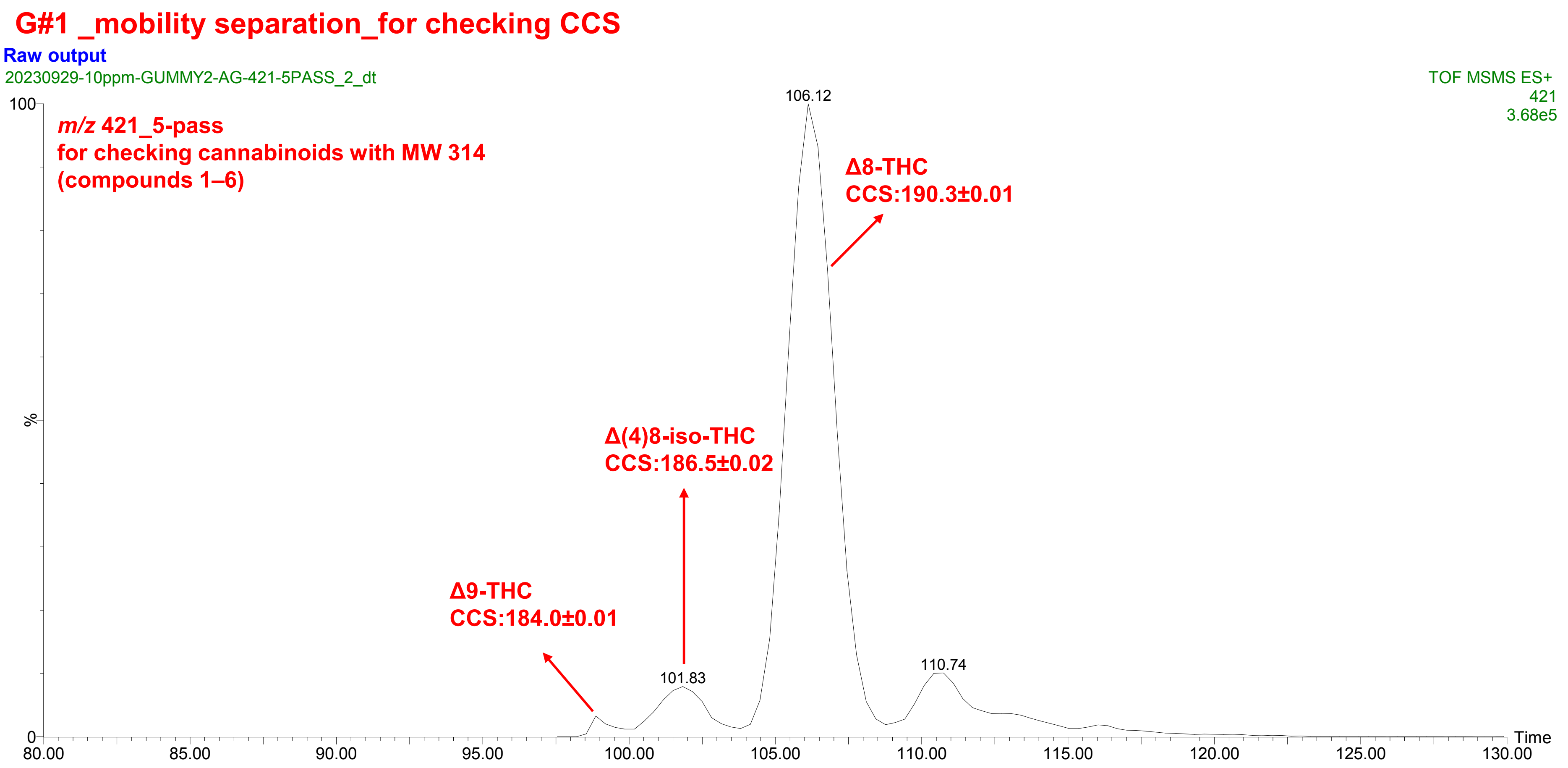

G#1 _mobility separation_for checking CCS
Raw output
20230929-10ppm-GUMMY2-AG-421-5PASS_2_dt
TOF MSMS ES+
421
3.68e5
m/z 421_5-pass
for checking cannabinoids with MW 314
(compounds 1–6)
Δ8-THC
CCS:190.3±0.01
Δ(4)8-iso-THC
CCS:186.5±0.02
Δ9-THC
CCS:184.0±0.01
106.12
101.83
110.74
100
%
0
80.00
85.00
90.00
95.00
100.00
105.00
110.00
115.00
120.00
125.00
130.00
Time

G#1_mobility separation+transfer fragmentation_for checking fragments

Raw output

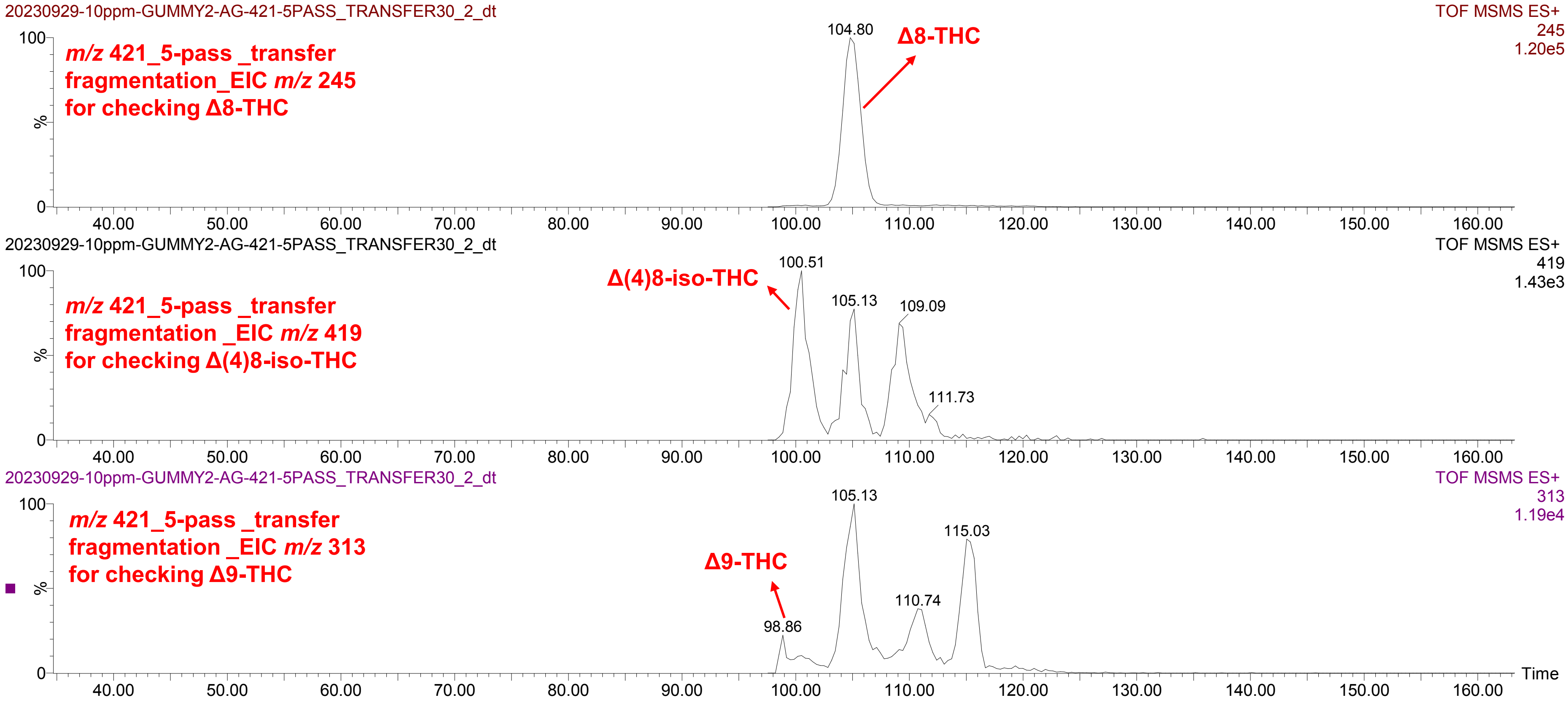

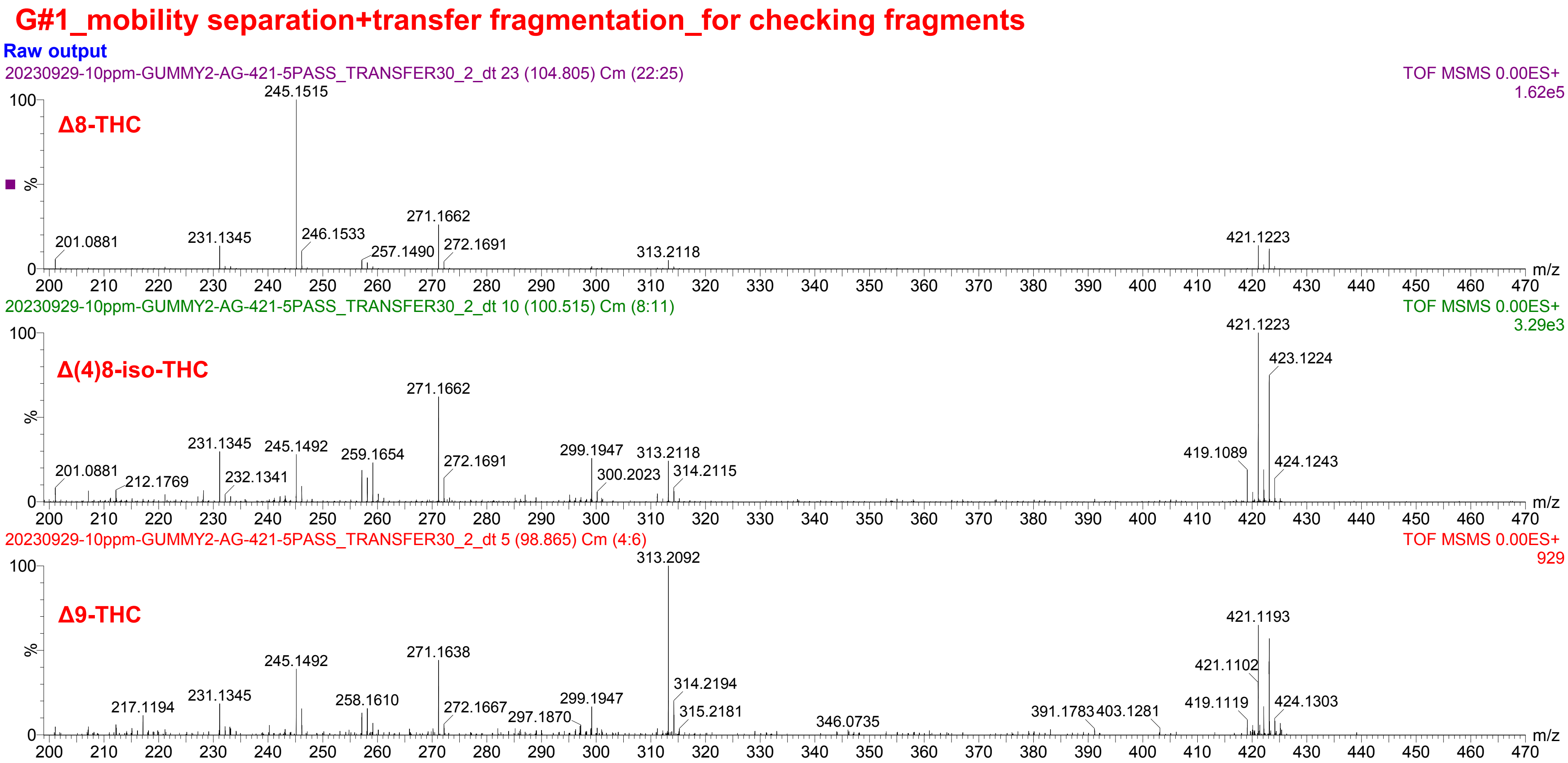

Figure S14-4. Mobiligram and mass spectra of cannabinoids in sample G#1.

G#2 _full scan_for checking Ag(I) adducts

Raw output

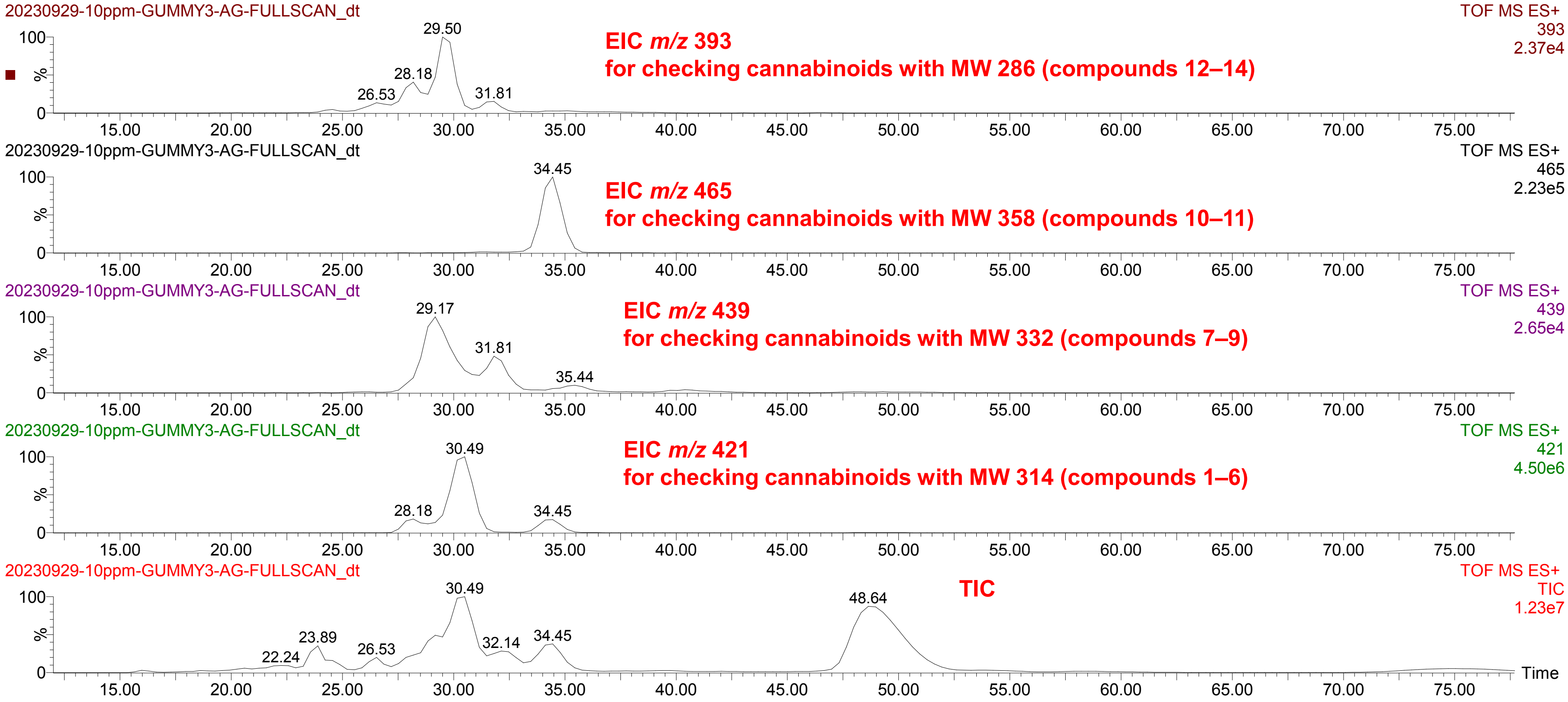

G#2 _SIM_for checking Ag(I) adducts

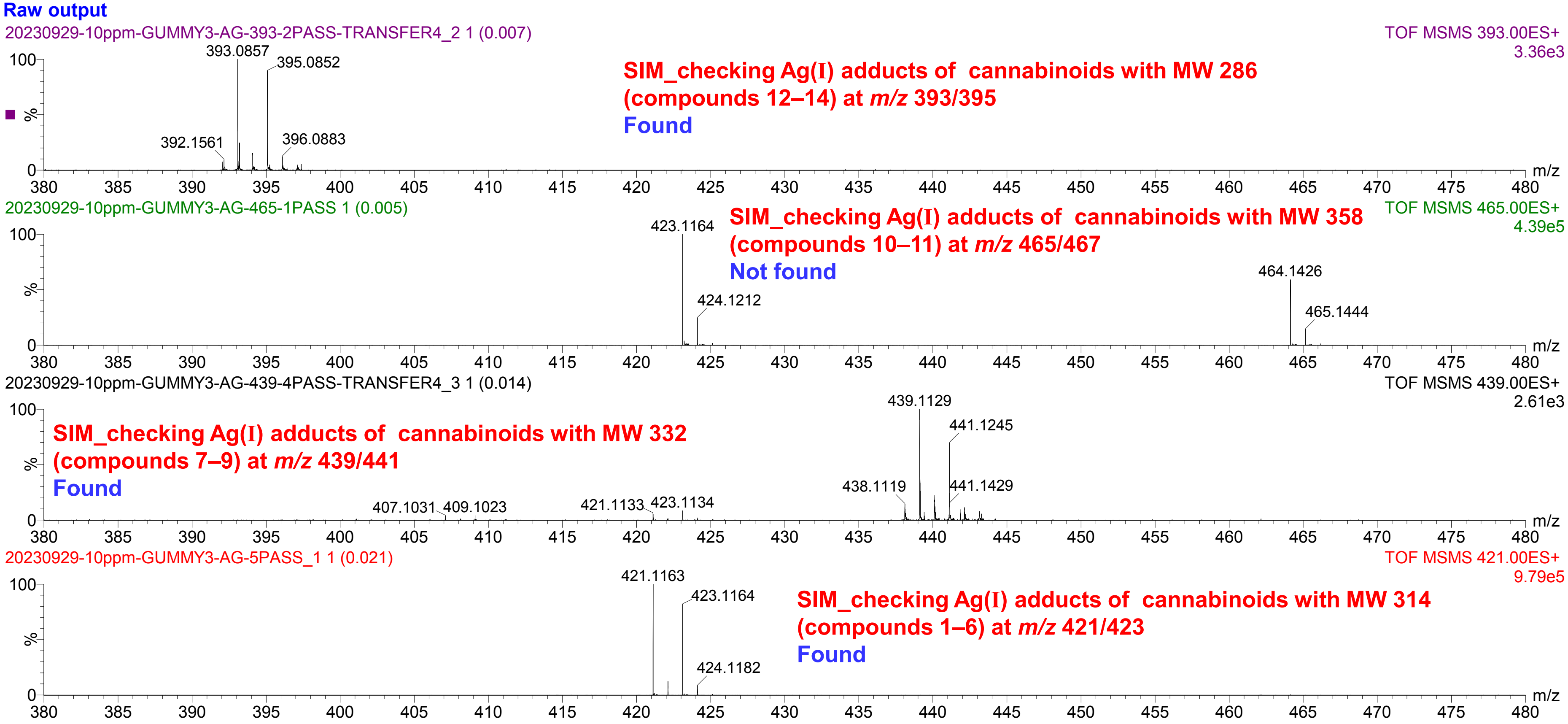

G#2 _mobility separation_for checking CCS

Raw output

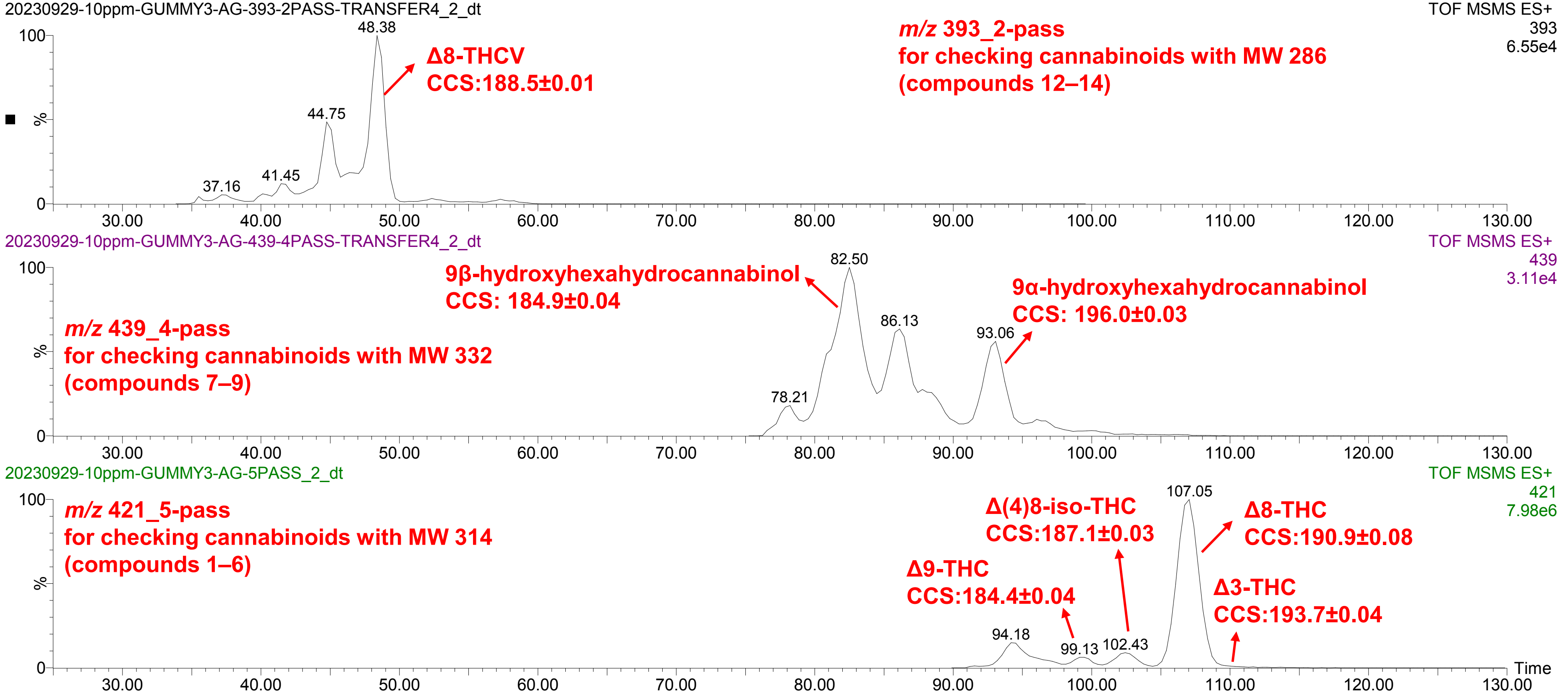

G#2_mobility separation+transfer fragmentation_for checking fragments

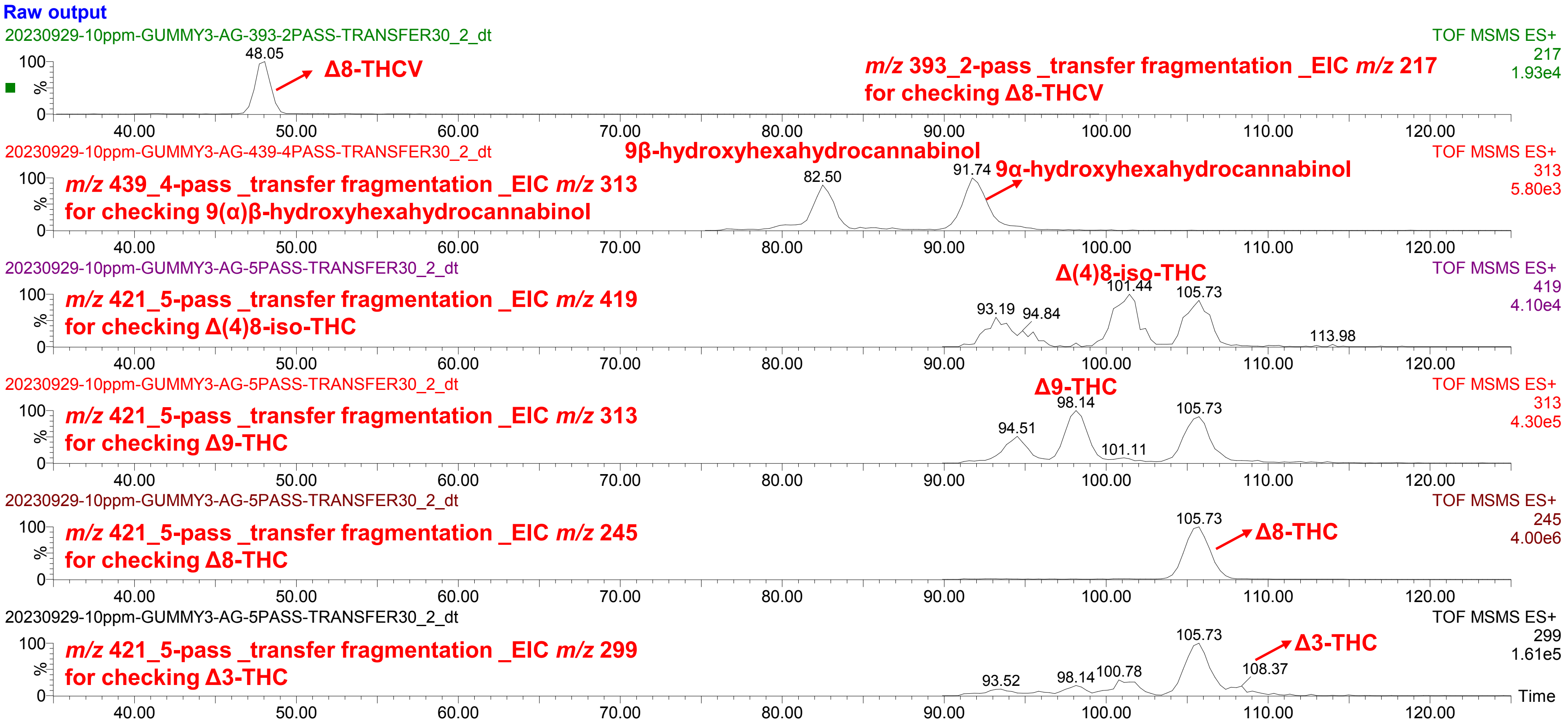

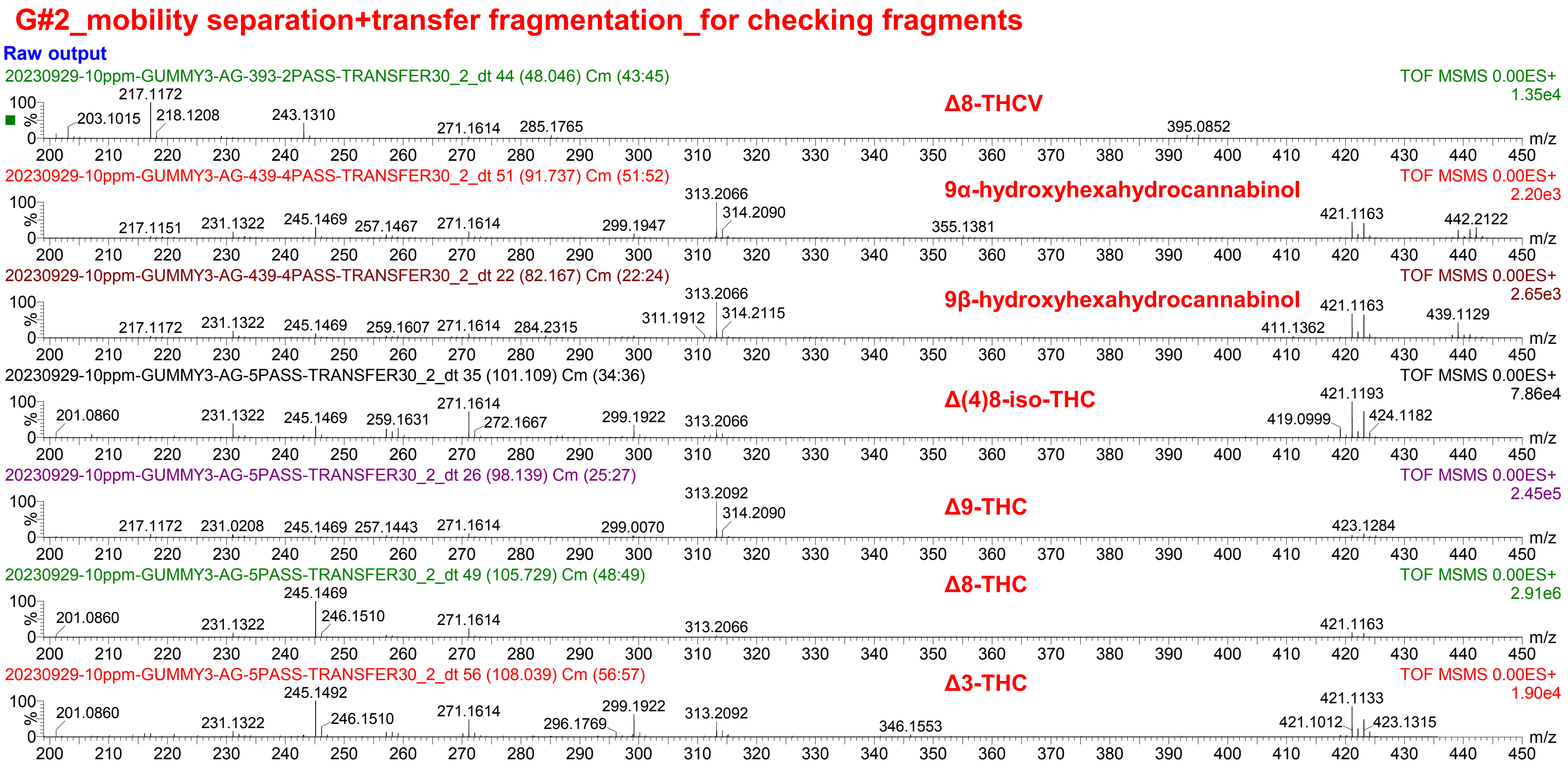

Figure S14-5. Mobiligram and mass spectra of cannabinoids in sample G#2.

R#1 _SIM_for checking Ag(I) adducts

Raw output

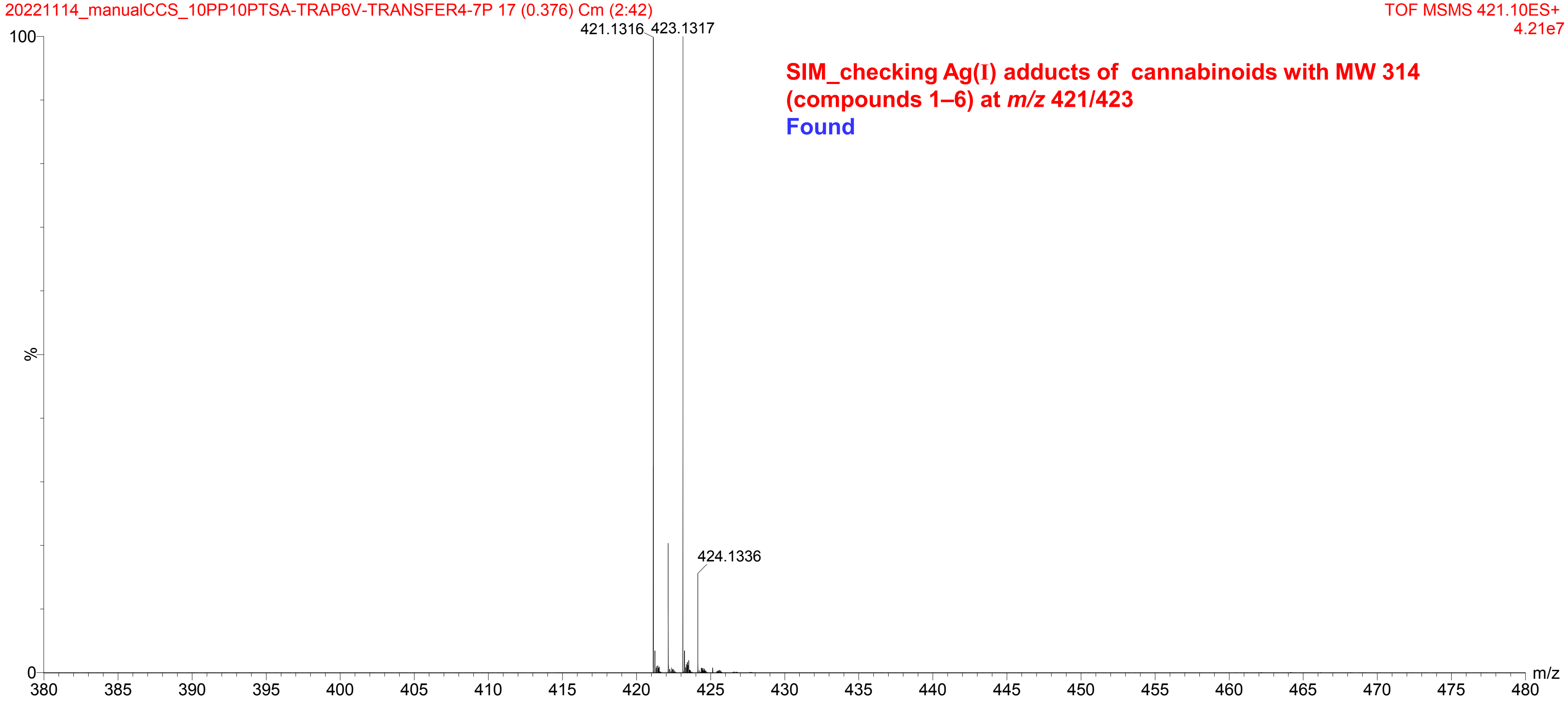

R#1 _mobility separation_for checking CCS

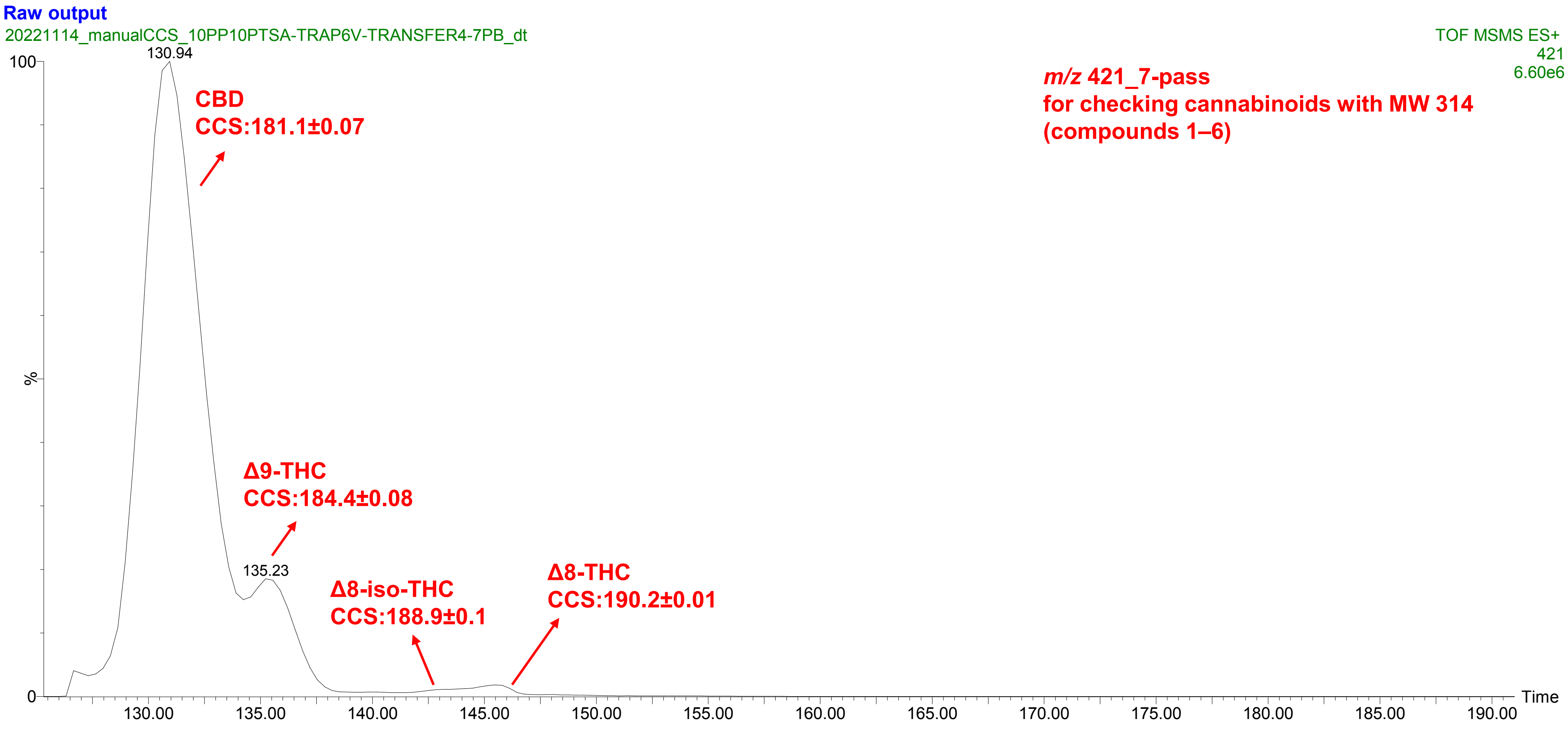

R#1_mobility separation+transfer fragmentation_for checking fragments

Raw output

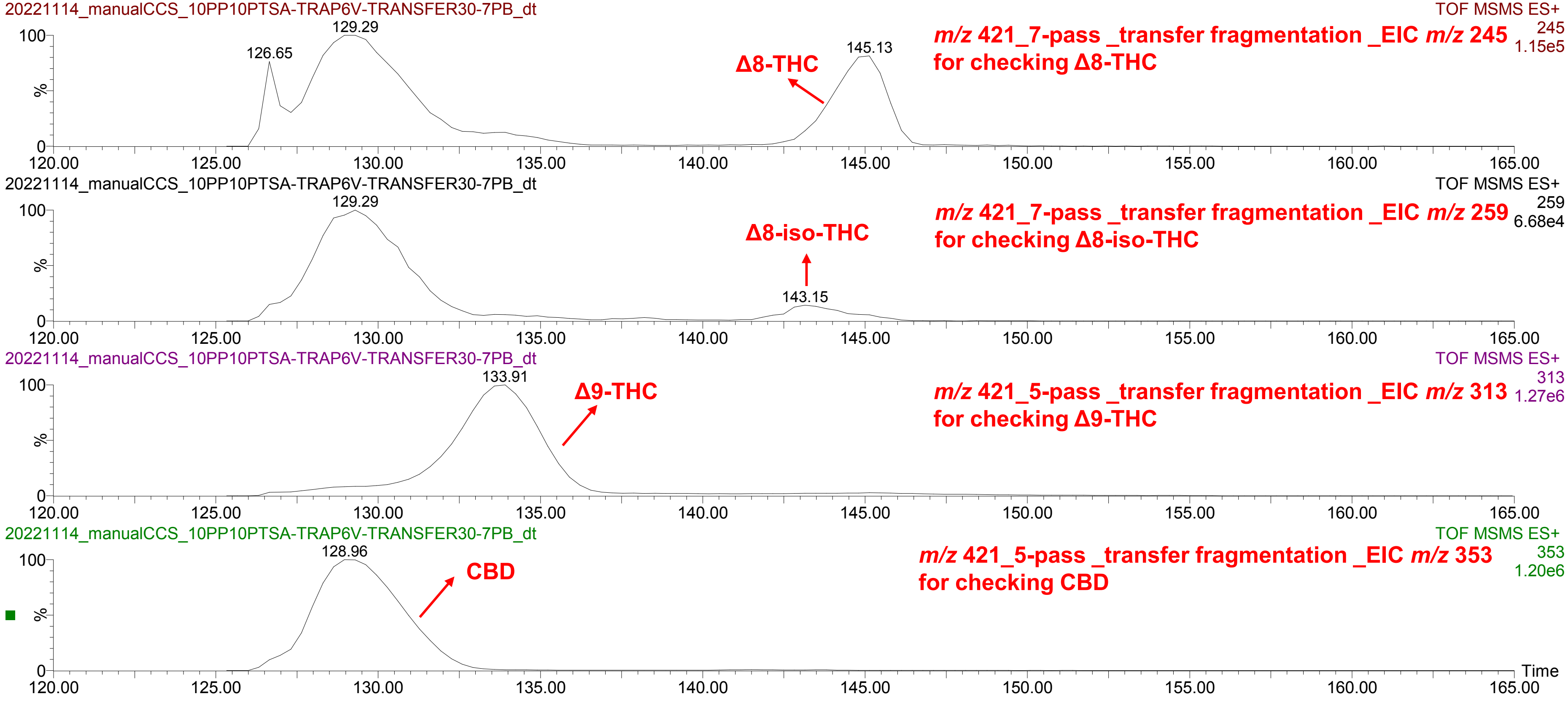

R#1_mobility separation+transfer fragmentation_for checking fragments

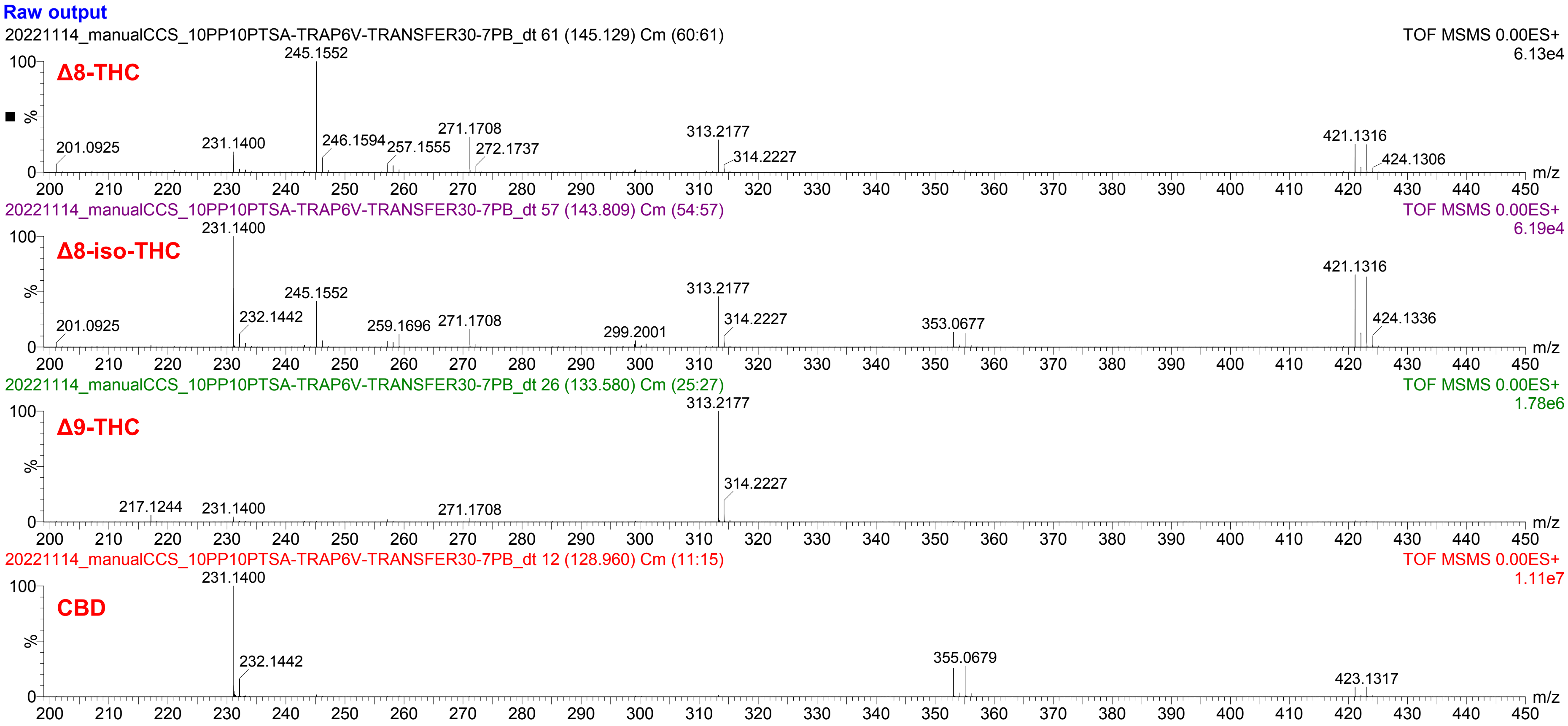

Figure S14-6. Mobiligram and mass spectra of cannabinoids in sample R#1.

R#2 _SIM_for checking Ag(I) isotope pattern

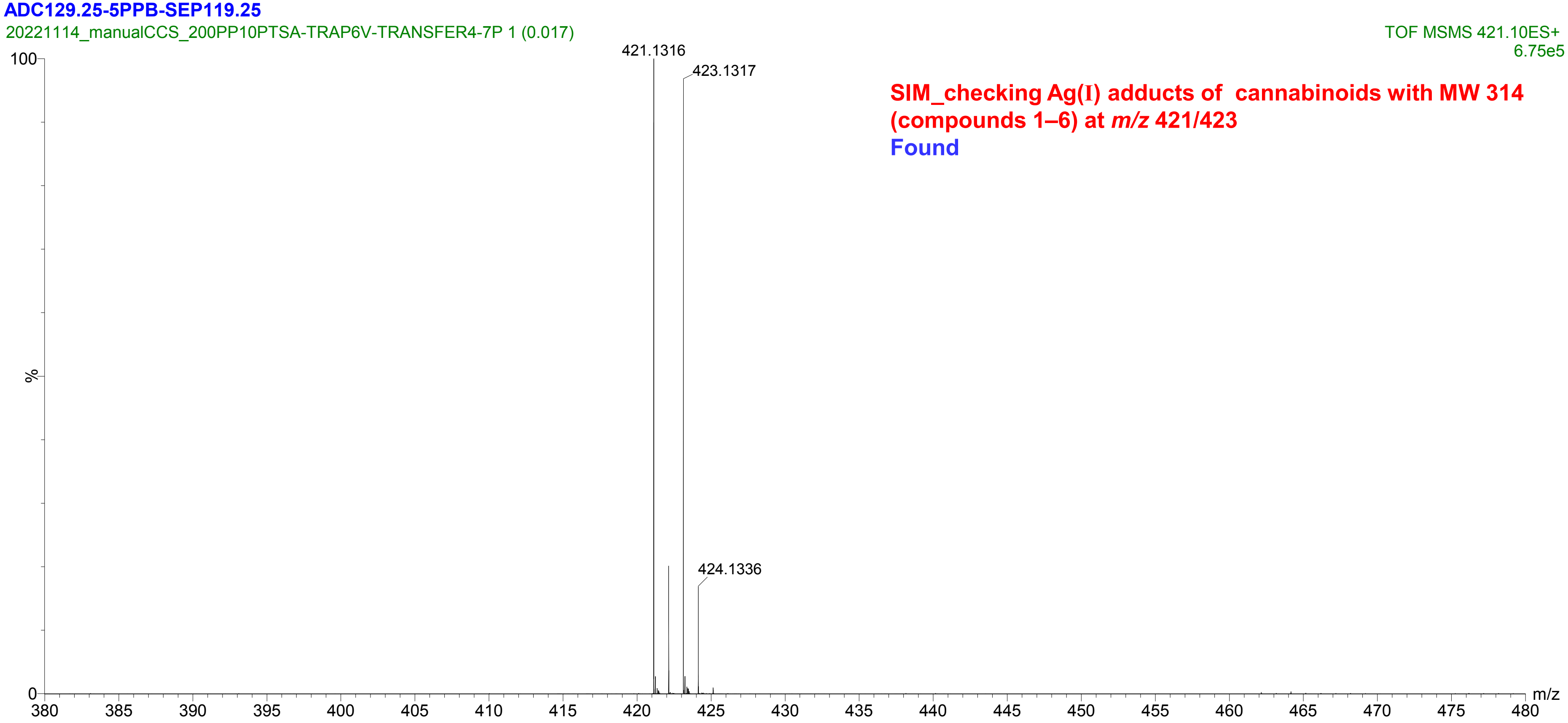

SIM_checking Ag(I) adducts of cannabinoids with MW 314 (compounds 1–6) at *m/z* 421/423

Found

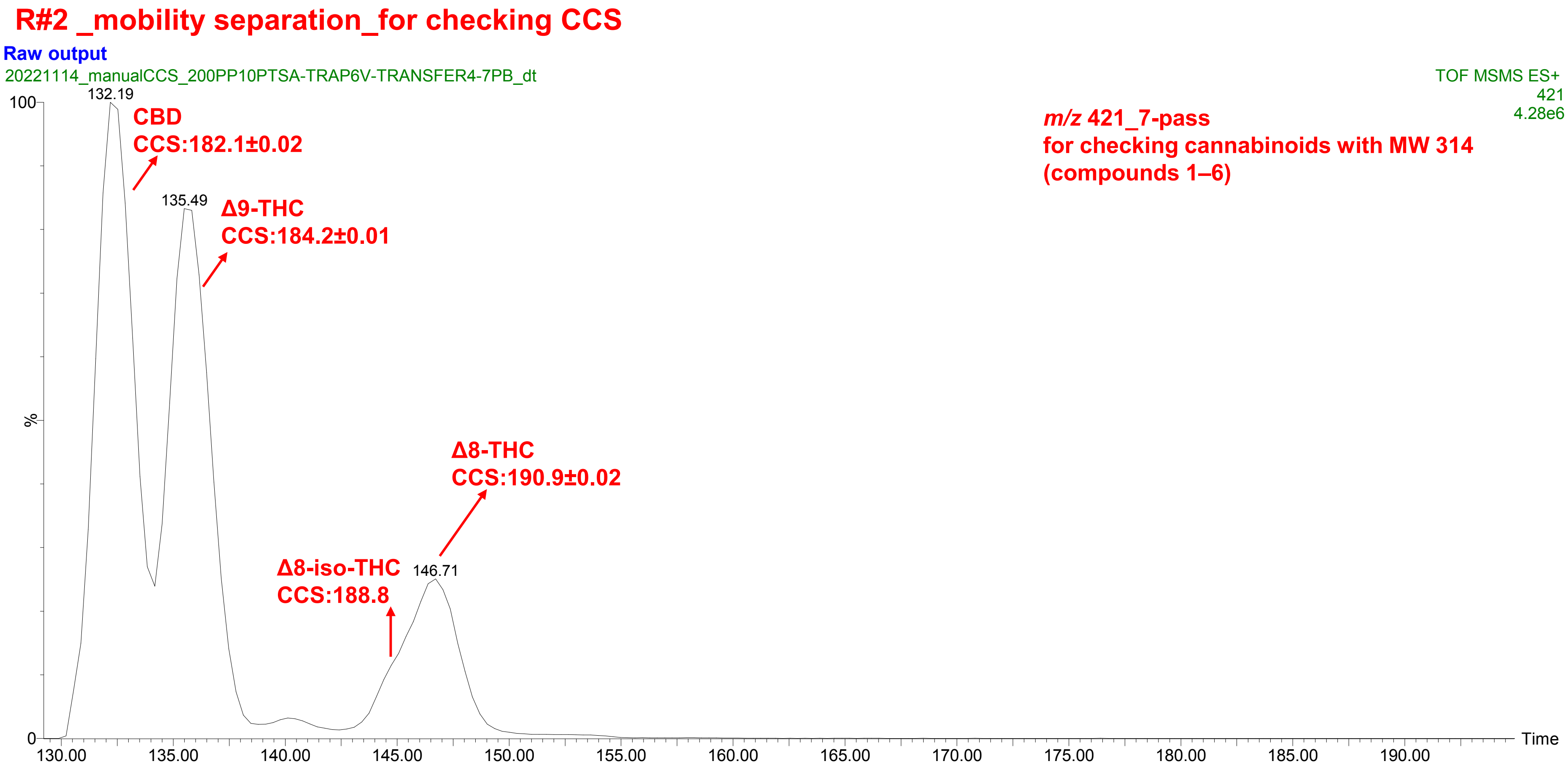
R#2 _mobility separation_for checking CCS
Raw output
20221114_manualCCS_200PP10PTSA-TRAP6V-TRANSFER4-7PB_dt
TOF MSMS ES+
421
4.28e6
m/z 421_7-pass
for checking cannabinoids with MW 314
(compounds 1–6)
132.19
CBD
CCS:182.1±0.02
135.49
Δ9-THC
CCS:184.2±0.01
Δ8-THC
CCS:190.9±0.02
Δ8-iso-THC
CCS:188.8
146.71
100
%
0
130.00
135.00
140.00
145.00
150.00
155.00
160.00
165.00
170.00
175.00
180.00
185.00
190.00
Time

R#2_mobility separation+transfer fragmentation_for checking fragments

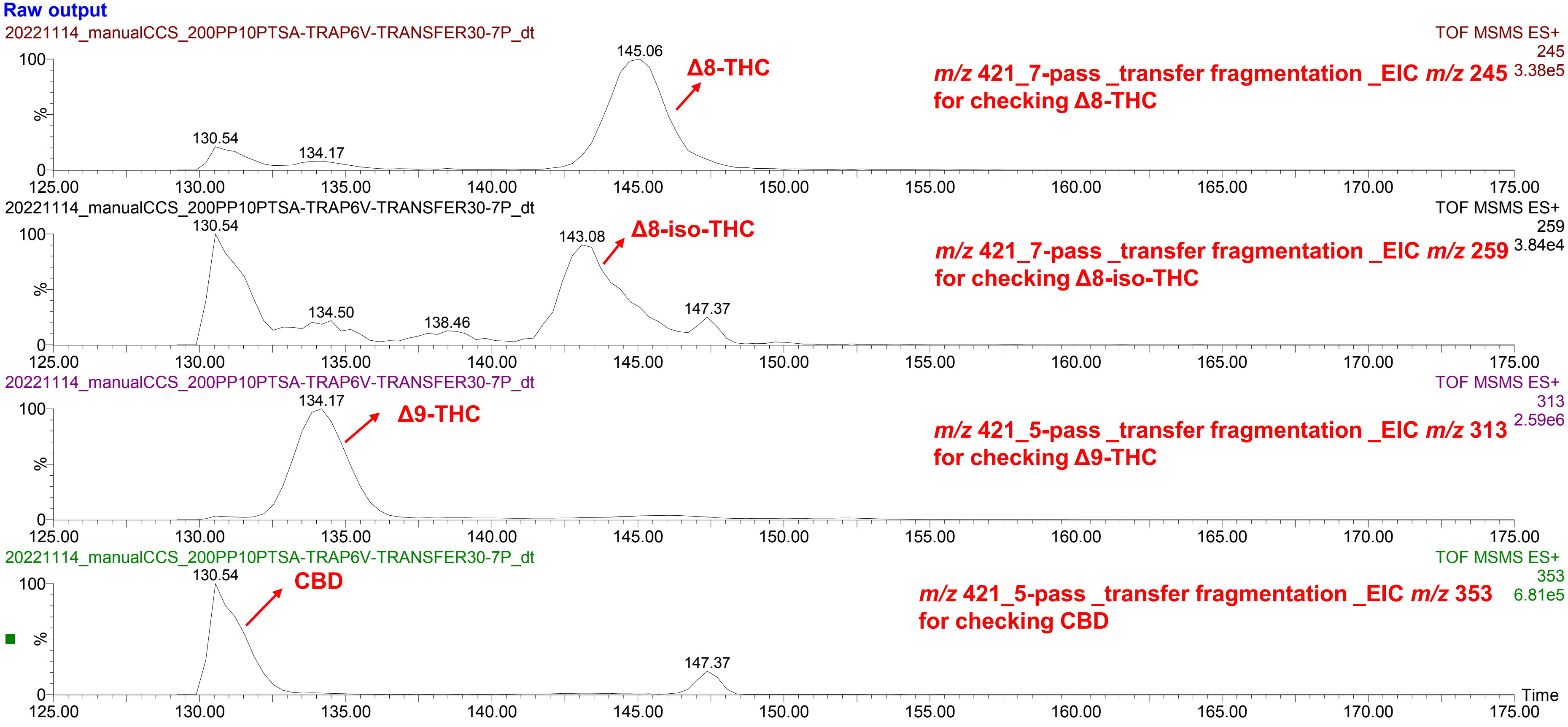

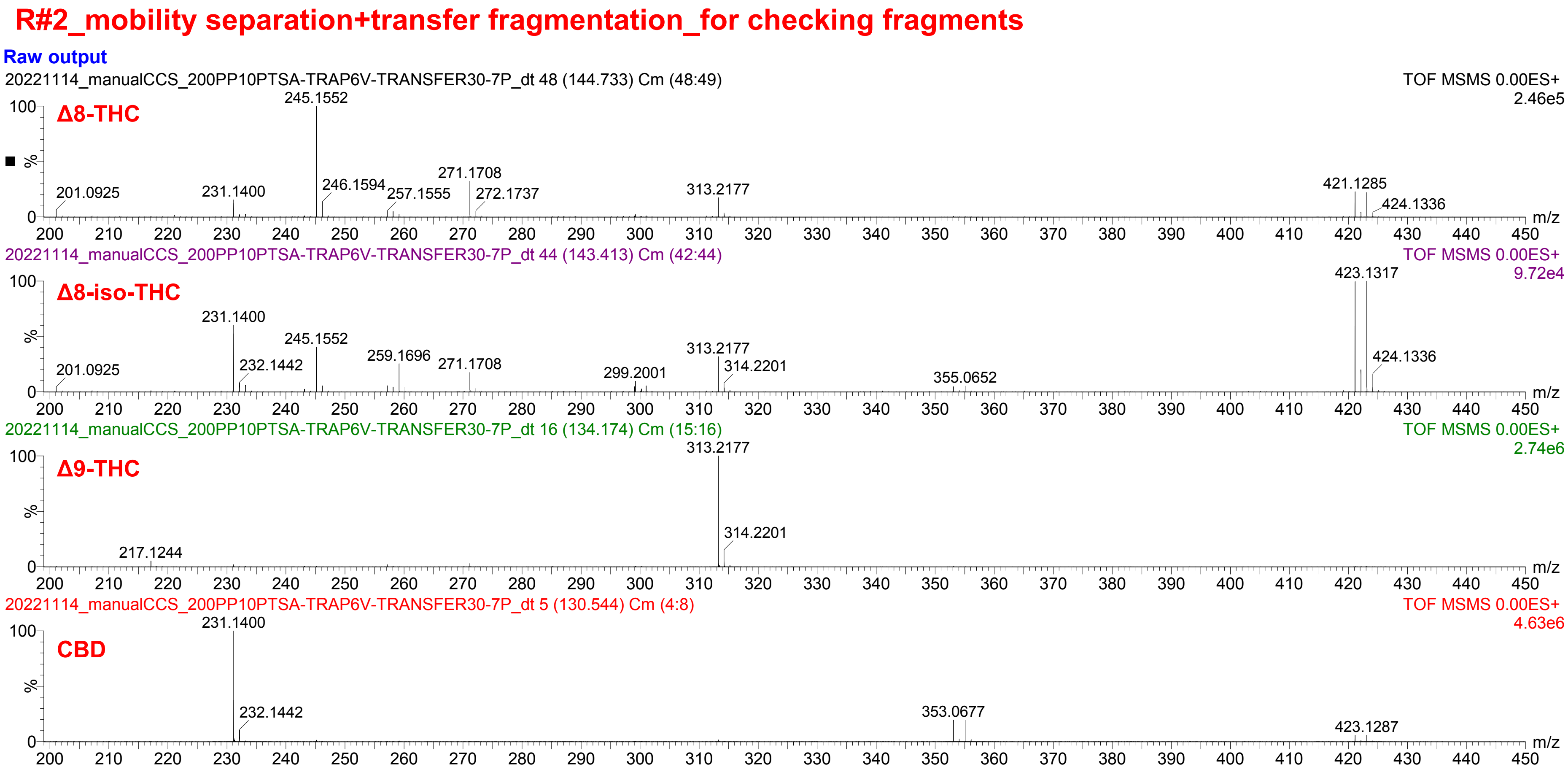

Figure S14-7. Mobiligram and mass spectra of cannabinoids in sample R#2.

R#3 _SIM_for checking Ag(I) adducts

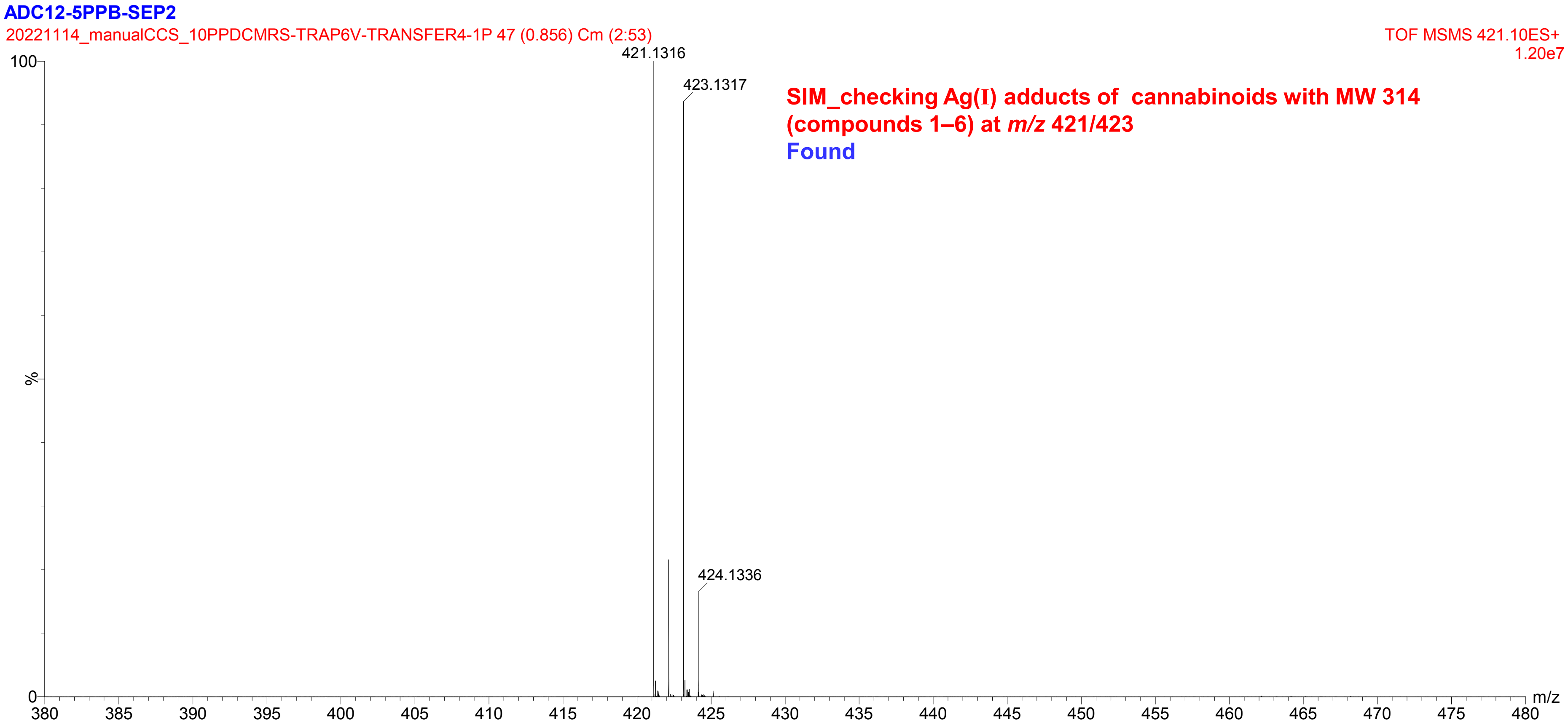

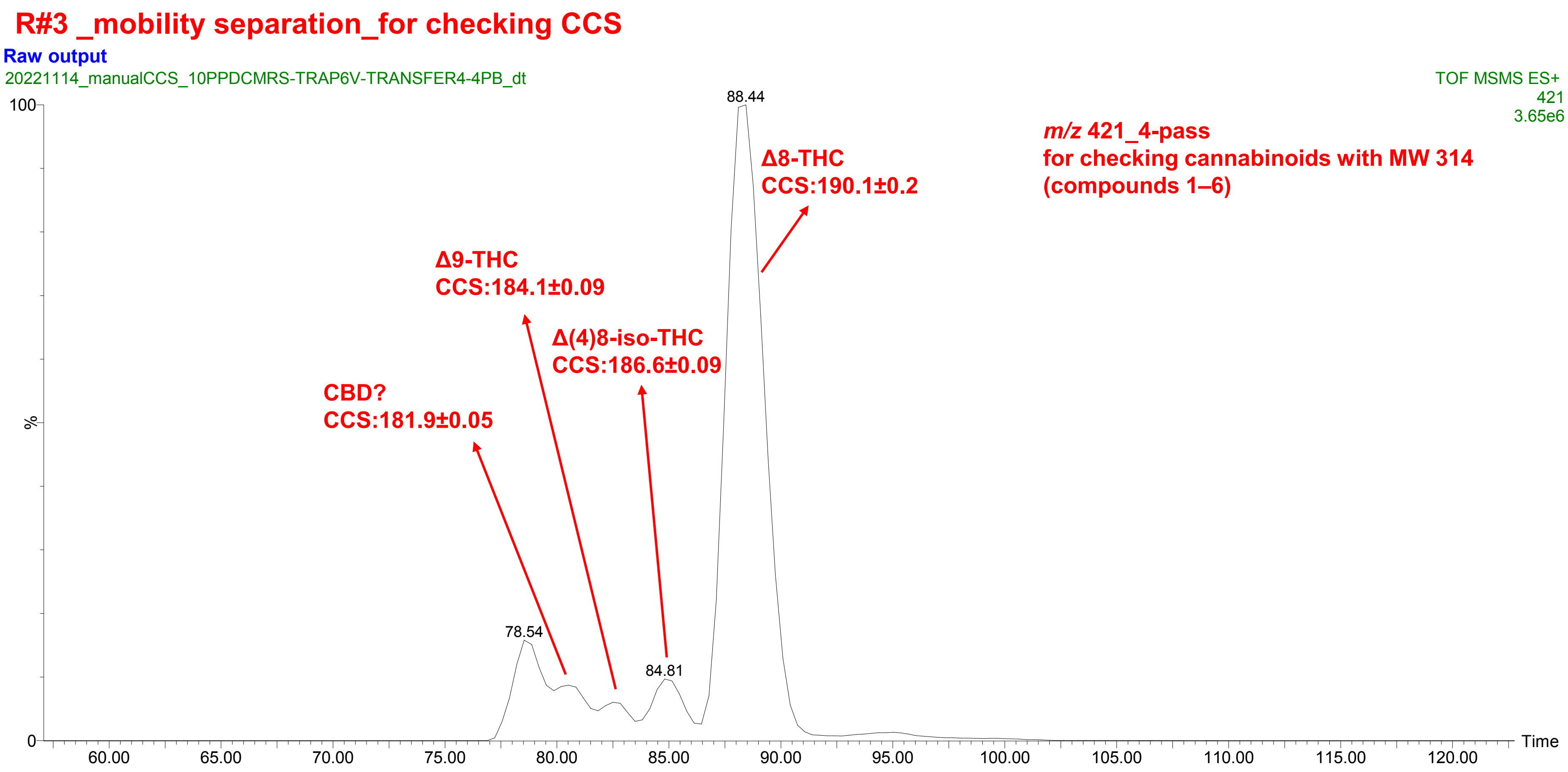
R#3 _mobility separation_for checking CCS
Raw output
20221114_manualCCS_10PPDCMRS-TRAP6V-TRANSFER4-4PB_dt
TOF MSMS ES+
421
3.65e6
m/z 421_4-pass
for checking cannabinoids with MW 314
(compounds 1–6)
Δ8-THC
CCS:190.1±0.2
Δ9-THC
CCS:184.1±0.09
Δ(4)8-iso-THC
CCS:186.6±0.09
CBD?
CCS:181.9±0.05
88.44
78.54
84.81
100
%
0
Time
60.00
65.00
70.00
75.00
80.00
85.00
90.00
95.00
100.00
105.00
110.00
115.00
120.00

R#3_mobility separation+transfer fragmentation_for checking fragments

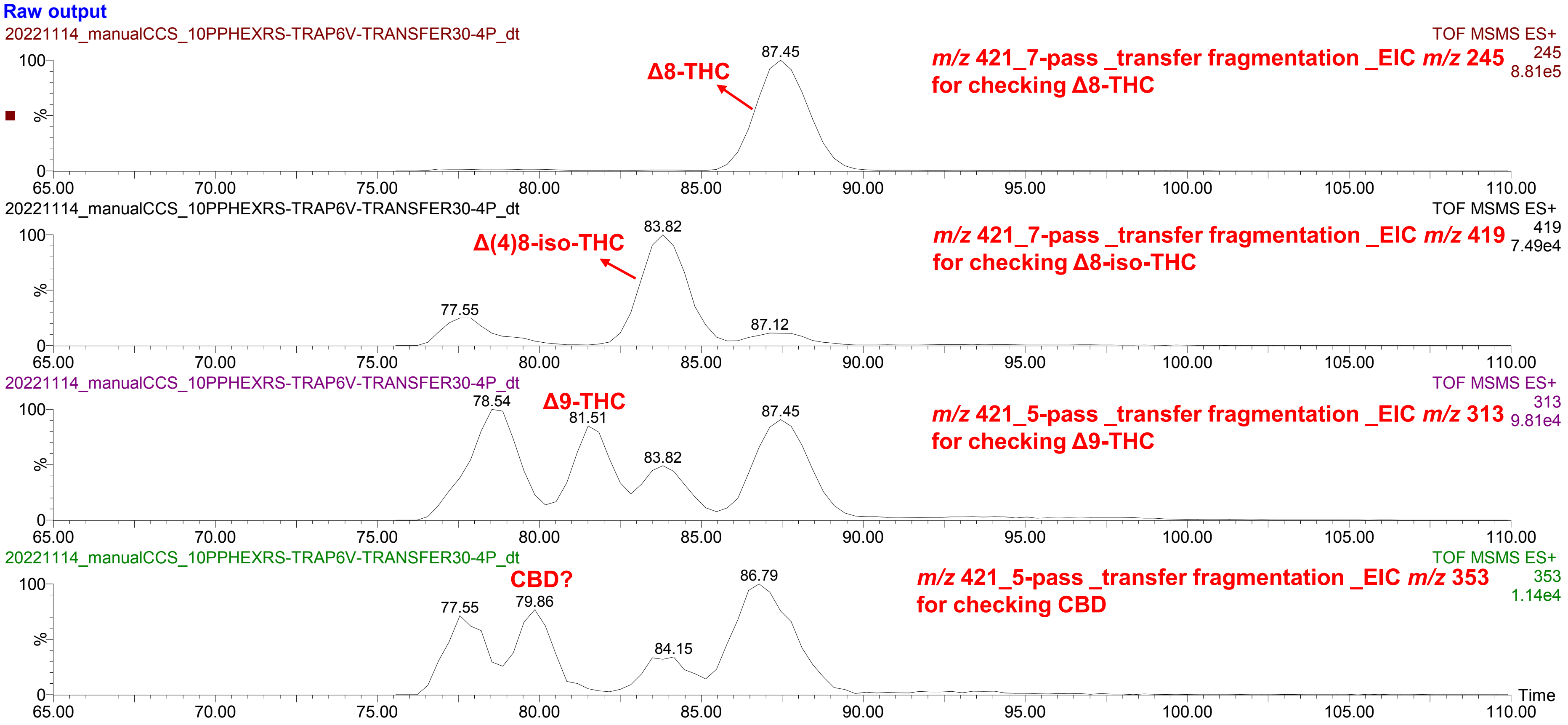

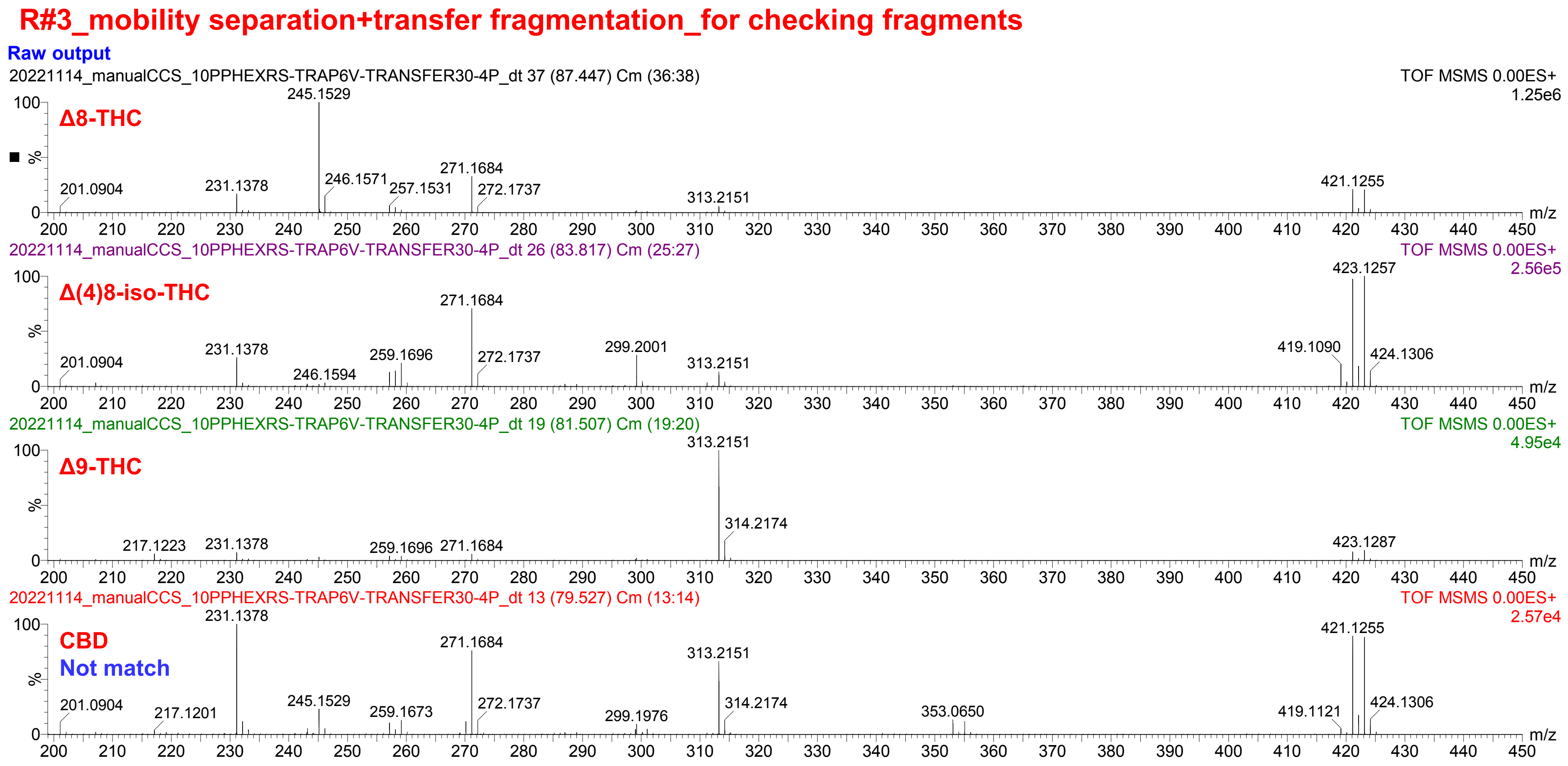

Figure S14-8. Mobiligram and mass spectra of cannabinoids in sample R#3.

R#4 _SIM_for checking Ag(I) adducts

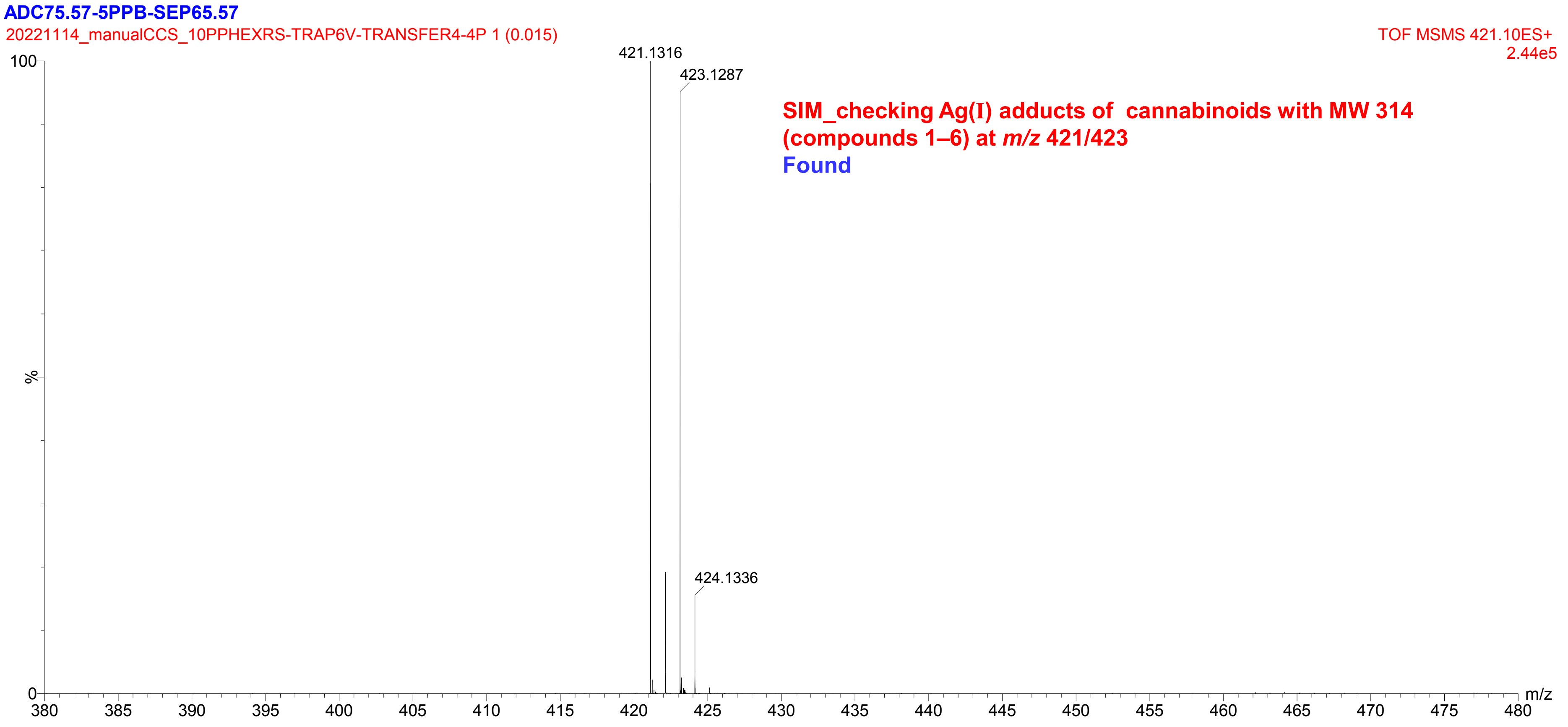

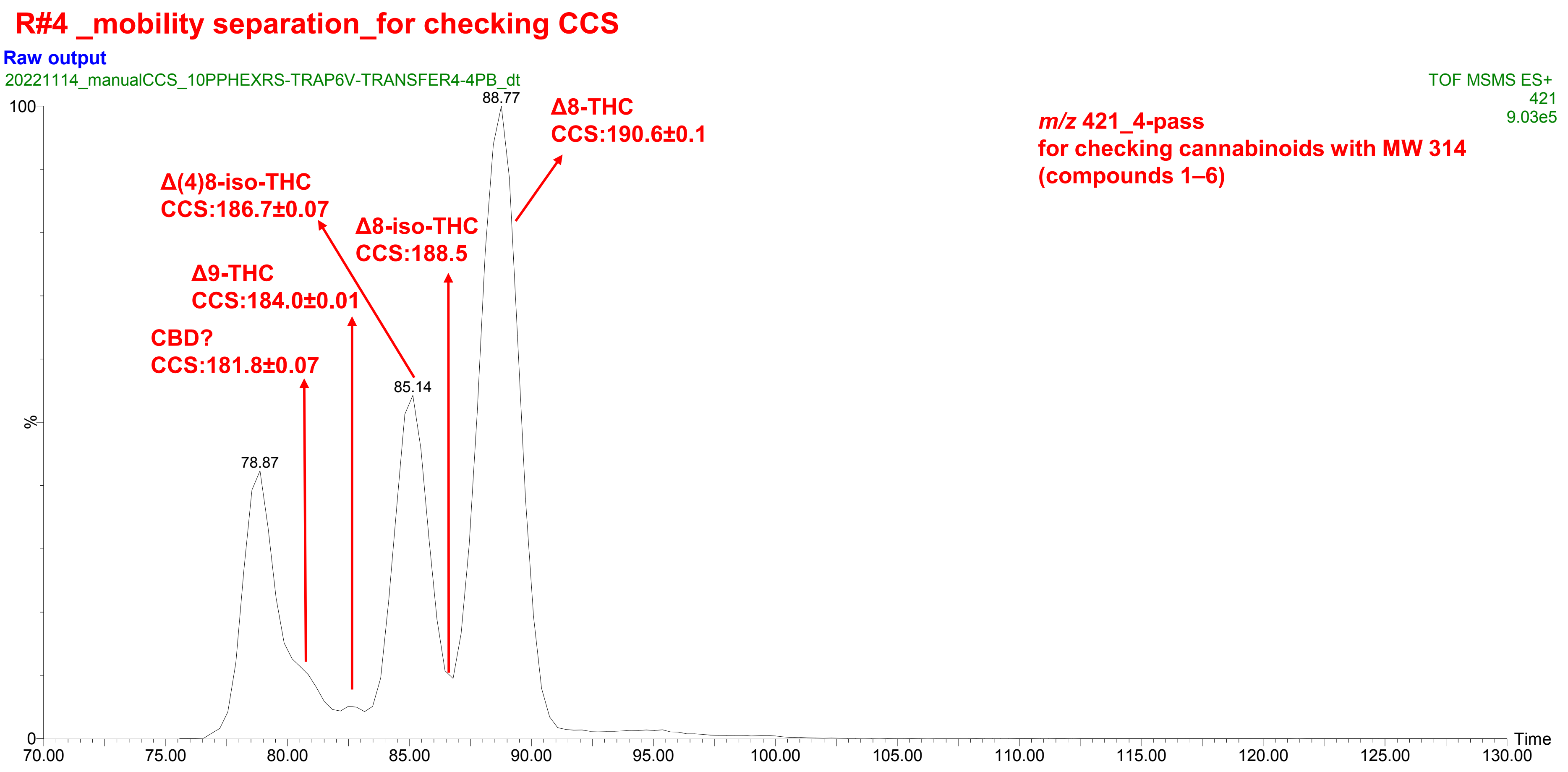
R#4 _mobility separation_for checking CCS
Raw output
20221114_manualCCS_10PPHEXRS-TRAP6V-TRANSFER4-4PB_dt
TOF MSMS ES+
421
9.03e5
m/z 421_4-pass
for checking cannabinoids with MW 314
(compounds 1–6)
Δ8-THC
CCS:190.6±0.1
Δ(4)8-iso-THC
CCS:186.7±0.07
Δ8-iso-THC
CCS:188.5
Δ9-THC
CCS:184.0±0.01
CBD?
CCS:181.8±0.07
88.77
85.14
78.87
100
%
0
70.00
75.00
80.00
85.00
90.00
95.00
100.00
105.00
110.00
115.00
120.00
125.00
130.00
Time

R#4_mobility separation+transfer fragmentation_for checking fragments

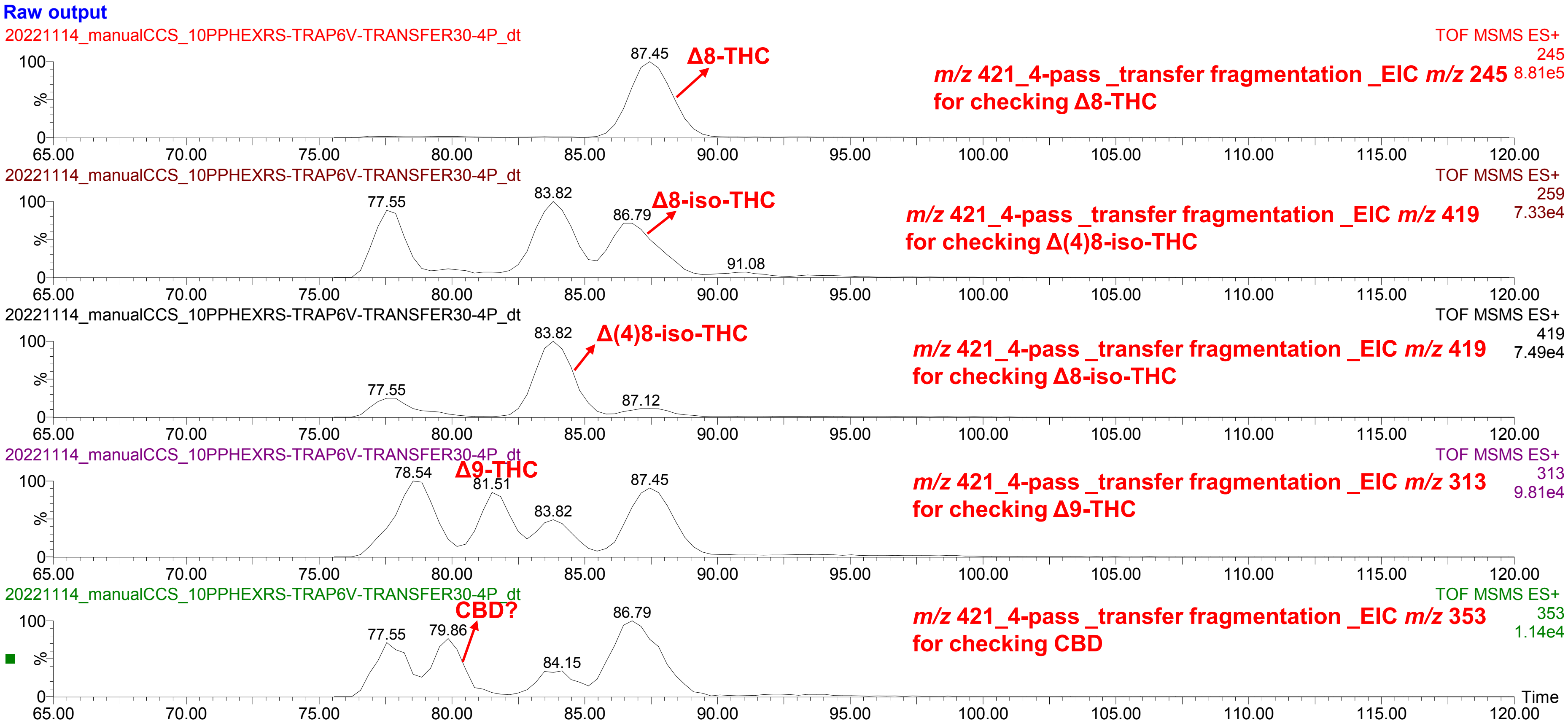

R#4_mobility separation+transfer fragmentation_for checking fragments

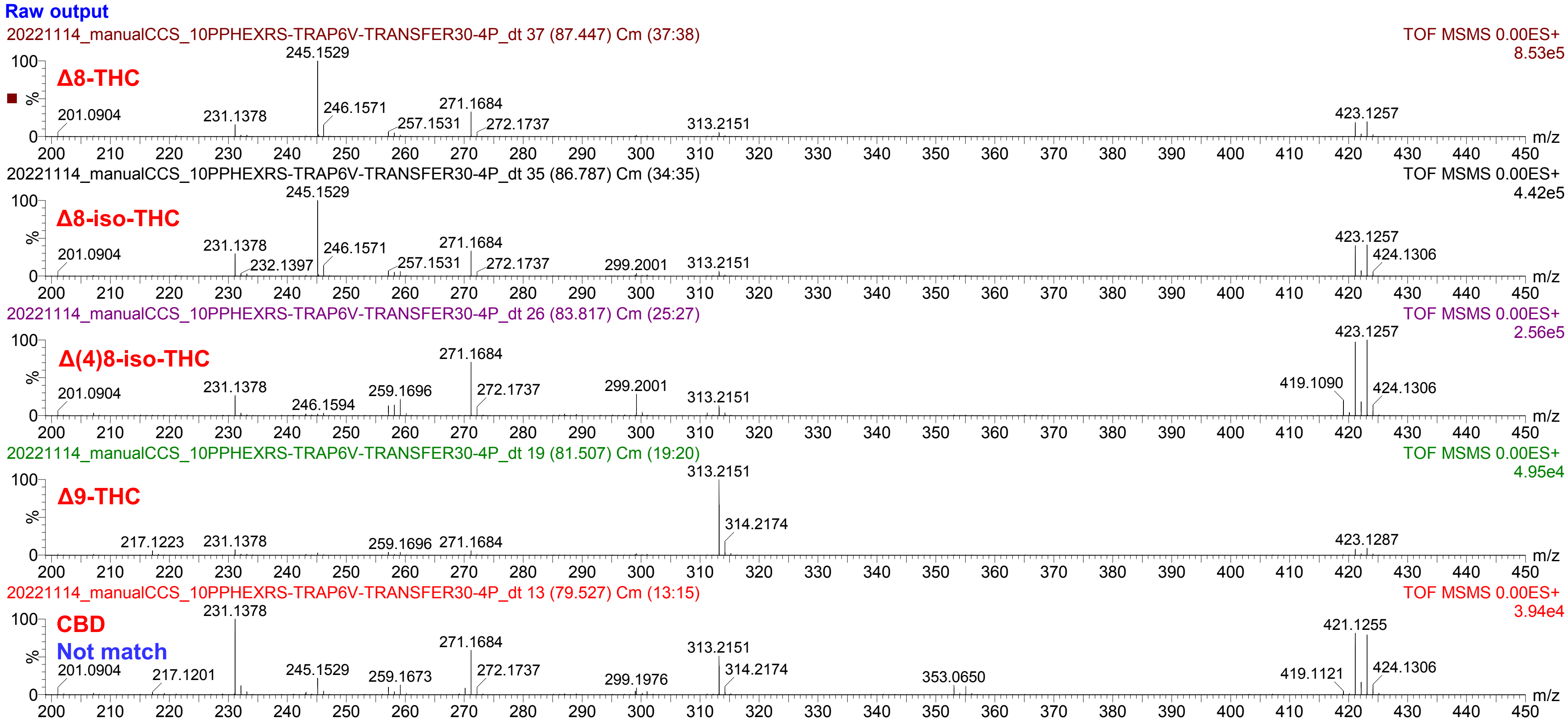

Figure S14-9. Mobiligram and mass spectra of cannabinoids in sample R#4.

R#5 _SIM_for checking Ag(I) adducts

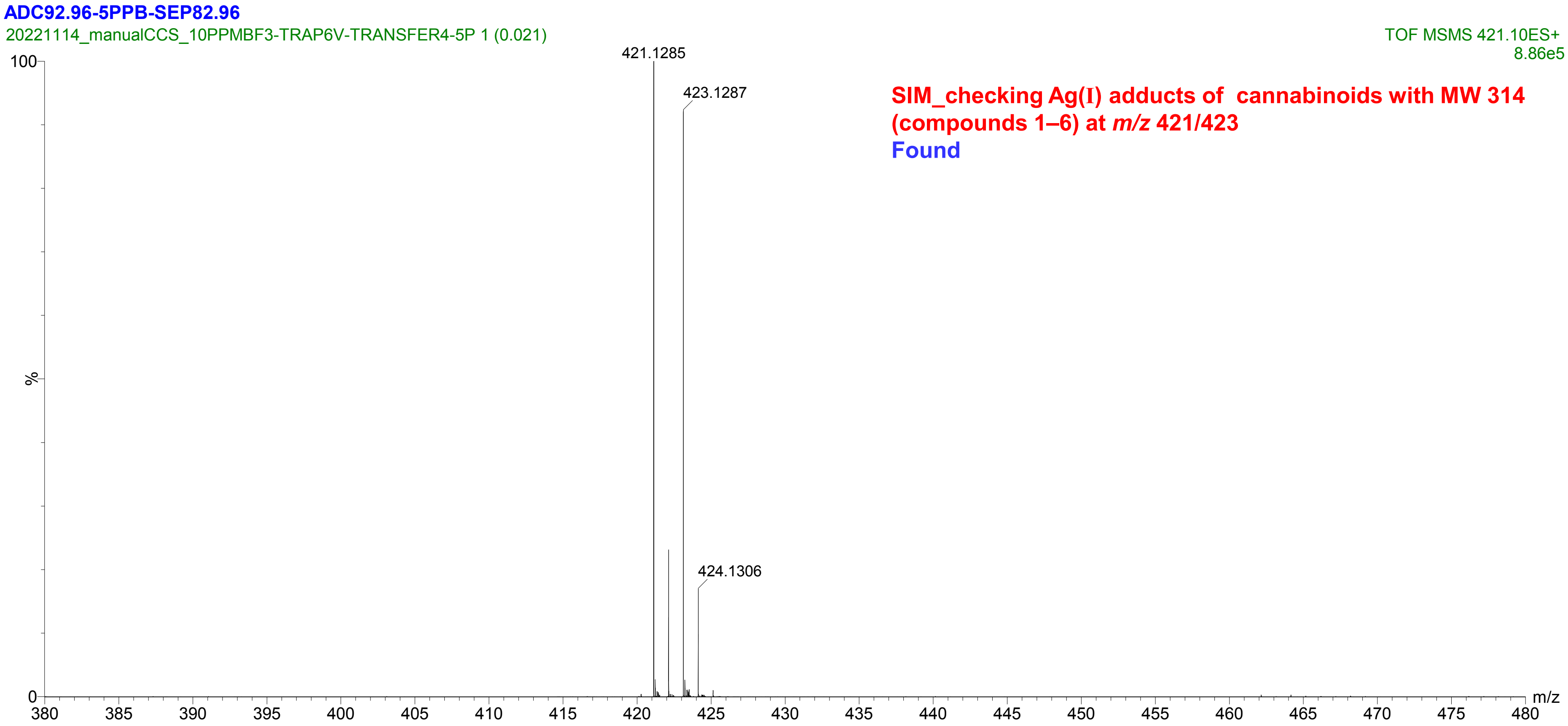

R#5 _mobility separation_for checking CCS

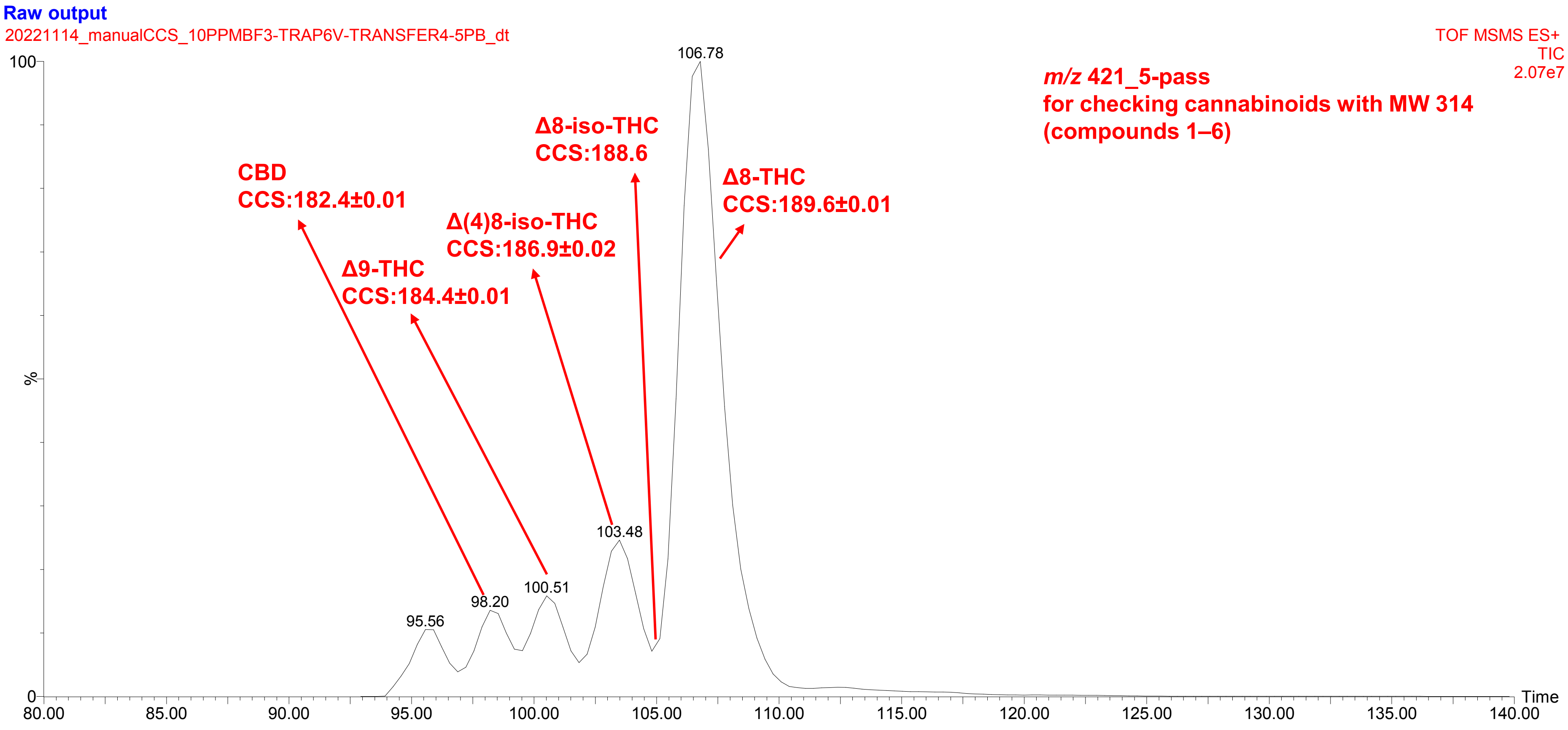

R#5_mobility separation+transfer fragmentation_for checking fragments

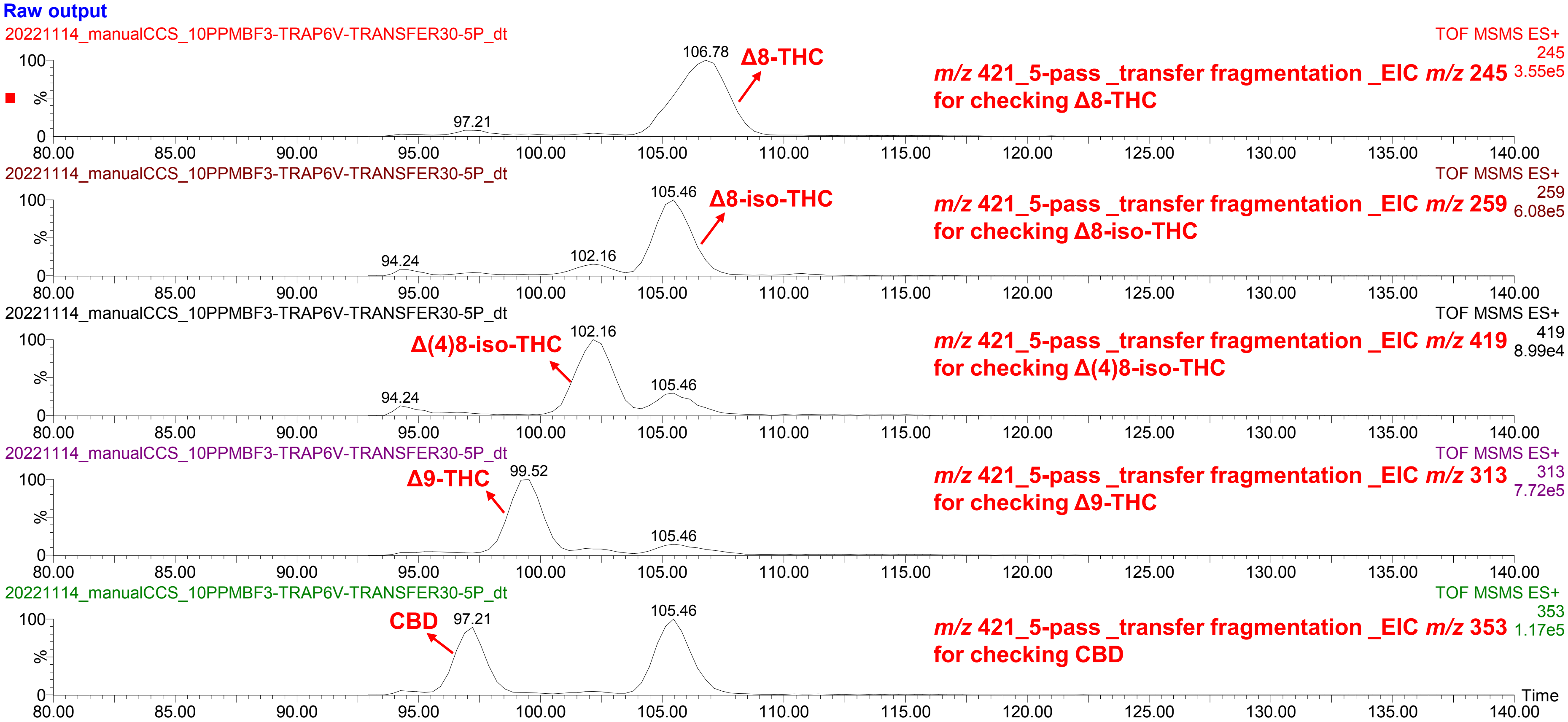

R#5_mobility separation+transfer fragmentation_for checking fragments

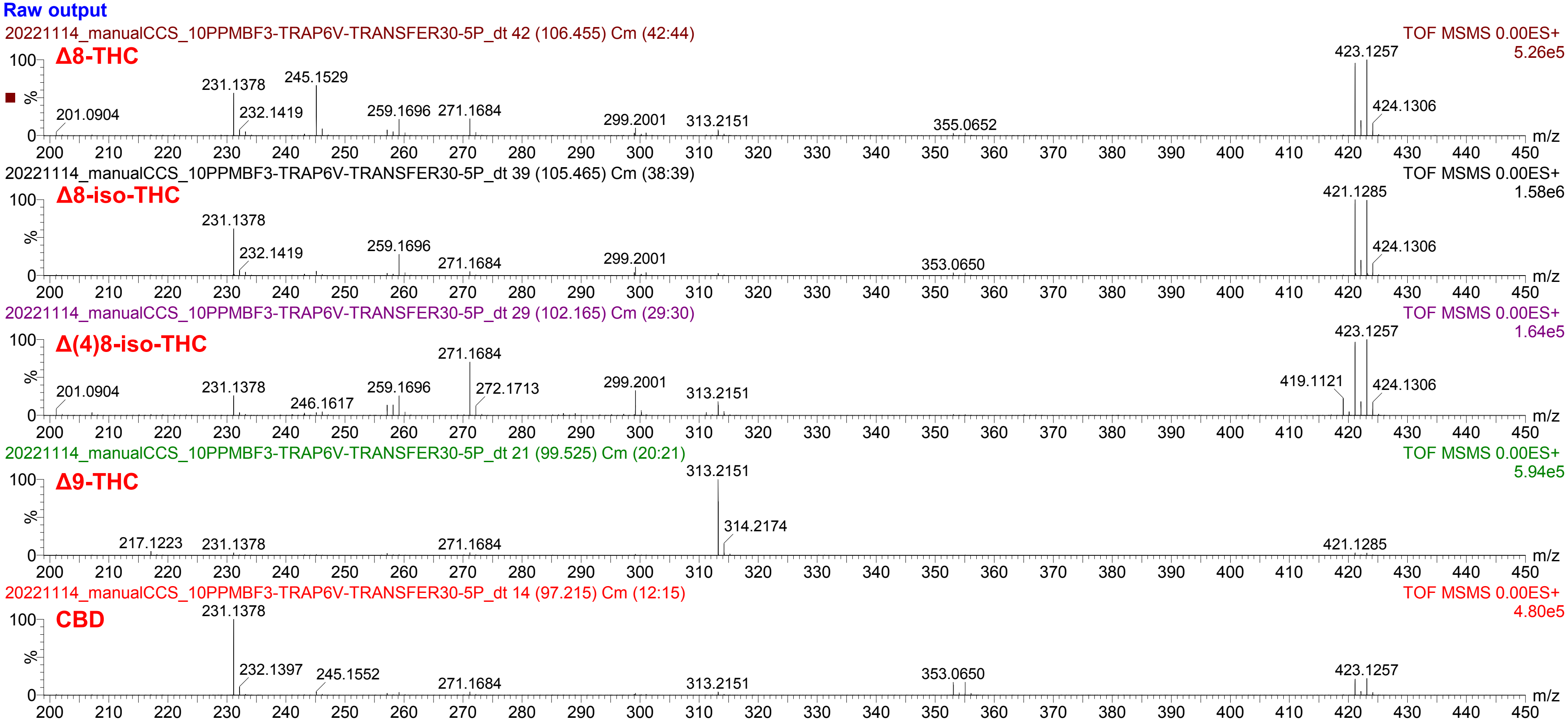

Figure S14-10. Mobiligram and mass spectra of cannabinoids in sample R#5.

R#6 _SIM_for checking Ag(I) adducts

Raw output

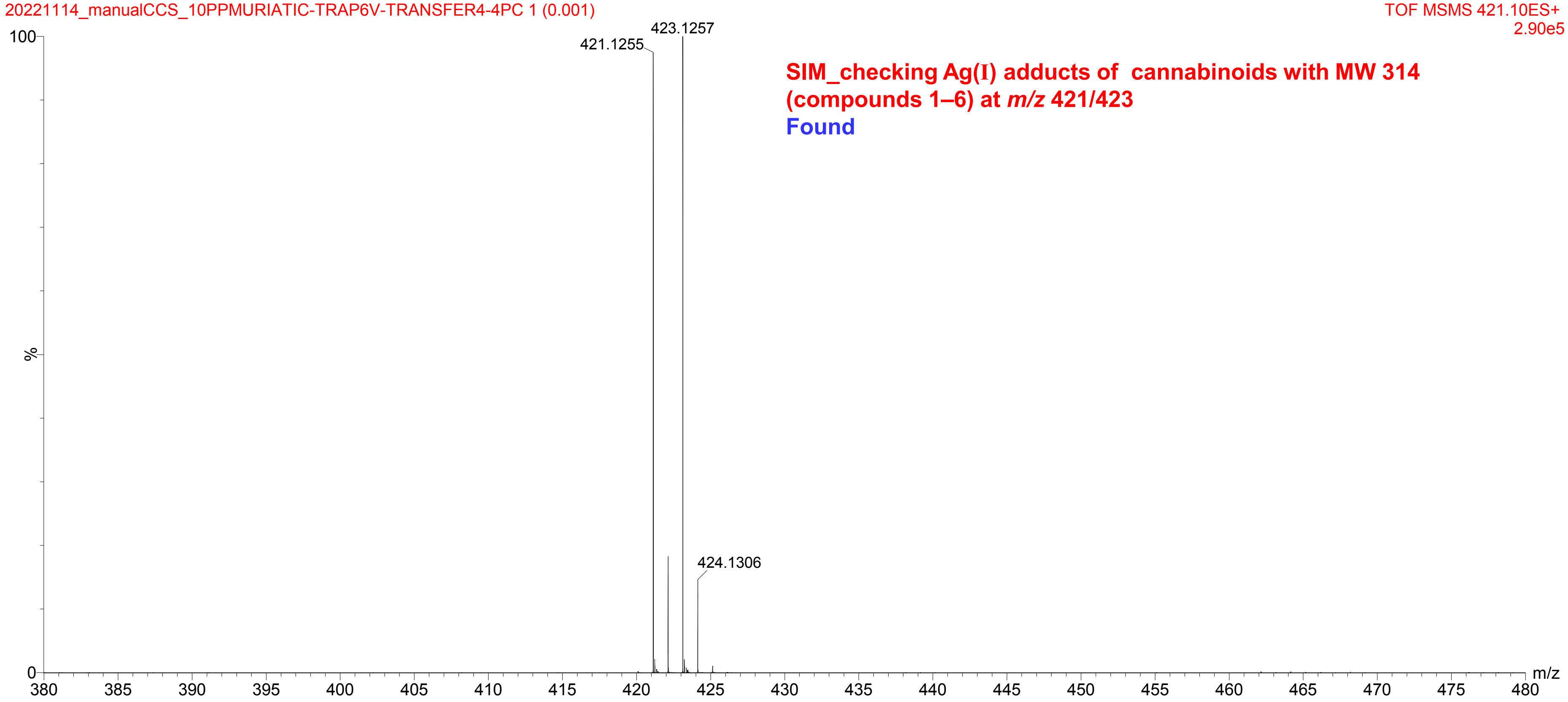

R#6 _mobility separation_for checking CCS

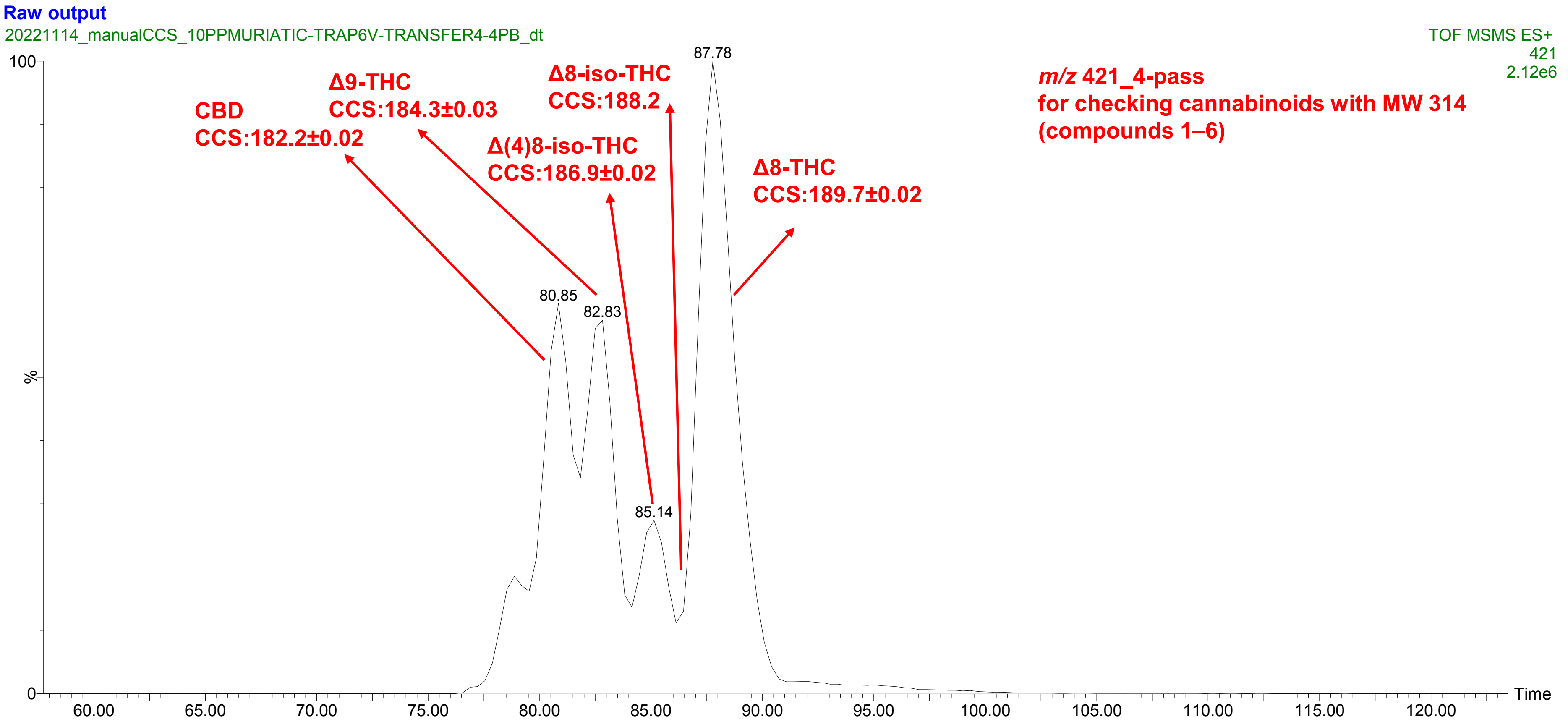

R#6_mobility separation+transfer fragmentation_for checking fragments

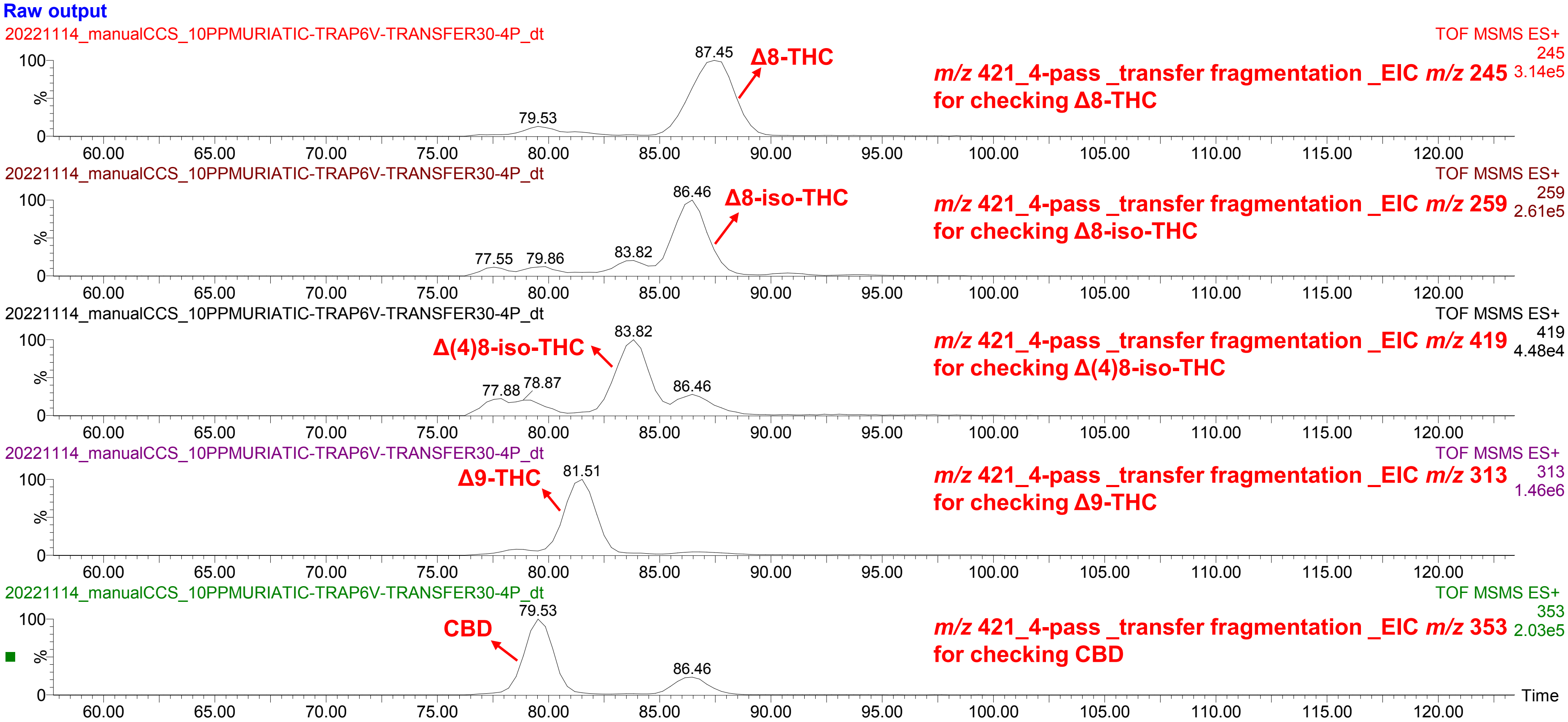

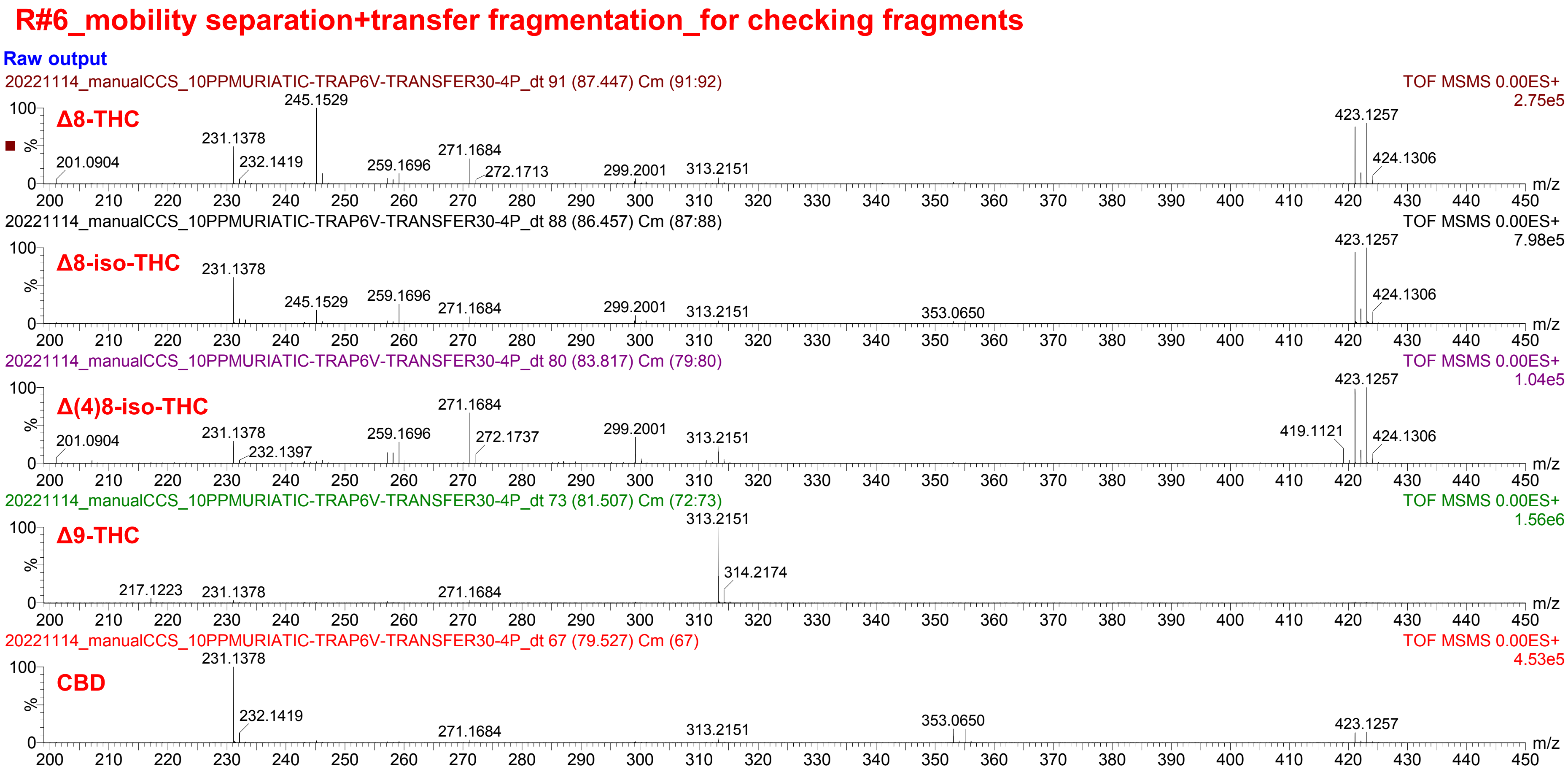

Figure S14-11. Mobiligram and mass spectra of cannabinoids in sample R#6.

R#7 _SIM_for checking Ag(I) adducts

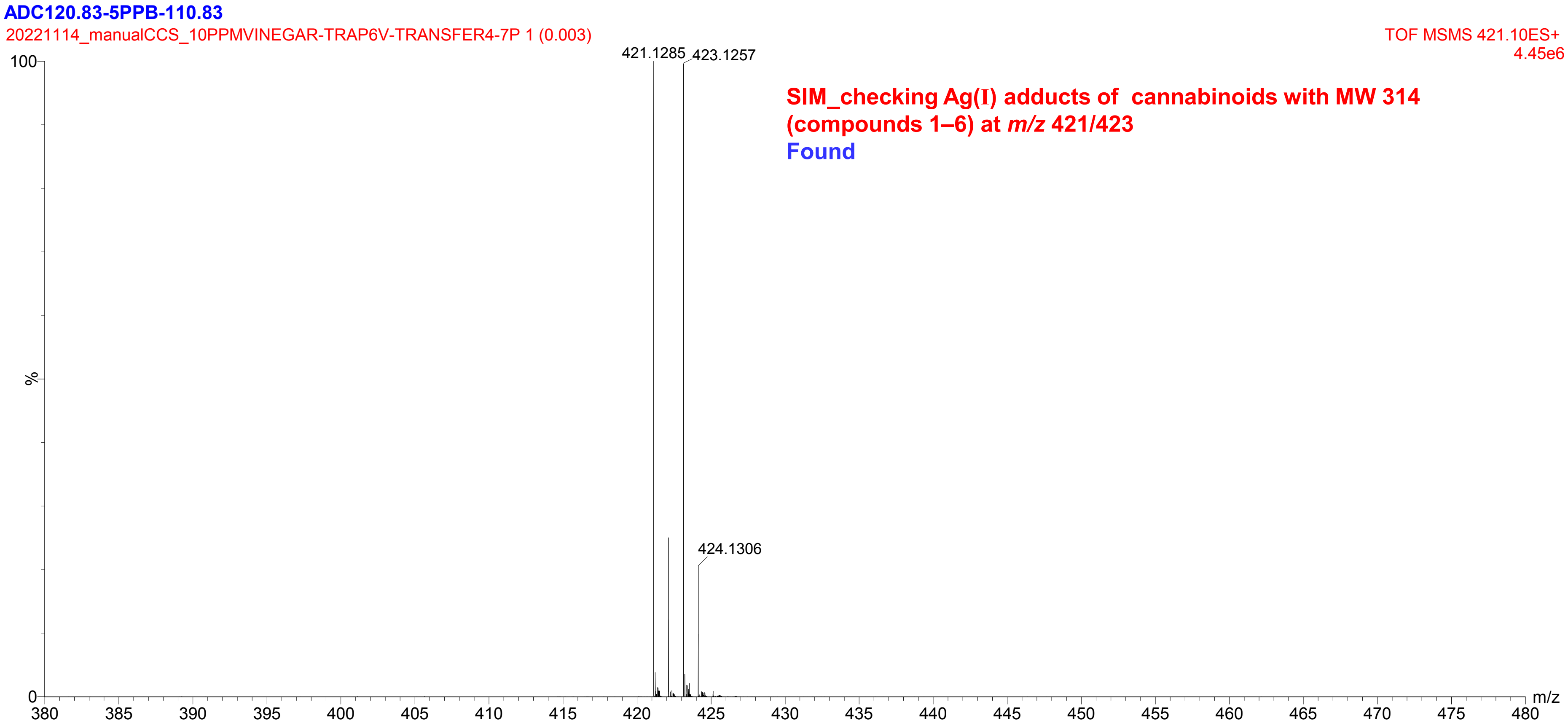

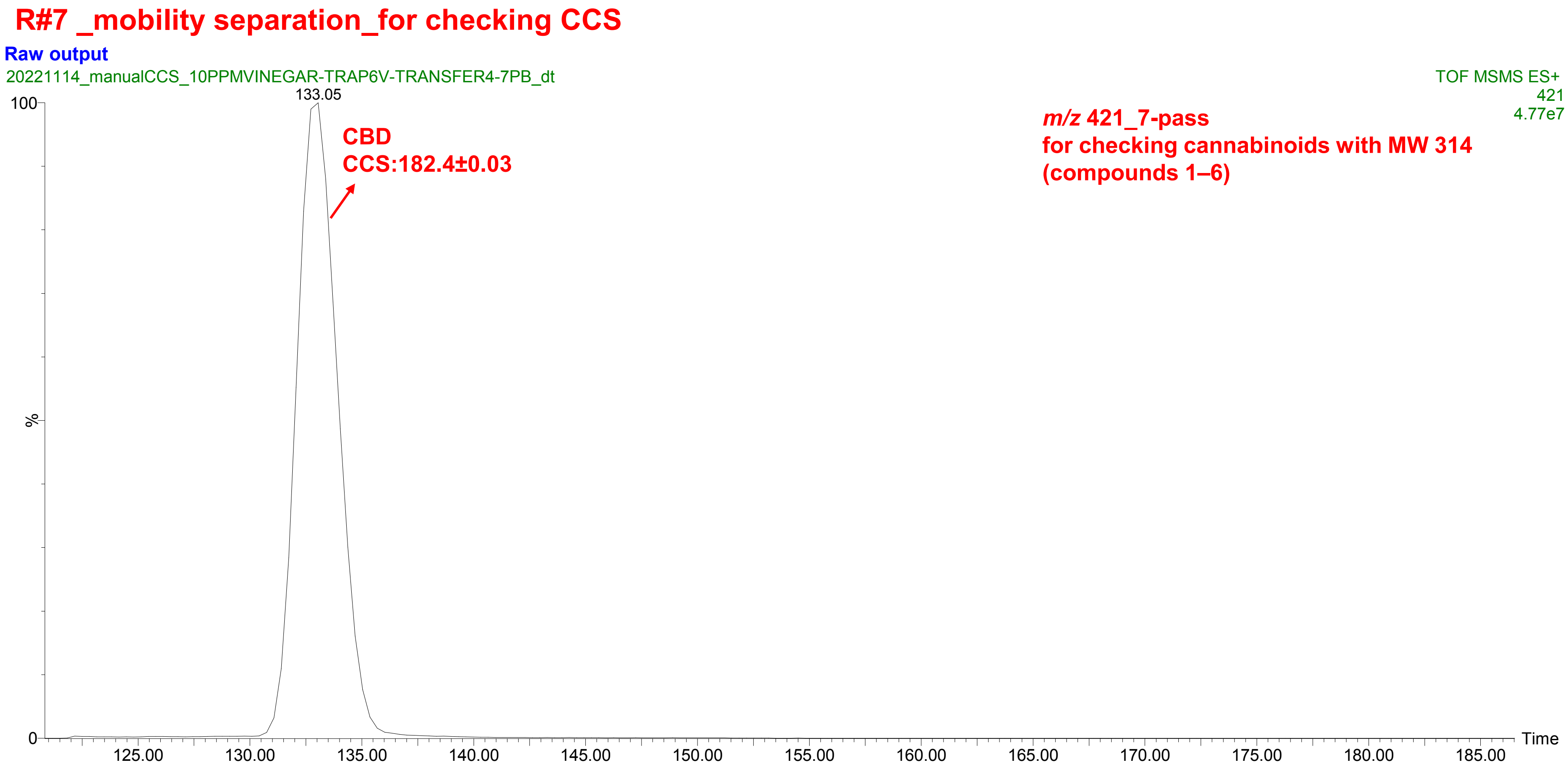
R#7 _mobility separation_for checking CCS
Raw output
20221114_manualCCS_10PPMVINEGAR-TRAP6V-TRANSFER4-7PB_dt
TOF MSMS ES+
421
4.77e7
m/z 421_7-pass
for checking cannabinoids with MW 314
(compounds 1–6)
133.05
CBD
CCS:182.4±0.03
100
%
0
125.00
130.00
135.00
140.00
145.00
150.00
155.00
160.00
165.00
170.00
175.00
180.00
185.00
Time

R#7_mobility separation+transfer fragmentation_for checking fragments

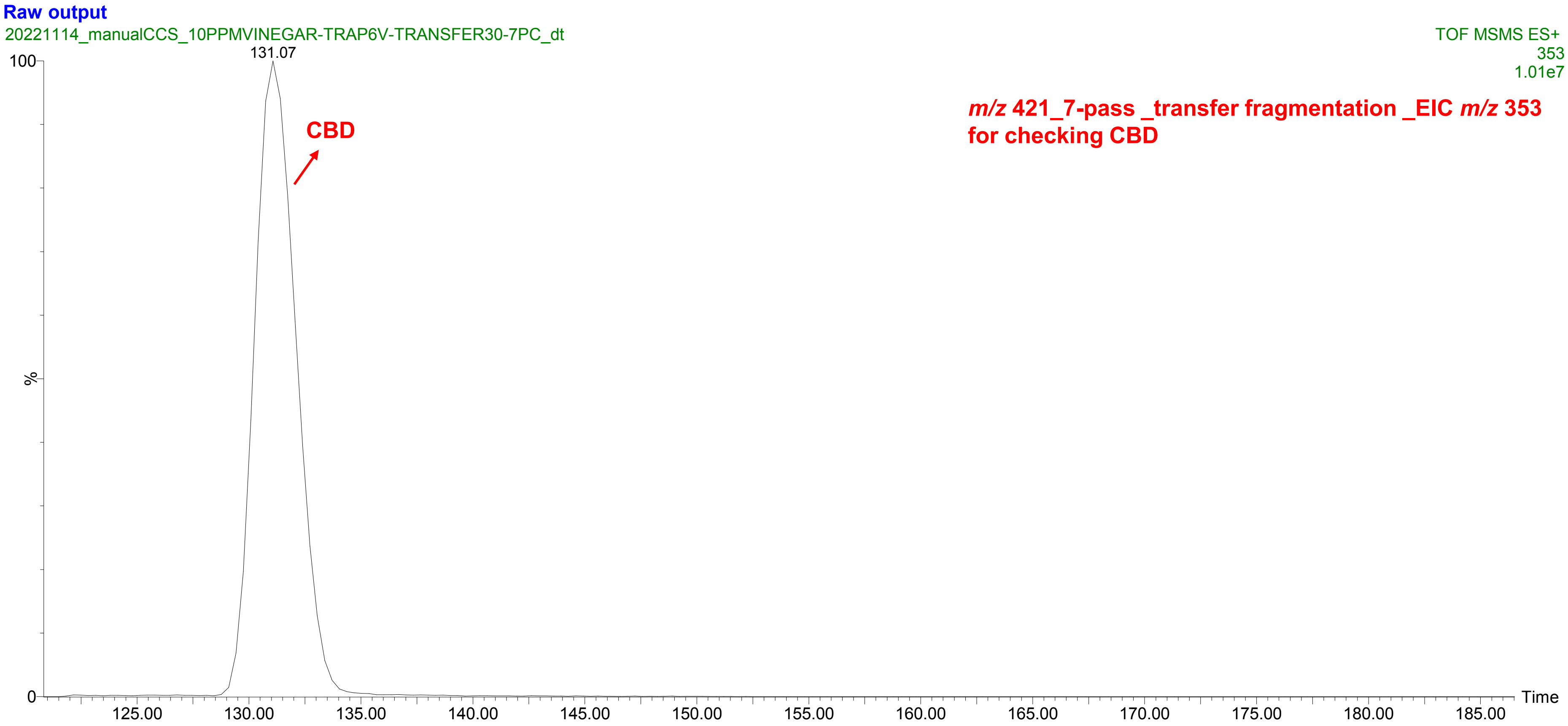

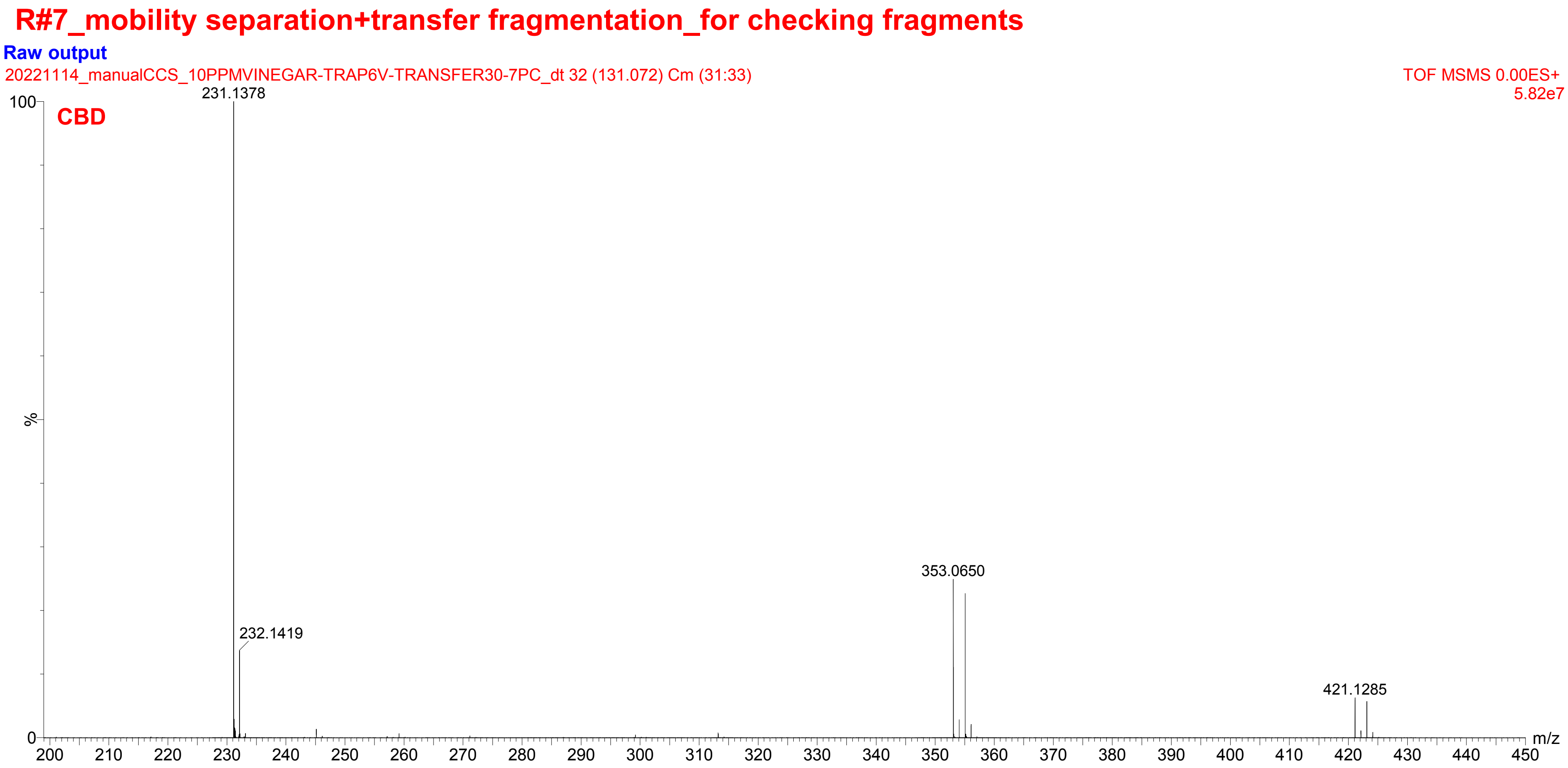

Figure S14-12. Mobiligram and mass spectra of cannabinoids in sample R#7.

R#8 _SIM_for checking Ag(I) adducts

Raw output

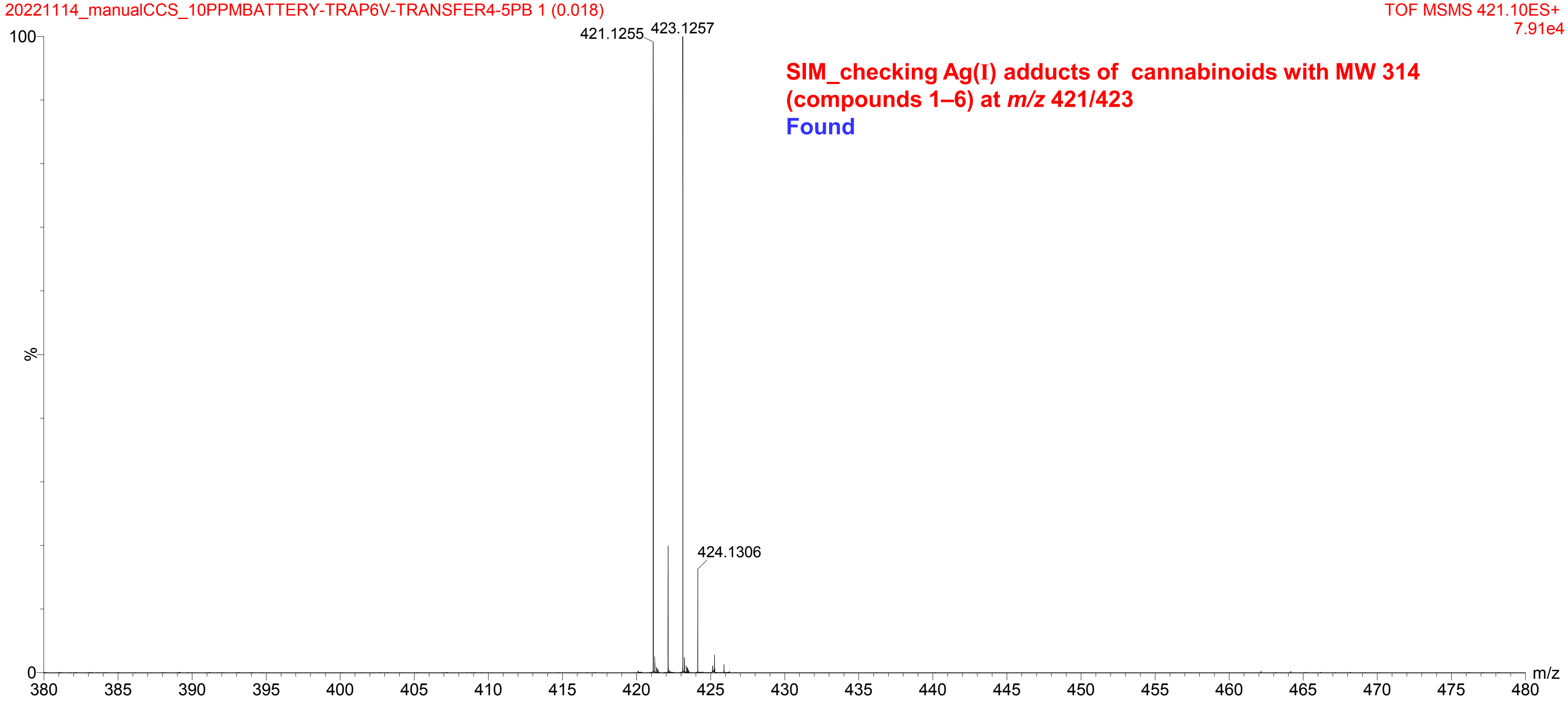

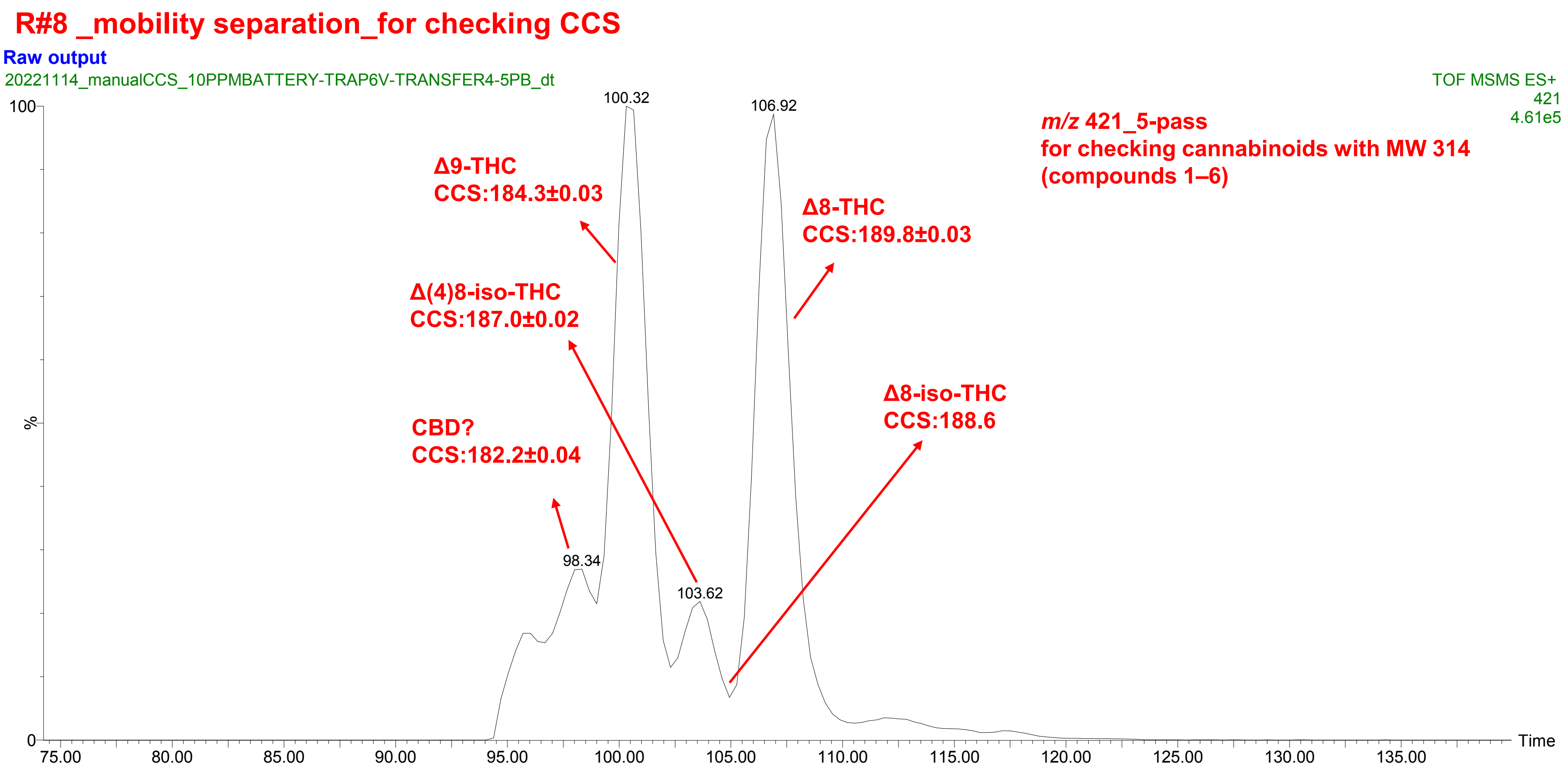
R#8 _mobility separation_for checking CCS
Raw output
20221114_manualCCS_10PPMBATTERY-TRAP6V-TRANSFER4-5PB_dt
TOF MSMS ES+
421
4.61e5
m/z 421_5-pass
for checking cannabinoids with MW 314
(compounds 1–6)
100.32
106.92
98.34
103.62
Δ9-THC
CCS:184.3±0.03
Δ8-THC
CCS:189.8±0.03
Δ(4)8-iso-THC
CCS:187.0±0.02
Δ8-iso-THC
CCS:188.6
CBD?
CCS:182.2±0.04
100
%
0
75.00
80.00
85.00
90.00
95.00
100.00
105.00
110.00
115.00
120.00
125.00
130.00
135.00
Time

R#8_mobility separation+transfer fragmentation_for checking fragments

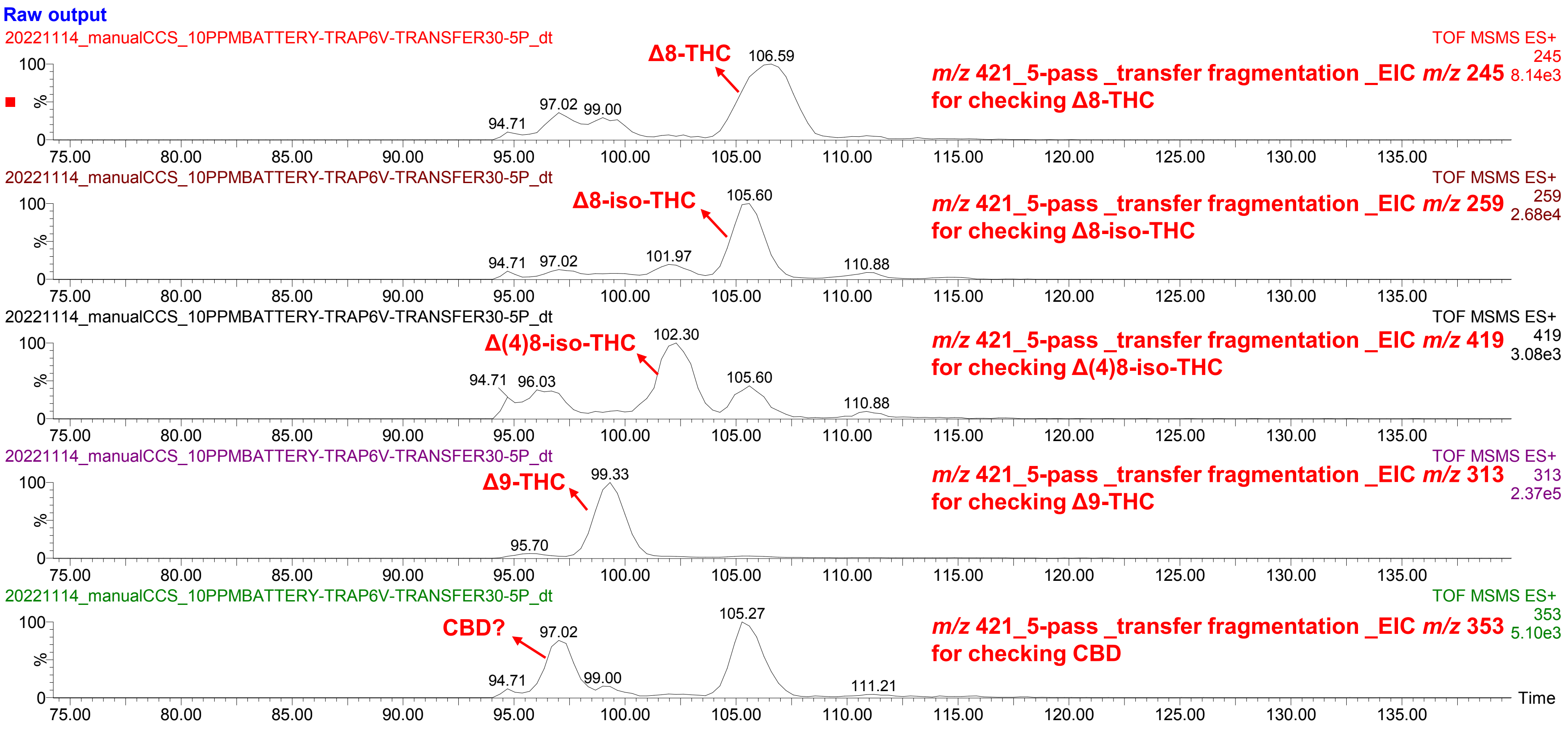

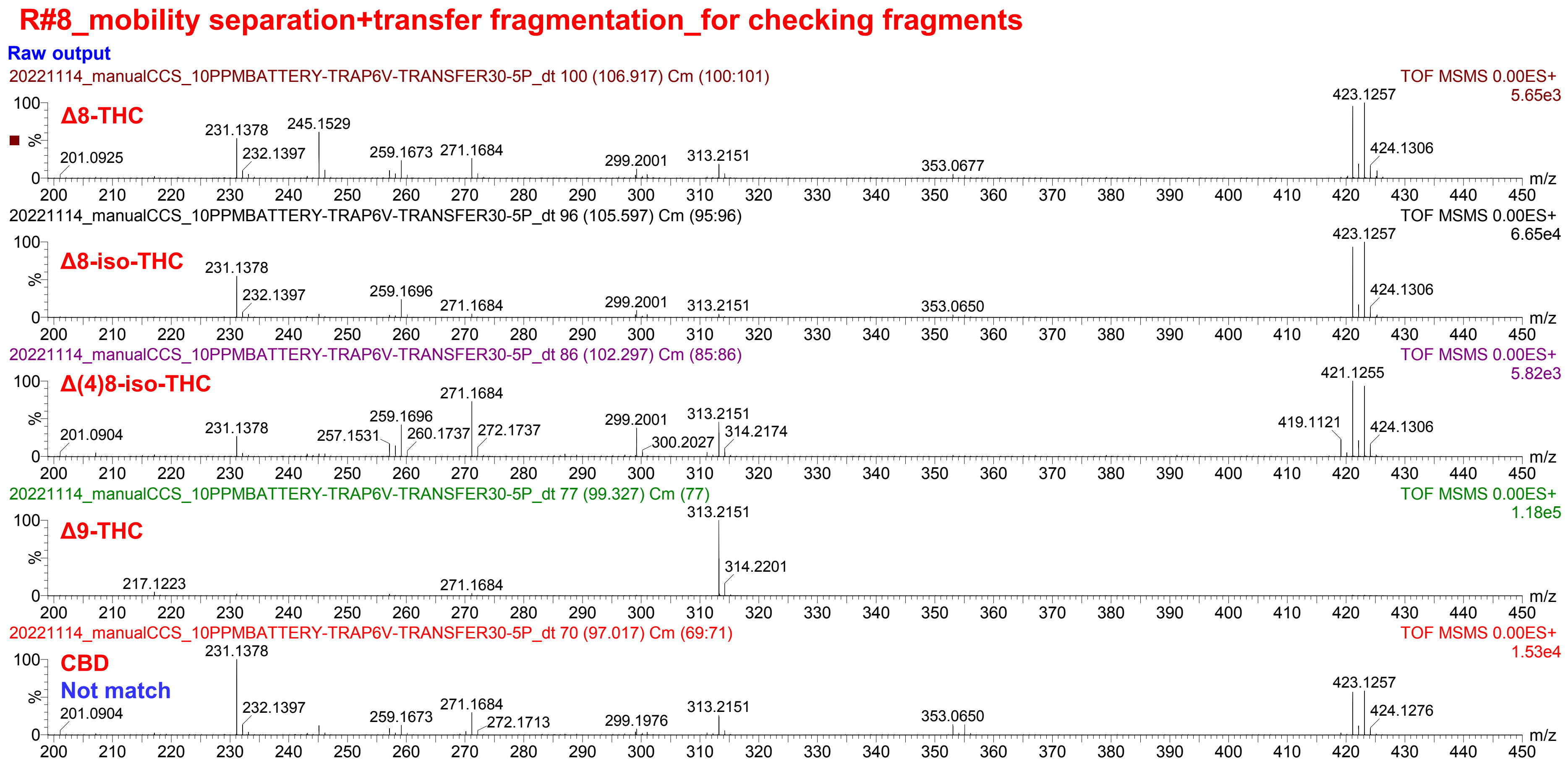

Figure S14-13. Mobiligram and mass spectra of cannabinoids in sample R#8.

Table S7. CCS values of detected cannabinoids in samples.

Sample	Δ8-THC	Δ9-THC	Δ3-THC	CBD	Δ8-iso-THC	Δ(4)8-iso-THC	THCA	CBDA	Δ8-THCV and Δ8-iso-THCV	Δ9-THCV	9α-hydroxyhexahydrocannabinol	9β-hydroxyhexahydrocannabinol	8-hydroxy-iso-THC
C#1	/	184.2±0.01 (0.2%)	/	182.1±0.01 (0.2%)	/	/	191.6±0.03 (0.4%)	189.2±0.01 (0.3%)	/	185.5 (0.3%)	/	/	/
C#2	/	184.4±0.01 (0.3%)	/	182.3±0.02 (0.3%)	/	/	192.1±0.01 (0.6%)	189.7±0.04 (0.6%)	/	185.1±0.01 (0.1%)	/	/	/
C#3	/	184.2±0.02 (0.2%)	/	/	/	/	191.6±0.02 (0.4%)	/	/	/	/	/	/
G#1	190.3±0.01 (−0.3%)	184.0±0.01 (0.05%)	/	/	/	186.5±0.02 (−0.3%)	/	/	/	/	/	/	/
G#2	190.9±0.08 (0)	184.4±0.04 (0.3%)	193.7±0.04 (0.4%)	/	/	187.1±0.03 (0)	/	/	/	/	196.0±0.03 (0.2%)	184.9±0.04 (−0.5%)	/
R#1	190.2±0.01 (−0.4%)	184.0±0.08 (0.05%)	/	181.1±0.07 (−0.4%)	188.9±0.1 (−0.3%)	/	NA	NA	NA	NA	NA	NA	NA
R#2	190.9±0.02 (0)	184.2±0.01 (0.2%)	/	182.1±0.02 (0.2%)	188.8* (−0.4%)	/	NA	NA	NA	NA	NA	NA	NA
R#3	190.1±0.2 (−0.4%)	184.1±0.09 (0.1%)	/	/	/	186.6±0.09 (−0.3%)	NA	NA	NA	NA	NA	NA	NA
R#4	190.6±0.1 (−0.2%)	184.0±0.01 (0.05%)	/	/	188.5* (−0.5%)	186.7±0.07 (−0.2%)	NA	NA	NA	NA	NA	NA	NA
R#5	189.6±0.01 (−0.7%)	184.4±0.01 (0.3%)	/	182.4±0.01 (0.3%)	188.6* (−0.5%)	186.9±0.02 (−0.1%)	NA	NA	NA	NA	NA	NA	NA
R#6	189.7±0.02 (−0.6%)	184.3±0.03 (0.2%)	/	182.2±0.02 (0.2%)	188.2* (−0.7%)	186.9±0.02 (−0.1%)	NA	NA	NA	N	NA	NA	NA
R#7	/	/	/	182.4±0.03 (0.3%)	/	/	NA	NA	NA	NA	NA	NA	NA
R#8	189.8±0.03 (−0.6%)	184.3±0.03 (0.2%)	/	/	188.6* (−0.5%)	187.0±0.02 (−0.05%)	NA	NA	NA	NA	NA	NA	NA

Values in brackets represent the relative deviation compared to the CCS values (${}^{TW}CCS_{N2}$ (Å^2)) from standards; ± SD (n=3); NA represents "not analyzed"; * represents the drift time decided by characteristic fragment due to the overlap of precursor ions.

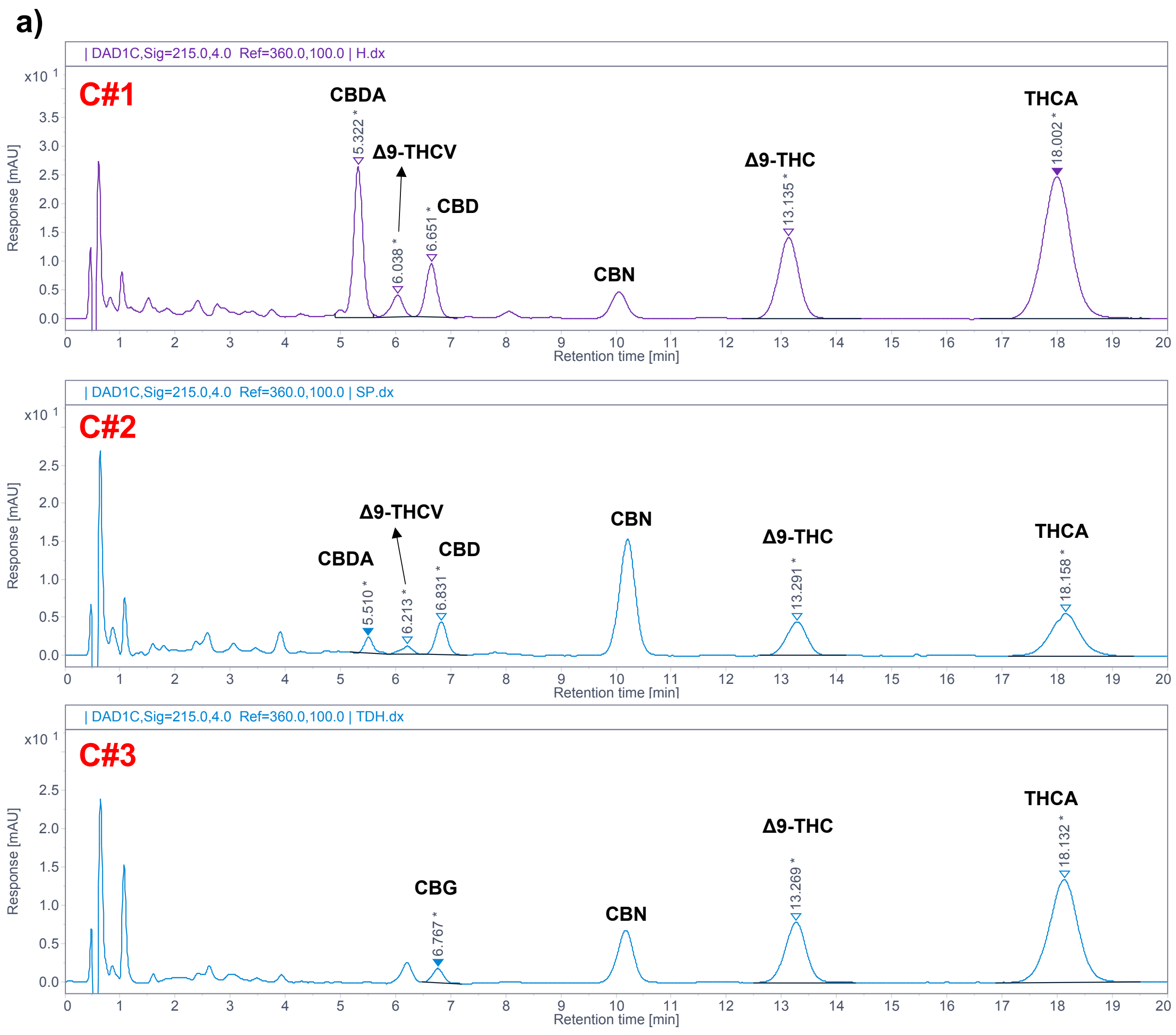

a)
| DAD1C,Sig=215.0,4.0 Ref=360.0,100.0 | H.dx
C#1
CBDA
Δ9-THCV
CBD
CBN
Δ9-THC
THCA
5.322 *
6.038 *
6.651 *
13.135 *
18.002 *
Response [mAU]
x10 1
Retention time [min]
| DAD1C,Sig=215.0,4.0 Ref=360.0,100.0 | SP.dx
C#2
CBDA
Δ9-THCV
CBD
CBN
Δ9-THC
THCA
5.510 *
6.213 *
6.831 *
13.291 *
18.158 *
Response [mAU]
x10 1
Retention time [min]
| DAD1C,Sig=215.0,4.0 Ref=360.0,100.0 | TDH.dx
C#3
CBG
CBN
Δ9-THC
THCA
6.767 *
13.269 *
18.132 *
Response [mAU]
x10 1
Retention time [min]

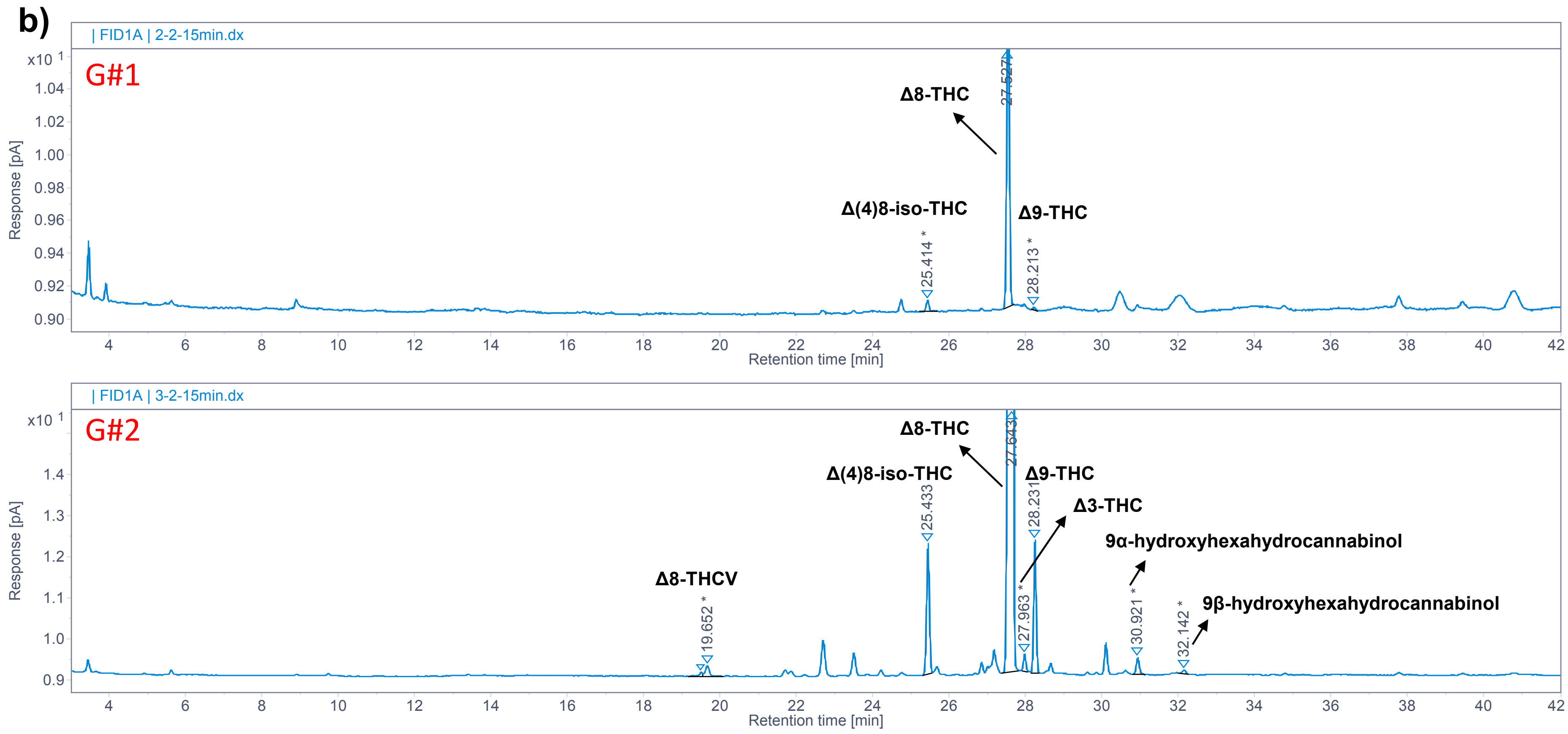

Figure S15. a) Reversed-phase UHPLC-UV (215 nm) profile of samples C#1, C#2, and C#3; b) GC-FID profile of samples G#1 and G#2.

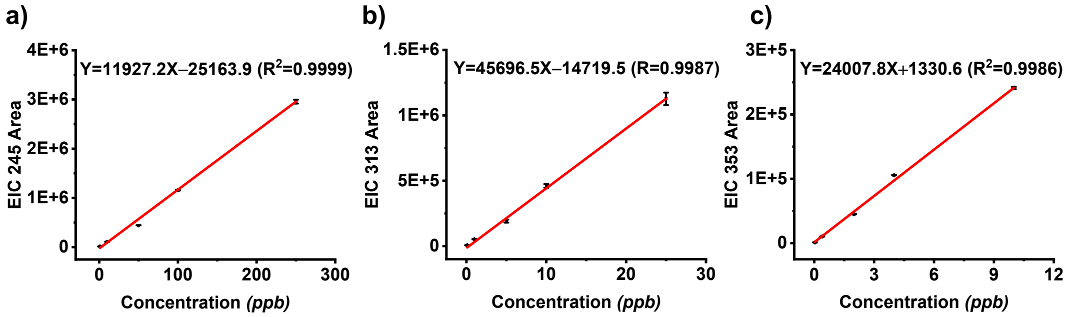

Figure S16. Calibration curves between the extracted ion chromatogram peak area of characteristic fragments and concentrations of a) Δ8-THC, b) Δ9-THC, and c) CBD. Error bars represent the standard deviation (n=3).

Table S8. Absolute weight percentages (w/w%, after solvent evaporation) of Δ8-THC, Δ9-THC, and CBD in acid-treated CBD mixtures by cIMS and GC-FID.

Sample	Δ8-THC			Δ9-THC			CBD		
	GC-FID	cIMS	Deviation	GC-FID	cIMS	Deviation	GC-FID	cIMS	Deviation
R#1	11 (2.6%)	15 (0.4%)	36%	49 (0.3%)	70 (0.4%)	43%	26 (2.0%)	36 (0.2%)	39%
R#2	26 (4.0%)	30 (3.2%)	15%	48 (0.1%)	61 (3.2%)	27%	6.9 (0.4%)	6.6 (3.1%)	−4%
R#3	78 (2.1%)	115 (0.9%)	47%	1.6 (1.1%)	2.4 (0.3%)	50%	ND[b]	ND[b]	/
R#4	70 (1.8%)	96 (1.3%)	37%	1.1 (5.6%)	2.9 (1.1%)	164%	ND[b]	ND[b]	/
R#5	14 (3.7%)	23 (1.1%)	64%	6.3 (5.2%)	11 (0.9%)	75%	ND	0.6 (2.2%)	/
R#6	8.1 (0.5%)	13 (0.7%)	61%	8.9 (1.2%)	14 (1.2%)	57%	4.2 (0.2%)	1.2 (1.8%)	−71%
R#7	ND	ND	/	ND	ND	/	48 (0.7%)	59 (0.5%)	23%
R#8	1.4 (1.1%)	3.1 (1.3%)	121%	5.0 (2.5%)	6.7 (1.0%)	34%	ND	ND	/

All values are average of triplicate experiments; ND: Not detected (<LOD).

Table S9. Ratio of Δ9-THC/Δ8-THC in acid-treated CBD mixtures analyzed by cIMS and GC-FID.

Sample	Δ8-THC		Δ9-THC		Δ9-THC/Δ8-THC ratio	
	GC-FID	cIMS	GC-FID	cIMS	GC-FID	cIMS
R #1	11 (2.6%)	15 (0.4%)	49 (0.3%)	70 (0.4%)	4.5	4.7
R #2	26 (4.0%)	30 (3.2%)	48 (0.1%)	61 (3.2%)	1.8	2.0
R #3	78 (2.1%)	115 (0.9%)	1.6 (1.1%)	2.4 (0.3%)	0.02	0.02
R #4	70 (1.8%)	96 (1.3%)	1.1 (5.6%)	2.9 (1.1%)	0.02	0.03
R #5	14 (3.7%)	23 (1.1%)	6.3 (5.2%)	11 (0.9%)	0.45	0.48
R #6	8.1 (0.5%)	13 (0.7%)	8.9 (1.2%)	14 (1.2%)	1.1	1.1
R #7	ND	ND	ND	ND	/	/
R #8	1.4 (1.1%)	3.1 (1.3%)	5.0 (2.5%)	6.7 (1.0%)	3.6	2.2

All values are average of triplicate experiments; ND: Not detected (<LOD).

Table S10. Comparison of LODs between the cIMS method and GC-FID method[1]

	cIMS	GC-FID
Injection volume	5 μL	1 μL
split ratios	NA	Split ratio for injector 1:10, split ratio for detector 1:1.
LOD expressed as concentration	0.2 ng·ml^{-1} for Δ8-THC, 0.04 ng·ml^{-1} for Δ9-THC, and 0.008 ng·ml^{-1} for CBD.	0.3 μg·ml^{-1} for Δ8-THC, 0.3 μg·ml^{-1} for Δ9-THC, and 1.3 μg·ml^{-1} for CBD.
LOD expressed as absolute mass	1 pg for Δ8-THC, 0.2 pg for Δ9-THC, and 0.04 pg for CBD.	15 pg for Δ8-THC, 15 pg for Δ9-THC, and 65 pg for CBD.

REFERENCES

[1] Huang, S.; van Beek, T. A.; Claassen, F. W.; Janssen, H.-G.; Ma, M.; Chen, B.; Zuilhof, H.; Salentijn, G. I. J. Comprehensive cannabinoid profiling of acid-treated CBD samples and Δ8-THC-infused edibles. *Food Chem.* **2023,** 138187.
[2] Cheng, L.-J.; Xie, J.-H.; Chen, Y.; Wang, L.-X.; Zhou, Q.-L. Enantioselective Total Synthesis of (−)-Δ8-THC and (−)-Δ9-THC via Catalytic Asymmetric Hydrogenation and SNAr Cyclization. *Org. Lett.* **2013**, *15*, 764-767.
[3] Srebnik, M.; Lander, N.; Breuer, A.; Mechoulam, R. Base-catalysed double-bond isomerizations of cannabinoids: structural and stereochemical aspects. *J. Chem. Soc., Perkin Trans* **1984**, 2881-2886.
[4] Dadiotis, E.; Mitsis, V.; Melliou, E.; Magiatis, P. Direct Quantitation of Phytocannabinoids by One-Dimensional 1H qNMR and Two-Dimensional 1H-1H COSY qNMR in Complex Natural Mixtures. *Molecules* **2022**, *27*, 2965.
[5] Radwan, M. M.; Wanas, A. S.; Gul, W.; Ibrahim, E. A.; ElSohly, M. A. Isolation and Characterization of Impurities in Commercially Marketed Δ8-THC Products. *J. Nat. Prod.* **2023**, *86*, 822-829.
[6] Hädener, M.; Kamrath, M. Z.; Weinmann, W.; Groessl, M. High-Resolution Ion Mobility Spectrometry for Rapid Cannabis Potency Testing. *Anal. Chem.* **2018**, *90*, 8764-8768.
[7] Zietek, B. M.; Mengerink, Y.; Jordens, J.; Somsen, G. W.; Kool, J.; Honing, M. Adduct-ion formation in trapped ion mobility spectrometry as a potential tool for studying molecular structures and conformations. *Int. J. Ion Mobil. Spectrom.* **2018**, *21*, 19-32.
